# Massive barcode-free chemical screenings enable the discovery of bioactive macrocycles with passive membrane permeability

**DOI:** 10.1038/s41467-026-71641-3

**Published:** 2026-04-10

**Authors:** J. Miguel Mata, Jingming Liu, Sean M. McKenna, Edith van der Nol, Marije Havermans, Ruud Delwel, Mike Filius, Chirlmin Joo, Maura Vallaro, Giulia Caron, Sebastian J. Pomplun

**Affiliations:** 1 LACDR, Leiden University, Leiden, 2333 CC The Netherlands; 2 Oncode Institute, Utrecht, 3521 AL The Netherlands; 3 Department of Hematology, Erasmus MC Cancer Institute, Rotterdam, Netherlands; 4 Kavli Institute of NanoScience, Department of BioNanoScience, Delft University of Technology, Delft, 2628 CJ The Netherlands; 5 Department of Physics, Ewha Womans University, Seoul, 03760 Republic of Korea; 6 Molecular Biotechnology and Health Sciences Department., CASSMedChem, University of Torino, via Quarello 15, 10135 Torino, Italy

**Keywords:** Drug discovery and development, Peptides, Combinatorial libraries

## Abstract

Synthetic macrocycles offer exceptional potential as therapeutics. However, most high-throughput discovery platforms rely on genetically encoded libraries of large peptide macrocycles, which typically are not optimized for drug like properties. Fully synthetic libraries offer greater flexibility in accessing broader chemical space. Leveraging recent advances in mass spectrometry based library techniques, here we report CycloSEL (Cyclic Self-Encoded Libraries), an end-to-end workflow, that screens synthetic macrocycle libraries enriched in drug-like ‘beyond rule of five’ features. The workflow relies on affinity selections and hit identification by tandem mass spectrometry, eliminating the need for genetic barcodes. We construct a 16 million-member library and validate the approach against the oncology target carbonic anhydrase IX, achieving robust enrichment and accurate identification of true binders. Applying CycloSEL to the acute myeloid leukemia target WD repeat-containing protein 5 (WDR5) yields a macrocycle with subnamolar affinity, and potent inhibition of the WDR5–Mixed-Lineage Leukemia 1 (MLL1) interaction. Subsequent modifications produce a chameleonic macrocycle with passive membrane permeability, serum stability, and anti-proliferative activity in leukemia cells. Together, these results demonstrate that CycloSEL enables discovery of drug-like macrocycles from fully synthetic libraries for intracellular targets.

## Abstract (web summary)

Synthetic macrocycles are promising therapeutics; however, most high-throughput discovery platforms rely on genetically encoded libraries of large peptide macrocycles, which are typically not optimized for drug-like properties. Here, the authors report CycloSEL (Cyclic Self-Encoded Libraries), an end-to-end workflow that screens synthetic macrocycle libraries enriched in drug-like ‘beyond rule of five’ features, based on affinity selections and hit identification by tandem mass spectrometry.

## Introduction

Macrocycles have emerged as powerful therapeutic modalities capable of targeting challenging biomolecular interactions^1–8^. Traditionally, drug discovery has focused on small molecules that adhere to Lipinski’s rule of five, since these compounds generally exhibit favorable absorption and cell permeability. However, challenging target classes, such as disease-relevant protein–protein interactions (PPIs), have required exploring chemical space “beyond the rule of five” (bRo5)^9^. Macrocycles hold a prominent role in this trend. Their larger size, compared to typical small-molecule drugs, allows them to inhibit shallow and extended protein–protein interactions^2,10–13^. Furthermore, their conformationally constrained cyclic structures grant them advantageous properties over flexible bRo5 compounds, often resulting in higher binding affinities, specificities and improved stability^2^.

Nevertheless, achieving adequate ADME (absorption, distribution, metabolism, and excretion) characteristics remains a major challenge for all bRo5 compounds, including macrocycles. To guide the design of “drug-like” macrocycles within beyond-Ro5 space, Kihlberg and colleagues established heuristic criteria based on large datasets of known compounds^1,14–16^. They proposed that bRo5 macrocycles with molecular weight below 1000 Da, fewer than six hydrogen-bond donors, cLogP between −2 and 10, and a polar surface area under 250 Å² are more likely to possess favorable physicochemical profiles. While each new compound must still be assessed experimentally, these guidelines help prioritize scaffolds with a higher probability of success in drug discovery campaigns.

**Fig. 1:**
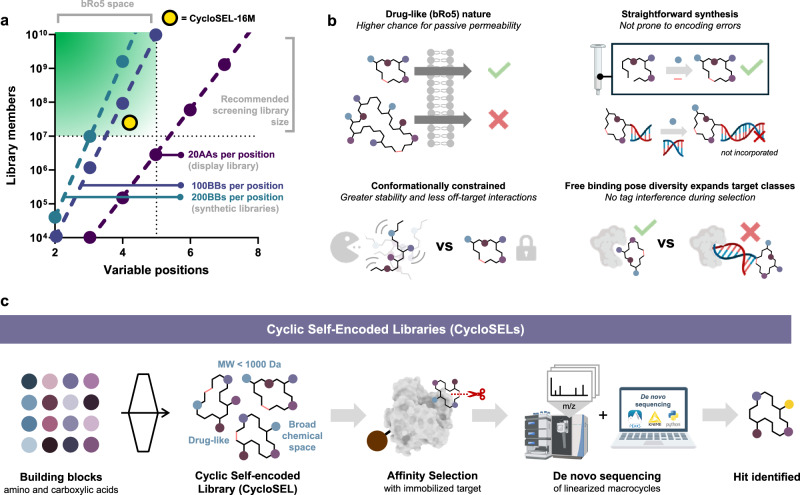
CycloSEL enables the screening of massive libraries of drug-like synthetic macrocycles. **a** Libraries relying on the molecular biology machinery (purple line) are limited to a restricted set of building blocks that require large ring sizes to reach a reasonable library size. Synthetic libraries (violet and blue lines) incorporate more diverse chemical space in compact macrocycle scaffolds and can reach high diversity while residing within the drug-like bRo5 space. The green box illustrates a simplification of the bRo5 space: peptidic macrocycles with over five building blocks plus cyclization linker are likely to exceed six hydrogen-bond donors (HBD) and molecular weight (MW) of 1000 Da. **b** Smaller macrocycles are more likely to achieve cell permeability. Compared to linear compounds, macrocycles (thanks to their locked structure) often display greater binding affinity, selectivity and stability. Solid-phase synthesis obviates intrinsic complications and limitations of DNA-encoded libraries (DELs) like DNA stability, solvent compatibility, and misencoding. Barcode-free libraries enable higher binding pose diversity sampling during selection and do not suffer from barcode interference, allowing for exploration of more vectors. **c** Schematic synthesis and screening workflow for CycloSELs.

Discovering novel high-affinity macrocycles requires the screening of large libraries, ideally with multimillion-member diversity. Phage and mRNA display enable the interrogation of very large cyclic peptide libraries (with up to 10^14^ members) by genetically encoding individual sequences^17,18^. However, these platforms typically require large sequences of 8–14 residues to achieve sufficient chemical diversity. Such large compounds reside outside the optimal bRo5 window (Fig. 1a, b), even if occasionally they can have intracellular activity^7^.

DNA-encoded libraries (DELs) have expanded the scope for macrocycle diversity in smaller scaffolds^4,6,19^ but they demand complex synthesis procedures, and can suffer from misencoded members when reactions are incomplete^20,21^. Moreover, all encoded technologies share the limitation of requiring macrocycles to be linked to bulky barcodes, which may interfere with target binding and the affinity selection process (Fig. 1a, b).

Individually synthesized synthetic macrocycle libraries can be screened without barcodes, but these libraries remain limited in size. Recent advances in fully synthetic macrocycle libraries using techniques like acoustic dispensing parallel synthesis have shown promise, but even these libraries remain limited to only a few thousand members, ultimately reducing the odds of identifying potent hits for challenging targets^3,22^.

Affinity selections from synthetic libraries combined with tandem mass spectrometry (AS-MS/MS) and de novo sequencing have recently gained traction and provide an intriguing alternative to molecular biology-based screening techniques^23–25^. Already, reports in the early 90s established the foundations for such chemical screenings with mass spectrometry-based decoding^26,27^. While these early studies relied on bead-bound libraries, recent developments made possible by substantial advances in mass spectrometry instrumentation and automated sequencing software enabled screening very large in solution libraries^23^. In AS-MS/MS, synthetic peptide libraries with up to 100 million members can be prepared by split-and-pool synthesis and panned against immobilized targets of interest. Enriched peptides are then identified by nanoLC-MS/MS analysis and de novo sequencing^28–30^. This workflow has previously been applied mainly for linear peptides, while interest in applying it to cyclic peptide libraries has been increasing with intriguing recent examples^31–35^. All of these studies focused on a typical peptide space, with no exploration of small macrocycles within the bRo5 space, aiming at delivering drug-like compounds designed for further therapeutic developments.

In this work, we introduce cyclic self-encoded libraries (CycloSEL), an AS-MS/MS platform for the discovery of small, drug-like macrocycles from fully synthetic, barcode-free, multimillion-member libraries, optimized to meet bRo5 criteria and address intracellular targets (Fig. 1c). By incorporating massive chemical diversity into compact scaffolds, we achieve library sizes up to 16 million members while retaining drug-like properties. This makes synthetic macrocyclic libraries a practical route to balance scale, diversity, and drug-likeness without sacrificing chemical diversity or drifting outside the bRo5 space (Fig. 1a). We demonstrate the technical robustness of the approach across hundreds of macrocycle variants that incorporate over 180 distinct building blocks and then prepared a 16 million-member library, of which most members reside within the drug-like bRo5 space. Using the cancer-related protein target CAIX, we demonstrate the affinity selection – MS/MS decoding workflow, achieving clear enrichment of true binders. We then showcase the application of the platform to rapidly discover a potent, cell-active inhibitor for the intracellular oncology target WDR5. A single selection round with CycloSEL identified a validated high-affinity hit (Biolayer interferometry *K*_D_ = 8 nM, single-molecule measurement *K*_D_ = 180 pM). Rapid hit elaboration yields a macrocycle capable of disrupting the leukemia-driving PPI between WDR5 and MLL with nanomolar potency, substantial passive membrane permeability due to chameleonic behavior, and activity against leukemia cell lines. Altogether, our platform is a valuable tool for screening massive libraries and generating early hits with high potency and with preoptimized drug-like properties, which promises to streamline future hit-to-lead developments.

## Results

### Synthesis and decoding of macrocycle libraries with compact scaffolds

**Fig. 2:**
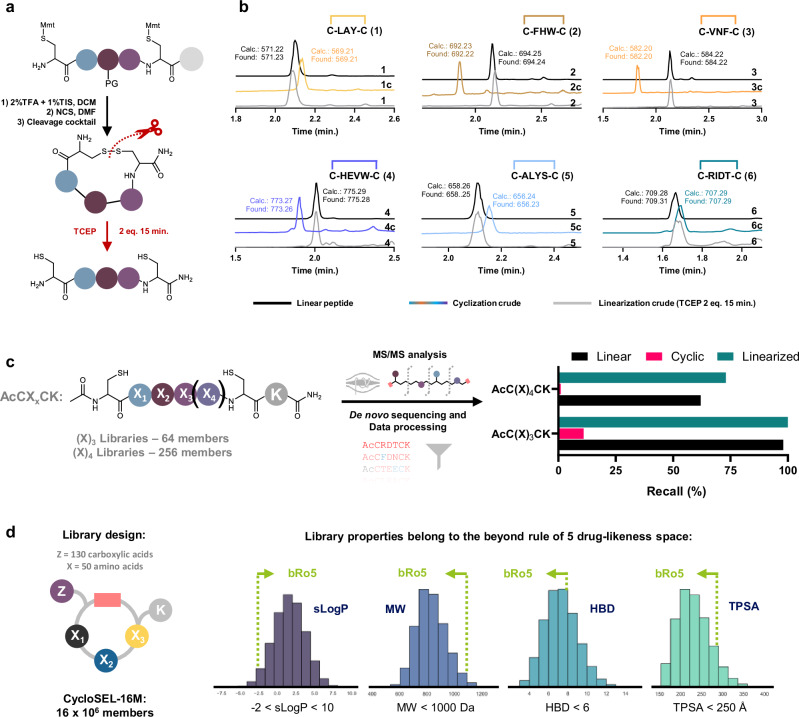
Robust chemistry enables the synthesis and decoding of combinatorial macrocycle libraries and leads to the preparation of a drug-like 16 million-membered library. **a** Solid-phase synthesis of CXXX(X)C-resin with Mmt-protected cysteines. Orthogonal Mmt removal and NCS oxidation results in macrocycle formation, subsequently globally deprotected and released from the resin. Mild TCEP treatment enables the linearization of the cycle. **b** The LC–MS traces of six examples demonstrate the quantitative cyclization and linearization. XIC search for linear precursor after cyclization or cyclic precursor after linearization were not found. Product peak shown by gray lines restored the expected linear mass. **c** Combinatorial synthesis of either 64 CX_3_CK or 256 CX_4_CK membered libraries. The libraries were analyzed by nanoLC-MS/MS by injection at 100 fmol/member, followed by de novo PEAKS sequencing. The bar graph shows how many of the library sequences were detected, in their linear form, in their cyclic form and in their re-linearized form. **d** CycloSEL-16M was prepared with building blocks optimized to adhere to bRo5 rules for macrocycles (property calculations were performed without the C-terminal lysine). Source data are provided as a Source Data file.

Pure synthetic macrocycles can be obtained by solid-phase synthesis followed by on-resin disulfide formation. Combinatorial libraries require robust synthesis procedures to ensure that all library compounds are efficiently produced in the parallelized split-and-pool setting, which does not allow for individual purifications. Aiming for compact scaffolds, we established the macrocycle synthesis on sequences with either three or four variable building blocks flanked by cysteines at the C- and N-terminus (CX_3_C and CX_4_C, X = amino acid). Disulfides enable rapid and robust cyclization of peptides. Specifically, we incorporated monomethoxytrityl (Mmt)-protected cysteines to enable orthogonal deprotection and cyclization directly on the resin, prior to global deprotection and cleavage. We synthesized three random sequences for each scaffold (compounds **1** to **6**) and first analyzed the synthesis outcome of the six linear precursors by LC–MS (Fig. 2a and Supplementary Fig. S1). For the on-resin cyclization, we used a strategy originally developed by Postma et al.: following orthogonal Mmt removal, the disulfide oxidation was performed by adding *N*-chlorosuccinimide to the resin, and subsequent global deprotection and cleavage release the cyclized compounds^36^. LC–MS analysis showed the quantitative transformation to the cyclic variants in all six examples. Crucially, disulfides also enable robust linearization chemistry, a requirement for tandem MS-based decoding of hit compounds, which is a fundamental step in our workflow. Therefore, subsequent treatment of peptides **1** to **6** with TCEP efficiently reduced the disulfide bond, yielding the linearized peptide (Fig. 2a and Supplementary Fig. S2). LC–MS analysis confirmed that the linearized products were obtained quantitatively and matched the initial linear variants, demonstrating the mild and efficient nature of the process.

Disulfide cyclization/linearization methodology enables high-fidelity synthesis and decoding of combinatorial synthetic macrocyclic libraries. We expanded our strategy from individual examples to defined combinatorial libraries in order to assess the robustness of both synthesis and decoding in the context of complex mixtures using de novo peptide sequencing. We prepared six different combinatorial libraries based on the previously defined scaffolds CX_3_CK, CX_4_CK, Ac-CX_3_CK, Ac-CX_4_CK, Mpa-X_3_CK, or Mpa-X_4_CK (Mpa - mercaptopropionic acid) via split-and-pool synthesis (Fig. 2b and Supplementary Fig. S3). In this way, we varied both ring size and N-terminal functionalization, and we included natural as well as unnatural building blocks in a one-to-one ratio at each variable position (X). With four different building blocks at each position X, the libraries contain either 64 or 256 members. We also included a C-terminal lysine in all libraries to ensure consistent ionization and fragmentation of y-ions for accurate sequencing annotation. We first cleaved all libraries in their linear form from the solid support, submitted them to nanoLC-MS/MS and analyzed the data using the peptide sequencing software PEAKS11^37,38^. PEAKS11 performs de novo peptide sequencing by interpreting tandem MS/MS spectra to directly infer amino acid sequences without relying on a database, using probabilistic scoring and fragment ion pattern recognition. To evaluate the efficiency of the synthesis and library sequencing, we computationally enumerated the full list of library peptides and compared to the actual sequencing output. The correct sequence recall was 90–95% for the scaffolds with three variable positions and 60–80% for the scaffolds with four variable positions. We then cyclized each library on solid support and analyzed the cleaved products using the same workflow. As de novo sequencing is only feasible for linear, ladder-generating structures, recall dropped to 0–10% for all examples, indirectly confirming successful cyclization of 90–100% of all library members (Supplementary Fig. S3 and Section 7). Next, we linearized the samples with TCEP and reanalyzed, bringing back the library recall to the initial high recall values (73–100%, Fig. 2b). By assessing in detail the individual libraries, we found that natural and unnatural building blocks were incorporated and decoded with the same efficiency. We also confirmed an overlap of 74–98% sequence identity recalled for linear and linearized samples (Supplementary Fig. S4 and Section 7).

Our recall rates of >70% for complex synthetic peptide mixtures obtained by de novo sequencing are in good agreement with previously reported achievable recalls^38^. Nevertheless, we investigated whether the “missing” peptides were absent due to synthesis issues or limitations inherent to mass spectrometry-based identification. To do so, we selected ten random peptides that were not identified by de novo sequencing and searched for their precursor masses in the raw LC–MS/MS data. In 7/10 cases, the peptide peak was detected, indicating that synthesis was not limiting; however, no MS/MS spectrum or only poor-quality spectra had been acquired. Given that real AS-MS/MS selection outputs typically contain only <100 hits^24,28,29,39^, thus exhibiting even lower complexity than our test samples, and considering that we were still able to recover the majority of peptides, we did not further optimize this step of the workflow. Overall, these results confirm that we can efficiently synthesize and decode macrocyclic peptide libraries with compact scaffolds and containing both natural and unnatural building blocks with high accuracy.

### Synthesis of a 16 million-membered library with drug-like properties

For the synthesis of a multimillion-membered library of synthetic macrocycles, we selected scaffold ZCX_3_CK with three variable building blocks (X) in the cycle and an additional exocyclic decorator (Z). We selected a set of 50 amino acids and 130 carboxylic acid decorators optimized for incorporation by solid-phase peptide synthesis (SPPS) and drug-like properties, previously curated in our group^40^. Virtual enumeration of the resulting 16 million-member library revealed that most members fall within the drug-like bRo5 space based on sLogP, molecular weight, hydrogen-bond donors, and TPSA distributions (Fig. 2b). These calculations were performed excluding the C-terminal lysine, as it functions as an aiding tag for mass spectrometry and not as part of the actual library design. In hits selected for further optimization, the activity of variants without lysine will need to be tested. Using split-and-pool synthesis, we prepared the actual library with all 180 building blocks (CycloSEL-16M). Before global deprotection and cleavage, we performed a quality control step by sampling two sets of 500 resin beads for test cleavage. Since each bead should display a single compound, we expected up to 1000 unique sequences. nLC-MS/MS analysis of 100 fmol per member, followed by PEAKS11 decoding, identified ~900 sequences matching the library design. Based on binomial proportions, this sample size provides a statistically robust estimate of the overall library quality with a 95% confidence interval (Supplementary Fig. S5 and Section 3.1.3). Observing 900 correctly synthesized beads (90%) allows extrapolation to the full library, to an overall synthesis quality of ~88–92%, providing an assessment of global synthesis fidelity. In this quality control step we also confirmed successful incorporation of 94% of the amino acid building blocks and 89% of the carboxylic acid modifiers (Supplementary Fig. S5). Important to note that the quality control step does not measure the total yield of each library peptide; it only confirms that a sufficient amount of each detected peptide was synthesized to meet the MS detection and MS/MS sequencing thresholds. Notably, the ability to perform a statistically representative quality control step to verify the presence and integrity of actual library members is a unique advantage of self-encoded libraries, unlike genetically encoded libraries, where incomplete reactions can cause mismatches between code and compound. After this validation, we cleaved the library, bulk purified it by reversed phase chromatography and obtained 326 mg (52% yield from a 500 µmol scale synthesis on 2 g of beads). Such a quantity, using previously described screening concentrations, would enable ~1700 individual selection runs. Considering triplicates for target and control proteins that would enable over 250 experiments. With this validated library, we proceeded confidently to affinity selections.

### CycloSEL validation with a discovery campaign against CAIX

High-affinity binders can be efficiently enriched from the CycloSEL-16M library. We first evaluated this platform using an affinity selection against CAIX, an oncology target overexpressed in hypoxic tumors^41^, promising for radionuclide cancer therapy^42^. Moreover, CAIX is particularly suitable for benchmarking library platforms due to its well-characterized preference for building blocks bearing aromatic sulfonamides, which facilitates the detection and quantification of true hits following selection^20,42–44^. In previous DEL studies involving macrocycles, aromatic sulfonamides have been attached to aliphatic linker structures, which likely avoid macrocycle clashes with the binding pocket^5,6,20^. In contrast, our library contains an aryl sulfonamide directly coupled to the exocyclic macrocycle amine, without any additional linker. It is therefore likely that only a subset of these structures can adopt conformations that allow productive binding to CAIX. As pulling down and detecting all possible 125,000 theoretical sulfonamide variants seemed unrealistic, we considered this design a suitable benchmark for evaluating our library.

Biotinylated CAIX was immobilized on magnetic streptavidin beads and incubated with the library (10 fmol/member, a quantity resulting in appropriate detection in the sensitivity range of the mass spectrometer). The library at this concentration (overall 160 µM) did not show signs of precipitation. For larger library sizes or different scaffolds, solubility limits, and with that maximal achievable library, size would have to be individually reevaluated. Non-binders were washed away, and retained binders were eluted using 6 M guanidine supplemented with 10 mM TCEP. After desalting, the eluate was analyzed by nLC-MS/MS and sequenced using PEAKS11. The exact affinity selection procedures are reported in Supplementary Section 8A. Parallel control selection against streptavidin was performed to identify and eliminate background binders.

**Fig. 3:**
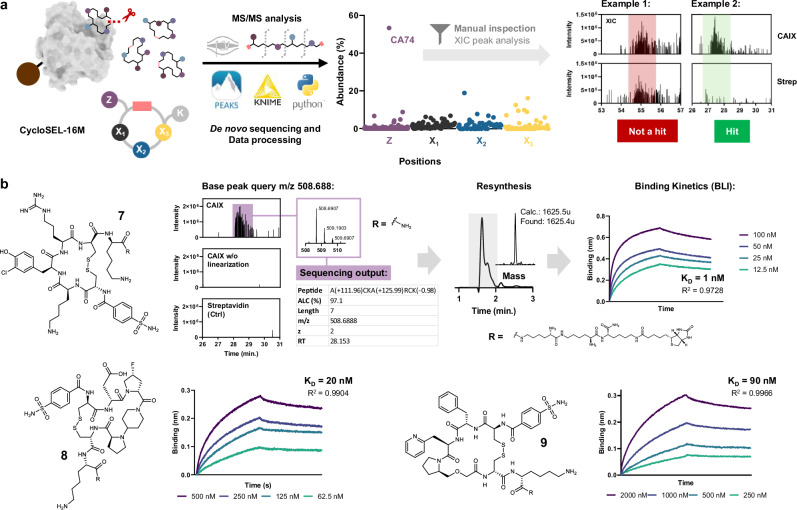
CycloSEL enriches true binders for CAIX. **a** CycloSEL-16M was screened against CAIX. nanoLC-MS/MS followed by computational data analysis indicated strong enrichment of aromatic sulfonamide CA74, indicating the successful enrichment of true binders during the selection. The dot plot indicates the relative enrichment of BBs in each variable position. The X-axis refers to building block identity. Hits are confirmed by manual XIC peak analysis. **b** Three hits containing the enriched CA74 were synthesized and tested by biolayer interferometry (BLI) for binding to CAIX. The curves show BLI association and dissociation, kinetic *K*_D_ and kinetic fitting *R*^2^. Source data are provided as a Source Data file.

For PEAKS we set an average confidence level threshold of 70%, then filtered for sequences matching the library design (ZCXXXCK) and analyzed the remaining list of 330 candidate sequences, resulting from four technical replicates. Plotting the BB frequencies for each position clearly indicates a strong enrichment of aromatic sulfonamide CA74. This BB was present in 176 compounds out of 330 filtered sequences, corresponding to a strongly statistically significant *p* value of 1.1 × 10^−275^ when compared to a random distribution (Fig. 3a, Supplementary Fig. S6, and Section 8.4 Table S4). As resynthesis and testing of false positives represent a substantial challenge and waste of time and resources in any screening campaign, we assessed whether based on further data filtering we could narrow down the candidate list to true hits. We picked a number of candidates with and without CA74 and analyzed the extracted ion chromatogram in the raw data and compared it to the same extracted ion chromatogram in the control selection. Only candidates showing a reasonable peak shape and absence of the same peak in the control sample were further considered hits. This manual data inspection step indicated that all the candidates containing aryl sulfonamide CA74 had distinct peaks in the CAIX sample and were absent in the control, indicating specific target binding. The other candidate sequences, without CA74, did not pass the filtering criteria and could be discarded (Supplementary Section 8.3). This manual filtering step, therefore, can further reduce the candidate list to larger fractions of true positives. Our selection likely did not pick up all possible hits present in the library. However, being able to detect >100 true hits candidates while minimizing false positives, appeared as a promising outcome.

While the presence of building block CA74 is already a strong indication for true hits, we further validated our selection outcome by resynthesizing three hits with diverse macrocycle compositions. We tested these biotinylated hits **7**, **8**, and **9** via biolayer interferometry (BLI) and obtained kinetic *K*_D_s of 1, 20, and 90 nM, respectively (Fig. 3b). While the kinetic BLI traces did result in good fits (with *R*^2^s between 0.97 and 0.99), the curves did not reach saturation at higher concentrations, indicating unideal binding behavior in these settings. However, as the experiment was meant as a confirmation of actual binding of predictable hits and not for exact *K*_D_ determination of novel chemical matter, and as the *K*_D_ closely fit the typical values found in literature for aryl sulfonamides binding to CAIX^20,42–44^, we did not further optimize the binding assay. Together, these results validate our CycloSEL platform as a viable approach for screening multimillion-membered libraries and efficiently enriching high-affinity ligands.

### Discovery of a low nanomolar hit against the oncology target WDR5

Next, we selected an intracellular drug target, WDR5, to test whether CycloSEL would enable us to identify potent inhibitors with translational potential for more advanced functional and cellular assays. WDR5 is a core component of the MLL histone methyltransferase complex and plays a central role in maintaining leukemogenic gene expression by scaffolding PPIs with rearranged MLL proteins^45,46^. Beyond MLL fusion proteins, WDR5 also facilitates chromatin recruitment of oncogenic transcription factors such as MYC^47^ and binds specific non-coding RNAs that modulate epigenetic regulation^48^, making it a multifaceted and compelling target for therapeutic intervention in AML and other malignancies. Following the successful application of CycloSEL to CAIX, where hit binding is driven predominantly by a single building block, we next sought to apply the platform to WDR5 as a more complex, yet druggable, target^49–51^.

**Fig. 4:**
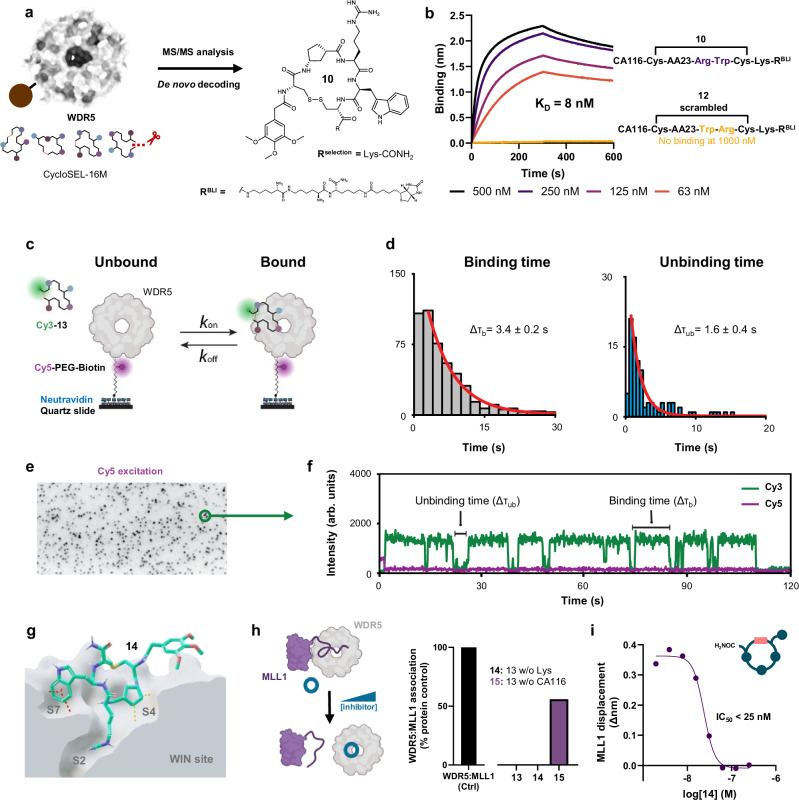
CycloSEL leads to the identification of a potent WDR5-MLL inhibitor. **a** CycloSEL screening against immobilized WDR5 identified a single confirmed hit, which was resynthesized (**10**). **b** BLI validation of hit **10** binding to WDR5 with *K*_D_ = 8 nM. The control **12** with two swapped BBs did not show any binding to the protein. **c** Schematic representation of the single-molecule fluorescence assay used to measure the binding kinetics of **13** to immobilized WDR5. Data obtained from triplicates. **d** Dwell-time distribution of the binding and dissociation events. **e** A CCD snapshot of immobilized Cy5-labeled WDR5 molecules under direct Cy5 excitation. Each bright dot corresponds to an individual WDR5 molecule. Subsequent binding and dissociation events of compound 14 are monitored using Cy3 excitation. **f** Intensity vs time plot. Cy3 binding events (green) colocalized with the initial Cy5 WDR5 signal obtained from red-laser excitation for 2 s (purple). **g** Docking model of the macrocycle **14** bound to the WDR5 WIN site. **h** Schematic representation of the inhibition of the WDR5-MLL PPI by macrocycles. The selection hit **13** disrupts the WDR5-MLL (MLL immobilized on BLI tips, WDR5 in solution at 50 nM treated with 100 nM inhibitor). The selection hit without the C-term Lys (**14**) maintains full activity. Removal of CA116 (replaced by acetyl) results in reduced (yet not abolished) activity (**15**). **i** Full inhibition curve of **14**. Source data are provided as a Source Data file.

We immobilized recombinant biotinylated WDR5 (Supplementary Fig. S7) on magnetic beads and screened our CycloSEL-16M library. After data analysis and filtering, we obtained only two sequences meeting the hit criteria for resynthesis (PEAKS confidence >70%, PEAKS output matching the library design, extracted mass for hit compound clearly detected by visual raw data inspection, extracted mass for hit compound not detected in control sample) (Supplementary Section 9 Table S5). While the detection of only two sequences out of a 16 million-membered library might seem surprising, these results match well with previous AS-MS/MS studies in which only 1–3 hits were identified, but then validated as excellent true hits^24,28,29^. We resynthesized the two candidate hits with a C-terminal biotin tag and validated binding to WDR5 by BLI. Compound **10** showed a binding affinity of 8 nM, while **11** did not bind to WDR5 in this assay (Fig. 4a, b and Supplementary Fig. S8). To test sequence-specific binding of macrocycle **10**, we also synthesized scrambled isomer **12** in which the arginine was flipped with tryptophan. Isomer **12** showed no association with WDR5, indicating that the exact positional composition of building blocks in the macrocycle is crucial for binding. The arginine in the hit macrocycle is a feature present also in the canonical MLL peptide, and other known inhibitors also present a guanidino group or a mimic thereof as a partial pharmacophore^49–52^.

To further validate binding of the selection hit, we performed a single-molecule fluorescence binding assay as an orthogonal experiment (Fig. 4c). In this experiment the protein is surface immobilized and the peptide in solution, better emulating the binding events in the affinity selection. This assay kinetically measures binding events at a single-molecule level via co-localization monitored by total internal reflection fluorescence microscopy^53^. We immobilized Cy5-labeled biotin-WDR5 on neutravidin coated quartz slides (Fig. 4c, d) and incubated with Cy3-labeled hit macrocycle **13**. Binding was characterized by two-state on and off transitions (Fig. 4e). From the dwell-time distributions (Fig. 4e), we extracted a binding rate (*k*_on_) of 2.1 ± 0.7 × 10⁹ M⁻¹ s⁻¹ and a dissociation rate (*k*_off_) of 0.4 ± 0.1 s⁻¹, resulting in a calculated *K*_D_ of 180 ± 8.8 pM. Taken together, while the affinity selection produced a single hit, this compound showed exquisite potency in WDR5 binding.

Macrocycle **14** likely binds to the WDR5 WIN site. Guided by the native protein–protein interactions of WDR5, we used pharmacophore-based modeling to dock macrocycle **14** (a variant of **10** without the BLI biotin tag and without C-terminal lysine) into the WIN site, the location of the AML-driving PPI with rearranged MLL. The docking results placed the macrocycle in a conformation consistent with the known canonical MLL peptide binding mode, positioning its central arginine deep in the pocket and capturing the expected pattern of hydrophobic and hydrogen-bond interactions (Fig. 4g). Overall, the model supports a binding mode in which the macrocycle adopts a well-defined pose within the WIN site. Full computational details can be found in Supplementary Section 4.1.3^53,54^.

Macrocycle 14 disrupts the AML-driving PPI between WDR5 and MLL. To experimentally confirm that macrocycle **14** binds to the WIN site as predicted by our model, and to evaluate its ability to block the WDR5–MLL interaction, we developed a BLI-based competition assay. We synthesized a biotinylated MLL peptide (Ac-ARAEVHLRKSAFD-*N*,Lys-Lys(Biotin) (**16**)) and immobilized it on BLI tips. Initial experiments confirmed binding between WDR5 and **16** with a dissociation constant of 103 nM (Supplementary Fig. S8). We then preincubated WDR5 (50 nM) with macrocycle **14** (100 nM) prior to the BLI association step. Under these conditions, no association was detected, indicating that macrocycle **14** fully occupied the WIN binding site and prevented interaction with the immobilized MLL peptide. We also prepared a variant still bearing the C-terminal lysine (**13**), which had been included as a fixed residue only to aid sequencing. Both variants **13** and **14** showed comparable inhibitory potency. (Fig. 4h). Given the uncertain role of the exocyclic carboxylic acid building block (based on docking), we also tested a variant in which this group was removed (**15**). This compound maintained partial inhibitory activity but was less potent than the original macrocycle (Fig. 4h). Finally, a full concentration–response analysis established an IC₅₀ value of <25 nM for macrocycle **14**, which corresponds to the lower detection limit of the assay (Fig. 4i).

### Optimization of the WDR5 hit to a stable and highly chameleonic macrocycle with passive permeability and cell activity

**Fig. 5:**
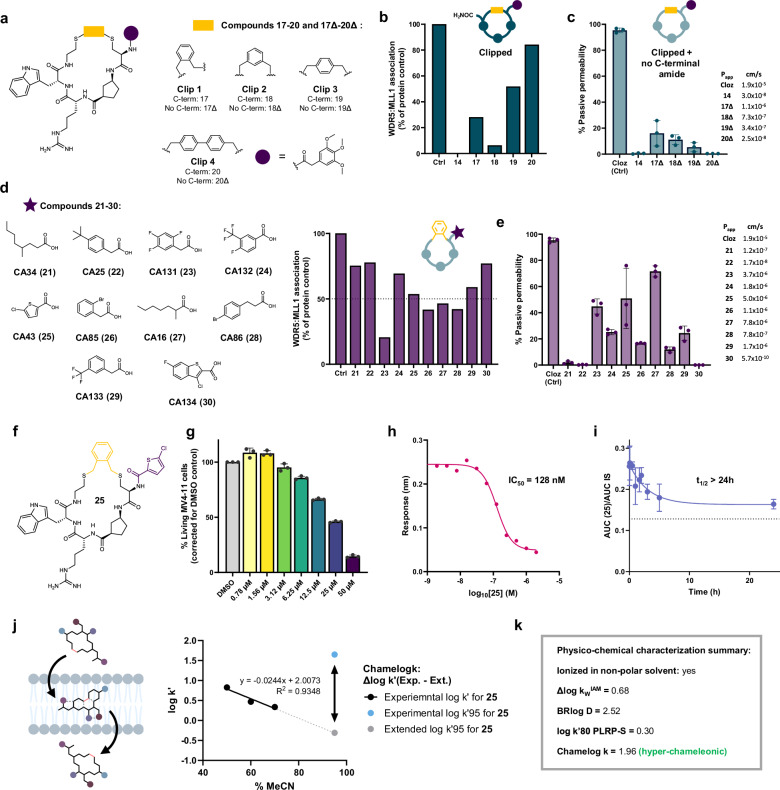
Rapid development of a chameleonic lead with passive membrane permeability, excellent serum stability and activity against AML cells. **a** Disulfide replacement with clip linkers. **b** Inhibition activity of variants **14** and **17**–**20**. **c** Membrane permeability of variants **14** and **17Δ-20Δ**, determined by PAMPA. Data presented as mean values ± SD for technical triplicates in the same plate. **d**, **e** Screening of different carboxylic acid decorators for inhibition activity by competitive BLI (100 nM for compound and 50 nM for WDR5) and PAMPA (100 µM for 18 h at RT). Data presented as mean values ± SD for technical triplicates in the same plate. **f** Lead compound **25**. **g** Cell proliferation inhibition caused by **25**. MV-4:11 cells were treated with the inhibitor for 72 h and measured via CellTiter-Glo®. Mean values and SDs are shown for technical triplicates measured on the same plate. **h** BLI inhibition curve of **25** against the WDR5-MLL PPI. **i** Serum stability of **25** measured in 25% human serum in PBS at 37 °C, intact compound determined via LC–MS. Data presented as mean values ± SD. **j** 25 displays unusually high chameleonicity measured via chromatographic methods. **k** Full physicochemical characterization panel for **25** using chromatographic methods. Source data are provided as a Source Data file.

Macrocycle **14** tolerates disulfide linker replacements. Because the disulfide linker required for the linearization step represents a likely point of instability for the macrocycle, we next investigated possible stable linker replacements. In our structural model, the disulfide linkage does not appear to engage in specific protein interactions and is instead rather solvent-exposed. We therefore explored replacements using clip chemistry with biscysteine reactive linkers^54^. Several linker variants (Fig. 5a and Supplementary Fig. S9) were tested by clipping the two cysteines. Macrocycles **17** and **18**, cyclized with clips **1** and **2** retained strong inhibition potency, while linkers **19** and **20** (clips **3** and **4**) caused substantial reduction of activity (Fig. 5b).

Next, we assessed the membrane permeability of the macrocycle variants using a PAMPA assay. We performed a first round of permeability testing with macrocycles **17**–**20**, as well as a linear analog where the cysteine positions were mutated to alanine (**s1**). None of the variants showed relevant permeability or good permeability/potency combinations (Supplementary Fig. S9). To further compact the ring, we prepared variants of the macrocycles **17**–**20** without a C-terminal amide (compounds **17Δ–20Δ**, Supplementary Fig. S10). At 100 µM, compounds **17Δ** and **18Δ** showed 16 and 11% permeability, respectively, after 18 h of incubation, while compound **14** showed <1% permeability. Macrocycles **19Δ** and **20Δ** displayed <5% permeability (Fig. 5c). We also explored alternative linker replacement strategies, including head-to-tail macrolactamization, and we performed site-selective backbone *N*-methylations (**s5** to **s23**). While these approaches preserved MLL1 displacement potency, they resulted in reduced permeability (Supplementary Fig. S11). Based on these results, we continued optimizing macrocycle **17Δ**, which combined strong MLL1 displacement potency (IC₅₀ = 32 nM; Supplementary Fig. S12) with promising PAMPA performance.

Optimization of the exocyclic decorator boosts membrane permeability. Seeking to further improve permeability, we set out to investigate the influence of the carboxylic acid decorator, as that functional group had been shown to be the least crucial for binding. We synthesized and tested a panel of 10 variants of macrocycle **17Δ** by incorporating different carboxylic acid decorators, including aliphatic, heteroaromatic and aromatic variants with diverse functional group patterns (compounds **21**–**30**, Fig. 5d). Five variants (**23**, **25**, **36**, **27**, and **28**) retained good potency in displacing MLL1 from WDR5 (equal or above 50% inhibition) when tested at 100 and 50 nM WDR5 (Fig. 5d). In parallel, we tested the ten variants in the PAMPA assay to assess their passive permeability. Strikingly, we observed a dramatic improvement in passive permeability for several decorator variants, especially for **23**, **25**, and **27**, reaching 45, 51, and 72%, respectively (Fig. 5e). The apparent permeability values (−log*P*_app_) for compounds **23**, **25**, and **27** was calculated to be, respectively, 5.4, 5.4, and 4.9 (Supplementary Section 3.1.3). Compounds with −log*P*_app_ < 6 are usually referred to as potentially permeable^55^.

Macrocycle **25** inhibits AML cancer cell proliferation. Encouraged by the permeability results, we tested the activity of the most promising variants (**23**, **25**, and **27**) in the AML cell line MV-4:11. These cells rely on the WDR5-MLL interaction and the disruption of this PPI is expected to result in inhibition of cell proliferation. We also took along the initial variant **17Δ**. While macrocycle **17Δ** did not inhibit cancer cell proliferation as expected given its low membrane permeability, variants **23**, **25**, and **27** did inhibit cell growth (Fig. 5f, g and Supplementary Fig. S13). Compound **25** had the most potent activity with a clear dose-dependent profile. As a control, we also treated HL-60 cells with variants **23**, **25**, and **27**. HL-60 cells are less WDR5 dependent and, indeed, the compounds had a markedly weaker effect on their proliferation. (Supplementary Fig. S13). For macrocycle **25**, we reconfirmed the in vitro potency in the BLI competition assay and obtained an IC_50_ of 128 nM (Fig. 5h). We also measured serum stability, and macrocycle **25** was still intact to >50% after 24 h.

Drawing from our library design strategy, which emphasizes bRo5-compatible properties, macrocycle **25** satisfied several of these parameters. However, its ten hydrogen-bond donors (HBDs) constitute a clear violation of bRo5 guidelines for drug-like compounds. To understand its unexpectedly high passive permeability, we carried out a detailed physicochemical characterization using chromatographic methods (Fig. 5j and Supplementary Section 3.1.3)^56,57^. These experiments showed that macrocycle **25** displays optimal lipophilicity, acceptable polarity, and marked chameleonicity - the ability to alter its conformation and its properties, when moving from a polar to a nonpolar environment^56,58^. Chameleonicity is known to enhance membrane permeability, as chameleonic molecules can adopt a more compact, less polar conformation in the interior of the membrane bilayer. The measurements to test chameleonic behavior entail isocratic chromatography with sequential runs at increasing percentage of organic solvent. Whereas a linear decrease in retention time is typically expected in an RP-HPLC system, chameleonic compounds deviate from this trend at very high organic-solvent concentrations^57^. Macrocycle **25** exhibited a Chamelogk >1.96, demonstrating a substantial deviation from the linear behavior and thus pronounced chameleonicity. For comparison, cyclosporin, often considered the benchmark macrocycle for chameleonic behavior, has a Chamelogk value of 1.25, which is typically classified as high chameleonicity. The value observed for compound **25**, therefore, represents an unusually elevated degree of chameleonicity. Thus, the remarkable permeability of macrocycle **25** can be mainly rationalized by its extraordinary chameleonicity, which appears sufficient to override its substantial hydrogen-bond donor load.

Taken together, the identification of lead macrocycle **25** showcases how the CycloSEL platform can be used for rapidly discovering novel inhibitors for intracellular targets.

## Discussion

Traditional macrocycle discovery platforms are powerful tools^6,22,59,60^, yet several challenges, like achieving drug-likeness, maintaining synthetic tractability, and expanding chemical space, represent meaningful limitations. Here, we showcase CycloSEL, a platform that directly integrates drug-like properties into library design, avoids barcode-related complications, and enables the construction of macrocycle libraries with massive size and diversity. In this study, we described the technological development and benchmarking of the platform and then demonstrated how CycloSEL enables the rapid identification of potent binders for the AML driver WDR5 that, via medicinal chemistry optimization, could be transformed to macrocycles with passive membrane permeability and activity in leukemia cells.

For large-scale screening and hit decoding, we used an affinity selection–tandem mass spectrometry (AS-MS/MS) workflow^23^. Because decoding relies on de novo peptide sequencing, clear fragmentation ladders are essential, which in turn requires linearization of cyclic compounds prior to MS/MS. Reported linearization methods include Edman degradation^32,34^, acidic imidazolidinone opening^31^, Pd-catalyzed ring opening^34^, thioether oxidation^35^, and disulfide reduction^33^. We chose disulfides for their robust and mild chemistry during both cyclization and linearization, and because they do not add bulk to our compact scaffolds. While convenient at the screening stage, we recognize the limitation that disulfides are prone to reductive cleavage inside cells, typically necessitating a linker replacement later. Depending on the binding mode, this can be straightforward or highly challenging. In our WDR5 case, modeling indicated the disulfide was solvent-exposed, and indeed it tolerated several stable replacements. Ideally, future software and workflow advances should be developed to allow screening and decoding of inherently stable macrocycles without requiring any linearization elements.

We worked with a library of 16 million compounds, and based on previous AS-MS/MS efforts, screening up to 100 million appears feasible^24,28,29^. DNA-encoded libraries (DELs) sometimes report even larger sizes and, in principle, access comparable chemical space. However, recent critical evaluations suggest that the highest discovery rates occur with libraries in the range of 10–100 million members^42,61^. Moreover, because no direct quality control can be performed on barcoded libraries, the integrity of very large DELs remains uncertain^4^. In contrast, CycloSEL and synthetic libraries in general rely on straightforward solid-phase synthesis, which does not require DNA handling, allows organic solvents, and supports optimized reaction conditions. Compounds are decoded directly from their own structure, eliminating the risk of misencoding observed in DELs^20^. This direct decoding also enables statistically significant sampling for quality control. Beyond these points, screening libraries without barcodes offers further benefits: it avoids potential barcode interference with target binding^62^ and removes the limitation in screening against nucleic acid–binding targets inherent to DEL formats. However, a minimalistic tag, the C-terminal lysine, still needed to be incorporated into the library design. This small constant scaffold modification resulted important for high-fidelity sequencing.

The CAIX selections yielded clear enrichment of a hit family containing the control building block with an aromatic sulfonamide, demonstrating that the workflow can successfully enable pull-down and MS-based identification of tens of potential hits. In contrast, the WDR5 campaign produced only two candidate hits, of which one was confirmed as a true binder. Given the later observation that the carboxylic acid tolerated replacement relatively well, it is somewhat surprising that more parent sequences were not identified directly during SEL screening. On the other hand, swapping just two building blocks within the ring completely abolished binding, highlighting the critical importance of that precise scaffold. The reason only a single hit emerged remains unclear—it may simply have been the only compound exceeding the affinity threshold needed to yield sufficient material for detection and decoding. Future work could explore less stringent or alternative selection conditions to broaden recovery. Even with advanced mass spectrometry instrumentation, platforms like CycloSEL do not rival the sensitivity achievable through PCR and deep sequencing. That said, it is worth emphasizing that while lower-affinity hits might have been missed, the true hit rate was extremely high in this case, eliminating the need to resynthesize large numbers of false positives, the ultimate enemy of selection platforms.

Our bRo5-optimized library enabled rapid development of a compound with passive permeability and cellular activity. The initial hit from screening showed poor permeability (<1% by PAMPA at 100 µM), but replacing the disulfide with a *o*-xylene clip and removing the C-terminal amide substantially improved this property, underscoring how starting from a bRo5-compliant library can facilitate macrocycle optimization. Interestingly, modifying the carboxylic acid decorator had an even greater effect on permeability, suggesting that individual building blocks can be major drivers of membrane penetration. Further systematic studies to identify such components could be highly valuable. Ultimately, our lead compound, which achieved 51% permeability and demonstrated activity against AML cells, complies with several of Kihlberg’s guidelines while still violating the maximum number of hydrogen-bond donors^14,15^. This highlights an important point: general adherence to bRo5 principles is beneficial, but every case is unique, and exceptions to the rules are clearly possible. In this case, a follow-up analysis on our lead macrocycle **25** revealed its strong chameleonic behavior, which likely allows it to cross the cell membrane.

Altogether, CycloSEL is a promising macrocycle discovery platform combining high chemical diversity, a drug-like bRo5 design space, and direct structure-based decoding at scale. By enabling the efficient discovery of ligands for intracellular protein–protein interactions, we envision CycloSEL as a powerful and accessible tool for tackling challenging targets and expanding the therapeutic potential of macrocycles.

## Methods

Detailed methods, synthetic procedures, compound characterization, building block selection, library enumeration, and software usage are described in the Supplementary Information.

### Manual solid-phase peptide synthesis (SPPS)

ProTide® Rink amide resin (loading 0.6 mmol/g, typical scale: 100 mg, 0.06 mmol) was loaded into a fritted syringe (5 mL), swollen in DMF (5 mL) for 5 min and then drained. Resin Fmoc deprotection was performed by addition of piperidine (20% in DMF, 5 mL), to the resin (1 × 1 min + 1 × 5 min), followed by draining and washing the resin with DMF (5 × 5 mL). Each Nα-Fmoc protected amino acid (0.18 mmol, 3 eq) was dissolved in DMF to a concentration of 0.25 M. After dissolving, the same volume of 0.24 M HATU in DMF solution was added to a final concentration of 0.125 M of amino acid and 0.120 M HATU. Immediately before the coupling, DIPEA (30 eq to resin loading) was added to the mixture to activate the amino acid. This solution, after 15 s, was added to the resin and reacted for 15 min, with occasional stirring. After completion of the coupling step, the syringe was drained, and the resin was washed with DMF (3 × 5 mL). Fmoc deprotection was performed by addition of piperidine (20% in DMF, 5 mL), to the resin (1 × 1 min + 1 × 5 min), followed by draining and washing the resin with DMF (5 × 5 mL).

### C-terminal amide-free bis-thiol manual peptide synthesis

TentaGel® S NH₂ 90 μM resin (loading 0.26 mmol/g, typical scale: 100 mg, 0.026 mmol) was loaded into a fritted syringe (5 mL), swollen in DMF (5 mL) for 5 min and then drained. Fmoc-Cysteamine-Suc (0.78 mmol, 3 eq) was first coupled to the resin and deprotected as described above. Elongation of the peptide sequence was carried out as described above.

### N-terminal acetylation

After deprotection and washing of the last amino acid building block, the resin was incubated with a mixture of DMF/AC2O/DIPEA (8:1:1 v/v) for 15 min. After the allotted time, the resin was then washed with 3x 5mL DMF and 2x 5mL DCM.

### Mmt deprotection

Resin containing the Mmt-protected biscysteine peptides was subjected to incubation with a solution of 2% TFA, 1% TIS in DCM for 15 min under agitation. The procedure was repeated until the resulting deprotection solution ran clear and not bright orange. In between incubations, the resin was washed three times with the same solution. Once deprotected, the resin was washed 3x with DMC and 3x DMF.

### Disulfide macrocyclization

The cyclization protocol was adapted from ref. ^36^. Resin containing the deprotected biscysteine peptides was subjected to incubation with 2 eq. of *N*-chlorosucinimide in DMF (100 uL per μmol of peptide) for 1 h. After incubation, the solution was discarded, and the resin was washed with 3x DMF, 3x DCM and dried before cleavage.

### Global acidic cleavage/deprotection

Upon completion of the peptide synthesis, the resin was treated with a cleavage cocktail containing 92.5% TFA, 5% water, 2.5% triisopropylsilane (TIS) (v/v) at room temperature for 90 min or with 87.5% TFA, 5% water, 5% 1,2-ethanedithiol (EDT) and 2% TIS (v/v) for 90 min (for free cys-containing linear peptides). The TFA volume was then reduced under a nitrogen stream, and cold diethyl ether (–20 °C) was added to precipitate the peptide. The resulting suspension was centrifuged at 10,000×*g* for 5 min and the liquid was discarded. After repeating this step once more, the pellet was dissolved in 70/30% water + 0.1%FA and acetonitrile + 0.1% FA.

### TCEP cleavage from SUC-functionalized resin

After C-terminal amide-free bis-thiol manual peptide synthesis, SUC-Cysteamine-functionalized resin was swollen in 1:1 MQ water/MeCN for 10 min. After draining, the resin was incubated with 100 mM TCEP HCl in 1:1 MQ water/MeCN (~50 µL /μmol peptide on resin) for 1 h. The solution was drained, and its volume was reduced under nitrogen and desalted using the flash C18 chromatography method C.

### Stapling macrocyclization

Dry peptide powder was dissolved in 30% MeCN/70% 100 mM NH_4_HCO_3_, pH 8 buffer to a final concentration of 1 mM. Then, the correct volume of staple stock in MeCN was added to the peptide solution to a final concentration of ~1 mM peptide and 1.2 mM staple (1.2 eq.). The reaction was stirred for 30 min or until full completion, followed by UPLC–HRMS.

### General procedure for split-and-pool combinatorial library synthesis

Coupling and Fmoc deprotection steps were performed according to the stoichiometries and procedures described above. Monosized Tentagel beads (30 μm, loading 0.26 mmol/g, variable scale) were loaded into a fritted syringe, swollen in DMF and then coupled to Fmoc-Rink Amide linker. For variable positions, the resin was suspended in DMF, divided into equal aliquots, coupled to the respective Fmoc amino acid and then pooled after Fmoc deprotection. Mmt deprotection, disulfide macrocyclization and cleavage were performed using the above mentioned procedures. Library desalting and purification was performed by flash C18 chromatography according to Method B. Small test libraries were stored in stock in 5% DMF/95% MQ + 0.1% FA water at −20 °C. High-diversity libraries were stored in DMSO stocks at −20 °C.

### Quality control for high-diversity libraries

About 1 mg of dry linear library resin was suspended in 1000 μL MQ + 0.1% FA and homogenized using sonication at 60 °C overnight. Once well suspended, 10 μL (~500 beads/peptide identities, ~4 pmol/peptide) was aliquoted to a plastic 1.7 mL Eppendorf tube and centrifuged at 15,000×*g* for 5 min. The supernatant was removed, and 100 μL of cleavage cocktail (87.5% TFA, 5% water, 5% 1,2-EDT, and 2% TIS) was added and incubated for 1 h 30 min. The cocktail was then evaporated under a stream of nitrogen and peptides suspended in 100 μL MQ + 0.1% FA. This sample was then subjected to StageTip purification (described below), lyophilization and reconstitution in 10 μL MQ + 0.1% FA. About 9 μL were subsequently injected for nLC-MS/MS analysis.

### Affinity selection

A KingFisher™ Duo Prime Purification System was used to perform our affinity selection experiments. The protocols were developed with BindIt 4.1 Software. Affinity selection experiments against CAIX and WDR5 were performed in duplicates with protein (150 pole) immobilized on Dynabeads MyOne Streptavidin T1 (1 mg) and library (10 fmol/member) with the King Fisher protocol as described in Supplementary Section 3.1.3 Tables 1 and 2, unless stated otherwise.

### Sample preparation after AS

Samples from the affinity selection procedure were subjected to StageTip desalting. The StageTips were prepared as described by Rappsilber et al. using C18 material from Empore SPE 47 mm disks (66883-U, Merck). The StageTips were pre-conditioned with 100 μL MeOH, 100 μL of 0.1% (v/v) FA in MeCN, and 100 μL of 0.1% (v/v) FA in MQ, respectively, by centrifuging for 3 min at 300×*g*. The samples were then loaded on the StageTips and washed with 100 μL of 0.1% (v/v) FA in MQ. Compounds were eluted by adding 100 μL of 0.1% (v/v) FA in MeCN:MQ (7:3) and subsequently lyophilized before resuspending in 10 μL 0.1% (v/v) FA in UPLC-MS grade water. Afterward, the 10 μL were transferred to a LC–MS vial, and 9 μL was injected into the LC–MS/MS system.

### Biolayer interferometry (BLI)

Biolayer interferometry (BLI) assays were performed in 96-well plates (GreinerBio-One, polypropylene, flat-bottom, chimney well) using an Octet R4 system (SATORIUS). Wells were filled with 200 μL of kinetic buffer, compound solution or protein solution, purified compounds. Proteins were dissolved in the appropriate kinetic buffer specific for each target (CAIX: 1× PBS, 0.02% Tween-20, 1 mg/mL BSA [0.1% w/v]; WDR5: 20 mM Tris, 500 mM NaCl, pH 8.0, 0.02% Tween-20, and 1 mg/mL BSA [0.1% w/v]).

#### Direct binding assay

Purified biotinylated compounds were dissolved in an appropriate kinetic buffer and immobilized onto the streptavidin biosensor for 60 s. Sensors were then dipped into the kinetic buffer for 60 s, a concentration gradient of protein solution for 300 s and into the kinetic buffer for 300 s. Measurements were carried out at 20 °C.

#### Competition assay

Purified biotinylated competitors were dissolved in the appropriate kinetic buffer and immobilized onto the streptavidin biosensors at 100 nM for 60 s. Sensors were then dipped into the kinetic buffer for 60 s, and the concentration gradient of macrocyclic hit in a constant concentration protein solution for 600 s. Measurements were carried out at 20 °C.

### Single-molecule fluorescence sample preparation adapted from ref. ^53^

Macrocycle 10 was labeled at the lysine sidechain using 2 eq. of Sulfo-Cy3-NHS (Lumiprobe, 21320) in DMF with excess of DIPEA overnight at room temperature. N-terminal biotinylation and fluorescent labeling of WDR5 were performed as described previously. In brief, 10 μM WDR5 was incubated with a 200-fold molar excess of 2PCA-DBCO for 24 h at 37 °C while shaking. The following day, unreacted 2PCA-DBCO was removed using Zeba™ Spin Desalting Columns (7 kDa MWCO; Thermo Fisher, 89882) following the manufacturer’s instructions. The protein was then labeled with a twofold molar excess of Azide-Cy5-biotin (Click Chemistry Tools, CCT1232) and incubated overnight at room temperature (23 ± 1 °C). Excess Cy5-biotin-azide was removed the next day using Zeba™ Spin Desalting Columns equilibrated in 1× PBS.

### Single-molecule data acquisition and analysis adapted from ref. ^53^

Single-molecule flow cells were prepared as previously described. After assembly of a microfluidic chamber, the slides were incubated with 20 μL of 0.1 mg/mL neutravidin (Thermo Fisher: 31000) for 2 min. Excess neutravidin was removed with 100 μL 1x PBS. Next, 50 μL of 75 pM Cy5-biotin-azide-labeled WDR5 was added to the microfluidic chamber. After 2 min of incubation, the unbound protein was washed away with 200 μL PBS. Then, 50 μL of 100 pM of Cy3-labeled peptides in imaging buffer (2.5 mM PCA (Sigma: 37580), 0.155 U/μL PCD (OYC Europe: 46852004) and 1 mM 6-hydroxy-2,5,7,8-tetramethylchroman-2-carboxylic acid (Trolox) (Sigma: 238813) in 1x PBS) was injected. Fluorescence signals are collected at 0.1 s exposure time. During the acquisition of the movie, the green laser is used to excite the Cy3 donor fluorophores. The fluorescence images were analysed by a custom script written in Python. The script collects the individual intensity hotspots in the acceptor channel and pairs them with intensity hotspots in the donor channel, after which the time traces are extracted. A two-state K-means clustering algorithm was applied on the sum of the donor and acceptor fluorescence intensities of individual molecules to determine an intensity threshold, with which the traces were divided into high- or low-intensity segments. The high-intensity segments that lasted for more than three consecutive frames were selected for further analysis. The kinetic rates are determined using the following equations.

The dissociation rate (*k*_off_) is calculated as follows:

1 $${k}_{{off}}=\frac{1}{{{{{\rm{\tau }}}}}_{{{{\rm{b}}}}}}$$

Where τ_b_ is the dwell-time of the macrocycle binding events.

The association rate (*k*_on_) is calculated as follows:

2 $${k}_{{on}}=\frac{1}{{{{{\rm{\tau }}}}}_{{{{\rm{ub}}}}}}\cdot c$$

Where τ_ub_ is the time in between binding events and *c* is the macrocycle concentration. The dissociation constant *K*_D_ is calculated as follows:

3 $${K}_{D}=\frac{{k}_{{off}}}{{k}_{{on}}}$$

### Serum stability assay

A 25% human serum solution was prepared in 1xPBS (pH = 7.4). Macrocycle stocks were diluted to a final concentration of 25 μM in the 25% serum solution. The mixture was vortexed immediately after protein addition. At different time points, a sample was retrieved, and the degradation reaction was quenched by the addition of 20% trichloroacetic acid (w/w) in 1x PBS supplemented with Fmoc-Lys(Ac)-OH as internal standard (IS) to a final concentration of 12.5 μM peptide and 12.5 μM IS. After incubating on ice for 1 h, the samples were centrifuged at 10,000×*g* for 5 min. The supernatant was then injected into the UPLC–HRMS (20 μL) and the AUC of both peptide and IS calculated by analyzing the XIC curve for each exact mass value. For each run and timepoint, the peptide AUC was then normalized to the IS AUC. The relative stability values were then plotted using GraphPad Prism 10.1.0 and a one-phase decay non-linear fit curve. Half-life times (t_1/2_) were calculated at the intersection of the curve with y = 50%.

### Parallel artificial membrane permeability assay (PAMPA)

Membrane permeability of cyclic peptides was determined using a pre-coated PAMPA plate system (Corning BioCoat, 353015). The plate system consisted of a 96-well microwell plate (donor wells) and a 96-well insert containing a PVDF membrane coated with tri-layers of phospholipids (acceptor wells). Permeability was measured from the bottom plate (donor wells) to the top insert (acceptor wells). Cyclic peptide or reference compound solutions were prepared at a concentration of 100 μM in 1xPBS buffer (pH 7.4) containing 1% DMSO, and 300 μl of the resulting solutions was placed into the donor wells of the PAMPA plate. Acceptor wells were prepared by adding 200 μl of PBS buffer (pH 7.4) containing 1% DMSO. The insert was stacked onto the donor plate, and the system was covered with a lid to avoid evaporation. After incubating the plates for 18 h at room temperature, the acceptor solutions were analyzed by UPLC–HRMS to determine permeable cyclic peptide concentrations. Additionally, 3:2 dilutions of the 100 μM cyclic peptide stocks were prepared in 1xPBS buffer (pH 7.4) containing 1% DMSO to yield a sample corresponding to the theoretical equilibrium between donor and acceptor wells. The samples were run on the UPLC–HRMS to measure the cyclic peptide concentration representing maximum permeability. Permeability percentage and apparent permeability were calculated using the equation below.

4 $${Permeability}\,\left(\%\right)=\,\frac{[{{Peptide}}_{{Acceptor}}]}{[{{Peptide}}_{{Equillibrium}}]}$$

where [*Peptide*_*Acceptor*_] represents the AUC measured on the UPLC–HRMS for the acceptor wells and [*Peptide*_*Equillibrium*_] represents the AUC measured for the theoretical equilibrium. AUCs were determined based on the XIC curve for each exact mass value.

Apparent permeability:

5 $${P}_{{app}}=\frac{{V}_{D}\times {V}_{A}}{\left({V}_{D}+{V}_{A}\right)\times A\times t}\times -{{{\mathrm{ln}}}}\left(1-\frac{\left[{{Peptide}}_{{Acceptor}}\right]}{\left[{{Peptide}}_{{Equillibrium}}\right]}\right)$$

where *V*_*D*_ and *V*_*A*_ represent, respectively, volume of donor (0.3 mL) and acceptor (0.2 mL), A represents membrane area (0.3 cm^2^), and *t* represents incubation time (18 h, 64.800 s).

### Physicochemical characterization of macrocycle 25, adapted from ref. ^57^

#### Chromatographic environments

The mobile phases for every descriptor consisted of isocratic solutions of 20 mM ammonium acetate at pH 7.0 and acetonitrile at various percentages (see the specific descriptor). Macrocycle 25 was dissolved in buffer/acetonitrile mixtures (v/v) at concentrations ranging from 50 to 100 μg/mL. Subsequently, 10 μL of each solution (injection volume) was injected at an isocratic flow rate of 1 mL/min and analyzed at 30 °C (oven temperature). Chromatographic measurements were then analyzed in duplicate based on the specific chromatographic conditions of each descriptor.

#### PLRP-S system

We measured the RT of every compound in the data set at six mobile phase conditions (50 to 100% MeCN) using the PLRP-S column. Next, we calculated the capacity factor (log *k*’ PLRP-S) using the equation below:

6 $$\log {k}^{{\prime} }\left(\%{MeCN}\right)=\frac{\log [{t}_{R}-{t}_{0}]}{{t}_{0}}$$

Where *t*_0_ is the dead time, and plotted it at each mobile phase composition (% *MeCN*).

#### Chamelogk

To quantify chameleonicity, the mobile phase conditions that respected a linear behavior for bRo5 compounds (50, 60, and 70% MeCN) were selected to build a linear trend. In addition, 95% MeCN was selected as the mobile phase condition with the highest capacity factor change and chosen for comparison and Chamelogk calculation. Moreover, acetone, caffeine, phenol, and a mixture of uracil, acetophenone and toluene were used as gold standards.

#### XBridge system

*BR*logD required the injection of samples into the X-Bridge column at 60% MeCN (predominant mobile phase constituent). The retention times and dead time (t_0_, baseline interference) were recorded, and the capacity factor log *k*’_60_ was calculated by adapting the log *k*’ equation. Lastly, the *BR*logD value was calculated from the equation below:

7 $${BR}\!\log D=3.31\times \log {k^{\prime} }_{60}+2.79$$

In this case, *BR*logD required the measurement of acetone, caffeine, ibuprofen, lidocaine, phenol, and a mixture of uracil, acetophenone, and toluene as gold standards.

#### IAM (immobilized artificial membrane) system

Log *k*_W_^IAM^ involved the injection of the samples into the IAM column at different mobile phases (from 10 to 50% MeCN). The retention times of the samples were recorded, and the capacity factor was calculated for each mobile phase condition, adapting the log *k*’ equation, where *t*_0_ is the retention time of citric acid. Besides, five standards (caffeine, carbamazepine, ketoprofen, theobromine, and toluene) were examined on a daily basis. Finally, the log *k*_W_^IAM^ value for each compound was calculated by extrapolating from the equation obtained with the five mobile phase conditions (10 to 50% MeCN) the capacity factor at a completely aqueous environment (100% buffer/0% MeCN). Δ log *k*_W_^IAM^ was previously defined by Grumetto et al. as

8 $$\Delta \log {k}_{W}^{{IAM}}={experimental}\log {k}_{W}^{{IAM}}-{{{\rm{clog}}}}{k}_{W}^{{IAM}}$$

with clog *k*_W_^IAM^ being the log *k*_W_^IAM^ value for nonpolar and neutral compounds with PSA = 0. Moreover, clog *k*_W_^IAM^ was correlated with the log *P* value (octanol/water) and afterward with the chromatographic descriptor *BR*logD using the equation below:

9 $${{{\rm{clog}}}}{k}_{W}^{{IAM}}={BR}\!\log D\times 0.92-1.03$$

### Cell viability

5000 HL-60 (DSMZ ACC3), 3000 MV411 (DSMZ ACC554), and 7000 MOLM13 (DSMZ ACC554) cells were plated into an opaque-colored 96-well plate (Greiner Bio-One cat no 655083). Cells were plated in triplicates in the presence of WDR5 compounds, in a twofold dilution series from 50 to 0.39 μM. WDR5 compounds were diluted in medium containing 2.5% DMSO. This results in a final DMSO concentration of 0.5%. Cell viability was measured at 72 h using the CellTiter-Glo® assay (Promega, cat no G7570). About 100 μl CellTiter-Glo reagent was added to each well, and the plates were shaken for 5 min at 500 rpm. Luminescent signal was stabilized by incubating the plates for 10 min at RT. Luminescence was measured on the Victor X3 plate reader (Perkin Elmer).

### Pharmacophore-based modeling

Using the published ligand-bound x-ray crystal structure of WDR5 (PDB: 6UCS) in the pharmacophore modeling tool “Pharmit”11, three pharmacophore interactions were defined in the bound ligand. These interactions served to constrain the macrocycle to form comparable interactions with the published ligand, while no constraint would be placed on the orientation or position of the additional building blocks present in the hit. Two H-bond donor interactions were defined deep in the S2 pocket, with an additional hydrophobic interaction defined at the entrance of S2. Taken together, these nodes mimicked the functional composition of an arginine residue. Next, low-energy poses for the macrocycle hit were generated in Pharmit, and conformers were aligned with the prepared pharmacophore model. >150 conformations were found as matches. An energy minimization step was performed, and an energy score filter (<−6) and maximum mRMSD (<4.0) was set to eliminate low-quality poses.

### PEAKS11

De novo peptide sequencing was performed by processing .raw files obtained from Orbitrap analysis using PEAKS Studio (version 11) from Bioinformatics Solutions Inc. (ON, Canada). HCD scans were merged within a 0.2 min and 0.02 Da window, mass precursor correction was used, and primary mass filtration was employed as appropriate. Auto de novo sequencing was performed using a 15 ppm precursor mass error and 0.02 Da fragment mass error, and with the modifications present in the table below correlated to the exact mass of alanine. Modifications were inserted either as fixed or variable. (+XX.XX) refers to the correlation between the unnatural building block and alanine, following the formula XX.XX = (Exact mass unnatural building block) − (Exact mass alanine) or as a fixed modification (for the case of Mpa and Lys). The maximum number of PTMs per sequence was defined as the same as the number of variable positions in the library scaffold. Ten candidate sequences were obtained for each preprocessed scan. Post-de novo data filtering was performed using Knime and Python workflows aimed at eliminating sequences with ALC below 80% as well as ensuring compliance with library design (peptide length and scaffold). Recalls are calculated using (number of found sequences)/(theoretical diversity of the library)*100 (%).

### Library enumeration and property calculation using Knime

Virtual generation of macrocyclic libraries were based on the following general protocol: Building blocks were supplied as SMILES in Tables, and reactions were supplied as SMARTS. Library enumeration workflow: (1) C-terminal amidation of Fmoc-Lys(Boc)-OH via RDKit Chemical Transformation; (2) Fmoc removal via RDKit One Component Reaction; (3) amide coupling of Fmoc-Cys(Mmt)-OH via RDKit Two Component Reaction; (4) Fmoc removal via RDKit One Component Reaction; (5) The elongation of the molecule was carried with consecutive cycles of Two and One Component reactions for amide coupling and Fmoc removal using all possible building blocks for each coupling position via matrix expansion and uniquifying products; (6) For library CycloSEL-16M, carboxylic acid capping in the exocyclic position was achieved with Two Component Reaction with matrix expansion. Side chain deprotection was carried out by providing deprotection SMARTS to a Chunk Loop enclosing a Chemical Transformation in chunks of 100,000 rows per cycle; (7) Macrocyclization was achieved by providing disulfide bridge formation SMARTS to a Chunk Loop enclosing a Chemical Transformation in chunks of 100,000 rows per cycle; (8) Molecular properties were calculated by feeding the enumerated library to a RDKit Descriptor Calculation node including sLogP, TPSA, ExactMW and NumHBD calculation features. All SMARTS reactions can be found in Supporting Information Section 4.1.3. Table S3.

### Reporting summary

Further information on research design is available in the Nature Portfolio Reporting Summary linked to this article.

## Supplementary information

Supplementary Information

Reporting Summary

Transparent Peer Review file

## Source data

Source data

## Data Availability

All data were available in the main text or the supplementary information, and from the corresponding author(s) upon request. Raw nLC-MS/MS files and raw single-molecule fluorescence can be found at DOI: 10.5281/zenodo.18936386. Source data are provided with this paper.
