# Supplementary material for: Massive barcode-free chemical screenings enable the discovery of bioactive macrocycles with passive membrane permeability
Source: Nat Commun. 2026 Apr 10;17:5034. doi: 10.1038/s41467-026-71641-3 (PMC13243568; doi:10.1038/s41467-026-71641-3)
Supplement: Supplementary file 1 — Supplementary Information [file 41467_2026_71641_MOESM1_ESM.pdf]

Supplementary Information for

Massive barcode-free chemical screenings enable the discovery of bioactive macrocycles with passive membrane permeability

J. Miguel Mata,[1,2,+] Jingming Liu,[1,2,+] Sean M. McKenna,[1,2] Edith van der Nol,[1,2] Marije Havermans,[2,3] Ruud Delwel,[2,3] Mike Filius[1,2,4], Chirlmin Joo[4], Maura Vallaro[5], Giulia Caron[5], Sebastian J. Pomplun*[1,2]

[1]LACDR, Leiden University; Leiden, 2333 CC, The Netherlands

[2]Oncode Institute; Utrecht, 3521 AL, The Netherlands

[3]Department of Hematology, Erasmus MC Cancer Institute, Rotterdam, Netherlands.

[4]Kavli Institute of NanoScience, Department of BioNanoScience, Delft University of Technology, Delft, 2628 CJ, The Netherlands

[5]Molecular Biotechnology and Health Sciences Department., CASSMedChem, University of Torino, via Quarello 15, 10135 Torino, Italy

*Corresponding author. Email: s.j.pomplun@lacdr.leidenuniv.nl

+These authors contributed equally to this work

Contents

1. Supplementary Figures 4
1.1 General procedure for macrocycle synthesis 4
1.2 TCEP opening studies 5
1.3 Library design optimization 6
1.4 Decoding bias analysis 7
1.5 CycloSEL-16M library quality control 8
1.6 Building block enrichment for CAIX selection 9
1.7 Biotinylated and native WDR5 validation 10
1.8 Binding curve of compound 11 and 16 to WDR5 11
1.9 Passive permeability of compounds 17-20 and s1-s4 12
1.10 General synthesis procedure for C-terminal amide-free clipped macrocycles 13
1.11 SAR studies for WDR5 binder 17Δ: Competition binding assay for C-terminal amide free, N-methylated variants and Lys-based cyclization linker 14
1.12 IC_{50} curve for compound 17Δ 15
1.13 Cell proliferation assays 16
2. Abbreviations 17
3. Materials and Methods 19
3.1 General information 19
3.1.1 Reagents and supplies 19
3.1.2 Instrumentation 19
3.1.3 General procedures 20
4.1.1 Protein expression, purification and quality control 29
4.1.2 Cell viability 30
4.1.3 Software and workflows for data analysis 30
5. Building block dictionary: AAs and CAs and exact mass difference for *de novo* sequencing 34
6. Library designs 42
7. Sequencing outcome for library scaffold optimization 45
7.1 $C(X)_3CK$ design: 45

7.2 $C(X)_4CK$ design: 46
7.3 $AcC(X)_3CK$ design: 48
7.4 $AcC(X)_4CK$ design: 49
7.5 $Mpa(X)_3CK$ design: 51
7.6 $Mpa(X)_4CK$ design: 52
8. Affinity selection against CAIX 54
8.1 Procedure 54
8.2 List of filtered sequences 54
8.3 Post-sequencing multi-stage manual triage (with examples) 63
8.4 Enrichment and p-value calculations for CAIX selection 67
8.5 Hit selection and manual inspection 68
9. Affinity Selection against WDR5 71
9.1 Procedure 71
9.2 List of filtered sequences 71
9.3 Hit selection and manual inspection 73
10. Compound information 75
11. LCMS data 98
12. Supplementary References 110

1. Supplementary Figures

1.1 General procedure for macrocycle synthesis

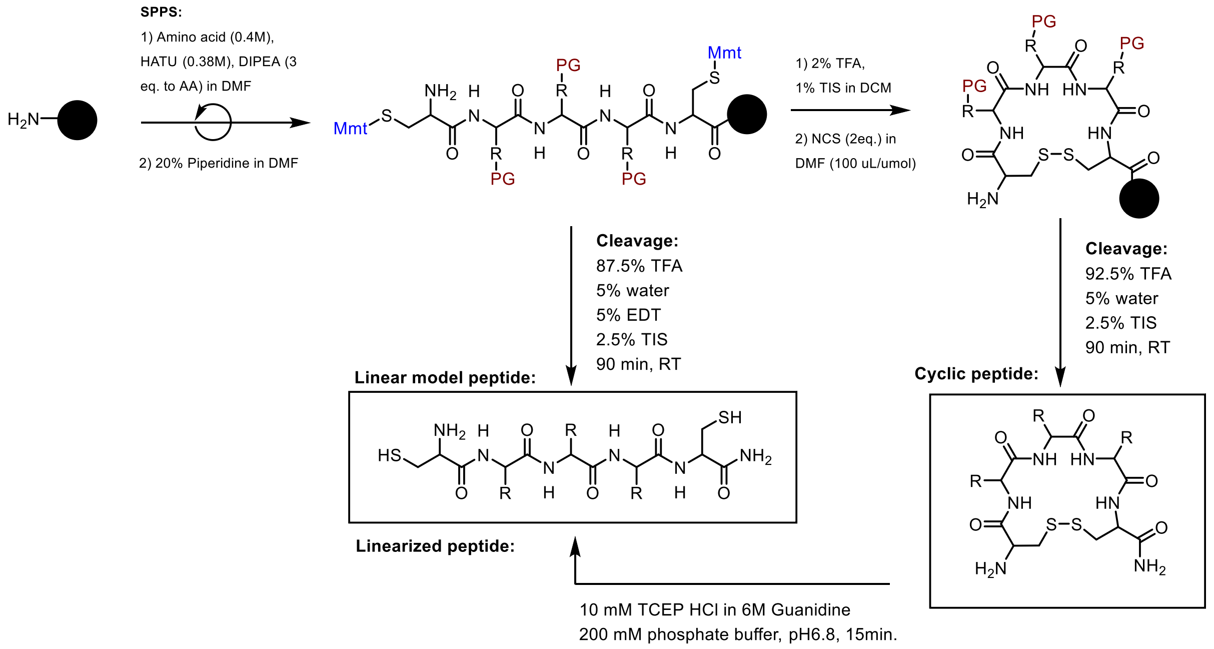

Figure S1: General synthesis route for the generation of linear peptides and cyclic and linearized macrocycles. R = amino acid side chain.

1.2 TCEP opening studies

Minimum required TCEP equivalents: Cyclic peptide **3** and **3c** was incubated with several equivalent amounts of TCEP HCL in 6M Guanidine 200 mM phosphate buffer pH 6.8 for 15 min under agitation at room temperature. After 15 min, HRMS analysis was carried out showing complete reduction of cyclic to linear peptide with as low as 1 eq. of TCEP.

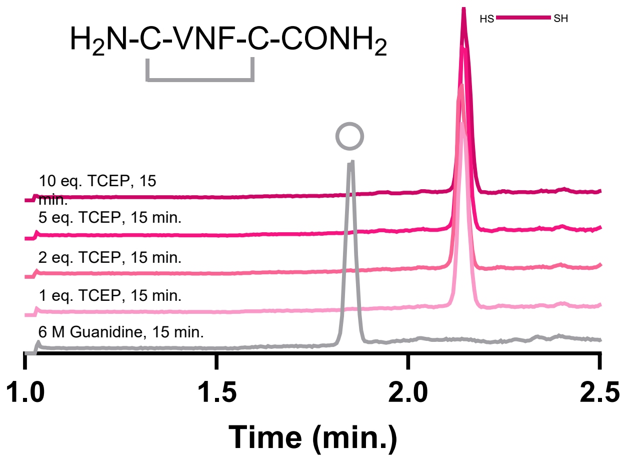

Figure S2: TIC for linear and linearized peptides across a panel of different TCEP equivalents.

1.3 Library design optimization

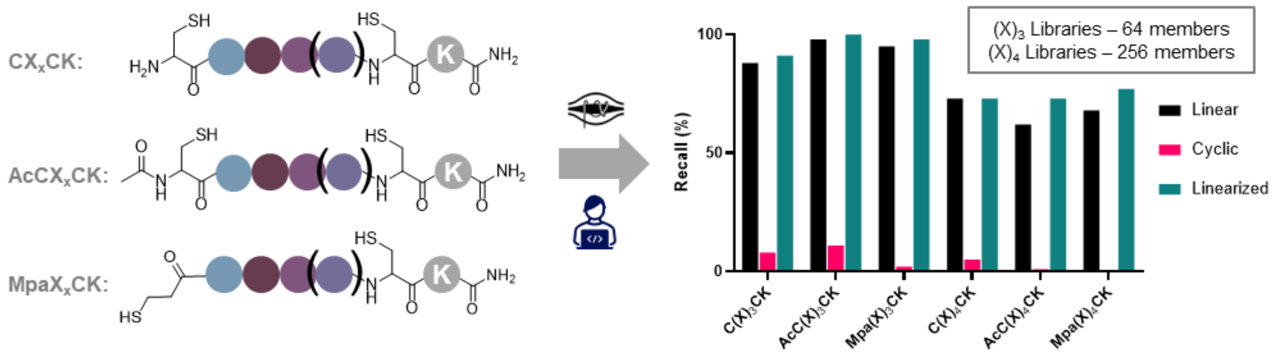

Figure S3: Library design optimization based on sequencing recall. Recall was calculated using the formula (found library sequences)/(library design sequences).

1.4 Decoding bias analysis

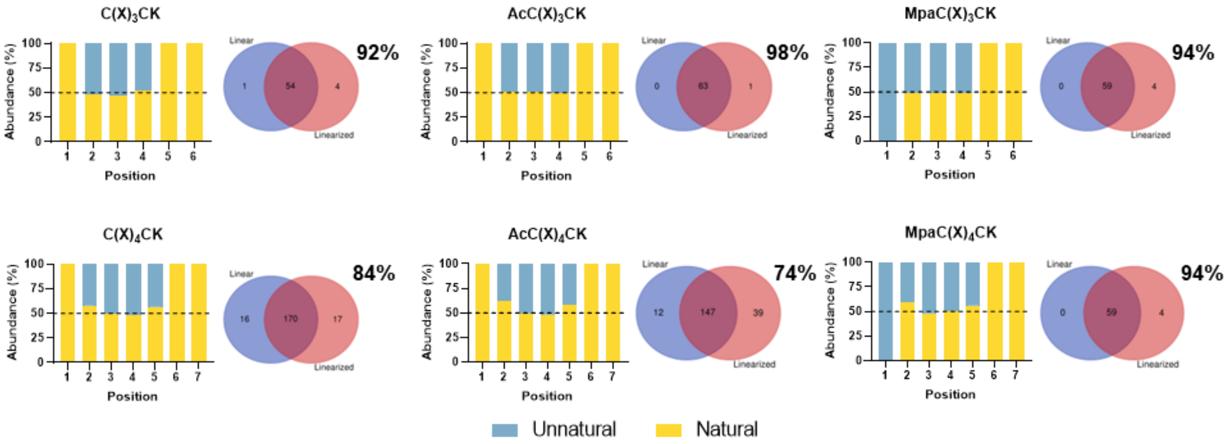

Figure S4: Unbiased MS/MS sequencing of natural and unnatural amino acids (blue and yellow plot). MS/MS of linear vs linearized libraries leads to robust and overlapping sequence identities. (Venn diagrams).

1.5 CycloSEL-16M library quality control

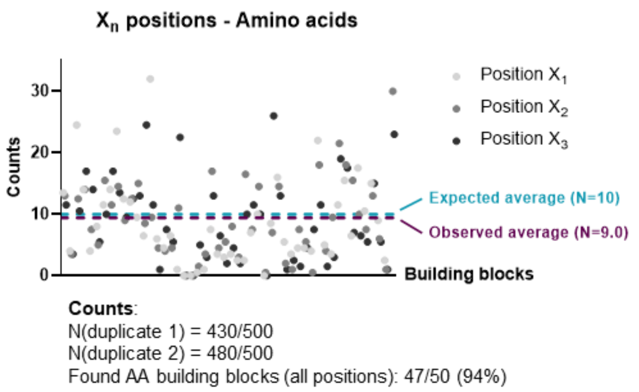

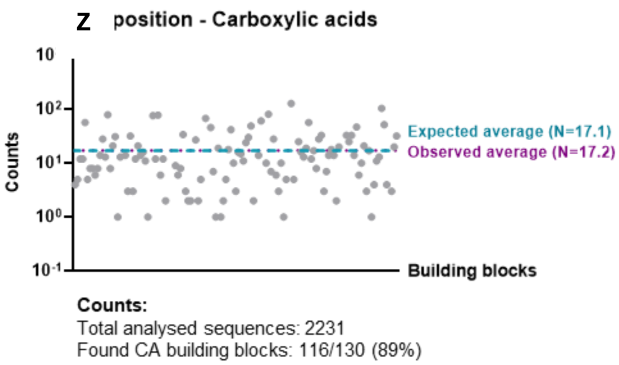

Figure S5: Quality control to CycloSEL-16M. QC to X_n and Z positions show detection of 94% and 89% respectively of all AA and Z building blocks used across 910/1000 and 2231 detected sequences and an expected building block distribution matching the theoretical distribution.

1.6 Building block enrichment for CAIX selection

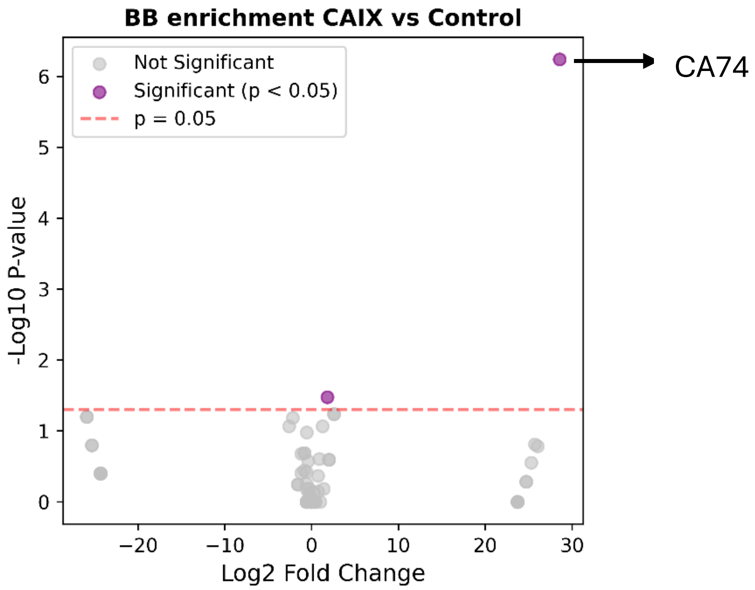

Figure S6: Vulcano plot of the enriched building blocks in an exemplary selection sample of CAIX against control sample (streptavidin + CycloSEL-16M).

1.7 Biotinylated and native WDR5 validation

UPLC-HRMS of expressed WDR5:

Calculated mass: 36841.75 Da

Found mass: 36837.7 Da

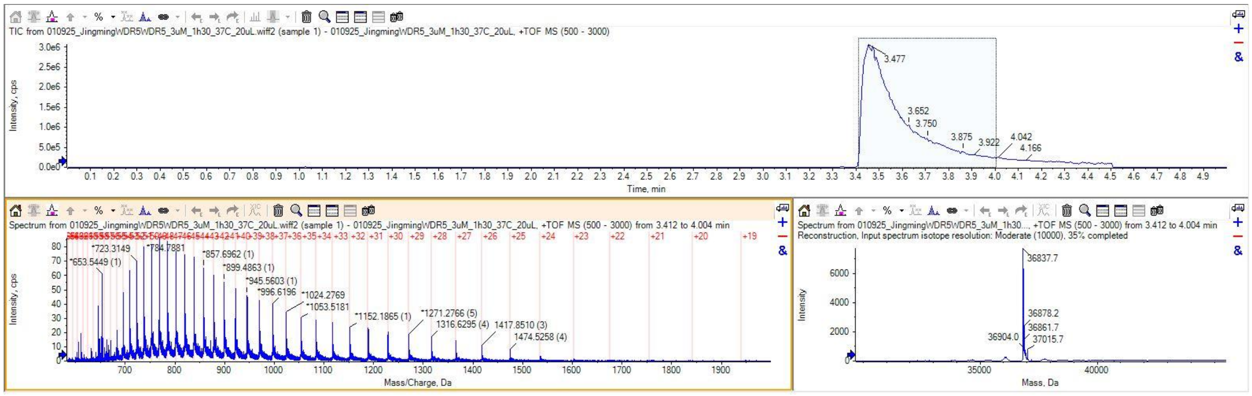

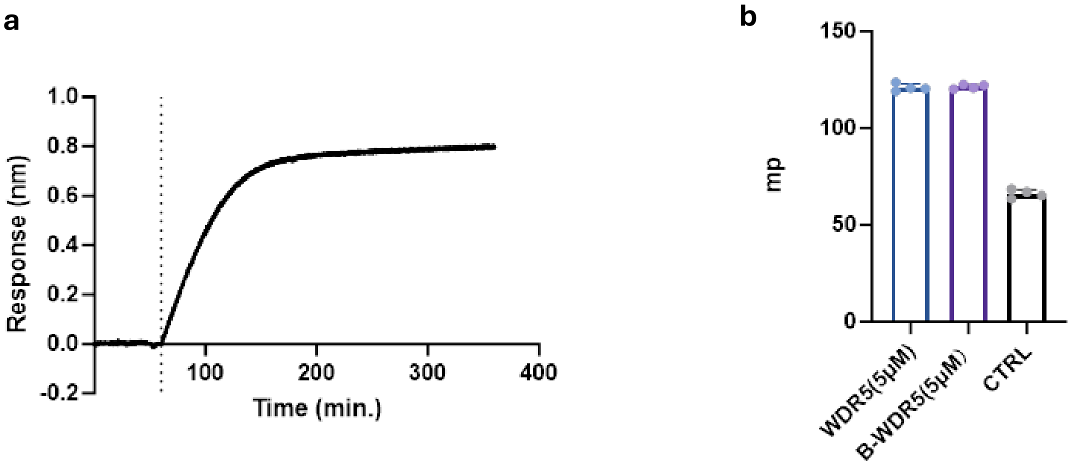

Figure S7: a) Association curve for manually biotinylated WDR5. Sensors were baselined for 60s before dipping in biotinylated WDR5 solution (200nM). **b)** Fluorescence polarization assay comparing the activity of WDR5 and biotinylated WDR5 (5μM protein, 10 nM Ac-ARTEVY-βA-FITC).

1.8 Binding curve of compound 11 and 16 to WDR5

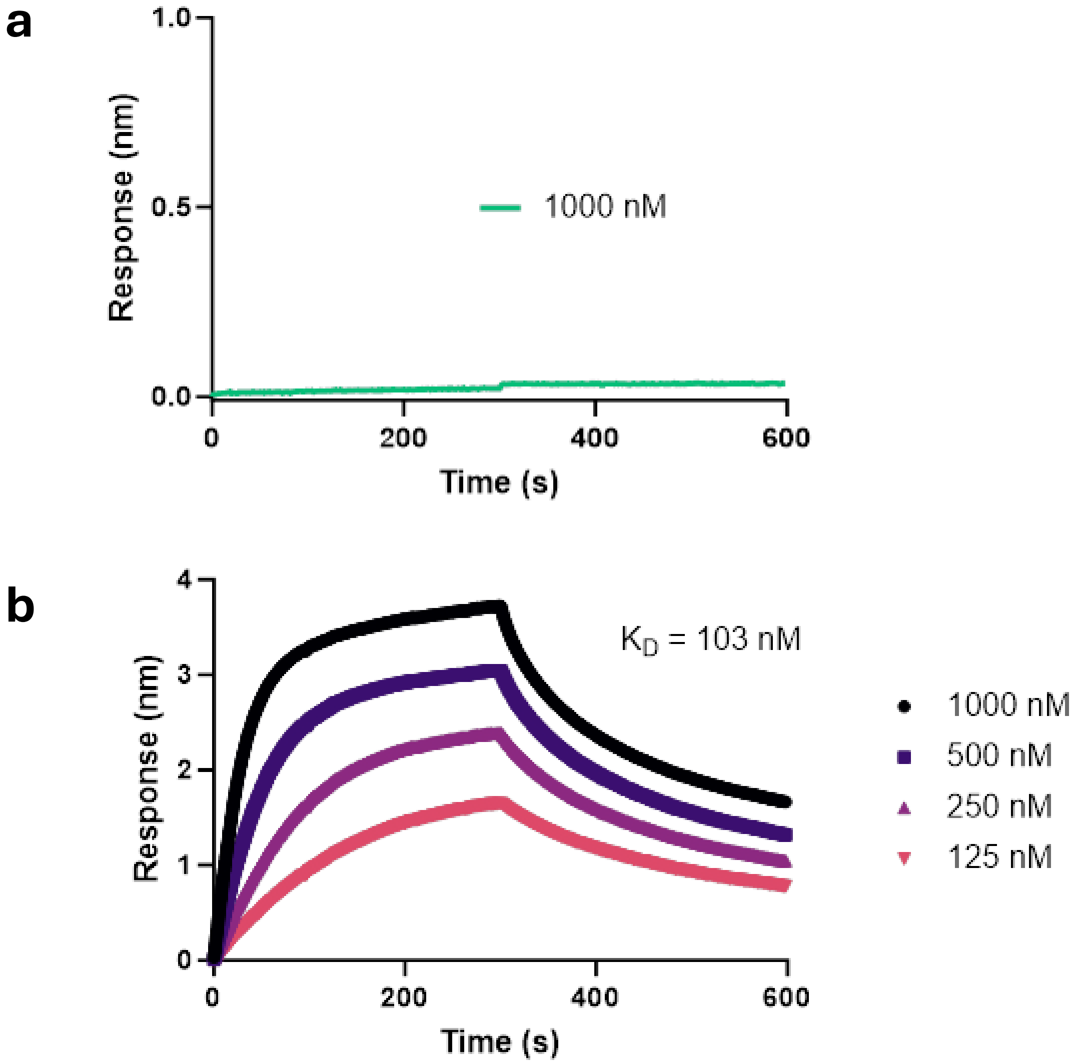

Figure S8: a) BLI for compound **11** against fixed concentration of WDR5. **11** was immobilized at 1 μM for 60 seconds on SA sensors and incubated in a concentration range of WDR5. **b)** BLI for compound **16** against a concentration range of WDR5. **16** was immobilized at 1 μM for 60 seconds on SA sensors and incubated in a concentration range of WDR5.

1.9 Passive permeability of compounds 17-20 and s1-s4

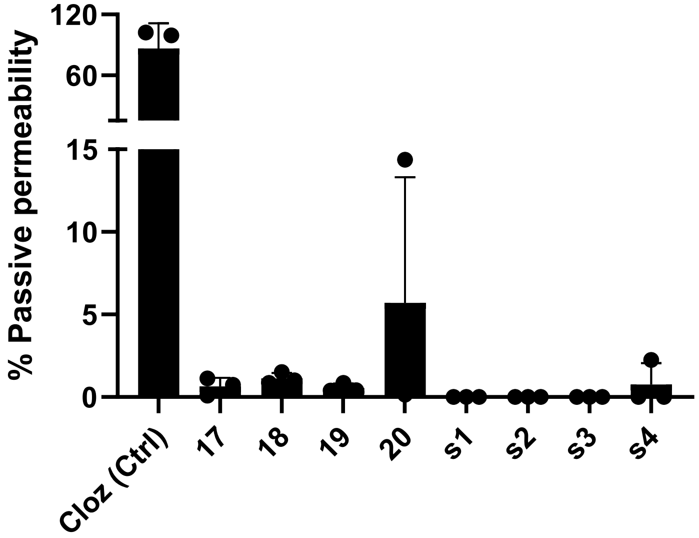

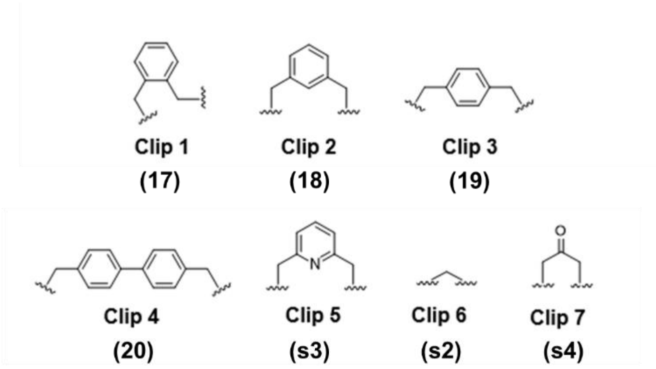

S1 – linear variant of 14

Figure S9: Passive permeability measured by PAMPA of variations of compound **14.**

1.10 General synthesis procedure for C-terminal amide-free clipped macrocycles

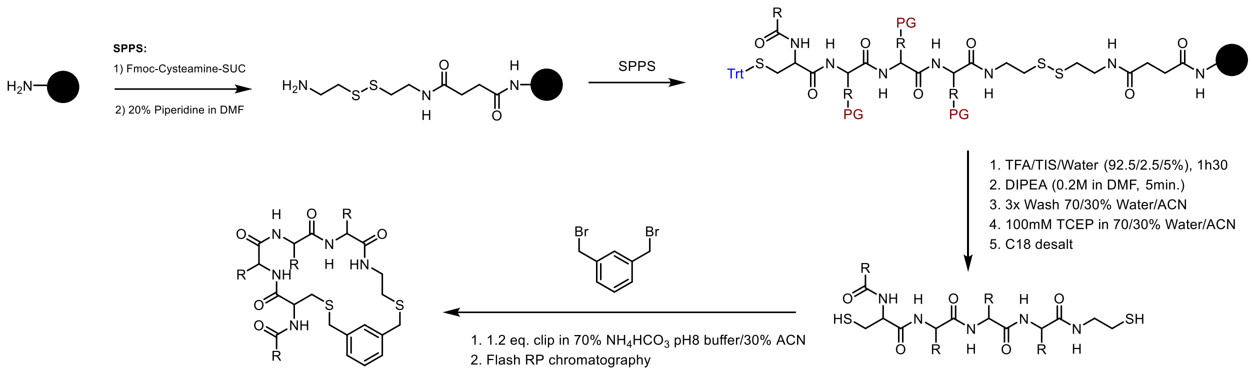

Figure S10: General synthesis route for the generation of clipped macrocycles without a C-terminal. R = amino acid chain.

1.11 SAR studies for WDR5 binder 17Δ: Competition binding assay for C-terminal amide free, N-methylated variants and Lys-based cyclization linker

Compound	Position Y	Linker N-term	Position X1	Position X2	Position X3	Linker C-term	Cyclization	Binding (ratio to control)	Permeability (%)
**17Δ**	CA116	Cys	AA23	Arg	Trp	Cysteamine	Clip 1	0.18	17.17
**s5**	CA116	N-Cys	AA23	Arg	Trp	Cysteamine	Clip 1	0.37	not tested
**s6**	CA116	Cys	AA23	Arg(Me)	Trp	Cysteamine	Clip 1	0.27	not tested
**s7**	CA116	Cys	AA23	Arg	N-Trp	Cysteamine	Clip 1	0.83	not tested
**s8**	CA116	Cys	AA23	Arg	Nal	Cysteamine	Clip 1	0.81	not tested
**s9**	CA116	N-Cys	AA23	Arg(Me)	Trp	Cysteamine	Clip 1	0.15	7.82
**s10**	CA116	N-Cys	AA23	Arg(Me)	Nal	Cysteamine	Clip 1	0.99	not tested
**s11**	CA116	Cys	AA23	Arg(Me,Me)	Trp	Cysteamine	Clip 1	0.14	not tested
**s12**	CA116	N-Cys	AA23	Arg(Me)	Trp(Me)	Cysteamine	Clip 1	0.17	5.73
**s13**	CA116	Orn	AA23	Arg(Me)	Trp(Me)		Macrolactamization	0.17	1.36
**s14**	CA116	Lys	AA23	Arg(Me)	Trp(Me)		Macrolactamization	0.07	0.64
**s15**	CA116	hLys	AA23	Arg(Me)	Trp(Me)		Macrolactamization	0.07	1.82
**s16**	CA116	N-Lys	AA23	Arg(Me)	Trp(Me)		Macrolactamization	0.91	not tested
**s17**	CA116	N-Lys(Me)	AA23	Arg(Me)	Trp(Me)		Macrolactamization	0.94	not tested
**s18**	CA116	Lys(Me)	AA23	Arg(Me)	Trp(Me)		Macrolactamization	0.26	1.12
**s19**	CA116	Lys	AA23	Ala	Trp(Me)		Macrolactamization	0.99	0.91
**s20**	No CA	Lys	AA23	Arg(Me)	Trp(Me)		Macrolactamization	0.28	1.09
**s21**	CA52	N-Lys	AA23	Arg(Me)	Trp(Me)		Macrolactamization	0.10	5.63
**s22**	CA102	N-Lys	AA23	Arg(Me)	Trp(Me)		Macrolactamization	0.16	1.20
**s23**	CA134	N-Lys	AA23	Arg(Me)	Trp(Me)		Macrolactamization	0.09	15.06

Figure S11: Simultaneous optimization of binding and passive permeability for parent compound **17Δ**. N-AA refers to backbone amide methylation and AA(Me) refers to side chain methylation.

1.12 IC_{50} curve for compound 17Δ

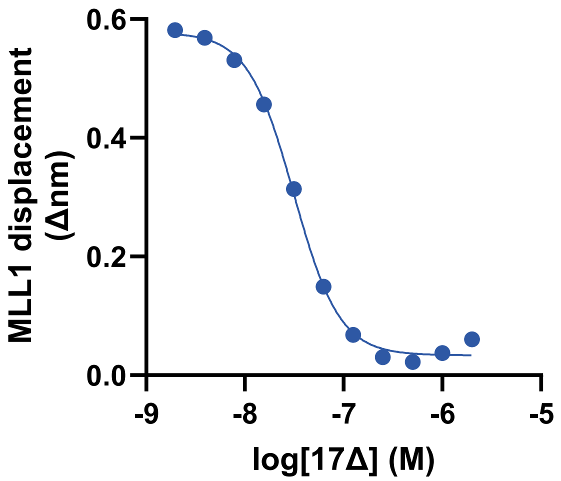

IC_{50} = 32 nM

Figure S12: BLI competition curve of compound **17Δ** against WDR5:compound **16**. Compound **16** was immobilized for 60 sec. at 100 nM. WDR5 (50nM) and compound **17Δ** (carrying concentrations) were reincubated and association was measured for 600 sec.

1.13 Cell proliferation assays

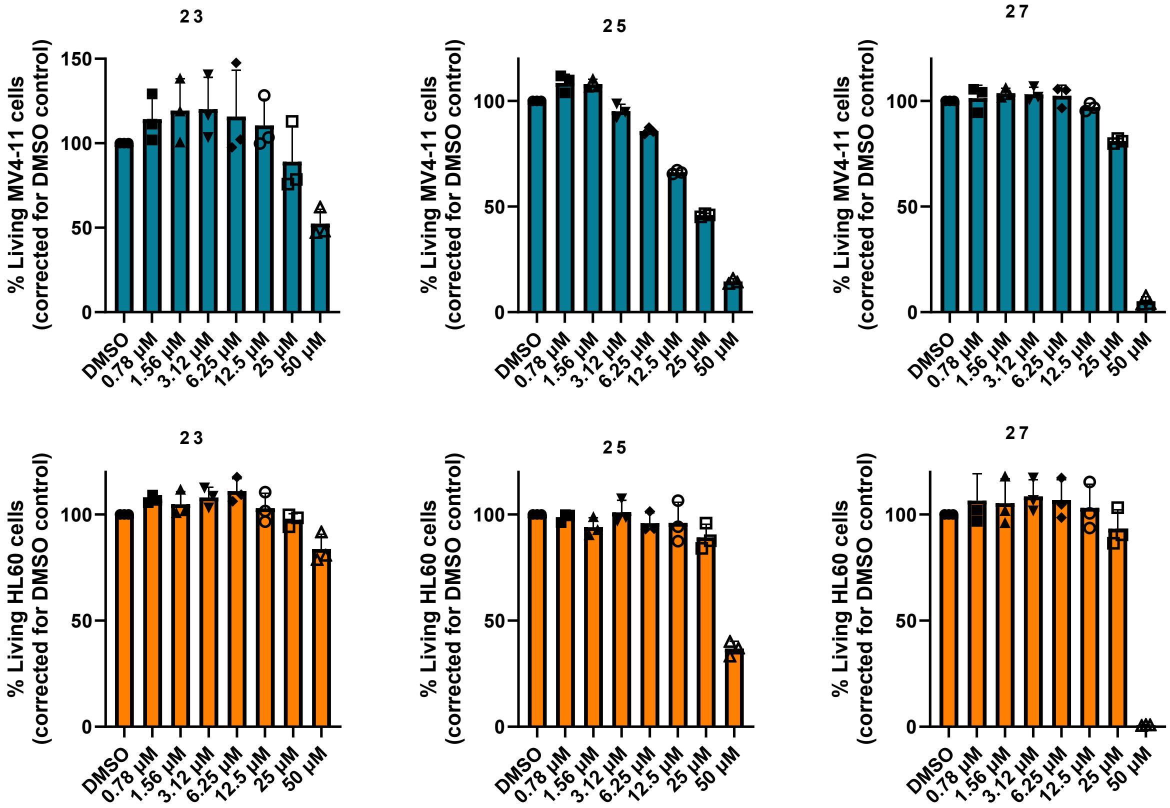

Figure S13: Cellular viability after treatment with cyclic peptides **23**, **25** and **27** in MV4-11 and HL60 cells via CellTiterGlo® assay. Mean values (bars) and SDs are shown for technical triplicates measured on the same plate.

2. Abbreviations

AA	Amino acid
Ac	Acetyl
Ac2O	Acetic anhydride
ADME	Absorption, distribution, metabolism, and excretion
ALC	Average level of confidence
AML	Acute myeloid leukemia
AS	Affinity selection
AUC	Area under the curve
BB	Building block
BLI	Biolayer interferometry
Boc	tert-Butyloxycarbonyl
bRo5	Beyond rule of 5
BSA	Bovine serum albumin
CA	Carboxylic acid
CAIX	Carbonic anhydrase IX
ACN	Acetonitrile
Cloz	Clozapine
DBU	1,8-Diazabicyclo[5.4.0]undec-7-ene
DCM	Dichloromethane
DEL	DNA-encoded library
DIC	N,N'-Diisopropylcarbodiimide
DIPEA	N,N-Diisopropylethylamine
DMAP	4-Dimethylaminopyridine
DMF	Dimethyl formamide
DMSO	Dimethylsulfoxide
EDT	Ethane-1,2-dithiol
Eq	Eqalent
FA	Formic acid
FBS	Fetal bovine serum
Fmoc	9-fluorenylmethyloxycarbonyl
KB	Kinetic buffer
HATU	Hexafluorophosphate Azabenzotriazole Tetramethyl Uronium
HBA	H-bond acceptor
HBD	H-bond donor
HCD	High-collision energy
LC	Liquid chromatography
MLL	Mixed Lineage Leukemia
Mmt	Monomethoxytrityl
Mpa	Mercaptopropionic acid
MQ	MilliQ

mRMSD	Median root mean square deviation
MS	Mass spectrometry
MS/MS	Tandem mass spectrometry
o-NBS-Cl	2-Nitrobenzenesulfonyl chloride
NCS	N-chlorosuccinimide
NMP	N-Methyl-2-pyrrolidone
OtBu	tert-butoxide
PAMPA	Parallel artificial membrane permeability assay
Pbf	2,2,4,6,7-Pentamethyl-2,3-dihydrobenzofuran-5-sulfonyl
PBS	Phosphate-buffered saline
PDB	Protein data bank
PDVF	Polyvinylidene fluoride
PPI	Protein-protein interaction
QTOF	Quadrupole Time-of-Flight
RP	Reverse-phase
RT	Room temperature
SEL	Self-encoded library
SPPS	Solid-phase peptide synthesis
TCEP	Tris(2-carboxyethyl)phosphine
TFA	Trifluoroacetic acid
TIC	Total ion Chromatogram
TIS	Triisopropylsilane
TOF	Time of flight
TPSA	Topological polar surface area
Trt	Trityl
WDR5	WD repeat domain 5
XIC	Extracted ion chromatogram

3. Materials and Methods

3.1 General information

3.1.1 Reagents and supplies

Chemicals: Reagents and solvents were purchased from Sigma-Aldrich (Merck), Fisher Scientific, BLDpharm, Fluorochem or VWR and were used without further purification unless stated otherwise. Carboxylic acids, aldehydes and Fmoc-protected amino acids building blocks were partially purchased from Chemspace. Tentagel® S NH2 90 µm (S30902) and TentaGel® M NH_2 30 µm (M30352) were purchased from Rapp-Polymere. Rink Amide ProTide Resin was purchased from CEM. H-Rink amide ChemMatrix® resin was purchased from Sigma-Aldrich.

Materials for affinity selection: Dynabeads MyOne Streptavidin T1 were purchased from ThermoFisher Scientific. A KingFisher™ Duo Prime Purification System was used to perform our affinity selection experiments. The protocols were developed with BindIt 4.1 Software.

Proteins: Biotinylated Human Carbonic Anhydrase IX (38-414), His,Avitag (CA9H82E3) was purchased from ACROBiosystems. WDR5 was expressed as described in section 3.1.4.

3.1.2 Instrumentation

UPLC-HRMS analysis: Compounds were analysed by LC–MS, using the Sciex X500b QTOF ESI-QToF mass spectrometer coupled to a Shimadzu Nexera UHPLC LC40DX3 with a Phenomenex Synergi™ 4 µm Fusion-RP 80 Å LC Column (50 x 2 mm) using the following parameters: flow rate = 0.5 mL/min, scan range = 350-1500 m/z, column temperature (°C) = 40.

LC Method: 0% solvent B over 1 min, followed by a linear gradient 0% to 60% solvent B over 3.5 min, followed by a linear gradient 60% to 95% solvent B over 0.1 min, followed by 95% solvent B over 0.4 min, followed by a linear gradient 95% to 0% solvent B over 0.5 min, followed by 0% solvent B over 1.5 min, flowrate 0.5 mL/min.

MS Method: Method duration: 5 min; Total scan time: 0.276 sec; Estimated cycles: 1086; Intact protein mode: False; Decrease detector voltage: False; Large protein (>70kDa):False; Ion Source: Source name: TurboIon Spray; Curtain gas: 35 psi; Ion source gas 1: 60 psi; Ion source gas 2: 60 psi; Temperature: 500 °C; Experiment: Scan type: TOFMS; Polarity: Positive; Spray voltage: 5500 V; CAD gas: 7; Time bins to sum: 4;Channel 1-4: True; TOF start mass: 350 Da; TOF stop mass 1500 Da; Accumulation time: 0.25; Declustering potential: 80V; Declustering potential spread: 0V; Collision energy: 10V; Collision energy spread: 0 V; Override Qjet RF value: False.

nanoLC-MS/MS analysis: Analysis was performed on an Vanquish™ Neo UHPLC system (Thermo Scientific) connected to an Orbitrap Exploris 240 mass spectrometer (Thermo Fisher Scientific). Samples were run on a Double nanoViper PepMap Neo column (2 µm particle size, 15 cm x 75 µm, Thermo Fisher Scientific, DNV75150PN) following a PepMap Neo Trap Cartridge (5 µm C18 300 µm X 5 mm). The standard nano-LC method was run without temperature control and a flow rate of 250 nL/min with the following gradient: isocratic gradient of 3% solvent B for 2 min, ramping linearly to

40% B in A over 60 min, followed by linear ramping to 50% over 5 min and consecutively linear ramping to 95% with solvent A = water (0.1% FA), and solvent B = 80% acetonitrile, 20% water (0.1% FA). Positive spray ionization was set at 1900 V. The following parameters were used for MS1 collection: resolution = 60.000, scan range (m/z) = 225-1000 m/z, maximum injection times = 300 ms, RF Lens = 80%, microscans = 1, AGC target = standard, default charge state = +2. For the data dependent MS/MS event the following settings were used: monoisotopic peak determination: peptide, expected LC peak width = 10sec, intensity threshold = 5e4, charged states 1-4, exclusion after 3 times occurring within 30sec, exclusion for 15sec, mass tolerance 10ppm, excluding isotopes, isolation window (m/z) = 2, normalized HCD collision energy = 30%, orbitrap resolution = 15000, first mass (m/z) = 120, normalized AGT target = 50%, microscans = 1, data type = centroid, total scans = 15.

3.1.3 General procedures

Manual solid-phase synthesis (SPPS):

A 5 mL fritted syringe was charged with 100 mg of ProTide® Rink amide resin (loading: 0.6 mmol/g; total: 0.06 mmol). The resin was swollen in 5 mL of DMF for 5 minutes before draining. To remove the Fmoc protecting group, the resin was treated with 5 mL of 20% piperidine in DMF (two cycles: 1 minute and 5 minutes), then drained and rinsed five times with 5 mL of DMF. For each coupling step, the Fmoc protected amino acid (0.18 mmol, 3 equivalents) was dissolved in DMF to reach a 0.25 M concentration. This was mixed with an equal volume of 0.24 M HATU in DMF, resulting in final concentrations of 0.125 M (amino acid) and 0.120 M (HATU). Activation was initiated by adding DIPEA (30 equivalents relative to resin loading) to the mixture. After 15 seconds, the solution was transferred to the resin and allowed to react for 15 minutes with periodic stirring. Following the coupling, the syringe was drained and the resin was washed three times with 5 mL of DMF. The Fmoc group was then removed again using the previous method: 5 mL of 20% piperidine in DMF (1-minute and 5-minute treatments), followed by a final series of five 5 mL DMF washes.

C-terminal amide-free bis-thiol manual peptide synthesis: TentaGel® S NH_2 90 µM resin (loading 0.26 mmol/g, typical scale: 100mg, 0.026 mmol) was loaded into a fritted syringe (5 mL), swollen in DMF (5 mL) for 5 minutes and then drained. Fmoc-Cysteamine-Suc (0.78 mmol, 3 eq) was first coupled to the resin and deprotected as described above. Elongation of the peptide sequence was carried out as described above.

C-terminal amide-free manual peptide synthesis for macrolactamization: Wang resin from Irish Biotech (loading 1 mmol/g, 100mg, 0.10 mmol) was loaded into a fritted syringe (5 mL), swollen in dry DCM (3 mL) for 60 minutes and then drained. In the meantime, a solution of the first Fmoc-amino acid to incorporate (4 eq) was prepared in 500uL of DMF and 3 mL dry DCM were added. The solution was added to the resin followed by 4 eq DIC and 0.1 eq DMAP. The solution was left to shake at room temperature overnight. The amino acid solution was drained and resin washed with 3 x 5mL DCM. Then, the left over unreacted resin was twice acetylated by addition of 5 mL of DMF/Ac_2O/DIPEA

solution (8:1:1 v/v) and left to react for 30 min. The resin was then drained and washed with 3 x 5mL DCM and 3 x 5mL DMF. Peptide elongation was achieved using the protocol described above.

Automatic solid-phase synthesis (SPPS): Peptide automatic synthesis was carried out on a SYRO I Multiple Peptide Synthesizer (Multisyntech) using 100 mg of the Chemmatrix® rink amide resin (loading 0.49 mmol/g, typical scale: 100 mg, 0.05 mmol. The synthesizer was charged with Fmoc-protected amino acid (Fmoc-AA-OH) solutions (0.4 M in DMF), HATU (0.36 M in DMF), Fmoc removal cocktail (20%/2%/78% = piperidine/formic acid/DMF) and pure DIPEA. As the first step in the peptide synthesis, the peptidyl resin was incubated at 70 °C in DMF while shaking for 10 min, after which the following coupling cycle was repeated until completion of the synthesis: Coupling: Fmoc-AA-OH (10 eq., 800 µL for the 41 µmol scale), HATU (9 eq., 800 µL for the 41 µmol scale) and DIPEA (28.7 eq., 100 uL for the 41 µmol scale) were sequentially added to the peptidyl resin and incubated at 70 °C for 8 minutes, with vigorous interval vortexing, followed by vacuum based draining of the resin. Washing: DMF (1.2 mL) was added to the peptidyl resin, incubated for 1 min at room temperature (RT) with vigorous vortexing followed by vacuum based draining of the resin. The step was repeated 3 times. Fmoc removal: Fmoc removal cocktail (1.5 mL) was added to the peptidyl resin incubated at 70 °C for 4 minutes, with vigorous interval vortexing. Washing: DMF (1.2 mL) was added to the peptidyl resin, incubated for 1 min at RT with vigorous vortexing followed by vacuum based draining of the resin. The step was repeated 3 times.

Site-selective backbone amide N-methylation: The methylation protocol was adapted from Helbik-Maciejewska et al[1]. After Fmoc deprotection, the resin was washed 3x5mL with NMP. For the NBS protection step, Compared to resin loading, 4 eq of o-NBS-Cl and 10 eq of DMAP were dissolved in NMP (4 mL per 100mg resin) and added to the resin. The mixture was left to react in an ultrasonic bath for 5 min at RT. After washing 3x5mL with NMP, methylation was achieved with a solution of 3 eq DBU and 10 eq $(Me)_2SO_4$ in NMP (4 mL per 100mg resin) added to the resin and left to react under the same conditions for 15 min. Resin was drained, washed 3x5mL with NMP and DBU/$(Me)_2SO_4$ was repeated once more. After washing and draining, the NBS group was deprotected by the resin was treated with a solution of 10 eq 2-mercaptoethanol and 5 eq DBU in NMP (4 mL per 100mg resin) for 5 min. After washing and draining, the deprotection procedure was repeated. Resin was then washed with 3x5mL DMF and synthesis followed as described above.

Peptide biotinylation: Peptides requiring a biotin tag for testing in kinetic assays were synthesized bearing a Lys-Lys-Lys(biotin) tag. For this, SPPS was used to, before synthesizing the peptide itself, couple Fmoc-Lys(Biotin) followed by to coupling cycles of Boc-Lys(Fmoc)-OH. Synthesis was then proceeded according to the general SPPS protocol.

Mmt deprotection: Resin containing the Mmt-protected biscysteine peptides were subjected to incubation with a solution of 2% TFA, 1% TIS in DCM for 15min under agitation. The procedure was repeated until the resulting deprotection solution ran clear and not bright orange. In between incubations, the resin was washed 3 times with the same solution. Once deprotected, the resin was washed 3 times with DMF.

Disulfide macrocyclization: The cyclization protocol was adapted from Postma et al[2]. Resin containing the deprotected biscysteine peptides was subjected to incubation with 2 eq. of N-chlorosucinimide in DMF (100 uL per µmol of peptide) for 1h. After incubation, the solution was discarded and the resin washed with 3x DMF, 3x DCM and dried before cleavage.

N-terminal acetylation: After deprotection and washing of the last AA building block, the resin was incubated with a mixture of DMF/AC_2O/DIPEA (8:1:1 v/v) for 15 min. After the allotted time, the resin was then washed with 3x5mL DMF and 2x 5mL DCM.

Stapling macrocyclization: dry peptide powder was dissolved in 30% ACN/70% 100mM NH_4HCO_3 pH 8 buffer to a final concentration of 1mM. Then, the correct volume of staple stock was added to the peptide solution to a final concentration of approx. 1mM peptide and 1.2 mM staple (1.2 eq.). The reaction was stirred for 30 min. or until full completion followed by UPLC-HRMS. The crude was then purified using either method A or C, depending on peptide crude purity.

Peptide cleavage: Acidic cleavage/deprotection: Upon completion of the peptide synthesis, the resin was treated with a cleavage cocktail containing 92.5% TFA, 5% water, 2.5% triisopropylsilane (TIS) (v/v) at room temperature for 90 min or with 87.5% TFA, 5% water, 5% 1,2-ethanedithiol and 2% triisopropylsilane (TIS) (v/v) for 90 min (for free cys-containing peptides). The TFA volume was then reduced under nitrogen stream and cold diethyl ether (–20 °C) was added to precipitate the peptide. The resulting suspension was centrifugated at 10000 rpm for 5 min and the liquid was discarded. After repeating this step once more, the pellet was dissolved in 70/30% water + 0.1%FA and acetonitrile + 0.1% FA.

TCEP cleavage from SUC-functionalized resin: after peptide synthesis, SUC-Cysteamine-functionalized Tentagel® S NH2 90 µm resin was swollen in 1:1 MQ water/ACN for 10 min. After draining, the resin was incubate with 100mM TCEP HCl in 1:1 MQ water/ACN (approx. 50uL /µmol peptide on resin) for 1h. The solution was drained and its volume reduced under nitrogen and desalted using purification method C.

Purification: The automatic flash chromatography was performed on a Biotage Selekt System with pre-packed flash cartridges (Biotage® Sfär Bio C18 - Duo 300 Å 20 µm). MQ 0.1% TFA (buffer A) and ACN 0.1% TFA (buffer B) were used as mobile phase.

Method A (for peptides): appropriate Biotage Sfär Bio C18 depending on sample mass, 2 CVs 90% A/10% B; 10 CVs linear ramp up to 30% A/70% B; step gradient up to 100% B, 2 CVs 100% B.
Method B (for libraries): appropriate Biotage Sfär Bio C18 depending on sample mass, 3 CVs 90% A/10% B; step gradient up to 30% A/70% B, 3 CVs 30% A/70% B.
Method C(for stapled peptides): appropriate Biotage Sfär Bio C18 depending on sample mass, 3 CVs 100% A/0% B; step gradient up to 50% A/50% B, 3 CVs 10% A/90% B.

General procedure for split-and-pool combinatorial library synthesis: Coupling and Fmoc deprotection steps were performed according to the stoichiometries and procedures described above. Monosized Tentagel beads (30 µm, loading 0.26 mmol/g, variable scale) were loaded into a fritted syringe, swollen in DMF and then coupled to Fmoc-Rink Amide linker. For variable positions,

the resin was suspended in DMF divided in equal aliquots, coupled to the respective Fmoc amino acid and then pooled after Fmoc deprotection. Mmt deprotection, disulfide macrocyclization and cleavage were carried out using the above mentioned procedures. Library desalting and purification was carried out by flash chromatography according Method B. Small test libraries were stored in stock in 5% DMF/95% MQ + 0.1% FA water at -20°C. High-diversity libraries were stored in DMSO stocks at -20°C.

Small test library linearization and MS/MS analysis Stocks of cyclic test libraries were diluted to 100 fmol/member in 10mM TCEP.HCl in MQ + 0.1%FA and shaken for 15 min. at room temperature as previously described. A 100 µL sample of the reaction crude was then subjected to StageTip[3] purification (C18 material first prepared by washing with 100uL MeOH, followed by 100 µL ACN + 0.1%FA, 100 uL MQ + 0.1%FA, 100uL sample, 100 uL MQ + 0.1%FA and finally elution using 100 uL 70/30 ACN + 0.1%FA/MQ + 0.1%FA. Eluent was then flash frozen and lyophilized. Powders were resuspended in 100 µL MQ + 0.1%FA and 1 µL injected for tandem MS analysis. Acetylation of the N-terminus was selected as a variable modification and Mpa was input as Alanine mass variation.

Synthesis of a 16.25E6 membered library with design ZCXXXCK, where Z represent carboxylic acids and X amino acids: 2.2 g of 30 µm TentaGel resin (0.26 mmol/g, 0.74 mmol, 1.625 x 10^7 beads) was transferred to a 25 mL peptide synthesis vessel, swollen in DMF, and then washed with DMF (3x). Fmoc-Rink amide linker (5 eq) was dissolved in HATU solution (0.38 M in DMF) activated with DIPEA (1.86 mL, 10.7 mmol) immediately prior to coupling, and added to resin bed. Coupling was performed for 30 min; after this time, resin was washed with DMF (100 mL). Fmoc removal was carried out by treatment of resin with 20% piperidine in DMF (1 x 30 mL flow wash; 1 x 15 mL, 5 min batch treatments). Resin was then washed with DMF (150 mL). Coupling of Fmoc-Cys(Mmt)-OH and Fmoc-Lys(Boc)-OH was done in a single pooled fraction.

At this stage, resin was suspended in DMF (20 mL), and divided in 50 parts for amino acid coupling (in 50x5 mL fritted syringes) and in 130 parts for carboxylic acid coupling (in 130x2 mL fritted syringes). Couplings were performed according to the procedure described in "Peptide synthesis procedures". After coupling for 60 min, resins were washed with DMF and treated with 20% piperidine in DMF for Fmoc deprotection. After, the resin was poured back into a 25 mL synthesis vessel, and washed with DMF (100 mL) and thoroughly mixed. The split-and-pool procedure was repeated for each variable position of the library design. Mmt deprotection, disulfide macrocyclization, cleavage and purification were performed as described in "Peptide Synthesis procedures". Lyophilized powder (360 mg, approx). Library was then dissolved in DMSO to pre-make a stock concentration of 160 mM and stored at -20C.

Quality control for high-diversity libraries: 1 mg of dry linear library resin was suspended in 1000 µL MQ + 0.1% FA and homogenized using sonication at 60 °C overnight. Once well suspended, 10 µL (approximately 500 beads/peptide identities, approx.. 4 pmol/peptide) was aliquoted to a plastic 1.7 mL Eppendorf tube and centrifugated at 15000 rpm for 5 min. The supernatant was removed and 100 µL of cleavage cocktail (87.5% TFA, 5% water, 5% 1,2-ethanedithiol and 2% triisopropylsilane) was

added and incubated for 1h30min. The cocktail was then evaporated under a stream of nitrogen and peptides suspended in 100 µL MQ + 0.1% FA. This sample as then subjected to StageTip purification as described above, lyophilization and reconstitution in 10 µL MQ + 0.1% FA. 9 µL were subsequently injected for nLC-MS/MS analysis.

Statistical analysis of CycloSEL-16M quality control: A random sample of 1000 beads was drawn (2x 500beads) from the 16-million–member library to assess synthesis quality. Each bead was treated as a Bernoulli trial (correctly synthesized or defective), so the number of correctly synthesized beads follows a binomial distribution $X \sim \text{Binomial}(n, p)$, where p is the true synthesis success rate. The observed proportion of correct beads was $\hat{p} = 900/1000 = 0.90$. Using the standard Wilson score formula for a 95% confidence interval, the resulting interval is approximately 0.88–0.92. This indicates that the true synthesis success rate in the full library lies between 88% and 92% with 95% confidence, providing a statistically significant estimate of overall library quality. The sample size $n = 1000$ is sufficient for this purpose, and finite population effects are negligible ($n/N \approx 0.006\%$).

Affinity Selection: The affinity selection experiments were executed using a KingFisher™ Duo Prime Purification System. All operational protocols were programmed and managed via BindIt 4.1 Software. Selection assays targeting CAIX and WDR5 were conducted in duplicate. For each run, 150 pmol of biotinylated protein was immobilized onto 1 mg of Dynabeads MyOne Streptavidin T1. This immobilized target was then exposed to the library, which was applied at a concentration of 10 fmol per member. Unless otherwise specified, these procedures followed the standardized KingFisher protocol detailed in Supplementary Table S1.

Table S1: Automated ASMS protocol summary.

	General		Beginning of step		Mixing/Heating		End of step		
	Buffer	Volume (µL)	Release time (s)	Release speed	Mixing time (min)	Temp	Mixing speed	Collect count	Collect time (s)
Bead uptake		100	-	-	5	r.t.	Bottom mix	5	10
Bead washing (3x)	A	1000	30	Medium	3	r.t.	Medium	5	10
Protein incubation	B	100	30	Medium	60	10 ^{o}C	Medium	5	10
Biotin blocking (2x)	C	1000	30	Medium	10	r.t.	Medium	5	10
Bead washing	A	1000	30	Medium	3	r.t.	Medium	5	10
Library Incubation	E	1000	30	Medium	60	10 ^{o}C	Medium	5	10
Bead washing (5x)	F	1000	30	Medium	0.5	r.t.	Medium	5	10
Elution (2x)	G	100	30	Medium	3	r.t.	Medium	5	10

Table S2: ASMS buffer summary.

Buffer	CAIX	WDR5
A	10% FBS, 1x PBS, 0.02% Tween-20	20m mM Tris, 500 mM NaCl, pH 8, 10% FBS, 0.02% Tween-20
B	Biotinylated CAIX (1.5 μM) in **A**	Biotinylated WDR5 (1.5 μM) in **A**
C	10% FBS, 1x PBS, 0.02% Tween-20, 400 μM d-biotin	20m mM Tris, 500 mM NaCl, pH 8, 10% FBS, 0.02% Tween-20, 400 μM biotin
D	10% FBS, 1x PBS	20m mM Tris, 500 mM NaCl, pH 8, 10% FBS,
E	10 fmol/member library in buffer **D**	10 fmol/member library in buffer **D**
F	1x PBS	20m mM Tris, 500 mM NaCl, pH 8
G	6M Guanidine HCl, 200 mM phosphate buffer, pH 6.8, 10 mM TCEP HCl	6M Guanidine HCl, 200 mM phosphate buffer, pH 6.8, 10 mM TCEP HCl

Sample preparation after AS: Following the affinity selection, samples were desalted using StageTips. These were constructed using C18 material sourced from Merck Empore SPE 47 mm discs (66883-U) according to Rappsilber et al. The StageTips underwent a three-step pre-conditioning sequence, with each step consisting of a 3-minute centrifugation at 300 rpm: 100 μL MeOH 100 μL of 0.1% (v/v) FA in CAN and 100 μL of 0.1% (v/v) FA in MQ water. The samples were loaded onto the conditioned StageTips and rinsed with 100 μL of 0.1% (v/v) FA in MQ. To retrieve the compounds, 100 μL of an elution solvent (0.1% (v/v) FA in a 7:3 ACN:MQ mixture) was applied. The resulting eluates were lyophilized to dryness and then reconstituted in 10 μL of 0.1% (v/v) FA in UPLC-MS grade water. This 10 μL volume was transferred into an LC-MS vial, from which a 9 μL aliquot was injected into the LC-MS/MS system for analysis.

Biolayer interferometry (BLI): Biolayer interferometry (BLI) assays were performed in 96 well plates (GreinerBio-One, polypropylene, flat-bottom, chimney well) using an Octet R4 system (SATORIUS). Wells were filled with 200 μL with kinetic buffer, compound solution or protein solution, purified compounds. Proteins were dissolved in the appropriate kinetic buffer specific for each target (CAIX: 1× PBS, 0.02% Tween-20, 1 mg/mL BSA [0.1% w/v]; WDR5: 20 mM Tris, 500 mM NaCl, pH 8.0, 0.02% Tween-20, 1 mg/mL BSA [0.1% w/v]).

<u>Direct binding assay:</u> Purified biotinylated compounds were dissolved in appropriate kinetic buffer and immobilized onto the streptavidin biosensor for 60 s. Sensors were then dipped into kinetic buffer for 60 s, concentration gradient of protein solution for 300s and into kinetic buffer for 300s. Measurements were carried out at 20 °C.

<u>Competition assay:</u> Purified biotinylated competitor were dissolved in appropriate kinetic buffer and immobilized onto the streptavidin biosensors at 100nM for 60s. Sensors were then dipped into kinetic buffer for 60 s and concentration gradient of macrocyclic hit in constant concentration protein solution for 600s. Measurements were carried out at 20 °C.

Single-molecule fluorescence sample preparation adapted from Filius et al.[4]: Macrocycle 10 was labelled at the lysine sidechain using 2 eq. of Sulfo-Cy3-NHS (Lumiprobe, 21320) in DMF with excess of DIPEA overnight at room temperature. N-terminal biotinylation and fluorescent labeling of WDR5 were performed as described previously. In brief, 10 μM WDR5 was incubated with a 200-fold molar excess of 2PCA-DBCO for 24 h at 37 °C with shaking. The following day, unreacted 2PCA-DBCO was removed using Zeba™ Spin Desalting Columns (7 kDa MWCO; ThermoFisher, 89882) following the manufacturer's instructions. The protein was then labeled with a 2-fold molar excess of Azide-Cy5-biotin (Click Chemistry Tools, CCT1232) and incubated overnight at room temperature (23 ± 1 °C). Excess Cy5-biotin-azide was removed the next day using Zeba™ Spin Desalting Columns equilibrated in 1× PBS.

Single-molecule data acquisition and analysis adapted from Filius et al.[5], Chandradoss et [6]. and Kim et al.[7]: Single-molecule flow cells were prepared according to previously described methods by first assembling a microfluidic chamber and incubating the slides with 20 μL of 0.1 mg/mL neutravidin (Thermofisher: 31000) for 2 minutes, followed by a 100 μL 1x PBS wash to remove any excess. Next, 50 μL of 75 pM Cy5-biotin-azide-labelled WDR5 was introduced into the chamber and allowed to incubate for 2 minutes before unbound protein was cleared with a 200 μL PBS wash. Imaging was then initiated by injecting 50 μL of 100 pM Cy3-labeled peptides suspended in an imaging buffer containing 2.5 mM PCA (Sigma: 37580), 0.155 U/μL PCD (OYC Europe: 46852004), and 1 mM Trolox (Sigma: 238813) in 1x PBS. Fluorescence data were recorded at a 0.1-s exposure time using a green laser to excite the Cy3 donor fluorophores, and the resulting movies were processed using a custom Python script that paired intensity hotspots from the acceptor and donor channels to extract time traces. A two-state K-means clustering algorithm was then applied to the combined donor and acceptor fluorescence intensities of individual molecules to define an intensity threshold for distinguishing high- and low-intensity segments. For the final kinetic analysis, only high-intensity segments that persisted for more than three consecutive frames were selected, with the specific kinetic rates determined by the following equations. The dissociation rate (koff) is calculated as follows:

$$k_{off} = \frac{1}{\tau_b} \quad (1)$$

Where τ_b is the dwell-time of the macrocycle binding events. The association rate (kon) is calculated as follows:

$$k_{on} = \frac{1}{\tau_{ub}} \cdot \mathrm{c} \quad (2)$$

Where τ_{ub} is the time in between macrocycle binding events and c is the macrocycle concentration. The dissociation constant KD is calculated as follows:

$$K_D = \frac{k_{off}}{k_{on}} \quad (3)$$

Serum stability assay: A 25% human serum solution was prepared in 1xPBS (pH = 7.4). Macrocycle stocks were diluted to a final concentration of 25 μM in the 25% serum solution. The mixture was vortexed immediately after protein addition. At different time points, a sample was retrieved and the degradation reaction was quenched by the addition of 20% trichloroacetic acid (w/w) in 1x PBS supplemented with Fmoc-Lys(Ac)-OH as internal standard (IS) to a final concentration of 12.5 μM peptide and 12.5 μM peptide IS. After incubating on ice for 1h, the samples were centrifuged at 10,000 rpms for 5 min. The supernatant was then injected into the UPLC-HRMS and the AUC of both peptide and IS by analyzing the XIC curve for each exact mass value. For each run and timepoint, the peptide AUC was then normalized to the IS AUC. The relative stability values were then plotted using GraphPad Prism 10.1.0 and a one-phase decay non-linear fit curve. Half-life times ($t_{1/2}$) were calculated at the intersection of the curve with y=50%.

Parallel artificial membrane permeability assay (PAMPA):

The membrane permeability of cyclic peptides was assessed using a pre-coated PAMPA plate system (Corning BioCoat, 353015) consisting of a 96-well donor microwell plate and a 96-well acceptor insert featuring a PVDF membrane coated with phospholipid trilayers. Permeability measurements were conducted by monitoring the transition from the bottom donor wells to the top acceptor wells. Solutions of cyclic peptides or reference compounds were prepared at 100 μM in 1xPBS (pH 7.4) with 1% DMSO, and 300 μl of these solutions were added to the donor wells, while the acceptor wells were filled with 200 μl of the same buffer-DMSO mixture. The insert was then stacked onto the donor plate and sealed with a lid to prevent evaporation during an 18-hour incubation at room temperature. Following incubation, UPLC–HRMS was used to analyze the acceptor solutions and determine the concentration of permeated peptides. To establish a reference for maximum permeability, 3:2 dilutions of the initial 100 μM stocks were prepared in 1xPBS (pH 7.4) with 1% DMSO, representing the theoretical equilibrium between the donor and acceptor chambers. These equilibrium samples were also analyzed via UPLC–HRMS, and the resulting data were used to calculate the permeability percentage and apparent permeability according to the equations provided below.

$$Permeability\ (\%) = \frac{[Peptide_{Acceptor}]}{[Peptide_{Equillibrium}]}\ (4)$$

where [PeptideAcceptor] represents the AUC measured on the UPLC–HRMS for the acceptor wells and [PeptideEquillibrium] represents the AUC measured for the theoretical equilibrium. AUCs were determined based on the XIC curve for each exact mass value.

Apparent permeability:

$$P_{app} = \frac{V_D \times V_A}{(V_D + V_A) \times A \times t} \times -ln\left(1 - \frac{[Peptide_{Acceptor}]}{[Peptide_{Equillibrium}]}\right)\ (5)$$

where VD and VA represent, respectively, volume of donor (0.3mL) and acceptor (0.2mL), A represents membrane area (0.3cm2) and t represents incubation time (18h, 64.800sec).

Physico-chemical characterization of macrocycle 25 adapted from Garcia et al[9].:

Chromatographic Environments: The chromatographic experiments were conducted using isocratic mobile phases composed of 20 mM ammonium acetate (pH 7.0) and varying percentages of acetonitrile, depending on the specific descriptor being measured. Macrocycle 25 was prepared in buffer/acetonitrile mixtures at concentrations between 50 and 100 µg/mL. For each analysis, a 10 µL injection volume was used at an isocratic flow rate of 1 mL/min, with the column oven maintained at a temperature of 30 °C. All chromatographic measurements were performed in duplicate, following the specific conditions required for each individual descriptor.

PLRP-S System: The retention time (RT) for each compound within the dataset was measured across six distinct mobile phase conditions, ranging from 50% to 100% MeCN, utilizing a PLRP-S column. Following these measurements, the capacity factor (log k' PLRP-S) was determined for each entry using the equation provided below.

$$\log k' \,(\%MeCN) = \frac{\log[t_R - t_0]}{t_0} \quad (6)$$

Where t0 is the dead time, and plotted it at each mobile phase composition (% MeCN).

Chamelogk: To quantify chameleonicity, a linear trend was established using mobile phase conditions that exhibited linear behavior for bRo5 compounds, specifically at 50%, 60%, and 70% MeCN. Furthermore, 95% MeCN was identified as the condition yielding the most significant change in capacity factor and was utilized for comparative analysis and the calculation of Chamelogk. Gold standards for these measurements included acetone, caffeine, and phenol, as well as a mixture containing uracil, acetophenone, and toluene.

XBridge System: To determine BRlogD, samples were injected into an X-Bridge column using a mobile phase primarily composed of 60% MeCN. Both the retention times and the dead time (t0), identified via baseline interference, were recorded to calculate the capacity factor log k'60 by adapting the standard log k' equation. Finally, the BRlogD value was derived using the specific formula provided below.

$$BRlogD = 3.31 \times \log k'_{60} + 2.79 \quad (7)$$

In this case, BRlogD required the measurement of acetone, caffeine, ibuprofen, lidocaine, phenol, and a mixture of uracile, acetophenone, and toluene as gold standards.

IAM (Immobilized Artificial Membrane) System: To determine Log kWIAM, samples were injected into an IAM column across a range of mobile phase conditions from 10% to 50% MeCN. Retention times were recorded for each condition to calculate the capacity factor by adapting the log k' equation, utilizing the retention time of citric acid as the dead time t0. In addition, a daily assessment of five standards (caffeine, carbamazepine, ketoprofen, theobromine, and toluene) was performed. The final log kWIAM value for each compound was derived by extrapolating the capacity factors from the five mobile phase points to a purely aqueous environment (100% buffer/0% MeCN), while Δ log kWIAM followed the definition previously established by Grumetto et al.:

$$\Delta \log k_W^{IAM} = experimental \log k_W^{IAM} - \text{clog}\, k_W^{IAM} \text{ (8)}$$

with clog kWIAM being the log kWIAM value for nonpolar and neutral compounds with PSA = 0. Moreover, clog kWIAM was correlated with the log P value (octanol/water) and afterward with the chromatographic descriptor BRlogD using equation below:

$$\text{clog}\, k_W^{IAM} = BRlogD \times 0.92 - 1.03 \text{ (9)}$$

3.1.4 Protein expression, purification and quality control

WDR5 Expression and Purification: A truncated human WDR5 construct encoding amino acids 22–334(GSSHHHHHHSSGLVPRGSHMMSATQSKPTPVKPNYALKFTLAGHTKAVSSVKFSPNGEWLASSSADK LIKIWGAYDGKFEKTISGHKLGISDVAWSSDSNLLVSASDDKTLKIWDVSSGKCLKTLKGHSNYVFCCNFNP QSNLIVSGSFDESVRIWDVKTGKCLKTLPAHSDPVSAVHFNRDGSLIVSSSYDGLCRIWDTASGQCLKTLIDD DNPPVSFVKFSPNGKYILAATLDNTLKLWDYSKGKCLKTYTGHKNEKYCIFANFSVTGGKWIVSGSEDNLVYI WNLQTKEIVQKLQGHTDVVISTACHPTENIIASAALENDKTIKLWKSDCAE) was synthesized by GenScript and subcloned into a pET-15b expression vector containing an N-terminal 6×His-SUMO tag using the NdeI and BlpI restriction sites. The WDR5 plasmid was then transformed into E. coli BL21 (DE3) cells. The cells were cultured in Luria–Bertani medium at 37 °C. When the optical density at 600 nm (OD600) reached 0.8, the temperature was lowered to 25 °C. Protein expression was induced by adding 1 mM isopropyl-β-D-thiogalactoside (IPTG), and the incubation continued for 16 hours at this temperature. The cells were harvested by centrifugation, resuspended in lysis buffer (20 mM Tris-HCl, 500 mM NaCl, pH 8.0), and then lysed using a homogenizer (FPG12800). The lysate was cleared by centrifugation, and the supernatant was collected. The protein was then bound to a nickel affinity column (HisTrap™, Cytiva) using an ÄKTA system and eluted using an imidazole gradient. The purified protein was verified by SDS PAGE and concentrated using a 10K molecular weight cut-off centrifugal concentrator. The concentration was determined using a NanoDrop spectrophotometer.

WDR5 biotinylation: WDR5 was biotinylated using the EZ-Link™ NHS-PEG4-Biotin reagent (Promega) according to the manufacturer's protocol. Briefly, the purified WDR5 protein was diluted to a concentration of approximately 1 mg/mL. The NHS-PEG4-biotin reagent was then added to the protein solution to a final concentration of 10–20 μM. The reaction was incubated for 1 hour at room temperature with gentle agitation to facilitate the biotinylation process. After the incubation, unreacted biotin reagent was removed using a 10K molecular weight cut-off centrifugal concentrator. The biotinylated WDR5 protein was stored at −80 °C for future use. The degree of biotinylation was confirmed using BioLayer Interferometry to verify biotin incorporation.

Fluorescence polarization assay: The availability of the binding pocket in the biotinylated WDR5 protein was confirmed by fluorescence polarization (FP) assays. Measurements were performed in buffer containing 20 mM Tris-HCl, 500 mM NaCl, pH 8.0, using 10 nM Ac-ARTEVY-βA-FITC as the fluorescent tracer and 5 μM WDR5. Assays were carried out in black 384-well microplates (Corning®, CLS3573) in a total volume of 60 μL per well by mixing 30 μL protein solution with 30 μL tracer

solution. The mixtures were gently mixed, briefly centrifuged, and incubated for 10 min at room temperature before measurement. Fluorescence polarization was recorded on a CLARIOstar plate reader (BMG Labtech) with an excitation wavelength of 482 nm and an emission wavelength of 520 nm.

Polarization (P) was calculated according to the following equation:

$$P = \frac{F_{\parallel} - F_{\perp}}{F_{\parallel} + F_{\perp}}$$

where F_∥and F_⊥are the fluorescence emission intensities parallel and perpendicular to the excitation light plane, respectively.

3.1.5 Cell viability

5000 HL60 (DSMZ ACC3), 3000 MV411 (DSMZ ACC554) and 7000 MOLM13 (DSMZ ACC554) cells were plated into an opaque colored 96 wells plate (Greiner Bio-one cat no 655083). Cells were plated in triplicates in presence of WDR5 compounds, in a two-fold dilution series from 50 µM to 0.39 µM. WDR5 compounds were diluted in medium containing 2.5% DMSO. This results into a final DMSO concentration of 0.5%. Cell viability was measured at 72h using CellTiter-Glo® assay (Promega, cat no G7570). 100 µl CellTiter-Glo reagent was added to each well and plates were shaken 5 minutes at 500 rpm. Luminescent signal was stabilized by incubating the plates 10 minutes at RT. Luminescence was measured on the Victor X3 plate reader (Perkin Elmer).

3.1.6 Software and workflows for data analysis

Summary on peptide analysis workflow

The analysis of the synthetic peptide library was performed using an automated workflow integrating Data-Dependent Acquisition (DDA) and the PEAKS computational suite. Upon injection into the nanoLC-MS/MS, peptides are first separated by hydrophobicity before entering the mass spectrometer. The instrument operates in DDA mode, which acts as an intelligent selector: it performs a high-resolution survey scan (MS1) to detect intact peptide masses, then immediately selects the most intense ions for fragmentation (MS/MS). To ensure high data quality and depth, the DDA settings utilize charge state screening to prioritize multi-charged peptides (e.g., z = +2 to +5) and intensity thresholds to filter out background noise. Furthermore, dynamic exclusion is applied to prevent the repetitive fragmentation of highly abundant peptides, allowing the instrument to "look deeper" into the library and capture lower-abundance sequences.

Once the raw data is acquired, PEAKS software initiates Feature Detection, a process that groups raw signals into distinct biological entities. Instead of analyzing isolated peaks, the software

identifies isotopic clusters and aligns signals across their chromatographic elution time. It then performs deconvolution, mathematically collapsing multiple charge states into a single monoisotopic neutral mass. This ensures the de novo algorithm begins with a highly accurate precursor mass.

The core of the analysis is the de novo sequencing algorithm, which reconstructs the peptide sequence directly from the MS/MS fragment ions (primarily b and y series). By calculating the mass gaps between fragment peaks, PEAKS identifies the specific amino acids that bridge the sequence. Unlike database searching, this method does not require a reference proteome, making it ideal for verifying synthetic constructs. Each resulting sequence is assigned an Average Local Confidence (ALC) score, representing the mean confidence level across all amino acid residues in the chain. This ALC score serves as a rigorous statistical filter; an ALC of > 70 or 80% typically denotes a high-fidelity identification where the spectral evidence strongly supports the proposed primary structure.

PEAKS11: De novo peptide sequencing was performed by processing .raw files obtained from Orbitrap analysis using PEAKS Studio (version 11) from Bioinformatics Solutions Inc. (ON, Canada). HCD scans were merged within a 0.2 minute and 0.02 Da window, mass precursor correction was used, and primary mass filtration was employed as appropriate. Auto *de novo* sequencing was performed using a 15 ppm precursor mass error and 0.02 Da fragment mass error, and with the modifications present in the table below correlated to the exact mass of alanine. Modifications were inserted either as fixed or variable. (+XX.XX) refers to the correlation between the unnatural building block and alanine following the formula XX.XX = (Exact mass unnatural building block) - (Exact mass alanine) or as fixed modification (for the case of Mpa and Lys). The maximum number of PTMs per sequence was defined as to be the same as the number of variable positions in the library scaffold. 10 candidate sequences were obtained for each preprocessed scan. Post-*de novo* data filtering was carried out using Knime and Python workflows aimed at eliminating sequences with ALC below 80% as well as ensure compliance with library design (peptide length and scaffold). Recalls are calculated using (number of found sequences)/(theoretical diversity of the library)*100 (%).

Knime Data Analytics Platform: KNIME 4.7.1 software was used for library enumeration, molecular property calculations and de novo sequencing data filtering. The following extensions were installed RDkit Nodes version 4.7., CDK version 1.5.6 and ChemAxon version 4.7.0.

<u>Library enumeration:</u> Virtual generation of macrocyclic libraries were based on the following general protocol: Building blocks were supplied as SMILES in Tables and reactions were supplied as SMARTS. Library enumeration workflow: 1) C-terminal amidation of Fmoc-Lys(Boc)-OH via RDKit Chemical Transformation; 2) Fmoc removal via RDKit One Component Reaction; 3) amide coupling of Fmoc-Cys(Mmt)-OH via RDKit Two Component Reaction; 4) Fmoc removal via RDKit One Component Reaction; 5) The elongation of the molecule was carried with consecutive cycles of Two and One Component reactions for amide coupling and Fmoc removal using all possible building blocks for each coupling position via matrix expansion and uniquifying products; 6) For library CycloSEL-16M, carboxylic acid capping in the exocyclic position was achieved with Two Component Reaction with matrix expansion. Side chain deprotection was carried out by providing deprotection SMARTS to a

Chunk Loop enclosing a Chemical Transformation in chunks of 100 000 rows per cycle; 7) Macrocyclization was achieved by providing disulfide bridge formation SMARTS to a Chunk Loop enclosing a Chemical Transformation in chunks of 100 000 rows per cycle; 8) Molecular properties were calculated by feeding the enumerated library to a RDKit Descriptor Calculation node including sLogP, TPSA, ExactMW and NumHBD calculation features.

Table S3: List of transformations (reactions and deprotections) used in Knime workflows.

Step	Reaction SMARTS
Fmoc-deprotection	[#7:17]-[#6:16](=[O:18])-[#8:15]-[#6:14]-[#6:1]-1-[c:5]2[c:9][c:8][c:7][c:6][c:4]2-[c:3]2[c:10][c:11][c:12][c:13][c:2]-12>>[#7:17].[O:18]=[#6:16]-[#8:15]-[#6:14]-[#6:1]-1-[c:2]2[c:13][c:12][c:11][c:10][c:3]2-[c:4]2[c:6][c:7][c:8][c:9][c:5]-12
Amide bond formation	[#7;A;X3;H2,H1;!$(NC=O);!$(NC=CC=O);!$(NC=S);!$(NC=N)!$(N-S):3].[#8;A;X1H0-,X2H1][#6;A;X3:2]=[O:1]>>[#7;A;X3:3][#6;X3:2]=[O;X1:1]

Protecting group	Deprotection SMARTS
Fmoc	[#7:17]-[#6:16](=[O:18])-[#8:15]-[#6:14]-[#6:1]-1-[c:5]2[c:9][c:8][c:7][c:6][c:4]2-[c:3]2[c:10][c:11][c:12][c:13][c:2]-12>>[#7:17].[O:18]=[#6:16]-[#8:15]-[#6:14]-[#6:1]-1-[c:2]2[c:13][c:12][c:11][c:10][c:3]2-[c:4]2[c:6][c:7][c:8][c:9][c:5]-12
Trt	[N:13][C:14]([c:12]1[c:1][c:2][c:3][c:4][c:5]1)([c:11]1[c:6][c:7][c:8][c:9][c:10]1)[c:15]1[c:16][c:17][c:18][c:19][c:20]1>>[N;A;X3,H2:13]
Trt	[n:13][C:14]([c:12]1[c:1][c:2][c:3][c:4][c:5]1)([c:11]1[c:6][c:7][c:8][c:9][c:10]1)[c:15]1[c:16][c:17][c:18][c:19][c:20]1>>[n;A;X3,H1:13]
Boc	[N:3]-[#6:4](=[O:10])-[#8:5][C:6]([#6:7])([#6:8])[#6:9]>>[N;A;X3:3]
Boc	[n:3]-[#6:4](=[O:10])-[#8:5][C:6]([#6:7])([#6:8])[#6:9]>>[n;A;X3;H1:3]
OtBu	[#6;H3:5][C:2]([#6;H3:4])([#6;H3:3])[#8:1]>>[#8:1]
NtBu	[#6;H3:5][C:2]([#6;H3:4])([#6;H3:3])[#7:1]>>[#7:1]
Pbf	[#6:11]-[#8:10]-[c:9]1[c:8][c:6](-[#6:7])[c:5]([c:14](-[#6:15])[c:12]1-[#6:13])[S:2]([#7:1])(=[O:3])=[O:4]>>[#7:1]
CA70	[#6:1][C:2]1([#6:10])[#8:3]-[#6@@H:4]-2-[#6:9]-[#8:8]-[#6:7]-[#6@@H:5]-2-[#8:6]1>>[#8:6]-[#6@H:5]-1-[#6:7]-[#8:8]-[#6:9]-[#6@H:4]-1-[#8:3]
CA8	[#6:8]-1-[#6:5][C:6]2([#6:7]-1)[#8:1]-[#6:2]-[#6:3]-[#8:4]2>>O=[#6:6]-1-[#6:7]-[#6:8]-[#6:5]-1
CA21	[O:1]=[#6:2]-[#6:3]-1-[#6:4]-[#6:5][C:6]2([#6:7]-[#6:8]-1)[#8:9]-[#6:10]-[#6:11]-[#8:12]2>>[O:1]=[#6:2]-[#6:3]-1-[#6:8]-[#6:7]-[#6:6](=[O:12])-[#6:5]-[#6:4]-1
CA44	[#7:1]-[#6:2](=[O:17])-[#8:3]-[#6:4](-[c:5]1[c:6][c:7][c:8][c:9][c:10]1)-[c:11]1[c:12][c:13][c:14][c:15][c:16]1>>[#7:1]

<u>De novo sequencing data filtering</u>: Output .csv files from PEAKS 11 were converted to .xlsx files and fed to an Excel reader node. Several column, row and cell splitter and row sorter nodes were used to

filter for rows with the correct peptide length, average level of confidence (ALC) over 80%. At this point, each peptide sequence is represented by a sequence of one-letter codes for natural amino acids and A(+xx.xx) codes for unnatural amino acids. Each sequence has been filtered for ALC, size and has appended its retention time (RT), charge (z), m/z and ALC. Before library design filtering, the list of sequences are fed to a Python script that translates the one letter and A(+xx.xx) codes to in-house defined barcodes for each building block. This translated list is then fed back to Knime and using Reference Row Filter nodes, the peptide positions are compared to a table containing the library design, retaining only peptides with the correct design. Duplicates are removed using a Duplicate Row Filter node.

ThermoFisher Freestyle: Output from sequencing data filtering is then manually analysed using Freestyle. For each remaining entry, we manually query for the detected m/z value by XIC. Hits are confirmed by having a clear enrichment peak compared to control and by validating the isotope and MS2 fingerprint.

Pharmacophore-based modelling: Using published ligand-bound x-ray crystal structure of WDR5 (PDB: 6UCS)[10] in pharmacophore modelling tool 'Pharmit'[11], three pharmacophore interactions were defined in the bound ligand. These interactions served to constrain the macrocycle to form comparable interactions with the published ligand, while no constraint would be placed on orientation or position of the additional building blocks present in the hit. Two H-bond donor interactions were defined deep in the S2 pocket with an additional hydrophobic interaction defined at the entrance of S2. Taken together, these nodes mimicked the functional composition of an arginine residue. Next, low energy poses for the macrocycle hit were generated in Pharmit, and conformers were aligned with the prepared pharmacophore model. >150 conformations were found as matches. An energy minimization step was performed, and an energy score filter (<-6) and maximum mRMSD (<4.0) was set to eliminate low-quality poses.

4. **Building block dictionary: AAs and CAs and exact mass difference for *de novo* sequencing**

Fixed Amidation at C-terminal Lys: -0.98

Variable building block modifications:

**Carbocylic acid (CA)**	**SMILES**	**Exact MW[CA]-Exact MW[Ala]**
CA1	O=C(O)c1ccoc1	22.968
CA2	O=C(O)c1cocn1	23.964
CA3	Cc1ocnc1C(=O)O	37.979
CA4	O=C(O)Cc1ccsc1	52.961
CA5	O=C(O)COc1ccc2c(c1)CCC2	103.031
CA6	O=C(O)C1CCOc2ccccc2O1	105.010
CA7	O=C(O)Cc1ccco1	36.984
CA8	O=C(O)C1CC2(C1)OCCO2	24.984
CA9	O=C(O)c1ccc2nonc2c1	74.975
CA10	O=C(O)C1CCc2ccccc21	73.020
CA11	COC(C)C(=O)O	15.000
CA12	CO[C@H]1CC[C@H](C(=O)O)CC1	69.047
CA13	Cc1cc(C(=O)O)cc(Cl)n1	81.961
CA14	O=C(O)c1ccc2cnccc2n1	84.995
CA15	O=C(O)CC1Cc2ccccc2C1	87.036
CA16	CCCCCC(C)C(=O)O	55.067
CA17	O=C(O)Cc1ccccn1	48.000
CA18	O=C(O)Cc1ccc2c(c1)CCO2	89.015
CA19	O=C(O)c1cncnc1	34.980
CA20	O=C(O)C1Cc2ccccc2O1	75.000
CA21	O=C(O)C1CCC2(CC1)OCCO2	53.015
CA22	CC1CC(C(=O)O)C1	25.020
CA23	O=C(O)Cc1ccc2ncccc2c1	98.016
CA24	O=C(O)c1cc2sccc2[nH]1	77.956
CA25	CC(C)(C)c1ccc(CC(=O)O)cc1	103.067
CA26	COc1cc(CCC(=O)O)on1	82.005
CA27	O=C(O)COc1ccc2ccccc2c1	113.015
CA28	CC(C)CCCC(=O)O	41.052
CA29	CCCCC[C@H]1CC[C@H](C(=O)O)CC1	109.114
CA30	O=C(O)c1cn2ccsc2n1	78.952
CA31	O=C(O)/C=C/c1nc2ccccc2s1	115.972
CA32	O=C(O)[C@@H]1CCCO1	27.000
CA33	C=CCC(=O)O	-3.011

CA34	CCCCC(C)CCC(=O)O	69.083
CA35	C=C1CC(C(=O)O)C1	23.005
CA36	O=C(O)c1ncoc1-c1ccccc1	99.995
CA37	Cc1cc(C)n2nc(C(=O)O)cc2n1	102.022
CA38	O=C(O)C1CC12CCC2	37.020
CA39	O=C(O)C1Cc2ccccc21	59.005
CA40	C#CCC(=O)O	-5.027
CA41	CC(C)(C)OC(=O)Nc1nc2c(ncn2CC(=O)O)c(=O)[nH]1	120.007
CA42	Cc1cn(CC(=O)O)c(=O)[nH]c1=O	95.001
CA43	O=C(O)c1ccc(Cl)s1	72.907
CA44	O=C(O)Cn1ccc(NC(=O)OC(c2ccccc2)c2ccccc2)nc1=O	80.001
CA45	CC(=O)N/C(=C\c1ccccc1)C(=O)O	116.026
CA46	CC(=O)Oc1ccc(C(=O)O)cc1	90.995
CA47	O=C(O)CCC(=O)c1ccc(Br)cc1	166.926
CA48	COc1cc(C(=O)O)cc(OC)c1Br	170.921
CA49	O=C(O)CCC(=O)c1ccc(Cl)cc1	122.976
CA50	O=C(O)c1ccc(F)c(Cl)c1	84.941
CA51	O=C(O)c1c(Cl)cccc1Cl	100.911
CA52	O=C(O)Cc1ccc(Cl)c(Cl)c1	114.927
CA53	O=C(O)c1cccc(F)c1	50.980
CA54	O=C(O)Cc1ccc(F)cc1	64.995
CA55	NC(N)=Nc1ccc(C(=O)O)cc1	90.022
CA56	O=C(O)c1cccc(O)c1	48.984
CA57	O=C(O)c1ccc(I)cc1	158.886
CA58	O=C(O)Cc1ccccc1I	172.901
CA59	O=C(O)c1cc(S(=O)(=O)O)ccc1O	128.941
CA60	COc1cc(C(=O)O)cc(OC)c1O	109.005
CA61	O=C(O)CNC(=O)c1cccc(C(F)(F)F)c1	157.998
CA62	COc1cc(C(=O)O)cc(OC)c1OC	123.021
CA63	O=C(O)CCCCBr	90.931
CA64	CC(C)c1ccc(C(=O)O)cc1	75.036
CA65	O=C(O)c1ccc[nH]1	21.984
CA66	O=C(O)c1ccc(F)cc1Br	128.890
CA67	O=C(O)C1CCC1	11.005
CA68	COCC(=O)O	0.984
CA69	O=C(O)c1cc(Br)cc(Br)c1	188.810
CA70	CC1(C)O[C@@H]2[C@H](O1)[C@@H](C(=O)O)O[C@H]2n1cnc2c(=O)[nH]cnc21	193.012
CA71	CCC(=O)O	-15.011
CA72	CCCCC(=O)O	13.020
CA73	COc1cccc(C(=O)O)c1	63.000
CA74	NS(=O)(=O)c1ccc(C(=O)O)cc1	111.962
CA75	CCCC(=O)O	-0.995

CA76	N#Cc1ccc(C(=O)O)cc1	57.984
CA77	O=C(O)C(=O)c1ccccc1	60.984
CA78	CC(=O)N1CCC(C(=O)O)CC1	82.042
CA79	O=C(O)c1cc(Br)ccc1Cl	144.861
CA80	CSc1ccc(C(=O)O)cc1	78.977
CA81	O=C(O)CCc1ccc(B(O)O)cc1	105.027
CA82	O=C(O)c1ccc(Br)cc1	110.900
CA83	Cc1cccc(Cl)c1C(=O)O	80.966
CA84	CC(C)(C)c1ccc(C(=O)O)cc1	89.052
CA85	O=C(O)Cc1ccccc1Br	124.915
CA86	O=C(O)CCc1ccc(Br)cc1	138.931
CA87	N#CCC(=O)O	-4.031
CA88	COc1ccc(C(=O)O)c(OC)c1	93.010
CA89	CN(C)c1cccc(C(=O)O)c1	76.031
CA90	COc1cc(C)c(C(=O)O)cc1OC	107.026
CA91	O=C(O)C(c1ccccc1)c1ccccc1	123.036
CA92	CCc1ccc(C(=O)O)cc1	61.020
CA93	CCCCCC(=O)O	27.036
CA94	CC(C)Oc1ccc(C(=O)O)cc1	91.031
CA95	COc1cccc(CC(=O)O)c1	77.015
CA96	O=C(O)/C=C/c1ccc2c(c1)OCO2	102.995
CA97	O=C(O)c1cccnc1	33.984
CA98	O=C(O)Cc1ccc(Oc2ccccc2)cc1	139.031
CA99	O=C(O)c1ccccc1-c1ccccc1	109.020
CA100	O=C(O)c1nc(O)nc(O)n1	67.965
CA101	O=C(O)c1ccc2c(c1)OCO2	76.979
CA102	O=C(O)Cc1ccc2ccccc2c1	97.020
CA103	O=C(O)c1ccccc1C(F)(F)F	100.976
CA104	O=C(O)Cc1cccc(C(F)(F)F)c1	114.992
CA105	O=C(O)CCc1cccnc1	62.016
CA106	O=C(O)C1CCCCCC1	53.052
CA107	CC(=O)O	-29.027
CA108	O=C(O)C(C(F)(F)F)C(F)(F)F	106.948
CA109	CC(=O)C(=O)O	-1.032
CA110	O=C(O)c1cc2ccccc2[nH]1	72.000
CA111	O=C(O)c1ccccc1	32.989
CA112	C#Cc1cccc(C(=O)O)c1	56.989
CA113	CN(C)Cc1ccc(C(=O)O)cc1	90.047
CA114	O=C(O)c1cnc2ccccc2c1	84.000
CA115	O=C(O)c1ccc(Cl)cc1	66.950
CA116	COc1cc(CC(=O)O)cc(OC)c1OC	137.036
CA117	O=C(O)CCCCl	32.966
CA118	O=C(O)c1cc(O)ccn1	49.979

CA119	Cn1c(C(=O)O)cc2ccccc21	86.016
CA120	O=C(O)Cc1ccccc1	47.005
CA121	CCCCCCCCCCCC(=O)O	111.130
CA122	O=C(O)C1CCCCC1	39.036
CA123	O=C(O)c1cc(=O)[nH]c(=O)[nH]1	66.969
CA124	O=C(O)c1cc(-c2ccccc2)nc2ccccc12	160.031
CA125	C#CCCC(=O)O	8.989
CA126	CS(=O)(=O)c1ccc(C(=O)O)cc1	110.967
CA127	COc1cc(C(=O)O)ccc1O	78.995
CA128	O=C(O)C1CCCS1	42.977
CA129	C#CC(=O)O	-19.042
CA130	CN(C)CC(=O)O	14.016
CA131	FC1=C(F)C=C(F)C(CC(O)=O)=C1	---
CA132	FC1=C(C=C(C=C1)C(O)=O)C(F)(F)F	---
CA133	FC(F)(F)C1=CC=CC(CC(O)=O)=C1	---
CA134	FC1=CC(SC(C(O)=O)=C2Cl)=C2C=C1	---
Mpa	OC(CCS)=O	16.961

**Amino acid (AA)**	**SMILES**	**Exact MW[AA]-Exact MW[Ala]**
AA1	CC1(C)Oc2ccc(C[C@H](NC(=O)OCC3c4ccccc4-c4ccccc34)C(O)=O)cc2O1	108.021
AA2	CN(C[C@H]1CC[C@@H](CC1)C(O)=O)C(=O)OCC1c2ccccc2-c2ccccc12	82.078
AA3	OC(=O)[C@@H](CCC(=O)NC1c2ccccc2Oc2ccccc12)NC(=O)OCC1c2ccccc2-c2ccccc12	237.079
AA4	OC(=O)[C@@H](Cc1cc(Br)c(O)c(Br)c1)NC(=O)OCC1c2ccccc2-c2ccccc12	247.847
AA5	OC(=O)CN1CN(c2ccccc2)C2(CCN(CC2)C(=O)OCC2c3ccccc3-c3ccccc23)C1=O	200.095
AA6	OC(=O)C1(CCN(Cc2ccccc2)CC1)NC(=O)OCC1c2ccccc2-c2ccccc12	145.089
AA7	OC(=O)[C@H](Cc1ccc(Cl)c(F)c1)NC(=O)OCC1c2ccccc2-c2ccccc12	127.983
AA8	OC(=O)[C@@H](Cc1ccccn1)NC(=O)OCC1c2ccccc2-c2ccccc12	77.027
AA9	OC(=O)C[C@H](Cc1ccc(F)c(F)c1)NC(=O)OCC1c2ccccc2-c2ccccc12	126.028
AA10	OC(=O)[C@@H]1Cc2ccccc2N1C(=O)OCC1c2ccccc2-c2ccccc12	74.016
AA11	OC(=O)c1cc2ccccc2cc1NC(=O)OCC1c2ccccc2-c2ccccc12	98.016

AA12	OC(=O)C1(CCC(F)(F)CC1)NC(=O)OCC1c2ccccc2-c2ccccc12	90.028
AA13	OC(=O)[C@@H]1CCCN1C1CCN(CC1)C(=O)OCC1c2ccccc2-c2ccccc12	109.089
AA14	OC(=O)[C@H]1CC[C@H](CNC(=O)OCC2c3ccccc3-c3ccccc23)CC1	68.063
AA15	OC(=O)COc1ccc2C(NC(=O)OCC3c4ccccc4-c4ccccc34)c3ccccc3CCc2c1	194.073
AA16	OC(=O)COC[C@H]1CCCN1C(=O)OCC1c2ccccc2-c2ccccc12	70.042
AA17	C[C@H](N(C)C(=O)OCC1c2ccccc2-c2ccccc12)C(O)=O	14.016
AA18	OC(=O)[C@H](Cc1ccc(O)c(Cl)c1)NC(=O)OCC1c2ccccc2-c2ccccc12	125.987
AA19	OC(=O)[C@H](Cc1c[nH]c2ccc(O)cc12)NC(=O)OCC1c2ccccc2-c2ccccc12	131.037
AA20	OC(=O)[C@@H]1CSCN1C(=O)OCC1c2ccccc2-c2ccccc12	43.972
AA21	CC(C)(C)c1ccc(C[C@@H](NC(=O)OCC2c3ccccc3-c3ccccc23)C(O)=O)cc1	132.094
AA22	OC(=O)[C@H](Cc1ccc(OP(O)(=O)OCc2ccccc2)cc1)NC(=O)OCC1c2ccccc2-c2ccccc12	171.993
AA23	OC(=O)[C@H]1CC[C@H](C1)NC(=O)OCC1c2ccccc2-c2ccccc12	40.031
AA24	OC(=O)CN1CCN(CC1)C(=O)OCC1c2ccccc2-c2ccccc12	55.042
AA25	OC(=O)C1(COC1)NC(=O)OCC1c2ccccc2-c2ccccc12	27.995
AA26	OC(=O)CC1CCN(CC1)C(=O)OCC1c2ccccc2-c2ccccc12	54.047
AA27	OC(=O)[C@@H]1CC2(CC2)CN1C(=O)OCC1c2ccccc2-c2ccccc12	52.031
AA28	OC(=O)[C@@H](Cc1ccc(Br)cc1)NC(=O)OCC1c2ccccc2-c2ccccc12	153.942
AA29	OC(=O)[C@@H]1C[C@H](F)CN1C(=O)OCC1c2ccccc2-c2ccccc12	44.006
AA30	OC(=O)c1ccc(NC(=O)OCC2c3ccccc3-c3ccccc23)cc1	48.000
AA31	OC(=O)[C@@H]1CC(F)(F)CN1C(=O)OCC1c2ccccc2-c2ccccc12	61.997
AA32	OC(=O)[C@@H](Cc1ccco1)NC(=O)OCC1c2ccccc2-c2ccccc12	66.011
AA33	OC(=O)[C@H](Cc1cncn1COCc1ccccc1)NC(=O)OCC1c2ccccc2-c2ccccc12	96.032
AA34	OC(=O)C1(CC1)NC(=O)OCC1c2ccccc2-c2ccccc12	12.000
AA35	O=C(O)[C@@H](N(C(OCC1C2=C(C3=C1C=CC=C3)C=CC=C2)=O)C)C4CCCC4	68.063
Ala	O=C(O)[C@H](C)NC(OCC1C2=CC=CC=C2C3=CC=CC=C13)=O	0.000
Arg	O=C(O)[C@@H](NC(OCC1C(C=CC=C2)=C2C3=C1C=CC=C3)=O)CCCNC(NS(C4=C(C)C(CC(C)(C)O5)=C5C(C)=C4C)(=O)=O)=N	85.064

Asn	O=C(O)[C@H](CC(NC(C1=CC=CC=C1)(C2=CC=CC=C2)C3=CC=CC=C3)=O)NC(OCC4C(C=CC=C5)=C5C6=C4C=CC=C6)=O	43.006
Asp	O=C(O)[C@@H](NC(OCC1C(C=CC=C2)=C2C3=C1C=CC=C3)=O)CC(OC(C)(C)C)=O	43.990
Gln	O=C(O)[C@H](CCC(NC(C1=CC=CC=C1)(C2=CC=CC=C2)C3=CC=CC=C3)=O)NC(OCC4C(C=CC=C5)=C5C6=C4C=CC=C6)=O	57.021
Glu	O=C(O)[C@@H](NC(OCC1C(C=CC=C2)=C2C3=C1C=CC=C3)=O)CCC(OC(C)(C)C)=O	58.005
Gly	O=C(O)CNC(OCC1C2=CC=CC=C2C3=CC=CC=C13)=O	-14.016
His	O=C(O)[C@@H](NC(OCC1C(C=CC=C2)=C2C3=C1C=CC=C3)=O)CC4=CN(C(C5=CC=CC=C5)(C6=CC=CC=C6)C7=CC=CC=C7)C=N4	66.022
Leu	O=C(O)[C@H](CC(C)C)NC(OCC1C2=CC=CC=C2C3=CC=CC=C13)=O	42.047
Lys	O=C(O)[C@@H](NC(OCC1C(C=CC=C2)=C2C3=C1C=CC=C3)=O)CCCCNC(OC(C)(C)C)=O	57.058
Phe	O=C(O)[C@H](CC1=CC=CC=C1)NC(OCC2C3=CC=CC=C3C4=CC=CC=C24)=O	76.031
Pro	O=C(O)[C@H]1N(C(OCC2C3=CC=CC=C3C4=CC=CC=C24)=O)CCC1	26.016
Ser	O=C(O)[C@@H](NC(OCC1C(C=CC=C2)=C2C3=C1C=CC=C3)=O)COC(C)(C)C	15.995
Thr	O=C(O)[C@@H](NC(OCC1C(C=CC=C2)=C2C3=C1C=CC=C3)=O)C(C)OC(C)(C)C	30.011
Trp	O=C(O)[C@@H](NC(OCC1C(C=CC=C2)=C2C3=C1C=CC=C3)=O)CC4=CN(C(OC(C)(C)C)=O)C5=C4C=CC=C5	115.042
Tyr	O=C(O)[C@@H](NC(OCC1C(C=CC=C2)=C2C3=C1C=CC=C3)=O)CC4=CC=C(OC(C)(C)C)C=C4	92.026

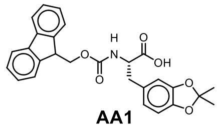

AA1

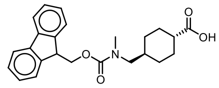

AA2

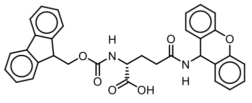

AA3

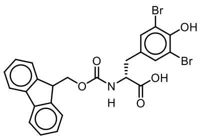

AA4

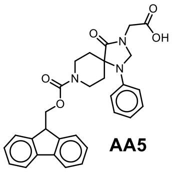

AA5

AA6

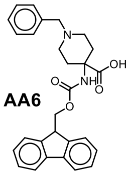

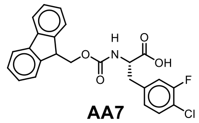

AA7

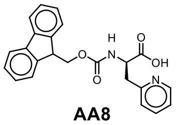

AA8

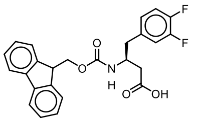

AA9

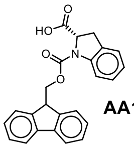

AA10

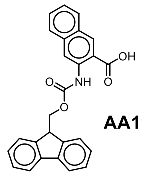

AA11

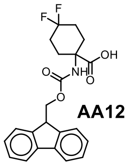

AA12

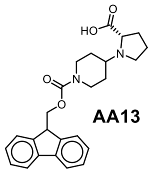

AA13

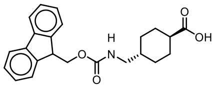

AA14

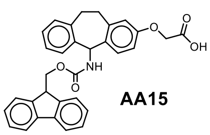

AA15

AA16

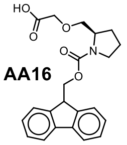

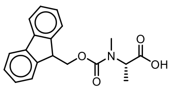

AA17

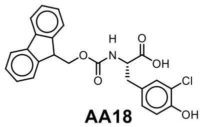

AA18

AA19

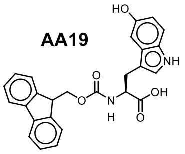

AA20

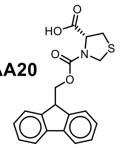

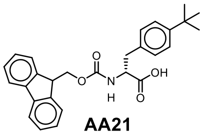

AA21

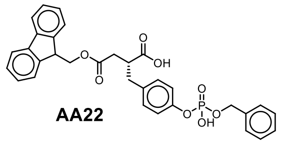

AA22

AA23

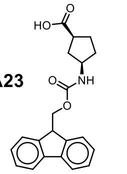

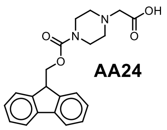

AA24

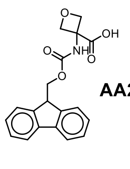

AA25

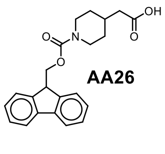

AA26

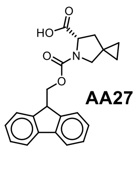

AA27

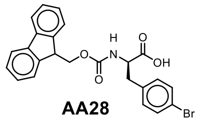

AA28

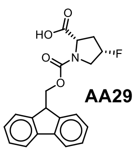

AA29

AA39

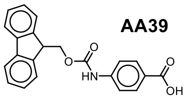

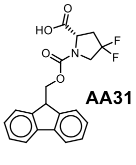

AA31

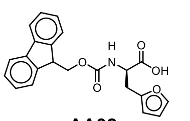

AA32

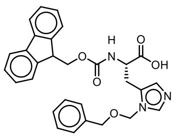

AA33

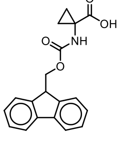

AA34

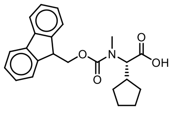

AA35

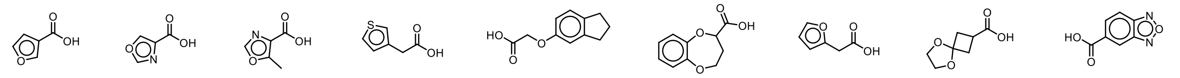

CA1 CA2 CA3 CA4 CA5 CA6 CA7 CA8 CA9

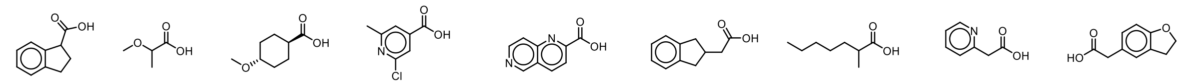

CA10 CA11 CA12 CA13 CA14 CA15 CA16 CA17 CA18

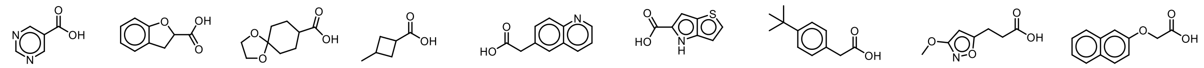

CA19 CA20 CA21 CA22 CA23 CA24 CA25 CA26 CA27

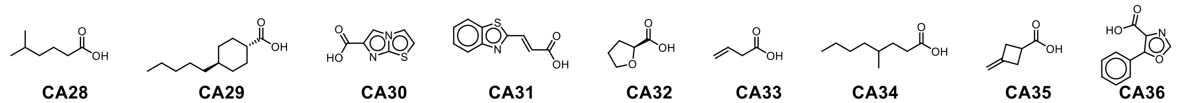

CA28 CA29 CA30 CA31 CA32 CA33 CA34 CA35 CA36

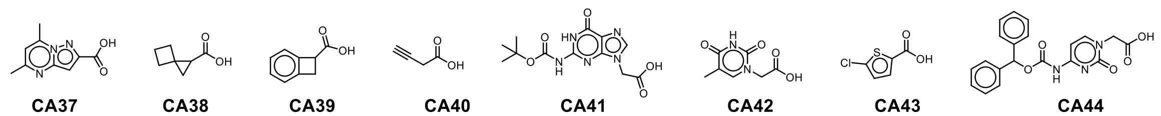

CA37 CA38 CA39 CA40 CA41 CA42 CA43 CA44

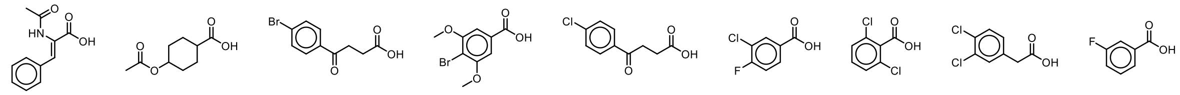

CA45 CA46 CA47 CA48 CA49 CA50 CA51 CA52 CA53

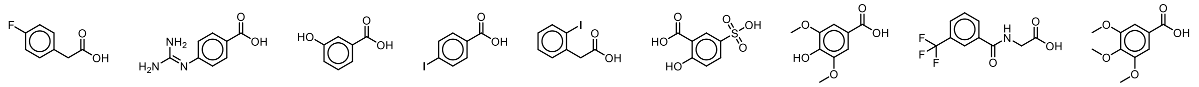

CA54 CA55 CA56 CA57 CA58 CA59 CA60 CA61 CA62

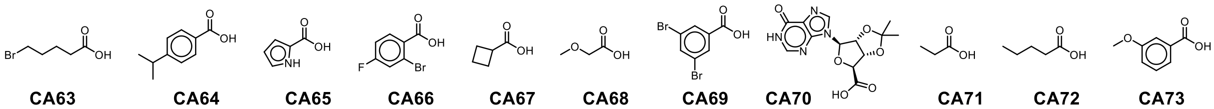

CA63 CA64 CA65 CA66 CA67 CA68 CA69 CA70 CA71 CA72 CA73

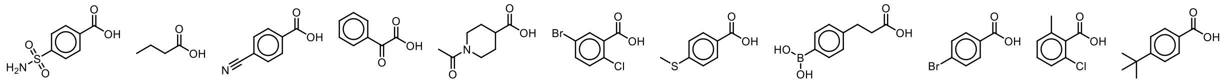

CA74 CA75 CA76 CA77 CA78 CA79 CA80 CA81 CA82 CA83 CA84

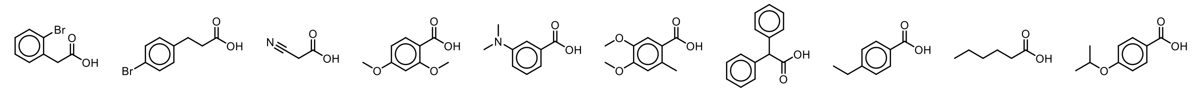

CA85 CA86 CA87 CA88 CA89 CA90 CA91 CA92 CA93 CA94

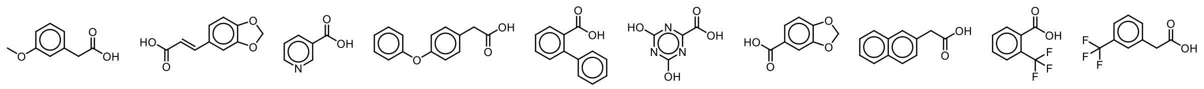

CA95 CA96 CA97 CA98 CA99 CA100 CA101 CA102 CA103 CA104

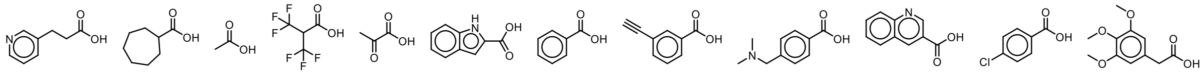

CA105 CA106 CA107 CA108 CA109 CA110 CA111 CA112 CA113 CA114 CA115 CA116

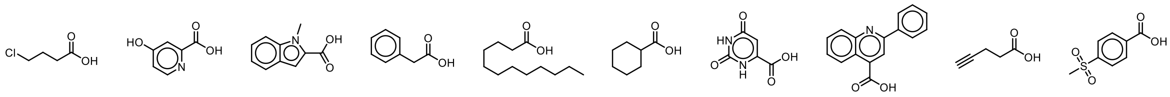

CA117 CA118 CA119 CA120 CA121 CA122 CA123 CA124 CA125 CA126

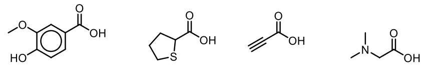

CA127 CA128 CA129 CA130

5. Library designs

Test libraries:

Library design C(X)$_3$CK:

N-term					C-term
Cys	AA28	AA32	AA25	Cys	Lys
Cys	AA29	AA27	AA12	Cys	Lys
Cys	Arg	Gly	Thr	Cys	Lys
Cys	Phe	Val	Pro	Cys	Lys

Library design C(X)$_4$CK:

**N-term**						**C-term**
Cys	AA24	AA28	AA32	AA25	Cys	Lys
Cys	AA5	AA29	AA27	AA12	Cys	Lys
Cys	Glu	Arg	Gly	Thr	Cys	Lys
Cys	Asn	Phe	Val	Pro	Cys	Lys

Library design AcC(X)$_3$CK:

**N-term**						**C-term**
Ac	Cys	AA28	AA32	AA25	Cys	Lys
Ac	Cys	AA29	AA27	AA12	Cys	Lys
Ac	Cys	Arg	Gly	Thr	Cys	Lys
Ac	Cys	Phe	Val	Pro	Cys	Lys

Library design AcC(X)$_4$CK:

**N-term**							**C-term**
Ac	Cys	AA24	AA28	AA32	AA25	Cys	Lys
Ac	Cys	AA5	AA29	AA27	AA12	Cys	Lys
Ac	Cys	Glu	Arg	Gly	Thr	Cys	Lys
Ac	Cys	Asn	Phe	Val	Pro	Cys	Lys

Library design Mpa(X)$_3$CK:

**N-term**					**C-term**
Mpa	AA28	AA32	AA25	Cys	Lys
Mpa	AA29	AA27	AA12	Cys	Lys
Mpa	Arg	Gly	Thr	Cys	Lys
Mpa	Phe	Val	Pro	Cys	Lys

**Library design Mpa(X)$_4$CK:**						
**N-term**						**C-term**
Mpa	AA24	AA28	AA32	AA25	Cys	Lys
Mpa	AA5	AA29	AA27	AA12	Cys	Lys
Mpa	Glu	Arg	Gly	Thr	Cys	Lys
Mpa	Asn	Phe	Val	Pro	Cys	Lys

CycloSEL-16M design:

Y			Cys	X=1	X=2	X=3	Cys	Lys
CA1	CA51	CA101		AA1	AA1	AA1		
CA2	CA52	CA102		AA2	AA2	AA2		
CA3	CA53	CA103		AA3	AA3	AA3		
CA4	CA54	CA104		AA4	AA4	AA4		
CA5	CA55	CA105		AA5	AA5	AA5		
CA6	CA56	CA106		AA6	AA6	AA6		
CA7	CA57	CA107		AA7	AA7	AA7		
CA8	CA58	CA108		AA8	AA8	AA8		
CA9	CA59	CA109		AA9	AA9	AA9		
CA10	CA60	CA110		AA10	AA10	AA10		
CA11	CA61	CA111		AA11	AA11	AA11		
CA12	CA62	CA112		AA12	AA12	AA12		
CA13	CA63	CA113		AA13	AA13	AA13		
CA14	CA64	CA114		AA14	AA14	AA14		
CA15	CA65	CA115		AA15	AA15	AA15		
CA16	CA66	CA116		AA16	AA16	AA16		
CA17	CA67	CA117		AA17	AA17	AA17		
CA18	CA68	CA118		AA18	AA18	AA18		
CA19	CA69	CA119		AA19	AA19	AA19		
CA20	CA70	CA120		AA20	AA20	AA20		
CA21	CA71	CA121		AA21	AA21	AA21		
CA22	CA72	CA122		AA22	AA22	AA22		
CA23	CA73	CA123		AA23	AA23	AA23		
CA24	CA74	CA124		AA24	AA24	AA24		
CA25	CA75	CA125		AA25	AA25	AA25		
CA26	CA76	CA126		AA26	AA26	AA26		
CA27	CA77	CA127		AA27	AA27	AA27		
CA28	CA78	CA128		AA28	AA28	AA28		
CA29	CA79	CA129		AA29	AA29	AA29		
CA30	CA80	CA130		AA30	AA30	AA30		

CA31	CA81			AA31	AA31	AA31		
CA32	CA82			AA32	AA32	AA32		
CA33	CA83			AA33	AA33	AA33		
CA34	CA84			AA34	AA34	AA34		
CA35	CA85			Ala	Ala	Ala		
CA36	CA86			Arg	Arg	Arg		
CA37	CA87			Asn	Asn	Asn		
CA38	CA88			Asp	Asp	Asp		
CA39	CA89			Gln	Gln	Gln		
CA40	CA90			Glu	Glu	Glu		
CA41	CA91			Gly	Gly	Gly		
CA42	CA92			His	His	His		
CA43	CA93			Leu	Leu	Leu		
CA44	CA94			Lys	Lys	Lys		
CA45	CA95			Phe	Phe	Phe		
CA46	CA96			Pro	Pro	Pro		
CA47	CA97			Ser	Ser	Ser		
CA48	CA98			Thr	Thr	Thr		
CA49	CA99			Trp	Trp	Trp		
CA50	CA100			Tyr	Tyr	Tyr		

6. Sequencing outcome for library scaffold optimization

6.1 C(X)$_3$CK design:

MS/MS analysis of library design C(X)$_3$CK Linear at 100 fmol/member, ALC > 70%:
Sequencing recall (duplicates combined): 88% (56/64 sequences detected)
Sequenced building block distribution:

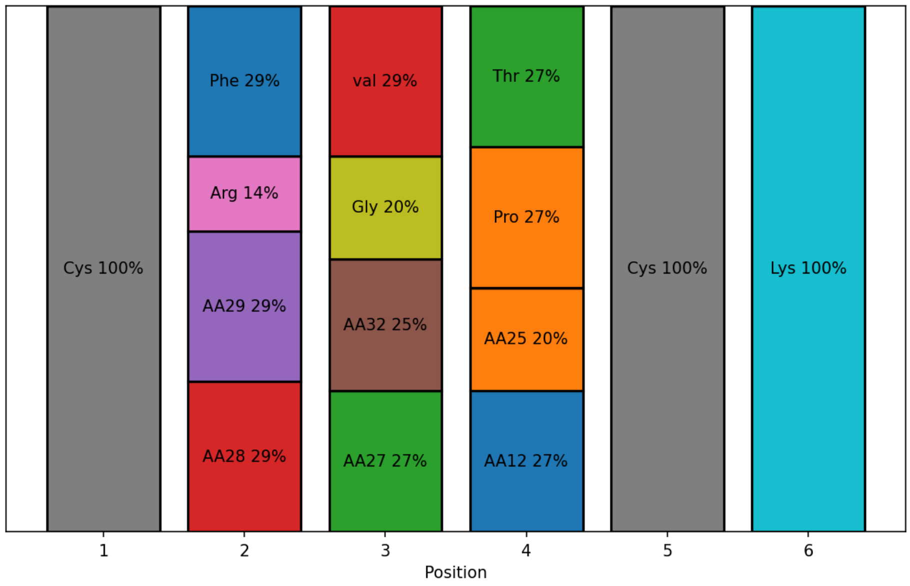

Library MS/MS analysis of library design C(X)$_3$CK Cyclic at 100 fmol/member, ALC > 70%:
Sequencing recall (duplicates combined): 8% (5/64 sequences detected)
Sequenced building block distribution:

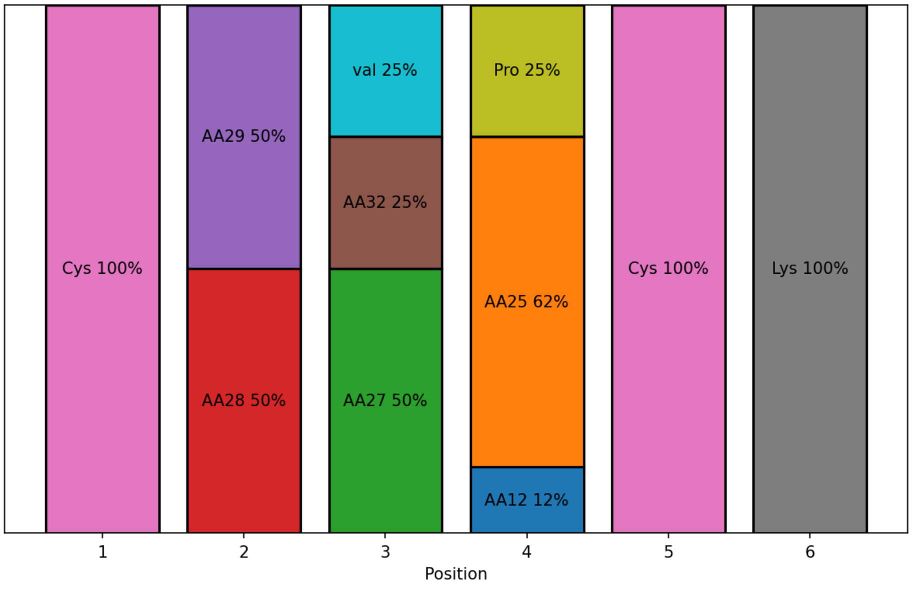

Library MS/MS analysis of library design C(X)$_3$CK Linearized at 100 fmol/member, ALC > 70%:
Sequencing recall (duplicates combined): 91% (58/64 sequences detected)
Sequenced building block distribution:

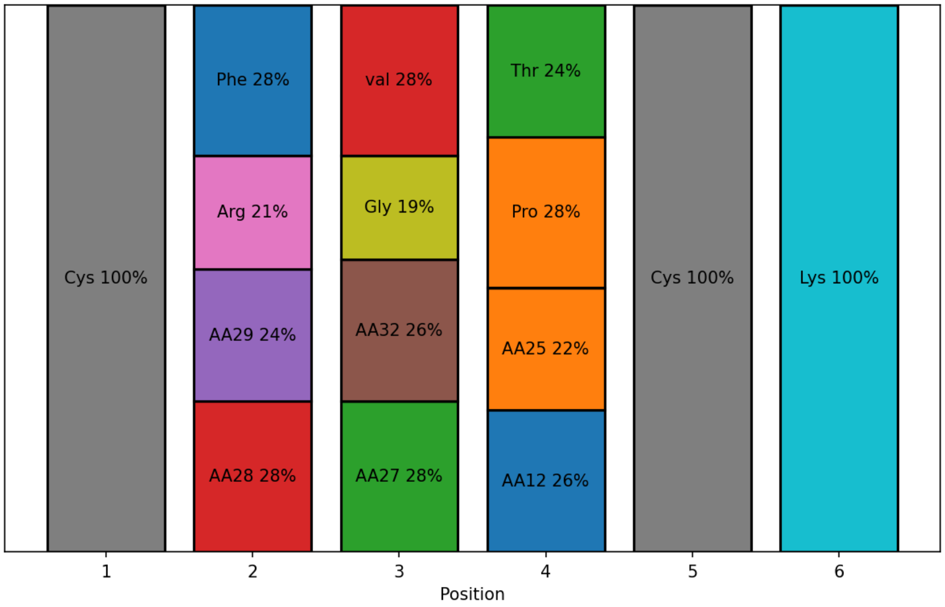

6.2 C(X)$_4$CK design:

Library MS/MS analysis of library design C(X)$_4$CK Linear at 100 fmol/member, ALC > 70%:
Sequencing recall (duplicates combined): 73% (186/256 sequences detected)
Sequenced building block distribution:

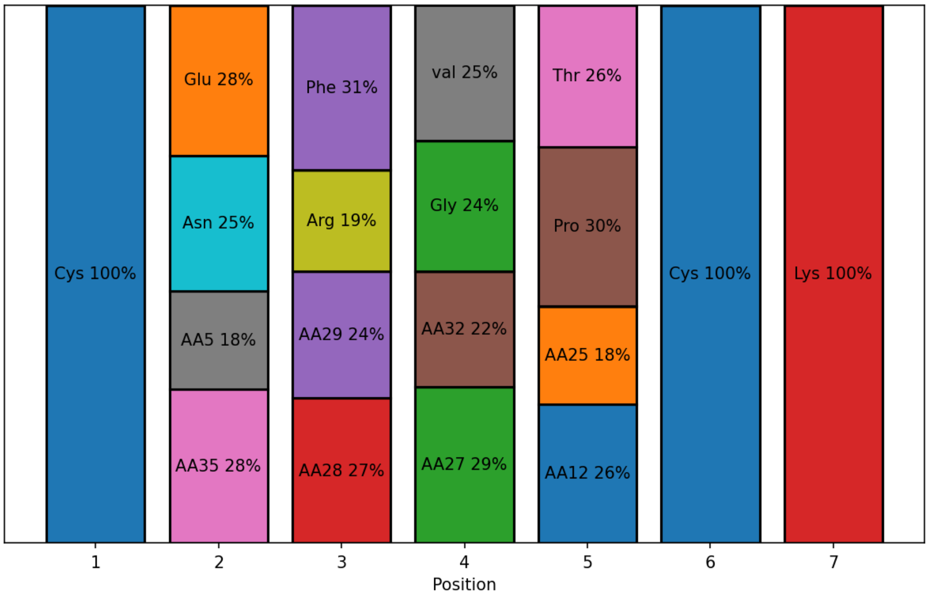

Library MS/MS analysis of library design C(X)$_4$CK Cyclic at 100 fmol/member, ALC > 70%:
Sequencing recall (duplicates combined): 5% (14/256 sequences detected)
Sequenced building block distribution:

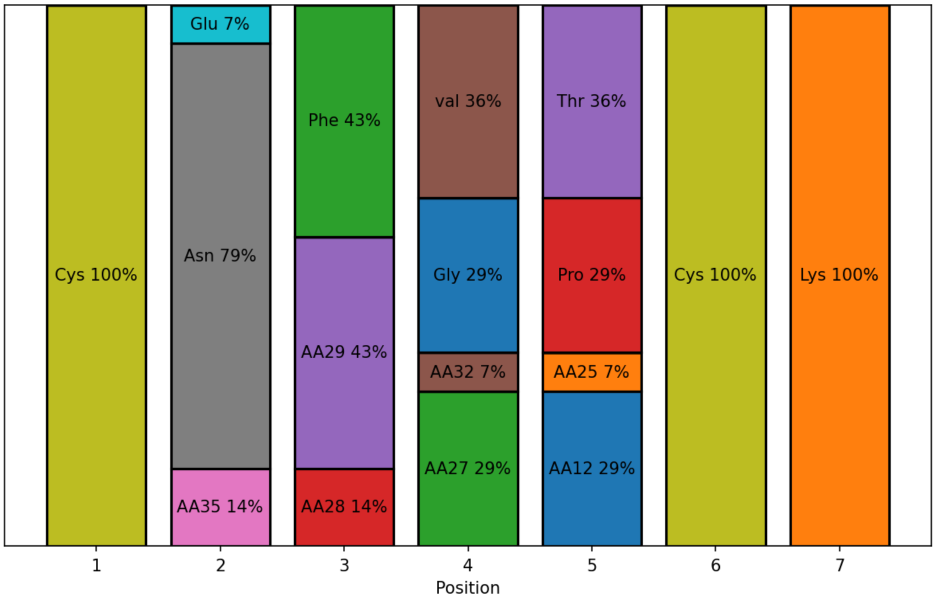

Library MS/MS analysis of library design C(X)$_4$CK Linearized at 100 fmol/member, ALC > 70%:
Sequencing recall (duplicates combined): 73% (187/256 sequences detected)
Sequenced building block distribution:

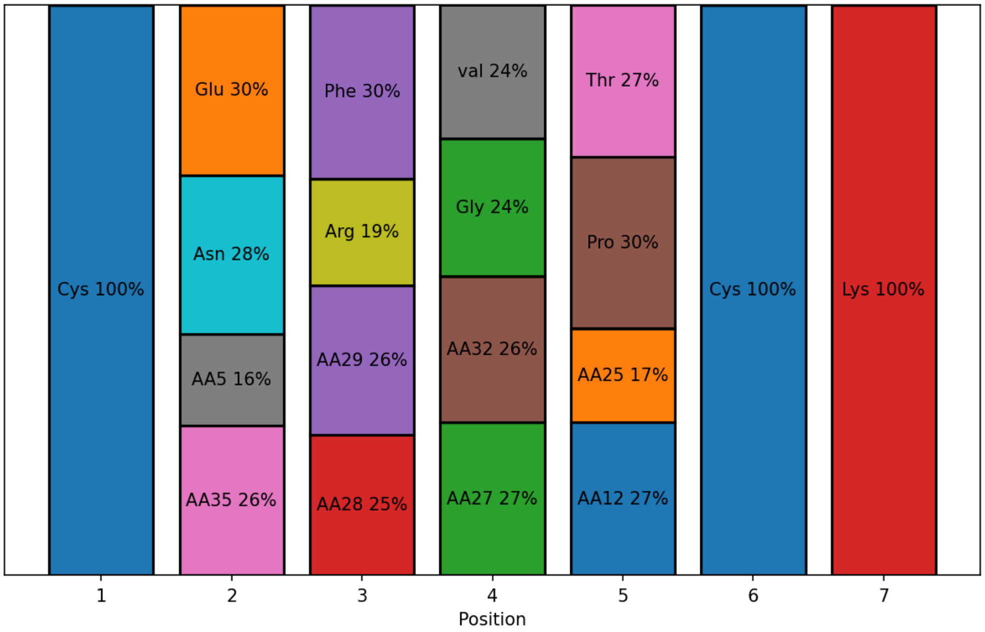

6.3 AcC(X)$_3$CK design:

Library MS/MS analysis of library design AcC(X)$_3$CK Linear at 100 fmol/member, ALC > 70%:
Sequencing recall (duplicates combined): 98% (63/64 sequences detected)
Sequenced building block distribution:

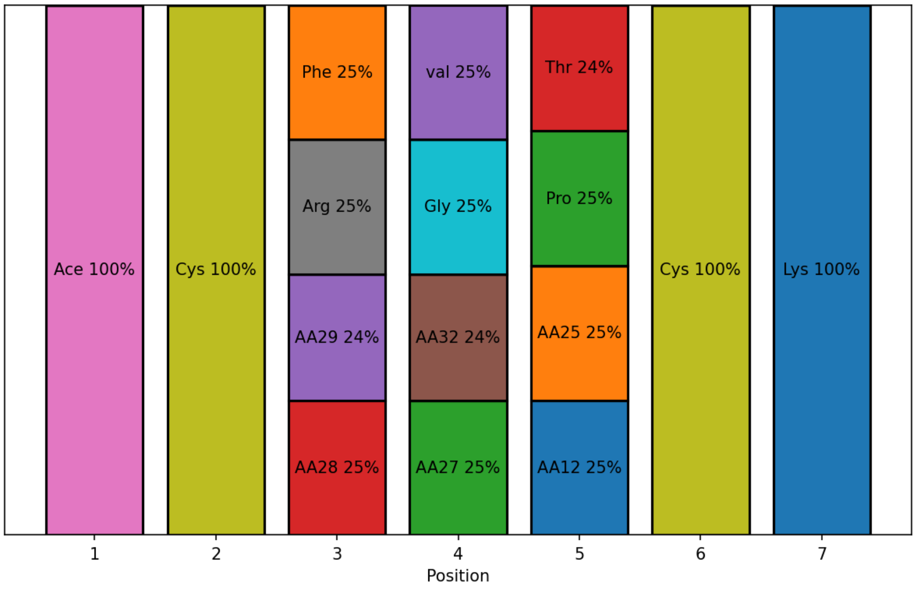

Library MS/MS analysis of library design AcC(X)$_3$CK Cyclic at 100 fmol/member, ALC > 70%:
Sequencing recall (duplicates combined): 11% (7/64 sequences detected)
Sequenced building block distribution:

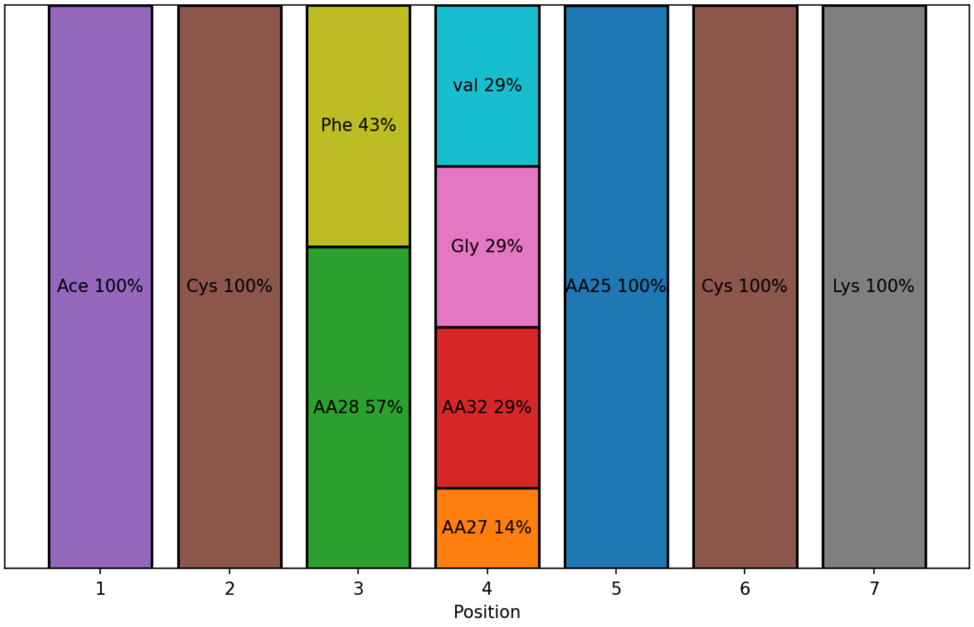

Library MS/MS analysis of library design AcC(X)$_3$CK Linearized at 100 fmol/member, ALC > 70%:
Sequencing recall (duplicates combined): 100% (64/64 sequences detected)
Sequenced building block distribution:

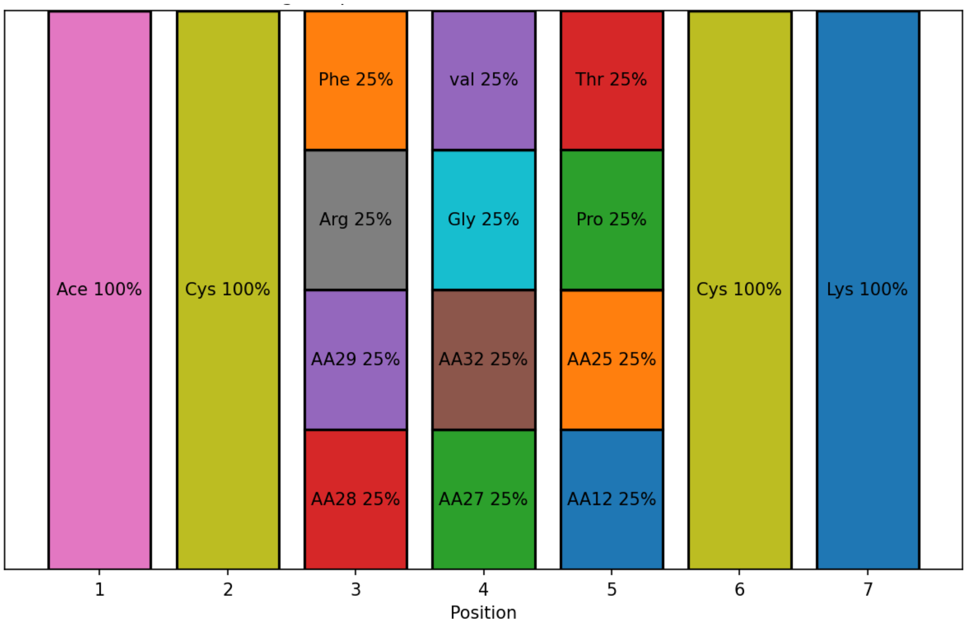

6.4 AcC(X)$_4$CK design:

Library MS/MS analysis of library design AcC(X)$_4$CK Linear at 100 fmol/member, ALC > 70%:
Sequencing recall (duplicates combined): 62% (159/256 sequences detected)
Sequenced building block distribution:

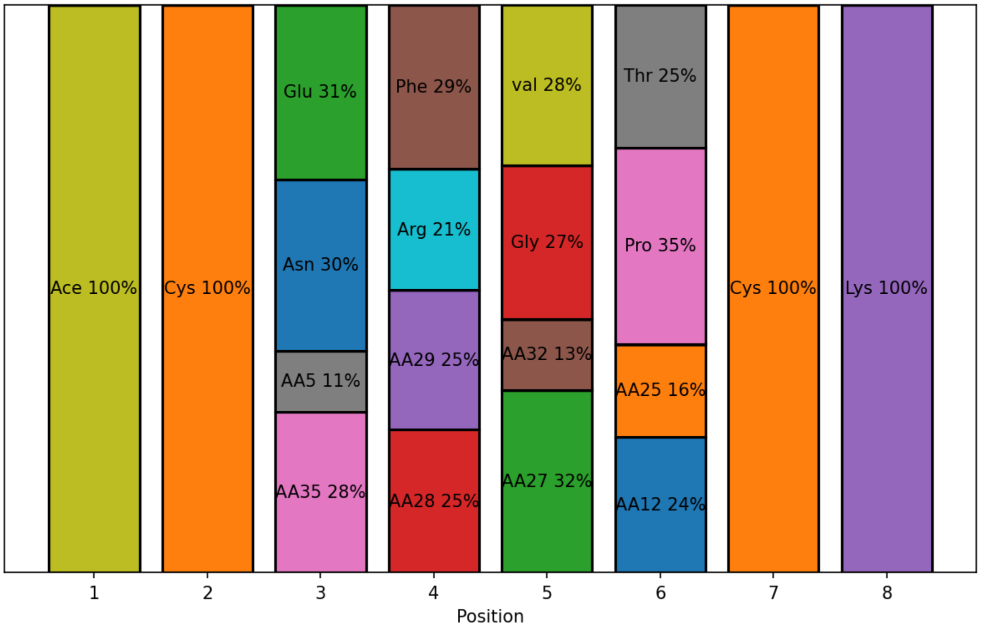

Library MS/MS analysis of library design Ac<u>C(X)$_4$CK Cyclic</u> at 100 fmol/member, ALC > 70%:
Sequencing recall (duplicates combined): 0% (0/256 sequences detected)

Library MS/MS analysis of library design Ac<u>C(X)$_4$CK Linearized</u> at 100 fmol/member, ALC > 70%:
Sequencing recall (duplicates combined): 73% (186/256 sequences detected)
Sequenced building block distribution:

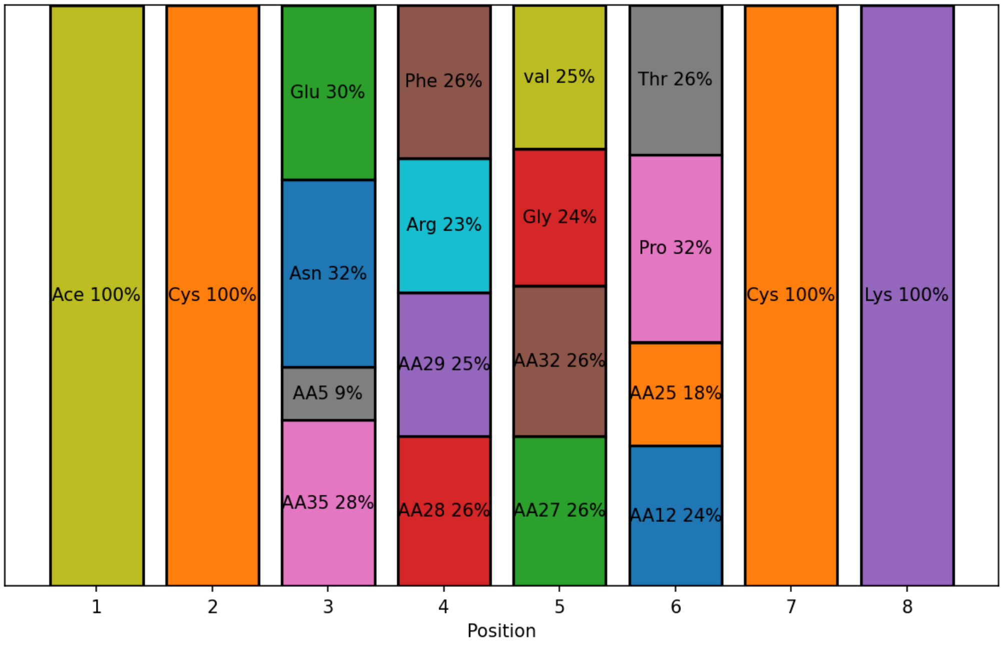

6.5 Mpa(X)$_3$CK design:

Library MS/MS analysis of library design <u>Mpa(X)$_3$CK Linear</u> at 100 fmol/member, ALC > 70%:
Sequencing recall (duplicates combined): 95% (61/64 sequences detected)
Sequenced building block distribution:

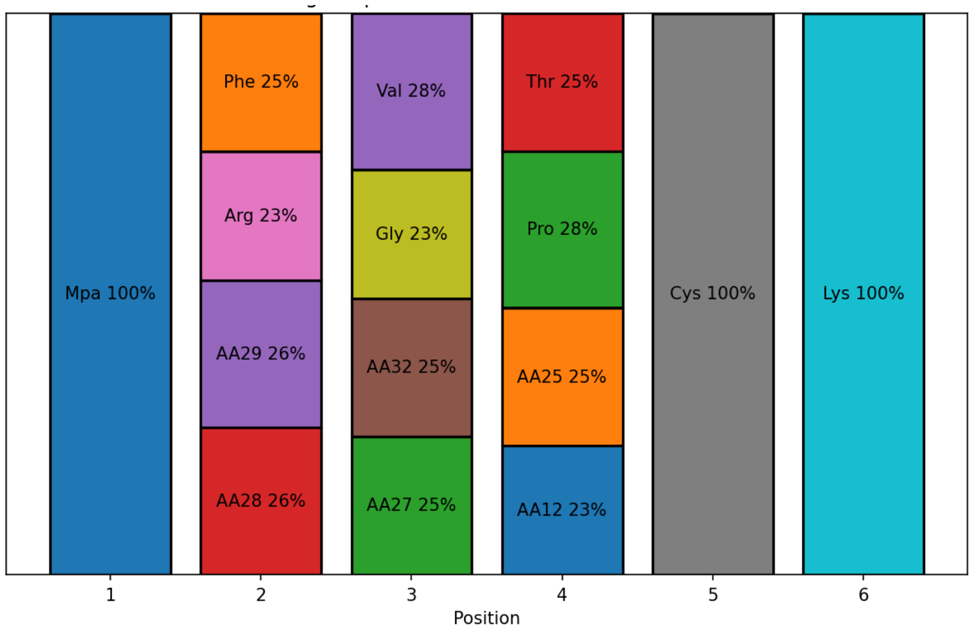

Library MS/MS analysis of library design <u>Mpa(X)$_3$CK Cyclic</u> at 100 fmol/member, ALC > 70%:
Sequencing recall (duplicates combined): 2% (1/64 sequences detected)
Sequenced building block distribution:

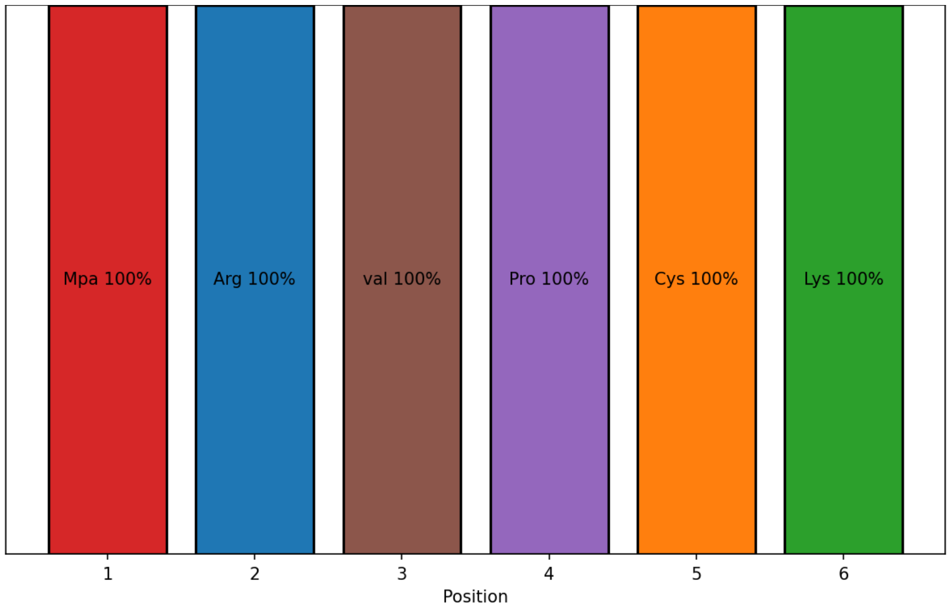

Library MS/MS analysis of library design Mpa(X)$_3$CK Linearized at 100 fmol/member, ALC > 70%:
Sequencing recall (duplicates combined): 98% (63/64 sequences detected)
Sequenced building block distribution:

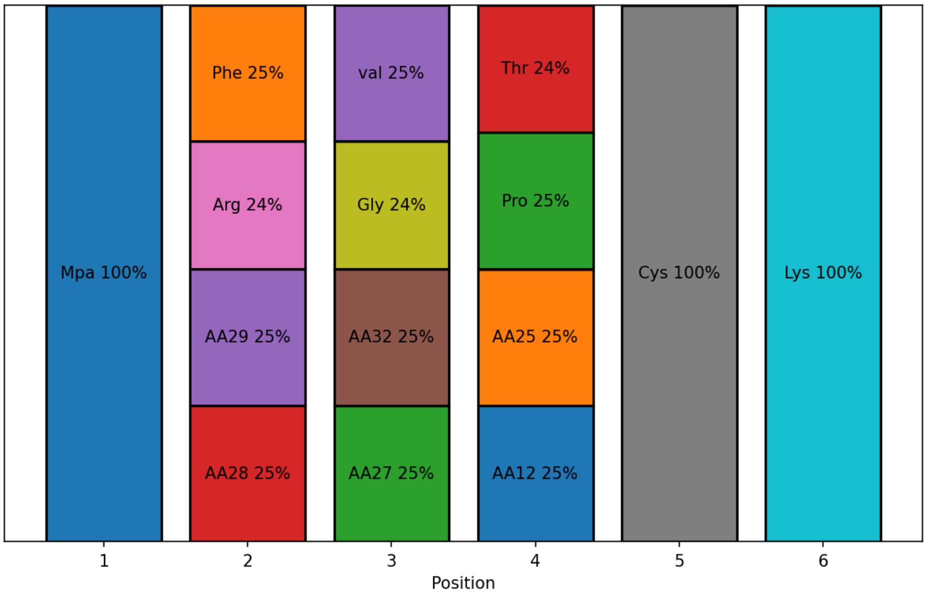

6.6 Mpa(X)$_4$CK design:

Library MS/MS analysis of library design Mpa(X)$_4$CK Linear at 100 fmol/member, ALC > 70%:
Sequencing recall (duplicates combined): 68% (175/256 sequences detected)
Sequenced building block distribution:

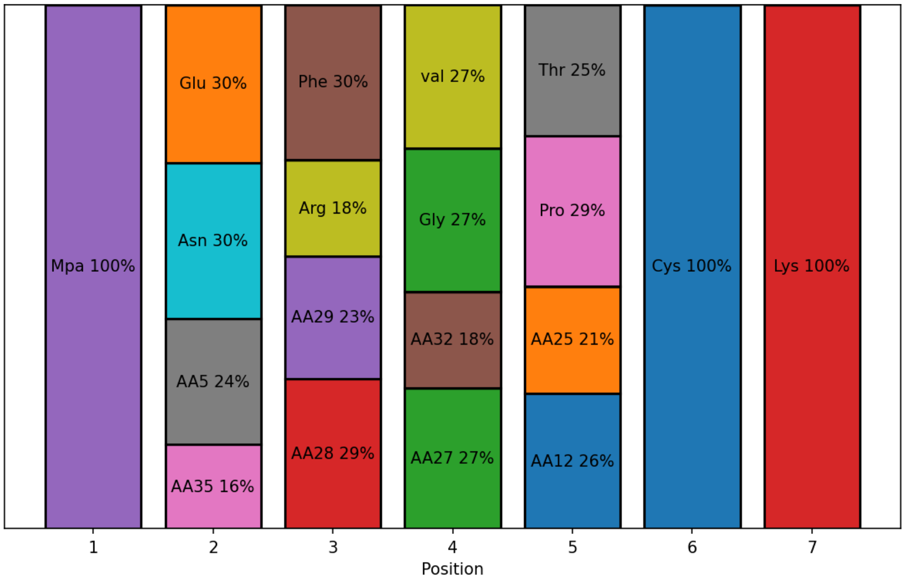

Library MS/MS analysis of library design Mpa(X)$_4$CK Cyclic at 100 fmol/member, ALC > 70%:
Sequencing recall (duplicates combined): 0% (1/256 sequences detected)
Sequenced building block distribution:

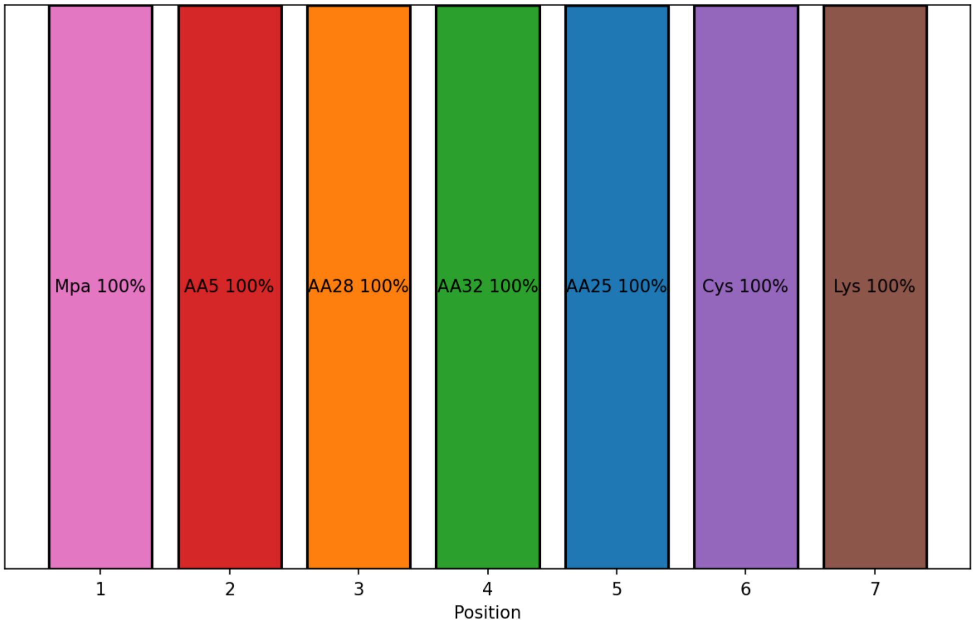

Library MS/MS analysis of library design Mpa(X)$_4$CK Linearized at 100 fmol/member, ALC > 70%:
Sequencing recall (duplicates combined): 77% (197/256 sequences detected)
Sequenced building block distribution:

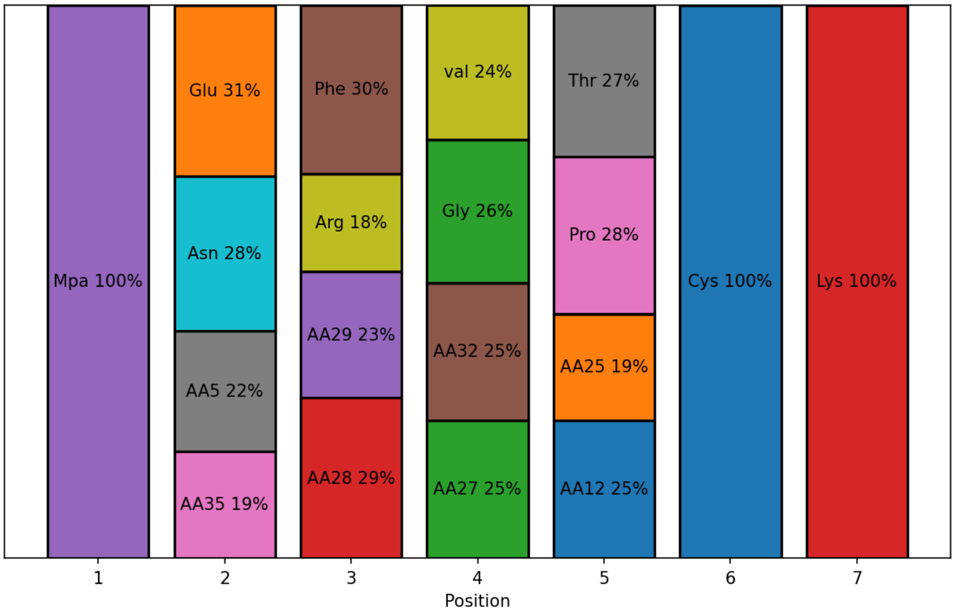

7. Affinity selection against CAIX

7.1 Procedure

MyOne Streptavidin T1 Dynabeads (100 µL of 10 mg/mL stock per well, measured in duplicates) were washed with 3x 1 mL 10% FBS, 1x PBS, 0.02% Tween-20. The beads were incubated with biotinylated CAIX (100 µL, 1.5 µM per well) in 10% FBS, 1x PBS, 0.02% Tween-20 for 1h at 4°C. The beads were washed 2x 1 mL 10% FBS, 1x PBS, 0.02% Tween20, 400 µM d-biotin and 1x 1 mL 10% FBS, 1x PBS, 0.02% Tween-20 before incubating with the library (1000 µL, 10 fmol/member per well) in 10% FBS, 1x PBS for 1h at 4°C. The beads were washed with 5x 1 mL of 1x PBS and subsequently eluted with 2x 6M Guanidine HCl in 20mM phosphate buffer pH 6.8 supplemented with 10 mM TCEP HCl (100 µL per well).

<u>Sample preparation after AS</u>: The StageTips were prepared as described by Rappsilber et al. using C18 material from Empore SPE 47 mm discs (66883-U, Merck) (46). The StageTips were pre-conditioned with 100 µL MeOH, 100 µL of 0.1% (v/v) FA in ACN and 100 µL of 0.1% (v/v) FA in MQ, respectively by centrifuging for 3 min at 300 rpm. The samples were then loaded on the StageTips and washed with 100 µL of 0.1% (v/v) FA in MQ. Compounds were eluted by adding 100 µL of 0.1% (v/v) FA in ACN:MQ (7:3). The samples were lyophilized before resuspending in 10 µL 0.1% (v/v) FA in UPLC-MS grade water. Afterwards, 9 µL was injected into the LC-MS/MS system.

7.2 List of filtered sequences

Table S4: Filtered sequences of CAIX selection. In green are represented sequences bearing the sulfonamide CA74 building block.

CA		X=1	X=2	X=3		
CA74	cys	AA14	AA13	AA23	cys	Lys
CA74	cys	AA30	AA23	AA13	cys	Lys
CA74	cys	AA26	AA13	AA23	cys	Lys
CA74	cys	AA26	AA23	AA13	cys	Lys
CA74	cys	AA1	Lys	Phe	cys	Lys
CA74	cys	Asp	Ala	AA2	cys	Lys
CA74	cys	AA1	AA13	AA23	cys	Lys
CA74	cys	AA29	AA14	Arg	cys	Lys
CA74	cys	Asp	Gln	Phe	cys	Lys
CA74	cys	Thr	AA8	AA16	cys	Lys
CA74	cys	Glu	AA13	AA23	cys	Lys
CA74	cys	AA17	AA17	Pro	cys	Lys
CA74	cys	Glu	AA23	AA13	cys	Lys
CA74	cys	AA7	Arg	Gly	cys	Lys
CA74	cys	AA17	Gly	AA26	cys	Lys

CA74	cys	AA14	AA23	AA13	cys	Lys
CA74	cys	AA1	AA23	AA13	cys	Lys
CA74	cys	Ala	Gly	AA23	cys	Lys
CA74	cys	AA17	AA26	Gly	cys	Lys
CA74	cys	AA9	Tyr	Arg	cys	Lys
CA74	cys	Tyr	AA24	AA26	cys	Lys
CA74	cys	Phe	Trp	Arg	cys	Lys
CA74	cys	Asp	Tyr	Ser	cys	Lys
CA74	cys	Arg	AA17	AA23	cys	Lys
CA74	cys	AA17	AA23	Ala	cys	Lys
CA74	cys	AA9	Gly	Asn	cys	Lys
CA74	cys	Trp	Phe	Arg	cys	Lys
CA74	cys	Arg	AA26	Pro	cys	Lys
CA74	cys	Lys	Asp	AA2	cys	Lys
CA74	cys	AA7	His	Gln	cys	Lys
CA74	cys	AA23	AA23	AA13	cys	Lys
CA74	cys	Gln	AA13	AA23	cys	Lys
CA74	cys	Gln	Gln	Pro	cys	Lys
CA74	cys	Arg	Pro	AA26	cys	Lys
CA74	cys	Tyr	AA8	AA16	cys	Lys
CA74	cys	Ala	Ala	Pro	cys	Lys
CA74	cys	Gly	Trp	AA17	cys	Lys
CA74	cys	AA9	Ala	Gln	cys	Lys
CA74	cys	Trp	Ala	Pro	cys	Lys
CA74	cys	AA9	Arg	His	cys	Lys
CA74	cys	Asp	AA17	Ala	cys	Lys
CA74	cys	AA13	Leu	Pro	cys	Lys
CA74	cys	AA16	Arg	Pro	cys	Lys
CA74	cys	Phe	Lys	AA2	cys	Lys
CA74	cys	Gly	AA8	AA27	cys	Lys
CA74	cys	Arg	AA14	AA34	cys	Lys
CA74	cys	Gln	AA23	AA13	cys	Lys
CA74	cys	AA26	AA32	AA13	cys	Lys
CA74	cys	Ala	AA13	AA23	cys	Lys
CA74	cys	His	Phe	Asn	cys	Lys
CA74	cys	AA26	Gln	Arg	cys	Lys
CA74	cys	AA23	Asn	Gln	cys	Lys
CA74	cys	Gln	AA14	Lys	cys	Lys
CA74	cys	Ala	Ala	AA26	cys	Lys
CA74	cys	Arg	Ala	AA26	cys	Lys
CA74	cys	Thr	AA34	AA16	cys	Lys
CA74	cys	AA13	AA14	AA23	cys	Lys
CA74	cys	Phe	His	Asn	cys	Lys

CA74	cys	AA16	Pro	Arg	cys	Lys
CA74	cys	Gln	AA14	AA13	cys	Lys
CA74	cys	AA34	AA1	Glu	cys	Lys
CA74	cys	AA1	AA8	Leu	cys	Lys
CA74	cys	Asp	AA14	AA13	cys	Lys
CA74	cys	Thr	AA14	AA13	cys	Lys
CA74	cys	AA32	AA23	AA13	cys	Lys
CA74	cys	AA14	Asp	AA13	cys	Lys
CA74	cys	AA23	AA30	AA13	cys	Lys
CA74	cys	AA23	Ser	AA26	cys	Lys
CA74	cys	Asp	AA2	AA13	cys	Lys
CA74	cys	Gly	Pro	AA23	cys	Lys
CA74	cys	Pro	AA2	AA13	cys	Lys
CA74	cys	Ser	Arg	AA26	cys	Lys
CA74	cys	Glu	AA17	AA23	cys	Lys
CA74	cys	AA26	Ala	AA29	cys	Lys
CA74	cys	AA17	AA29	Ala	cys	Lys
CA74	cys	Glu	AA14	Lys	cys	Lys
CA74	cys	Ala	AA23	AA13	cys	Lys
CA74	cys	Lys	Leu	His	cys	Lys
CA74	cys	Ser	AA14	Arg	cys	Lys
CA74	cys	AA6	Asp	AA1	cys	Lys
CA74	cys	AA27	AA12	AA8	cys	Lys
CA74	cys	Tyr	AA13	AA23	cys	Lys
CA74	cys	Tyr	AA23	AA13	cys	Lys
CA74	cys	Thr	AA14	Arg	cys	Lys
CA74	cys	Glu	Trp	Arg	cys	Lys
CA74	cys	Glu	Ala	AA29	cys	Lys
CA74	cys	AA29	AA23	AA13	cys	Lys
CA74	cys	Gly	AA23	Pro	cys	Lys
CA74	cys	AA13	AA14	Thr	cys	Lys
CA74	cys	AA6	Ala	AA16	cys	Lys
CA74	cys	Ser	AA2	AA13	cys	Lys
CA74	cys	Asp	Ala	AA17	cys	Lys
CA74	cys	Gly	AA34	AA26	cys	Lys
CA74	cys	AA7	AA26	AA23	cys	Lys
CA74	cys	AA23	Thr	AA23	cys	Lys
CA74	cys	Gly	Arg	AA26	cys	Lys
CA74	cys	AA10	Ala	AA23	cys	Lys
CA74	cys	Ala	Gly	AA26	cys	Lys
CA74	cys	Ala	Gly	AA31	cys	Lys
CA74	cys	AA23	AA14	AA13	cys	Lys
CA74	cys	AA23	AA14	Arg	cys	Lys

CA74	cys	Gln	AA26	Gly	cys	Lys
CA74	cys	Phe	AA32	AA23	cys	Lys
CA74	cys	AA6	AA32	AA2	cys	Lys
CA74	cys	Phe	Gly	Gly	cys	Lys
CA74	cys	Thr	AA20	AA23	cys	Lys
CA74	cys	AA6	Ser	AA26	cys	Lys
CA74	cys	AA9	Gly	Ala	cys	Lys
CA74	cys	Glu	Arg	AA2	cys	Lys
CA74	cys	Gln	Lys	AA26	cys	Lys
CA74	cys	AA23	Arg	AA23	cys	Lys
CA74	cys	AA30	Pro	AA16	cys	Lys
CA74	cys	Lys	AA29	AA26	cys	Lys
CA74	cys	Phe	Gln	AA13	cys	Lys
CA74	cys	Thr	AA23	AA13	cys	Lys
CA74	cys	His	AA34	AA2	cys	Lys
CA74	cys	Lys	Phe	Arg	cys	Lys
CA74	cys	Leu	Gly	Ala	cys	Lys
CA74	cys	AA14	Thr	AA13	cys	Lys
CA74	cys	AA23	Leu	Pro	cys	Lys
CA74	cys	His	Asp	AA2	cys	Lys
CA74	cys	AA29	Lys	AA26	cys	Lys
CA74	cys	AA34	AA23	AA13	cys	Lys
CA74	cys	AA14	Arg	AA23	cys	Lys
CA74	cys	AA20	Thr	AA23	cys	Lys
CA74	cys	AA11	AA34	AA26	cys	Lys
CA74	cys	Arg	AA34	Leu	cys	Lys
CA74	cys	Lys	AA29	AA13	cys	Lys
CA74	cys	Asp	AA34	AA26	cys	Lys
CA74	cys	Ala	Gly	AA14	cys	Lys
CA74	cys	AA29	Glu	AA13	cys	Lys
CA74	cys	Leu	Pro	AA17	cys	Lys
CA74	cys	AA23	AA20	AA13	cys	Lys
CA74	cys	Arg	Leu	AA34	cys	Lys
CA74	cys	AA6	Lys	AA26	cys	Lys
CA74	cys	Ala	AA29	AA9	cys	Lys
CA74	cys	Trp	Ala	Gly	cys	Lys
CA74	cys	Asn	AA34	AA13	cys	Lys
CA74	cys	Arg	AA23	AA17	cys	Lys
CA74	cys	AA10	AA23	Pro	cys	Lys
CA74	cys	AA34	Asn	AA13	cys	Lys
CA74	cys	AA32	AA34	AA13	cys	Lys
CA74	cys	Ala	AA29	Gly	cys	Lys
CA74	cys	AA17	Ala	AA29	cys	Lys

CA74	cys	Asp	Leu	AA23	cys	Lys
CA74	cys	AA17	Glu	AA23	cys	Lys
CA74	cys	AA30	AA23	AA27	cys	Lys
CA74	cys	AA26	Glu	AA13	cys	Lys
CA74	cys	AA29	Ala	AA9	cys	Lys
CA74	cys	Ala	AA29	Arg	cys	Lys
CA74	cys	AA13	Tyr	Gly	cys	Lys
CA74	cys	AA17	AA26	AA13	cys	Lys
CA74	cys	AA17	Tyr	AA13	cys	Lys
CA74	cys	AA23	Asp	Pro	cys	Lys
CA74	cys	AA9	His	Gly	cys	Lys
CA74	cys	AA25	AA10	AA13	cys	Lys
CA74	cys	AA34	Asn	AA1	cys	Lys
CA74	cys	AA23	Phe	Arg	cys	Lys
CA74	cys	His	Glu	AA26	cys	Lys
CA74	cys	Lys	AA18	Arg	cys	Lys
CA74	cys	Phe	AA8	AA16	cys	Lys
CA74	cys	Trp	Arg	AA23	cys	Lys
CA74	cys	Asn	Asn	AA26	cys	Lys
CA74	cys	AA9	Gly	Gly	cys	Lys
CA74	cys	AA9	Lys	AA29	cys	Lys
CA74	cys	AA17	Arg	AA26	cys	Lys
CA74	cys	AA7	Asp	Arg	cys	Lys
CA74	cys	AA20	Glu	AA23	cys	Lys
CA74	cys	AA7	AA24	AA23	cys	Lys
CA74	cys	AA9	AA24	AA26	cys	Lys
CA74	cys	Phe	Ala	AA17	cys	Lys
CA74	cys	AA27	AA1	AA23	cys	Lys
CA74	cys	AA1	AA10	AA24	cys	Lys
CA74	cys	AA28	AA24	AA26	cys	Lys
CA74	cys	Tyr	AA8	AA26	cys	Lys
CA74	cys	Ser	Asp	AA13	cys	Lys
CA50	cys	Phe	AA2	AA16	cys	Lys
CA51	cys	Gly	Glu	AA2	cys	Lys
CA52	cys	AA24	Ala	AA29	cys	Lys
CA55	cys	Glu	Gln	AA26	cys	Lys
CA55	cys	AA30	AA23	AA13	cys	Lys
CA55	cys	AA26	AA8	AA27	cys	Lys
CA62	cys	AA26	Ala	AA29	cys	Lys
CA62	cys	AA26	AA29	Ala	cys	Lys
CA64	cys	AA18	Gly	AA13	cys	Lys
CA64	cys	AA33	Asn	AA27	cys	Lys
CA80	cys	Ala	AA8	AA27	cys	Lys

CA80	cys	Phe	Leu	Pro	cys	Lys
CA80	cys	AA14	AA8	AA27	cys	Lys
CA80	cys	AA27	AA31	AA5	cys	Lys
CA75	cys	AA20	AA20	AA32	cys	Lys
CA107	cys	AA14	AA13	AA23	cys	Lys
CA107	cys	Gln	AA8	AA27	cys	Lys
CA107	cys	Trp	AA8	AA27	cys	Lys
CA107	cys	AA14	AA23	AA13	cys	Lys
CA107	cys	AA23	AA23	AA13	cys	Lys
CA107	cys	AA20	AA8	AA27	cys	Lys
CA107	cys	Tyr	AA23	AA13	cys	Lys
CA107	cys	Gln	AA17	Trp	cys	Lys
CA107	cys	AA23	AA13	AA23	cys	Lys
CA107	cys	AA23	AA14	AA13	cys	Lys
CA107	cys	AA13	AA14	AA23	cys	Lys
CA107	cys	AA10	AA8	AA27	cys	Lys
CA107	cys	AA26	AA8	AA27	cys	Lys
CA107	cys	AA20	Ala	AA26	cys	Lys
CA107	cys	AA2	AA8	AA27	cys	Lys
CA107	cys	AA30	AA8	AA27	cys	Lys
CA3	cys	AA17	AA31	AA2	cys	Lys
CA3	cys	Tyr	AA8	AA27	cys	Lys
CA3	cys	AA30	AA23	Pro	cys	Lys
CA10	cys	AA7	Lys	AA16	cys	Lys
CA15	cys	AA21	His	AA23	cys	Lys
CA18	cys	Asp	AA8	AA16	cys	Lys
CA118	cys	AA14	AA8	AA27	cys	Lys
CA24	cys	AA24	Ala	Gly	cys	Lys
CA26	cys	Asp	AA8	AA16	cys	Lys
CA28	cys	AA34	AA10	AA26	cys	Lys
CA43	cys	AA23	AA2	Pro	cys	Lys
CA29	cys	AA17	AA30	AA2	cys	Lys
CA30	cys	Tyr	AA8	AA27	cys	Lys
CA30	cys	AA17	AA8	AA27	cys	Lys
CA30	cys	AA31	AA8	AA27	cys	Lys
CA30	cys	AA17	Trp	AA17	cys	Lys
CA30	cys	AA16	AA23	AA16	cys	Lys
CA30	cys	AA24	AA20	AA16	cys	Lys
CA30	cys	AA2	AA17	Trp	cys	Lys
CA30	cys	Gln	Tyr	AA16	cys	Lys
CA30	cys	AA13	AA17	Trp	cys	Lys
CA30	cys	AA23	AA8	Pro	cys	Lys
CA30	cys	AA32	Ser	AA2	cys	Lys

CA30	cys	AA9	AA8	AA27	cys	Lys
CA30	cys	AA29	AA8	AA27	cys	Lys
CA30	cys	Trp	AA10	AA24	cys	Lys
CA30	cys	AA27	AA8	AA27	cys	Lys
CA30	cys	AA2	AA10	AA24	cys	Lys
CA30	cys	AA2	AA8	AA27	cys	Lys
CA30	cys	Phe	AA26	AA2	cys	Lys
CA30	cys	AA14	AA8	AA27	cys	Lys
CA30	cys	Pro	AA17	Trp	cys	Lys
CA30	cys	AA2	Trp	AA17	cys	Lys
CA30	cys	AA15	Gln	AA16	cys	Lys
CA31	cys	Asp	AA8	AA16	cys	Lys
CA31	cys	Phe	Gly	AA2	cys	Lys
CA31	cys	Phe	AA17	AA26	cys	Lys
CA31	cys	Gly	Phe	AA2	cys	Lys
CA31	cys	AA11	AA8	AA27	cys	Lys
CA33	cys	AA31	AA8	AA27	cys	Lys
CA36	cys	Ala	Gly	AA16	cys	Lys
CA36	cys	AA8	AA27	AA2	cys	Lys
CA73	cys	Ala	Gly	AA16	cys	Lys
CA78	cys	AA6	AA8	AA27	cys	Lys
CA76	cys	Tyr	AA8	AA27	cys	Lys
CA87	cys	AA26	AA20	AA16	cys	Lys
CA88	cys	Ser	AA8	AA27	cys	Lys
CA88	cys	Pro	Phe	AA16	cys	Lys
CA88	cys	AA17	AA23	AA26	cys	Lys
CA88	cys	Phe	AA20	AA27	cys	Lys
CA88	cys	Thr	Gln	AA16	cys	Lys
CA88	cys	AA30	AA23	AA16	cys	Lys
CA88	cys	AA17	AA14	Pro	cys	Lys
CA88	cys	AA17	AA26	AA23	cys	Lys
CA88	cys	Phe	Gly	AA2	cys	Lys
CA88	cys	AA30	Asn	AA26	cys	Lys
CA88	cys	Gly	Arg	AA2	cys	Lys
CA89	cys	Trp	Ala	AA2	cys	Lys
CA89	cys	Pro	AA17	Trp	cys	Lys
CA89	cys	Asp	AA8	AA16	cys	Lys
CA89	cys	AA14	AA17	Trp	cys	Lys
CA89	cys	Asp	AA8	AA27	cys	Lys
CA89	cys	AA7	Tyr	AA2	cys	Lys
CA89	cys	AA26	AA8	AA27	cys	Lys
CA89	cys	AA14	AA8	AA27	cys	Lys
CA89	cys	Ala	AA8	AA27	cys	Lys

CA89	cys	AA2	AA8	AA27	cys	Lys
CA89	cys	Asn	AA8	AA27	cys	Lys
CA89	cys	AA20	AA26	AA16	cys	Lys
CA89	cys	Phe	AA8	AA27	cys	Lys
CA89	cys	AA20	Glu	AA5	cys	Lys
CA90	cys	AA17	Ala	AA29	cys	Lys
CA90	cys	Asp	AA2	AA16	cys	Lys
CA94	cys	AA26	AA8	AA27	cys	Lys
CA95	cys	Gly	Ala	AA16	cys	Lys
CA95	cys	AA29	AA8	AA27	cys	Lys
CA120	cys	Asp	AA8	AA16	cys	Lys
CA100	cys	Thr	AA29	AA16	cys	Lys
CA101	cys	Phe	Leu	Pro	cys	Lys
CA101	cys	Ser	AA8	AA27	cys	Lys
CA101	cys	Leu	Phe	Pro	cys	Lys
CA101	cys	Phe	Gly	AA2	cys	Lys
CA101	cys	AA30	Gly	Gly	cys	Lys
CA101	cys	Ala	AA17	Pro	cys	Lys
CA110	cys	AA17	AA31	Gln	cys	Lys
CA124	cys	Arg	AA29	AA2	cys	Lys
CA124	cys	Gln	AA31	Asp	cys	Lys
CA105	cys	AA10	Trp	AA17	cys	Lys
CA114	cys	Phe	AA8	AA27	cys	Lys
CA114	cys	AA19	Gly	AA2	cys	Lys
CA114	cys	AA23	Asn	AA1	cys	Lys
CA114	cys	Phe	Asn	Pro	cys	Lys
CA114	cys	Asn	Phe	AA23	cys	Lys
CA114	cys	Glu	AA2	AA16	cys	Lys
CA114	cys	Phe	Asn	AA23	cys	Lys
CA114	cys	AA23	AA1	Asn	cys	Lys
CA114	cys	AA2	AA8	AA27	cys	Lys
CA114	cys	AA30	AA8	AA27	cys	Lys
CA114	cys	AA31	AA8	AA27	cys	Lys
CA114	cys	Asp	AA8	AA16	cys	Lys
CA114	cys	Ala	AA8	AA27	cys	Lys
CA114	cys	AA29	AA8	AA27	cys	Lys
CA114	cys	AA2	AA10	AA24	cys	Lys
CA114	cys	AA10	AA8	AA27	cys	Lys
CA114	cys	AA20	AA27	AA5	cys	Lys
CA114	cys	AA10	Trp	AA17	cys	Lys
CA114	cys	AA17	AA8	AA27	cys	Lys
CA114	cys	AA27	AA8	AA27	cys	Lys
CA114	cys	Tyr	Gln	AA16	cys	Lys

CA114	cys	AA26	AA8	AA27	cys	Lys
CA114	cys	Phe	AA26	AA16	cys	Lys
CA114	cys	AA26	AA9	AA2	cys	Lys
CA114	cys	Pro	AA8	AA27	cys	Lys
CA114	cys	AA17	Trp	AA17	cys	Lys
CA114	cys	Phe	Gly	AA5	cys	Lys
CA114	cys	AA21	Gly	AA2	cys	Lys
CA114	cys	Gly	AA8	AA27	cys	Lys
CA111	cys	AA31	AA8	AA27	cys	Lys
CA111	cys	Gly	Ala	AA16	cys	Lys
CA111	cys	Pro	AA8	AA27	cys	Lys
CA128	cys	Ala	AA8	AA27	cys	Lys
CA128	cys	Lys	AA26	AA20	cys	Lys
CA128	cys	Arg	AA29	AA26	cys	Lys

7.3 Post-sequencing multi-stage manual triage (with examples)

After an affinity selection, a post-sequencing multi-stage manual triage was conducted to isolate true positive hits:

- Sequence Filtering: Proposed candidates were first filtered to ensure alignment with the library design scaffold (XCXXXCK), where C represents fixed cysteines and K a fixed C-terminal lysine.
- Chromatographic Validation: For each candidate, an Extracted Ion Chromatogram (XIC) was generated from the raw data to confirm the presence of a well-defined peak with a characteristic chromatographic shape at the expected retention time.
- Differential Specificity Analysis: To eliminate non-specific binders (e.g., highly hydrophobic peptides), the exact mass of each candidate was searched in the control sample raw data. A candidate was only qualified as a "true hit" for re-synthesis if a distinct peak was detected in the target sample while being absent at the corresponding retention time in the control.

Example 1:

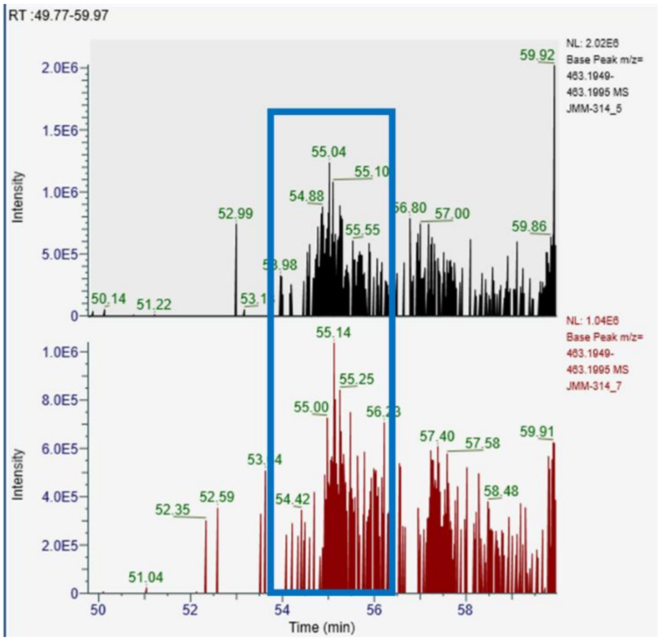

Candidate: A(+84.00)CFA(+77.03)A(+52.03)CK(-0.98)
ALC (%): 97.2
m/z: 463.19717
RT: 55.0462

Exclusion criteria: no unique peak for sample over control.

Example 2:

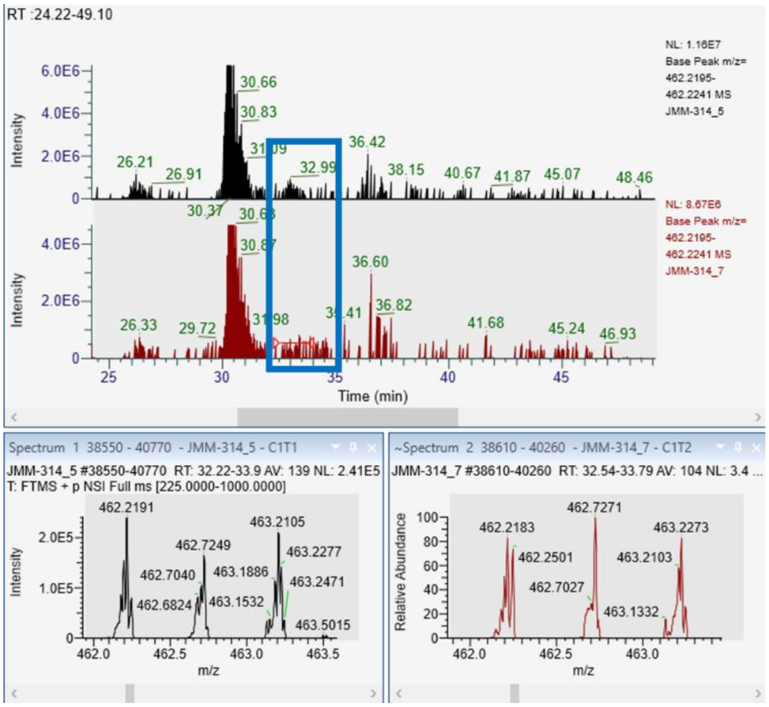

Candidate:
A(+90.02)CA(+48.00)A(+40.03)A(+109.09)CK (-0.98)
ALC (%): 92.9
m/z: 462.22177
RT: 32.9913

Exclusion criteria: no unique peak for sample over control. Isotope patterns shown to avoid nuance.

Example 3:

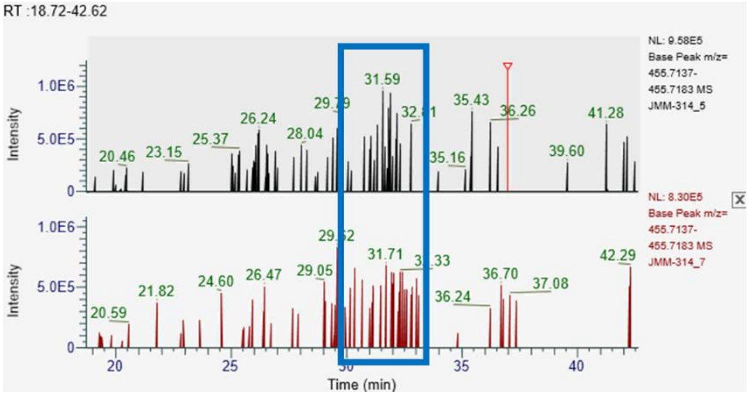

Candidate:
A(+91.03)CA(+54.05)A(+77.03)A(+52.03)CK(-0.98)
ALC (%): 92.7
m/z: 455.71603
RT: 31.8541

Exclusion criteria: no unique peak for sample over control.

Example 4:

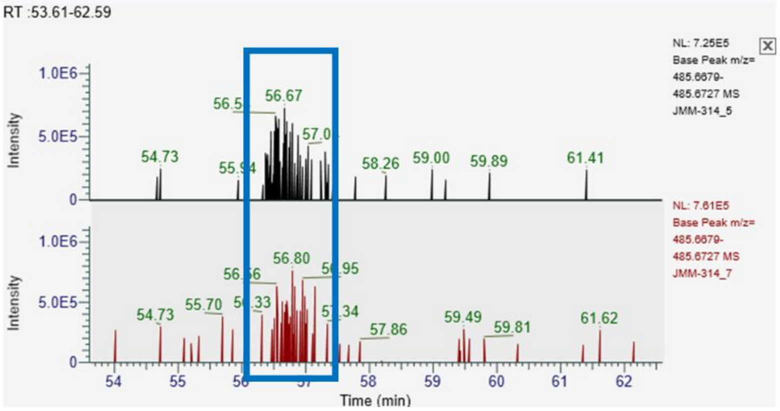

Candidate:
A(+78.95)CA(+126.03)A(+77.03)A(+52.03)CK
(-0.98)
ALC (%): 92.3
m/z: 485.67026
RT: 56.4878

Exclusion criteria: no unique peak for sample over control.

Example 5:

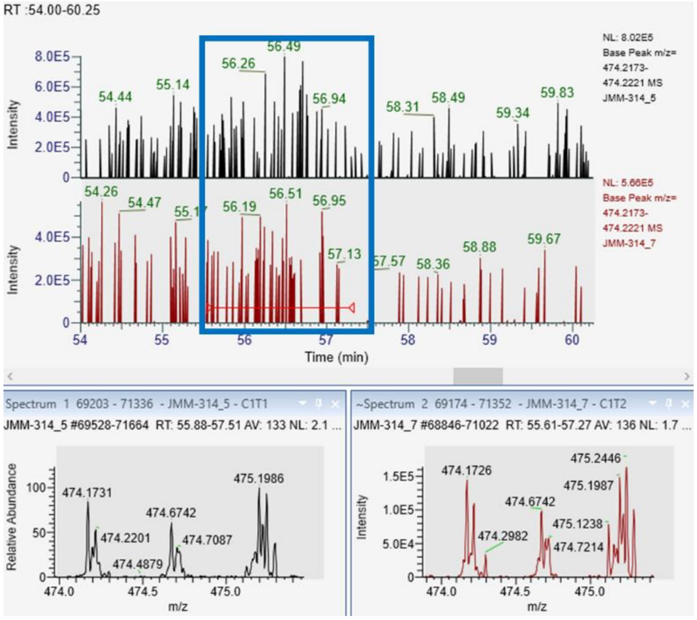

Candidate:
A(+100.00)CA(+77.03)A(+52.03)A(+82.08)CK
(-0.98)
ALC (%): 85.6
m/z: 474.2197
RT: 56.3887

Exclusion criteria: no unique peak for sample over control. Isotope patterns shown to avoid nuance.

Example 6:

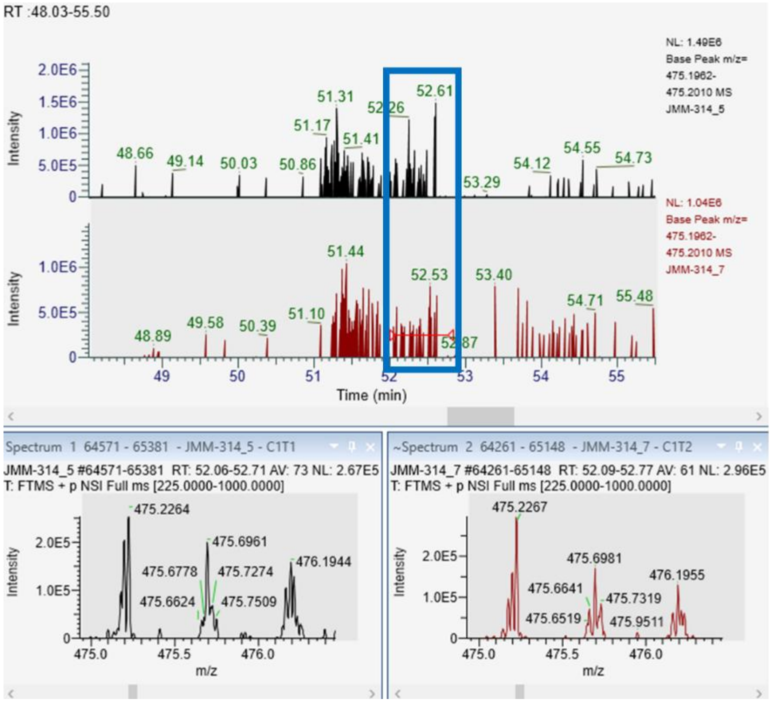

Candidate:
A(+84.94)CFA(+82.08)A(+70.04)CK(-0.98)
ALC (%): 80.1
m/z: 475.19861
RT: 52.395

Exclusion criteria: no unique peak for sample over control. Isotope patterns shown to avoid nuance.

7.4 Enrichment and p-value calculations for CAIX selection

True hit rate calculation: (number of sequences bearing CA74)/(number of enriched hits after background and control subtraction)*100.

Enrichment calculation: The enrichment can be calculated as follows: [(# found binders)/(# compounds after selection)]:[(# total binders)/(# total library)]. Given that actual total number of binders presents in the library is unknown, usually the maximum enrichment is calculated, postulating the number of found binders equal to the total number of binders[12].

p-value calculation: calculated using the following Python script:

```
####### CycloSEL-16M #######

library_size = 130 * 50 * 50 * 50

strcts_w_substrct = 1 * 50 * 50 * 50

compounds_after_selection = 330

found_binders = 176


structs_wo_substrct = library_size - strcts_w_substrct

nonHits_w_substrct = strcts_w_substrct - found_binders

hits_wo_substrct = compounds_after_selection - found_binders

nonHits_wo_subsrct = library_size - compounds_after_selection -
nonHits_w_substrct


data = pd.DataFrame({

    "sulfonamide containing structures": [found_binders, nonHits_w_substrct],

    "structures without sulfonamide": [hits_wo_substrct, nonHits_wo_subsrct]

}, index=["After AS-MS", "Before AS-MS"])



odds_ratio, p_value = fisher_exact(data)


print("P-value:", p_value
```

7.5 Hit selection and manual inspection

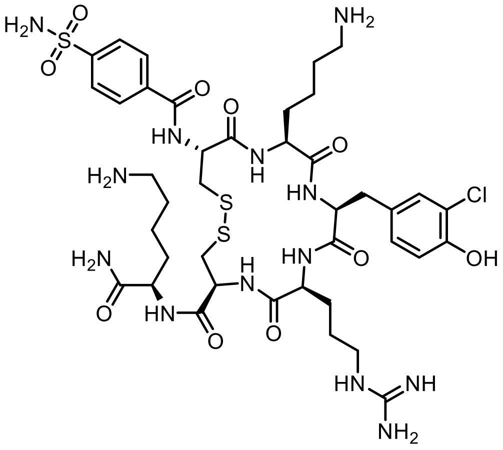

Isotope pattern for RT 28-29min:

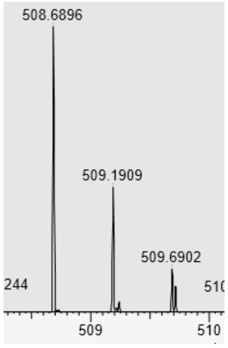

PEAKS 11 de novo sequencing output:

Peptide	Length	ALC (%)	m/z	z	RT
A(+111.96)CKA(+125.99)RCK(-0.98)	7	97.1	508.6888	2	28.153

Manual base peak query m/z = 508.689:

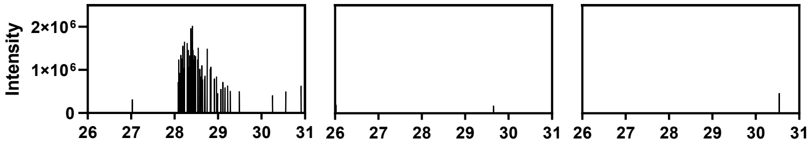

CAIX + linearization step

CAIX without linearization step

Streptavidin + linearization step

Fragmentation fingerprint:

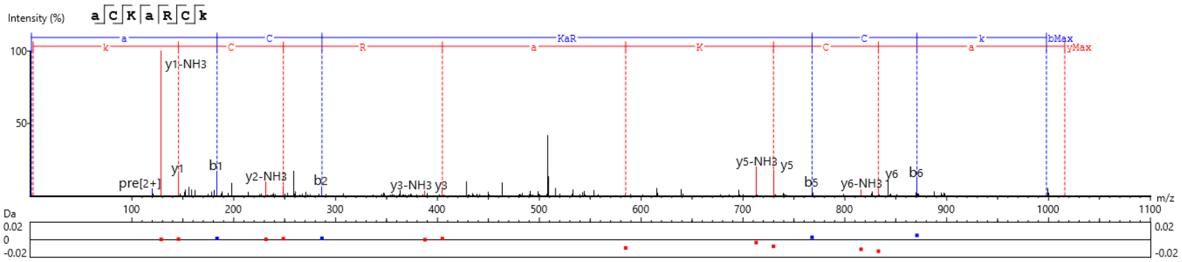

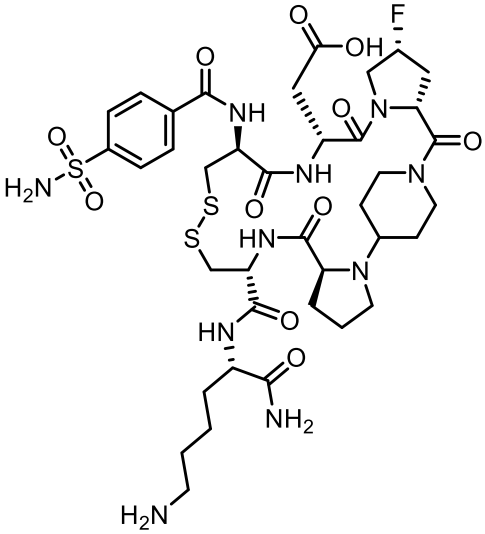

PEAKS 11 de novo sequencing output:

Peptide	ALC (%)	Length	m/z	z	RT
A(+111.96)CDA(+44.01)A(+109.09)CK(-0.98)	94.6	7	473.1713	2	28.3008

Manual base peak query m/z = 508.689:

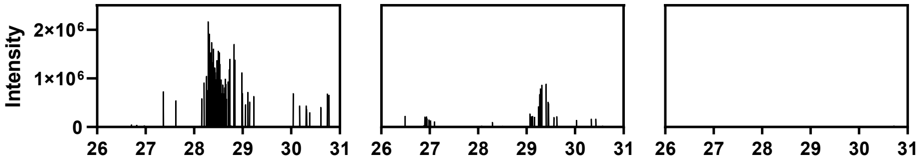

CAIX + linearization step

CAIX without linearization step

Streptavidin + linearization step

Isotope pattern for RT 28-29min:

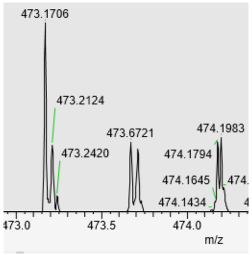

Fragmentation fingerprint:

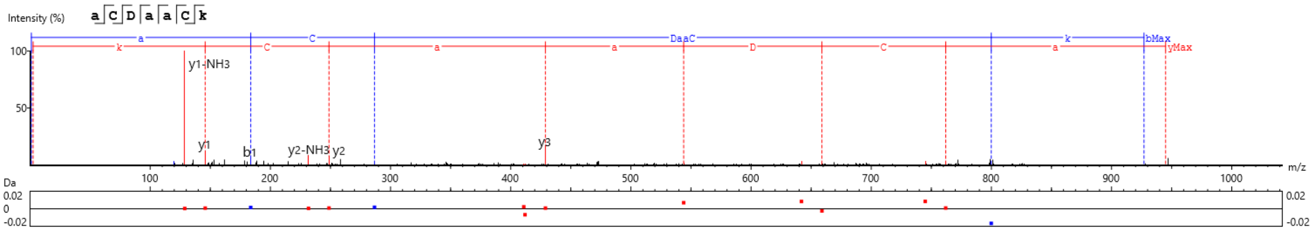

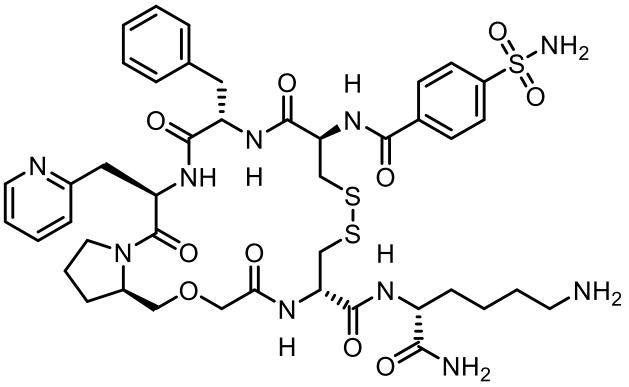

PEAKS 11 de novo sequencing output:

Peptide	ALC (%)	Length	m/z	z	RT
A(+111.96)CFA(+77.03)A(+70.04)CK(-0.98)	94.5	7	486.1803	2	27.4571

Manual base peak query m/z = 486.311:

Isotope pattern for RT 27-28min:

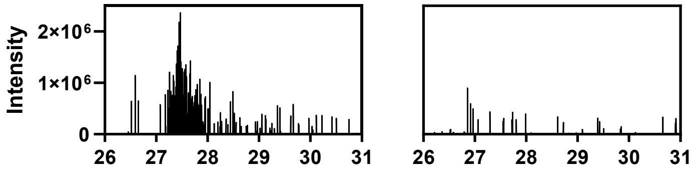

CAIX + linearization step

Streptavidin + linearization step

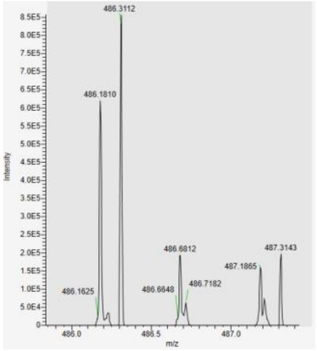

Fragmentation fingerprint:

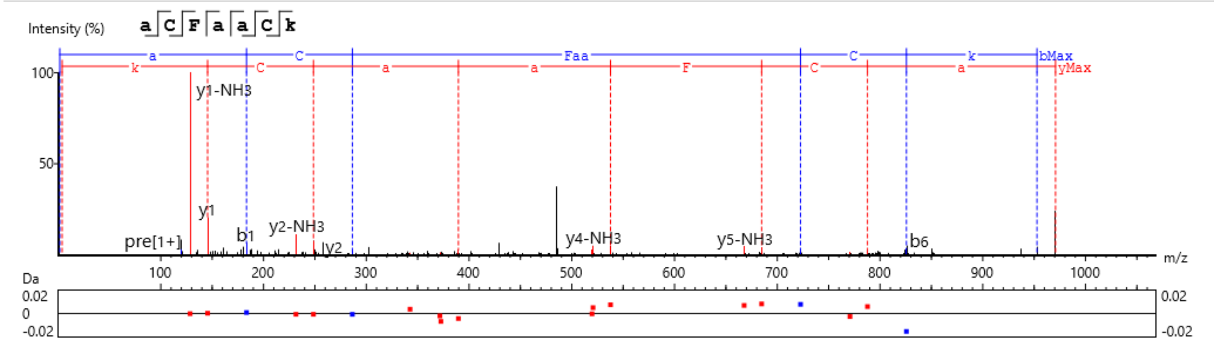

8. Affinity Selection against WDR5

8.1 Procedure

MyOne Streptavidin T1 Dynabeads (100 µL of 10 mg/mL stock per well, measured in duplicates) were washed with 3x 1 mL 10% FBS, 20 mM Tris, 500 mM NaCl pH8, 0.02% Tween-20. The beads were incubated with biotinylated WDR5 (100 µL, 1.5 µM per well) in 10% FBS, 20 mM Tris, 500 mM NaCl pH8, 0.02% Tween-20 for 1h at 4°C. The beads were washed 2x 1 mL 10% FBS, 20 mM Tris, 500 mM NaCl pH8, 0.02% Tween20, 400 µM d-biotin and 1x 1 mL 10% FBS, 20 mM Tris, 500 mM NaCl pH8, 0.02% Tween-20 before incubating with the library (1000 µL, 10 fmol/member per well) in 10% FBS, 20 mM Tris, 500 mM NaCl pH8 for 1h at 4°C. The beads were washed with 5x 1 mL of 20 mM Tris, 500 mM NaCl pH8 and subsequently eluted with 2x 6M Guanidine HCl in 20mM phosphate buffer pH 6.8 supplemented with 10 mM TCEP HCl (100 µL per well).

Sample preparation after AS: The StageTips were prepared as described by Rappsilber et al. using C18 material from Empore SPE 47 mm discs (66883-U, Merck) (46). The StageTips were pre-conditioned with 100 µL MeOH, 100 µL of 0.1% (v/v) FA in ACN and 100 µL of 0.1% (v/v) FA in MQ, respectively by centrifuging for 3 min at 300 rpm. The samples were then loaded on the StageTips and washed with 100 µL of 0.1% (v/v) FA in MQ. Compounds were eluted by adding 100 µL of 0.1% (v/v) FA in ACN:MQ (7:3). The samples were lyophilized before resuspending in 10 µL 0.1% (v/v) FA in UPLC-MS grade water. Afterwards, 9 µL was injected into the LC-MS/MS system.

8.2 List of filtered sequences

Table S6: Filtered sequences of CAIX selection. In yellow are represented that passed manual inspection (clear enrichment XIC peak over background).

CA		X=1	X=2	X=3		
CA116	Cys	AA23	Arg	Trp	Cys	Lys
CA59	Cys	Asn	Asn	AA26	Cys	Lys
CA10	Cys	AA32	Ala	Phe	Cys	Lys
CA107	Cys	Thr	Lys	AA24	Cys	Lys
CA110	Cys	Phe	Gly	Pro	Cys	Lys
CA114	Cys	AA31	AA8	AA27	Cys	Lys
CA114	Cys	AA30	AA8	AA27	Cys	Lys
CA114	Cys	AA2	AA8	AA27	Cys	Lys
CA114	Cys	Phe	AA8	AA27	Cys	Lys
CA114	Cys	AA30	AA17	Trp	Cys	Lys
CA114	Cys	AA30	Trp	AA17	Cys	Lys
CA114	Cys	AA29	AA8	AA27	Cys	Lys
CA114	Cys	AA31	AA17	Trp	Cys	Lys
CA114	Cys	AA10	Trp	AA17	Cys	Lys
CA114	Cys	AA26	AA8	AA27	Cys	Lys
CA114	Cys	Ala	AA8	AA27	Cys	Lys

CA114	Cys	AA17	AA8	AA27	Cys	Lys
CA114	Cys	AA17	Trp	AA17	Cys	Lys
CA114	Cys	Ser	AA10	AA24	Cys	Lys
CA114	Cys	Phe	AA12	AA16	Cys	Lys
CA114	Cys	Ser	AA8	AA27	Cys	Lys
CA118	Cys	AA14	AA8	AA27	Cys	Lys
CA129	Cys	Ser	Ala	AA29	Cys	Lys
CA14	Cys	AA30	Trp	AA17	Cys	Lys
CA19	Cys	Leu	AA30	AA26	Cys	Lys
CA20	Cys	AA30	Ala	Trp	Cys	Lys
CA3	Cys	Phe	AA8	AA27	Cys	Lys
CA30	Cys	AA2	AA8	AA27	Cys	Lys
CA30	Cys	Phe	AA8	AA27	Cys	Lys
CA30	Cys	AA31	AA8	AA27	Cys	Lys
CA30	Cys	AA12	AA8	AA27	Cys	Lys
CA30	Cys	AA14	AA8	AA27	Cys	Lys
CA30	Cys	Trp	Ala	AA2	Cys	Lys
CA31	Cys	AA11	AA8	AA27	Cys	Lys
CA37	Cys	Pro	AA8	AA27	Cys	Lys
CA49	Cys	AA17	Phe	AA26	Cys	Lys
CA49	Cys	AA12	Pro	AA2	Cys	Lys
CA51	Cys	Gln	AA25	AA16	Cys	Lys
CA55	Cys	AA30	AA23	AA13	Cys	Lys
CA73	Cys	Phe	Gly	AA2	Cys	Lys
CA73	Cys	AA30	AA17	AA2	Cys	Lys
CA89	Cys	Phe	AA8	AA27	Cys	Lys
CA89	Cys	Gln	AA8	AA27	Cys	Lys
CA89	Cys	AA2	AA8	AA27	Cys	Lys
CA89	Cys	Tyr	AA10	AA24	Cys	Lys
CA89	Cys	AA10	Asp	Arg	Cys	Lys
CA89	Cys	AA27	AA11	AA16	Cys	Lys
CA9	Cys	Thr	AA31	AA14	Cys	Lys
CA90	Cys	AA1	Gly	AA2	Cys	Lys
CA96	Cys	AA1	AA8	AA16	Cys	Lys
CA99	Cys	Gly	Ala	AA13	Cys	Lys
CA37	Cys	Asn	Asn	AA26	Cys	Lys

8.3 Hit selection and manual inspection

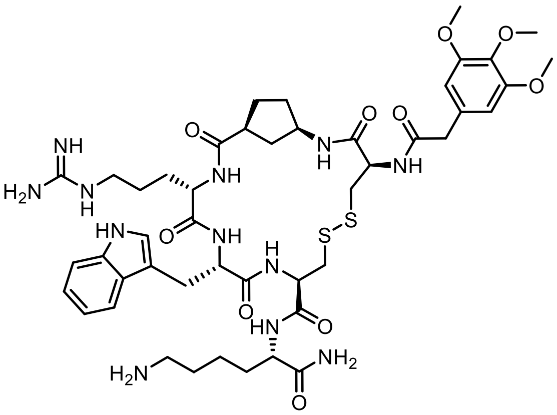

PEAKS 11 de novo sequencing output:

Peptide	ALC (%)	Length	m/z	z	RT
A(+137.04)CA(+40.03)WRCK(-0.98)	86.2	7	507.238	2	31.545

Isotope pattern for RT 31-32min:

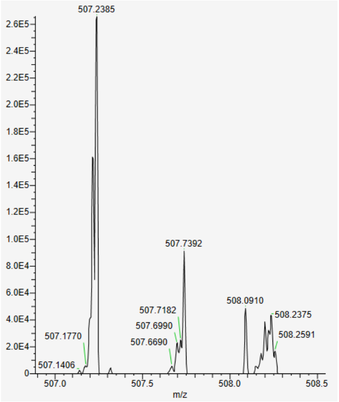

Manual base peak query m/z = 507.238:

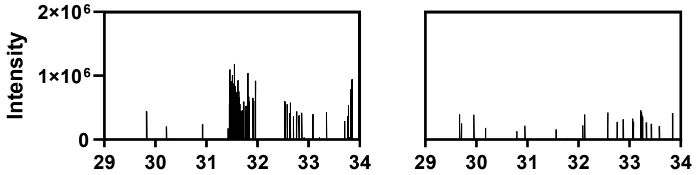

WDR5 + linearization step

Streptavidin + linearization step

Fragmentation fingerprint:

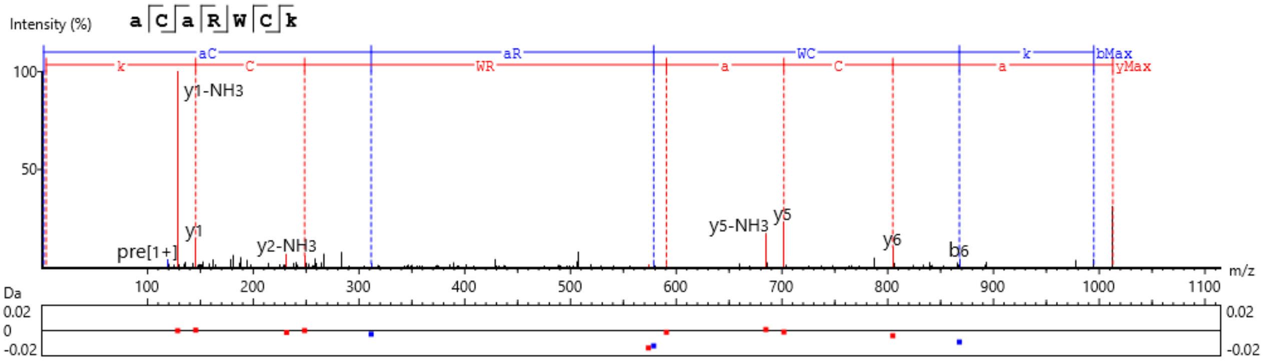

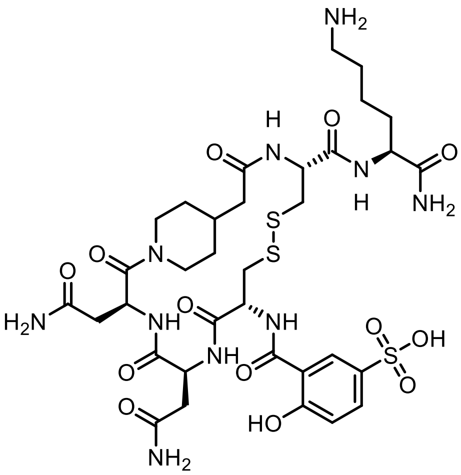

Isotope pattern for RT 32-32min:

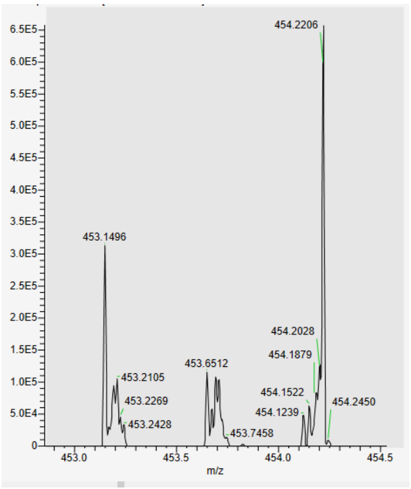

PEAKS 11 de novo sequencing output:

Peptide	ALC (%)	Length	m/z	z	RT
128.94)CNNA(+54.05)CK(-0.98)	85	7	453.149	2	32.894

Manual base peak query m/z = 453.149:

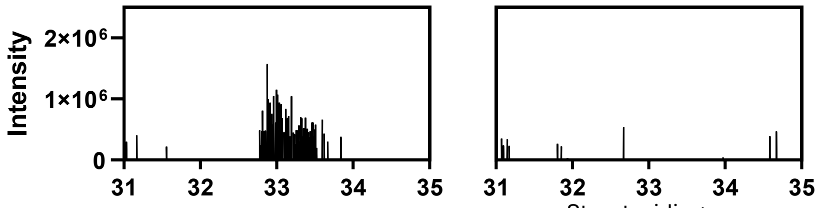

WDR5 + linearization step

Streptavidin + linearization step

Fragmentation fingerprint:

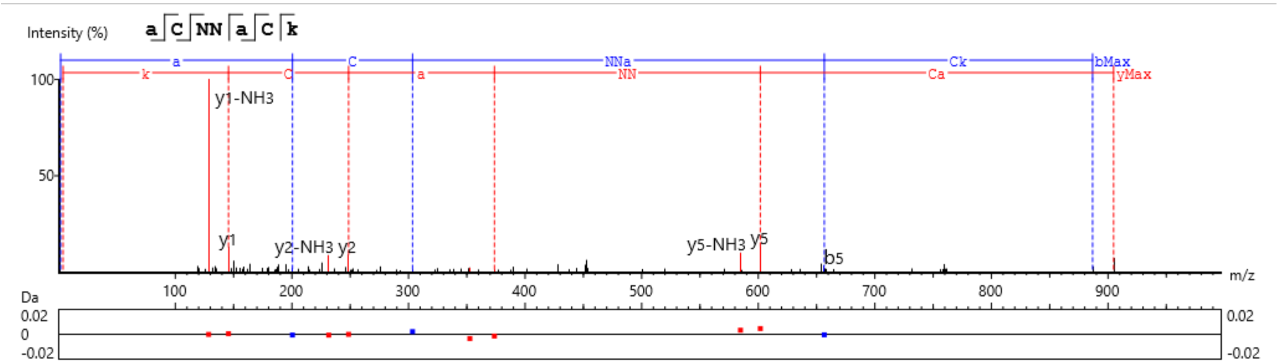

9. Compound information

**Compound:**	**1**
Sequence:	CLAYC (linear)
Structure	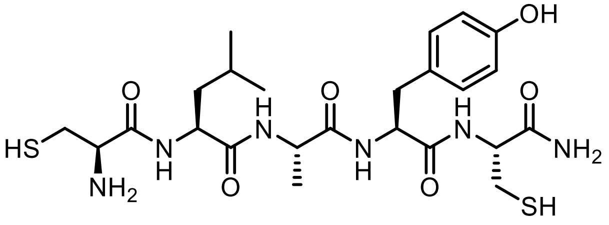
Synthesis:	Automated SPPS
Purification:	used without further purification
HRMS (ESI-QToF) *m/z*:	[M+H]+ calc. 571.237 found 571.232

**Compound:**	**1c**
Sequence:	CLAYC (cyclic)
Structure	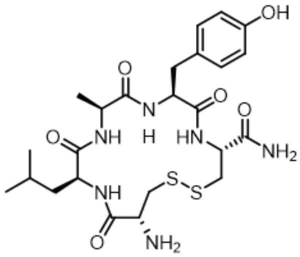
Synthesis:	Automated SPPS
Purification:	used without further purification
HRMS (ESI-QToF) *m/z*:	[M+H]+ calc. 569.222 found 569.217

**Compound:**	**2**
Sequence:	CFHWC (linear)
Structure	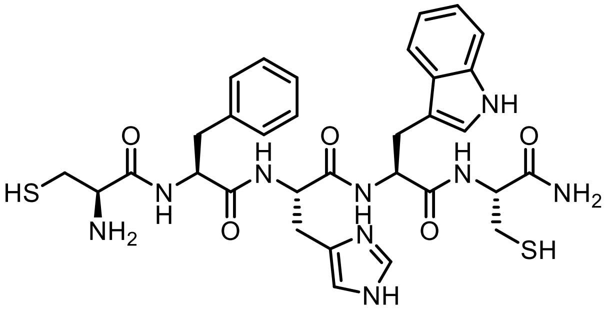
Synthesis:	Automated SPPS
Purification:	used without further purification
HRMS (ESI-QToF) *m/z*:	[M+H]+ calc. 694.258 found 694.241

**Compound:**	**2c**
Sequence:	CFHWC (cyclic)
Structure	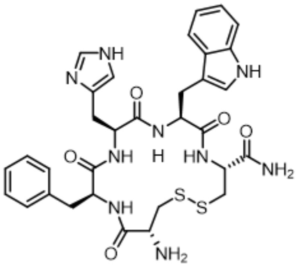
Synthesis:	Automated SPPS
Purification:	used without further purification
HRMS (ESI-QToF) *m/z*:	[M+H]+ calc. calc. 692.243 found 692.227

**Compound:**	**3**
Sequence:	CVNFC (linear)
Structure	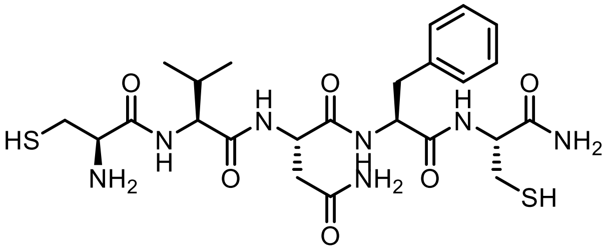
Synthesis:	Automated SPPS
Purification:	used without further purification
HRMS (ESI-QToF) *m/z*:	[M+H]+ calc. calc. 584.209 found 584.228

**Compound:**	**3c**
Sequence:	CVNFC (cyclic)
Structure	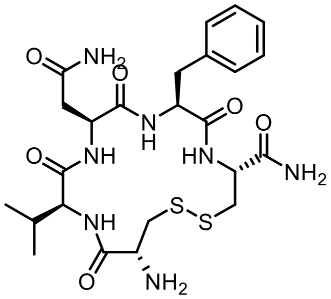
Synthesis:	Automated SPPS
Purification:	used without further purification
HRMS (ESI-QToF) *m/z*:	[M+H]+ calc. calc. 582.216 found 582.201

**Compound:**	**4**
Sequence:	CHEVWC (linear)
Structure	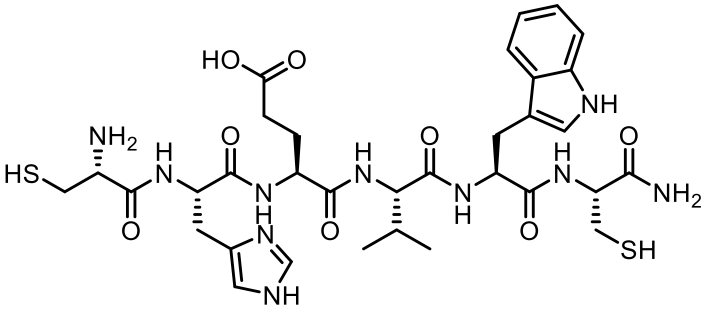
Synthesis:	Automated SPPS
Purification:	used without further purification
HRMS (ESI-QToF) *m/z*:	[M+H]+ calc. calc. 775.301 found 775.283

**Compound:**	**4c**
Sequence:	CHEVWC (cyclic)
Structure	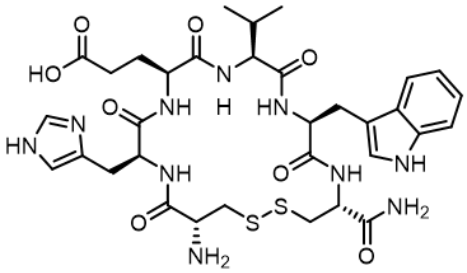
Synthesis:	Automated SPPS
Purification:	used without further purification
HRMS (ESI-QToF) *m/z*:	[M+H]+ calc. calc. 773.288 found 773.266

**Compound:**	**5**
Sequence:	CALYSC (linear)
Structure	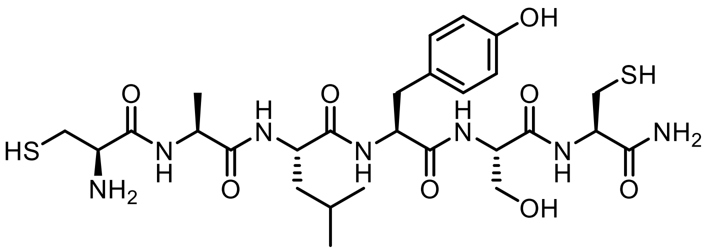
Synthesis:	Automated SPPS
Purification:	used without further purification
HRMS (ESI-QToF) *m/z*:	[M+H]+ calc. calc. 658.269 found 658.254

**Compound:**	**5c**
Sequence:	CALYSC (cyclic)
Structure	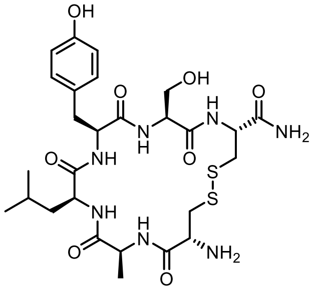
Synthesis:	Automated SPPS
Purification:	used without further purification
HRMS (ESI-QToF) *m/z*:	[M+H]+ calc. calc. 656.253 found 656.239

**Compound:**	**6**
Sequence:	CRITDC (linear)
Structure	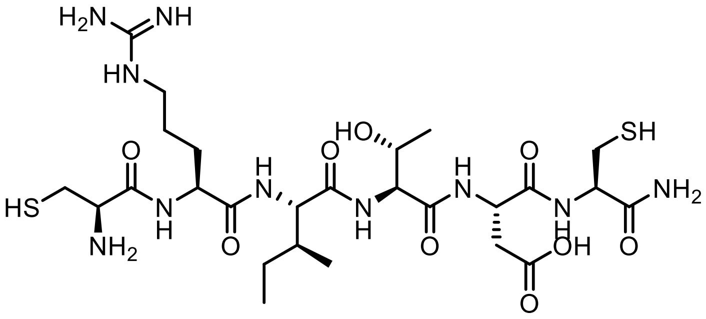
Synthesis:	Automated SPPS
Purification:	used without further purification
HRMS (ESI-QToF) *m/z*:	[M+H]+ calc. calc. 709.312 found 709.311

**Compound:**	**6c**
Sequence:	CRITDC (cyclic)
Structure	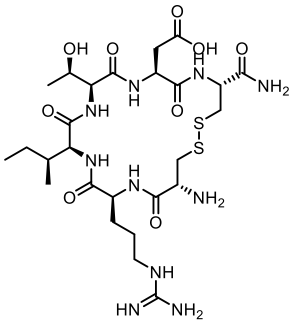
Synthesis:	Automated SPPS
Purification:	used without further purification
HRMS (ESI-QToF) *m/z*:	[M+H]+ calc. calc. 707.296 found 707.296

**Compound:**	**7**
Structure	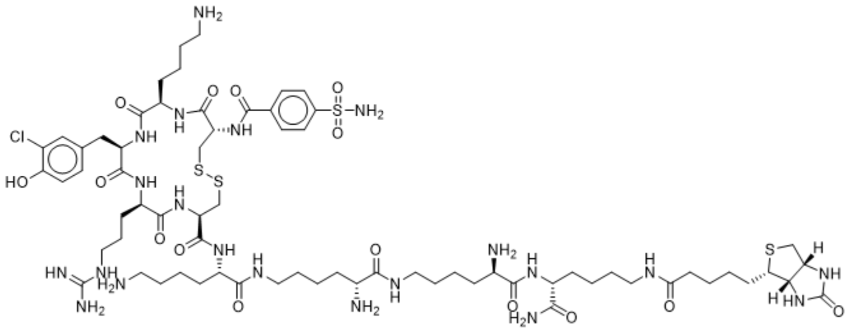
Synthesis:	Manual SPPS
Purification:	Flash chromatography Method A
HRMS (ESI-QToF) *m/z*:	[M+2H]⁺ calc. 812.86 found 812.86

**Compound:**	**8**
Structure	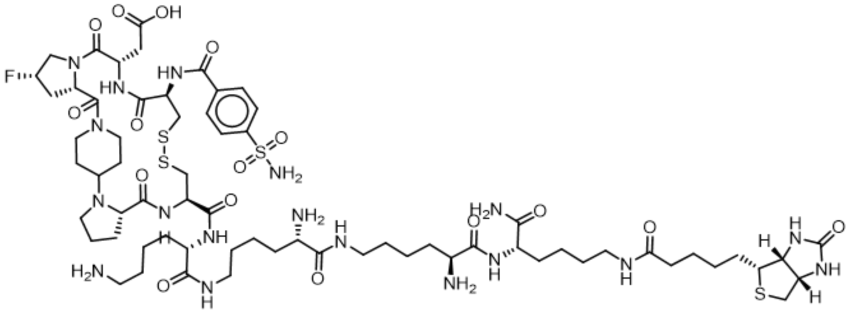
Synthesis:	Manual SPPS
Purification:	Flash chromatography Method A
HRMS (ESI-QToF) *m/z*:	[M+2H]⁺ calc. 777.35 found 777.35

Compound:	9
Structure	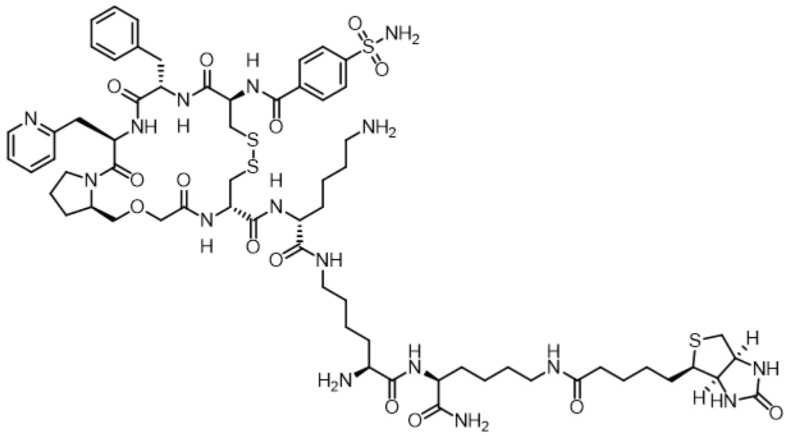
Synthesis:	Manual SPPS
Purification:	Flash chromatography Method A
HRMS (ESI-QToF) *m/z*:	$[M+H]^+$ calc. 1451.11 found 1451.61

**Compound:**	**10**
**Structure**	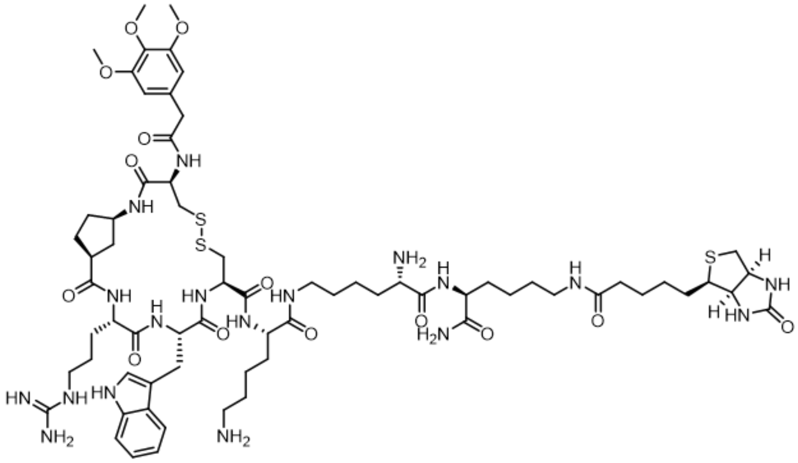
**Synthesis:**	**Manual SPPS**
**Purification:**	**Flash chromatography Method A**
**HRMS (ESI-QToF) *m/z*:**	**$[M+H]^+$ calc. 1493.72 found 1493.72**

**Compound:**	**11**
Structure	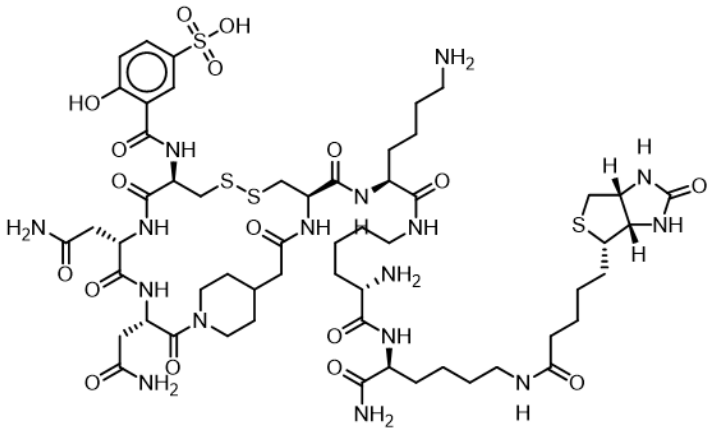
Synthesis:	Manual SPPS
Purification:	Flash chromatography Method A
HRMS (ESI-QToF) *m/z*:	[M+H]$^+$ calc. 1385.55 found 1385.55

**Compound:**	**12**
Structure	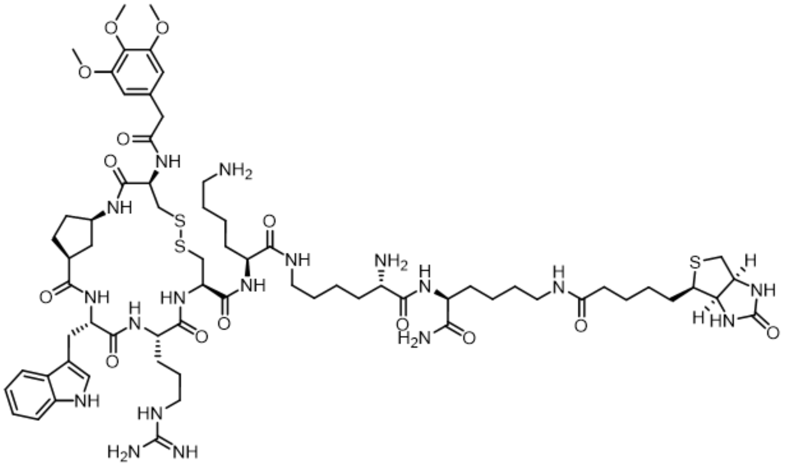
Synthesis:	Manual SPPS
Purification:	Flash chromatography Method A
HRMS (ESI-QToF) *m/z*:	[M+2H]$^+$ calc. 747.36, found 747.36

**Compound:**	**13**
Structure	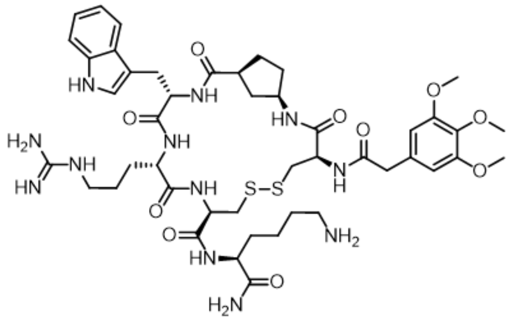
Synthesis:	Manual SPPS
Purification:	Flash chromatography Method A
HRMS (ESI-QToF) *m/z*:	$[M+2H]^+$ calc. 1011.45, found 1011.45

**Compound:**	**14**
Structure	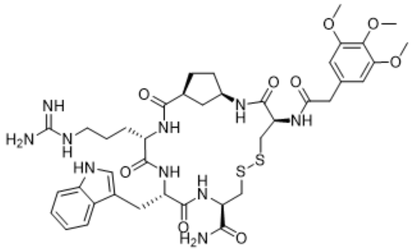
Synthesis:	Manual SPPS
Purification:	Flash chromatography Method A
HRMS (ESI-QToF) *m/z*:	$[M+H]^+$ calc. 883.35, found 883.35

**Compound:**	**15**
Structure	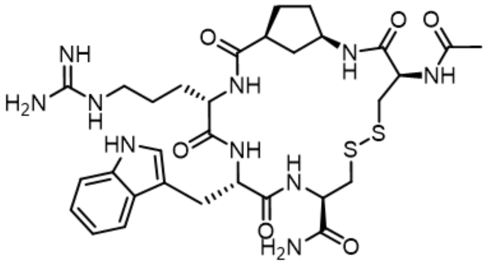
Synthesis:	Manual SPPS
Purification:	Flash chromatography Method A
HRMS (ESI-QToF) *m/z*:	$[M+H]^+$ calc. 717.29, found 717.30

**Compound:**	**16**
Sequence:	Ac-ARAEVHLRKSAFD-N,Lys-Lys(Bio)-CONH2
Structure	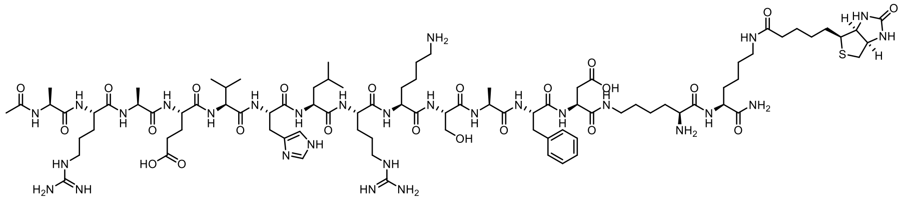
Synthesis:	Manual SPPS
Purification:	Flash chromatography Method A
HRMS (ESI-QToF) *m/z*:	$[M+H]^+$ calc. 1020.45, found 1020.45

**Compound:**	**17**
Structure	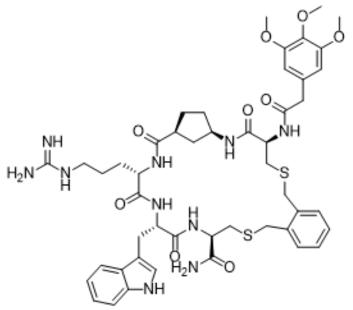
Synthesis:	Manual SPPS
Purification:	Flash chromatography Method A
HRMS (ESI-QToF) *m/z*:	$[M+H]^+$ calc. 987.41, found 987.42

**Compound:**	**18**
Structure	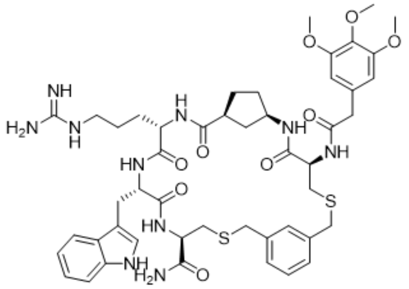
Synthesis:	Manual SPPS
Purification:	Flash chromatography Method A
HRMS (ESI-QToF) *m/z*:	$[M+H]^+$ calc. 987.42, found 987.42

**Compound:**	**19**
Structure	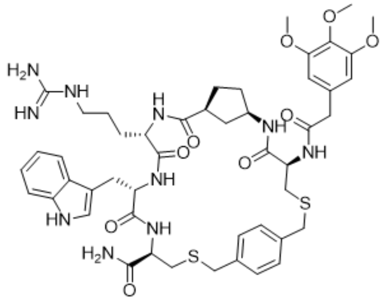
Synthesis:	Manual SPPS
Purification:	Flash chromatography Method A
HRMS (ESI-QToF) *m/z*:	$[M+H]^+$ calc. 987.42, found 987.42

**Compound:**	**20**
Structure	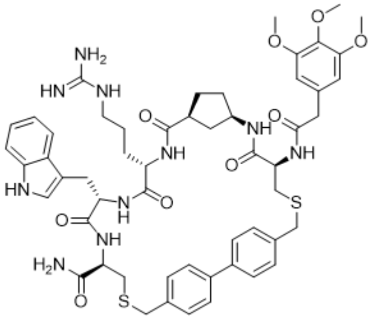
Synthesis:	Manual SPPS
Purification:	Flash chromatography Method A
HRMS (ESI-QToF) *m/z*:	$[M+H]^+$ calc. 1063.45, found 1063.45

**Compound:**	**17Δ**
Structure	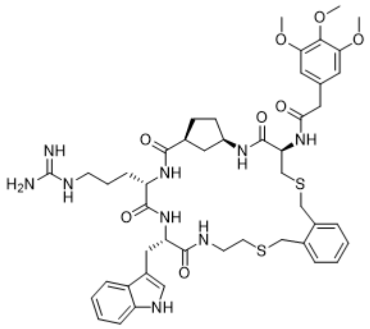
Synthesis:	Manual SPPS
Purification:	Flash chromatography Method A
HRMS (ESI-QToF) *m/z*:	$M+H]^+$ calc. 944.41, found 944.41

**Compound:**	**18Δ**
Structure	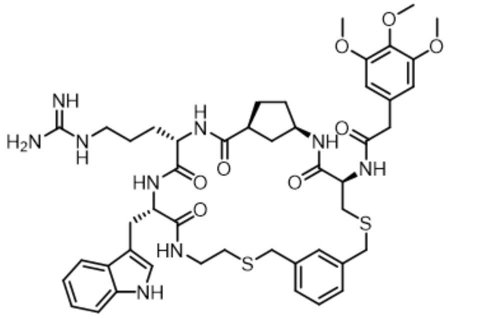
Synthesis:	Manual SPPS
Purification:	Flash chromatography Method A
HRMS (ESI-QToF) *m/z*:	$[M+H]^+$ calc. 944.42, found 944.42

**Compound:**	**19Δ**
Structure	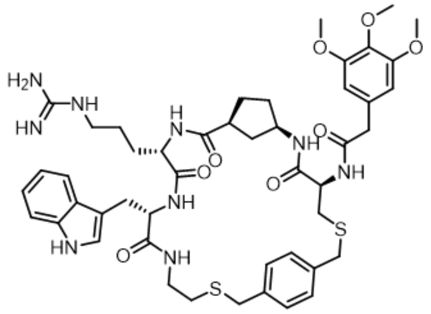
Synthesis:	Manual SPPS
Purification:	Flash chromatography Method A
HRMS (ESI-QToF) *m/z*:	$[M+H]^+$ calc. 944.42, found 944.42

**Compound:**	**20Δ**
Structure	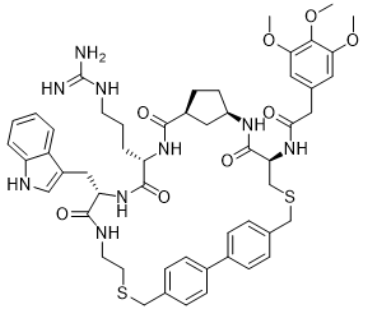
Synthesis:	Manual SPPS
Purification:	Flash chromatography Method A
HRMS (ESI-QToF) *m/z*:	$[M+H]^+$ calc. 1020.45, found 1020.45

**Compound:**	**21**
Structure	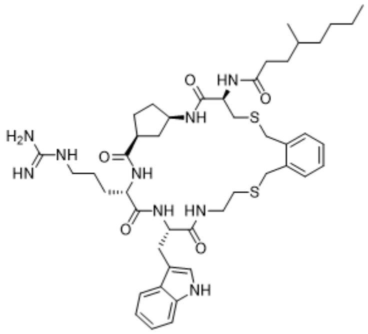
Synthesis:	Manual SPPS
Purification:	Flash chromatography Method A
HRMS (ESI-QToF) *m/z*:	$[M+H]^+$ calc. 876.46, found 876.46

**Compound:**	**22**
Structure	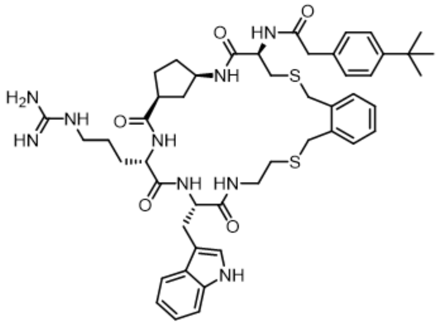
Synthesis:	Manual SPPS
Purification:	Flash chromatography Method A
HRMS (ESI-QToF) *m/z*:	$[M+H]^+$ calc. 910.45, found 910.45

**Compound:**	**23**
Structure	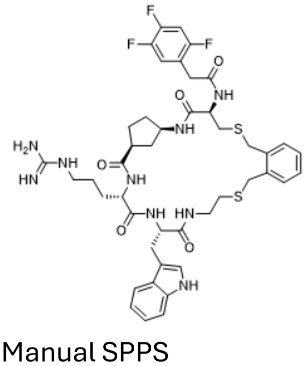
Synthesis:	Manual SPPS
Purification:	Flash chromatography Method A
HRMS (ESI-QToF) *m/z*:	$[M+H]^+$ calc. 908.36, found 908.35

**Compound:**	**24**
Structure	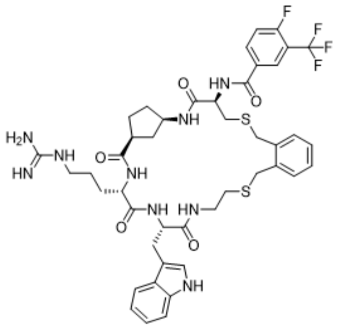
Synthesis:	Manual SPPS
Purification:	Flash chromatography Method A
HRMS (ESI-QToF) *m/z*:	$[M+H]^+$ calc. 926.35, found 926.35

**Compound:**	**25**
Structure	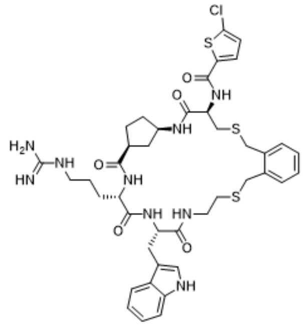
Synthesis:	Manual SPPS
Purification:	Flash chromatography Method A
HRMS (ESI-QToF) *m/z*:	$[M+H]^+$ calc. 880.29, found 880.28

**Compound:**	**26**
Structure	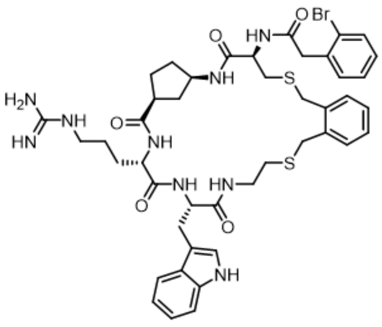
Synthesis:	Manual SPPS
Purification:	Flash chromatography Method A
HRMS (ESI-QToF) *m/z*:	$[M+H]^+$ calc. 932.29, found 932.29

**Compound:**	**27**
Structure	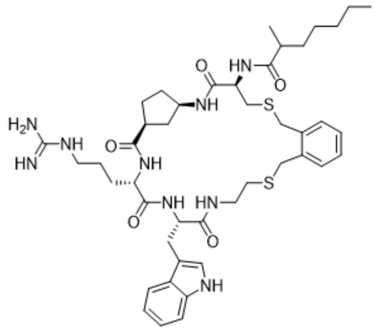
Synthesis:	Manual SPPS
Purification:	Flash chromatography Method A
HRMS (ESI-QToF) *m/z*:	$[M+H]^+$ calc. 862.45, found 862.45

**Compound:**	**28**
Structure	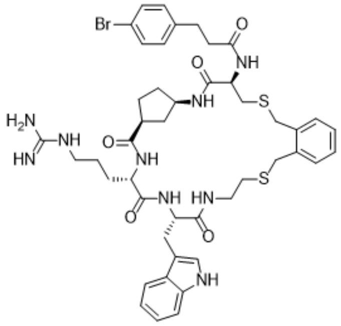
Synthesis:	Manual SPPS
Purification:	Flash chromatography Method A
HRMS (ESI-QToF) *m/z*:	$[M+H]^+$ calc. 946.31, found 946.31

**Compound:**	**29**
Structure	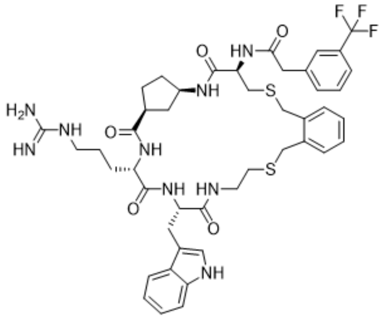
Synthesis:	Manual SPPS
Purification:	Flash chromatography Method A
HRMS (ESI-QToF) *m/z*:	$[M+H]^+$ calc. 922.37, found 922.37

**Compound:**	**30**
Structure	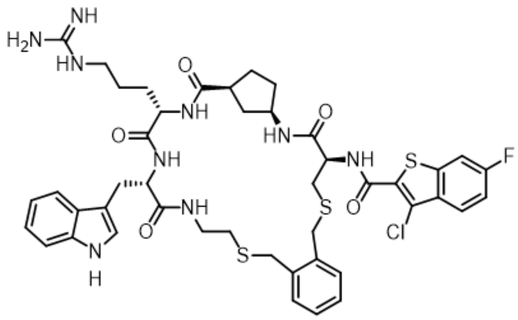
Synthesis:	Manual SPPS
Purification:	Flash chromatography Method A
HRMS (ESI-QToF) *m/z*:	$[M+H]^+$ calc. 948.29, found 948.29

**Compound:**	**s1**
Structure	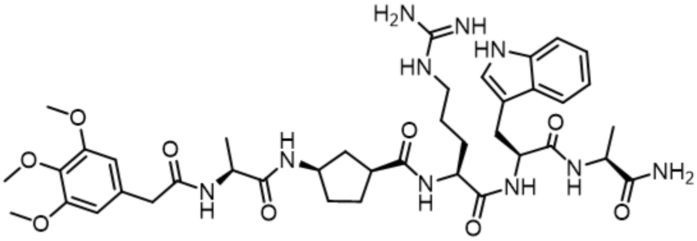
Synthesis:	Manual SPPS
Purification:	Flash chromatography Method A
HRMS (ESI-QToF) *m/z*:	$[M+H]^+$ calc. 821.43, found 821.43

**Compound:**	**s2**
Structure	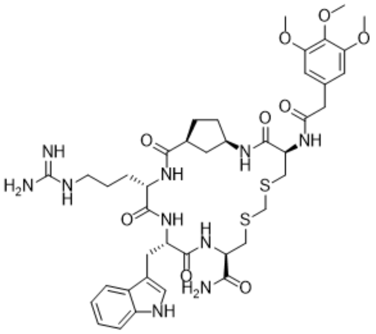
Synthesis:	Manual SPPS
Purification:	Flash chromatography Method A
HRMS (ESI-QToF) *m/z*:	$[M+H]^+$ calc. 897.37, found 897.37

**Compound:**	**s3**
Structure	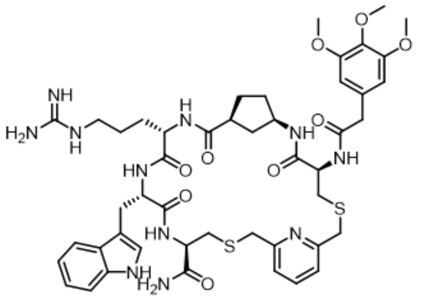
Synthesis:	Manual SPPS
Purification:	Flash chromatography Method A
HRMS (ESI-QToF) *m/z*:	$[M+H]^+$ calc. 988.42, found 988.42

**Compound:**	**s4**
Structure	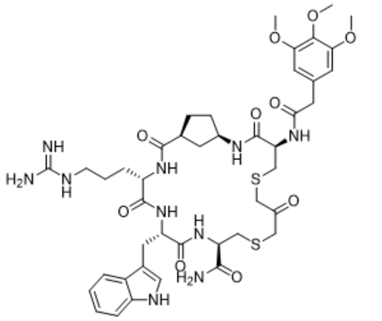
Synthesis:	Manual SPPS
Purification:	Flash chromatography Method A
HRMS (ESI-QToF) *m/z*:	$[M+H]^+$ calc. 939.38, found 939.38

**Compound:**	**s5**
Structure	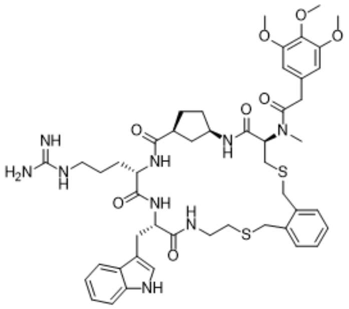
Synthesis:	Manual SPPS
Purification:	Flash chromatography Method A
HRMS (ESI-QToF) *m/z*:	$[M+H]^+$ calc. 958.43, found 958.43

**Compound:**	**s6**
Structure	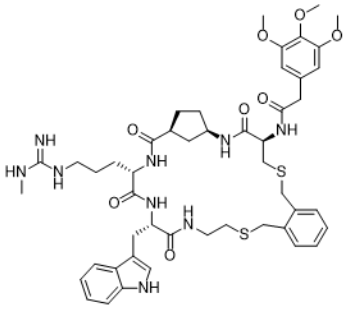
Synthesis:	Manual SPPS
Purification:	Flash chromatography Method A
HRMS (ESI-QToF) *m/z*:	$[M+H]^+$ calc. 958.43, found 958.43

**Compound:**	**s7**
Structure	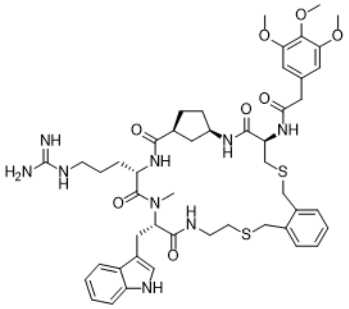
Synthesis:	Manual SPPS
Purification:	Flash chromatography Method A
HRMS (ESI-QToF) *m/z*:	$[M+H]^+$ calc. 958.43, found 958.44

**Compound:**	**s8**
Structure	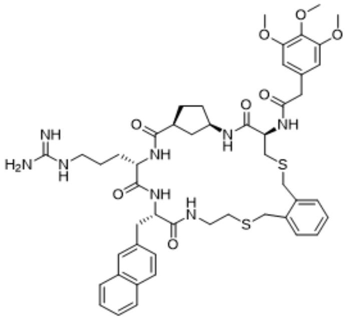
Synthesis:	Manual SPPS
Purification:	Flash chromatography Method A
HRMS (ESI-QToF) *m/z*:	$[M+H]^+$ calc. 955.42, found 955.40

**Compound:**	**s9**
Structure	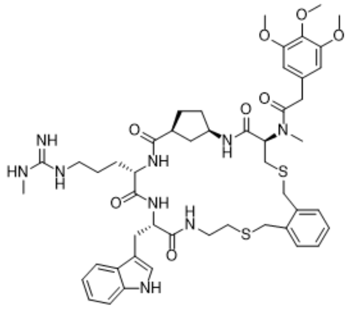
Synthesis:	Manual SPPS
Purification:	Flash chromatography Method A
HRMS (ESI-QToF) *m/z*:	$[M+H]^+$ calc. 972.45, found 972.45

**Compound:**	**s10**
Structure	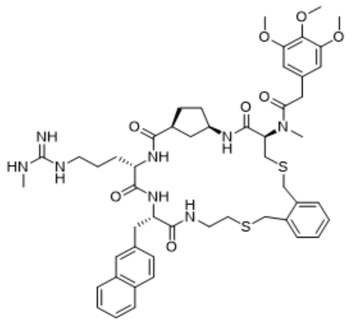
Synthesis:	Manual SPPS
Purification:	Flash chromatography Method A
HRMS (ESI-QToF) *m/z*:	$[M+H]^+$ calc. 983.45, found 983.45

**Compound:**	**s11**
Structure	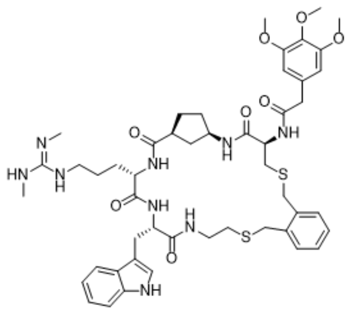
Synthesis:	Manual SPPS
Purification:	Flash chromatography Method A
HRMS (ESI-QToF) *m/z*:	$[M+H]^+$ calc. 972.45, found 972.44

**Compound:**	**s12**
Structure	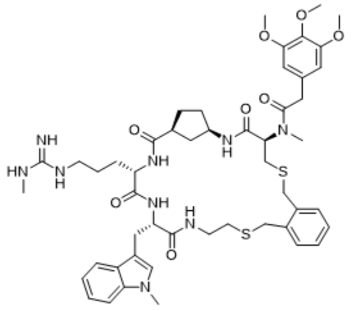
Synthesis:	Manual SPPS
Purification:	Flash chromatography Method A
HRMS (ESI-QToF) *m/z*:	[M+H]$^+$ calc. 986.46, found 986.46

**Compound:**	**s13**
Structure	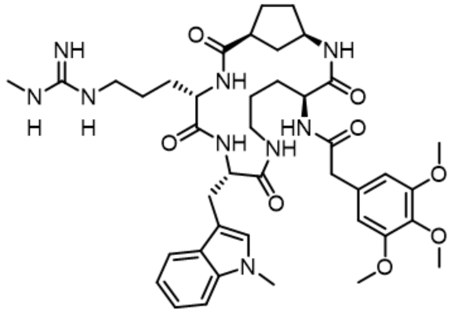
Synthesis:	Manual SPPS
Purification:	Flash chromatography Method A
HRMS (ESI-QToF) *m/z*:	[M+H]$^+$ calc. 804.44, found 804.44

**Compound:**	**s14**
Structure	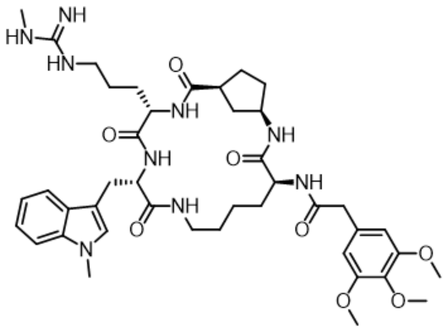
Synthesis:	Manual SPPS
Purification:	Flash chromatography Method A
HRMS (ESI-QToF) *m/z*:	[M+H]$^+$ calc. 818.46, found 818.45

**Compound:**	**s15**
Structure	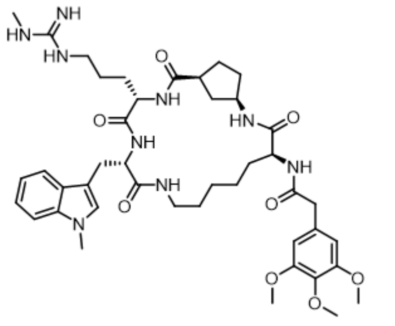
Synthesis:	Manual SPPS
Purification:	Flash chromatography Method A
HRMS (ESI-QToF) *m/z*:	$[M+H]^+$ calc. 832.47, found 832.47

**Compound:**	**s16**
Structure	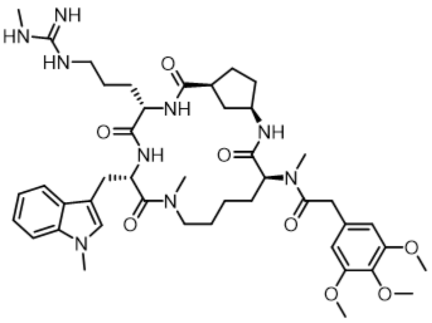
Synthesis:	Manual SPPS
Purification:	Flash chromatography Method A
HRMS (ESI-QToF) *m/z*:	$[M+H]^+$ calc. 846.49, found 846.48

**Compound:**	**s27**
Structure	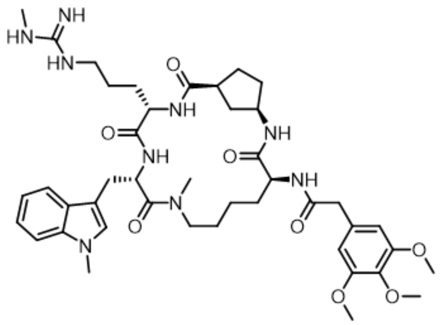
Synthesis:	Manual SPPS
Purification:	Flash chromatography Method A
HRMS (ESI-QToF) *m/z*:	$[M+H]^+$ calc. 832.47, found 832.46

**Compound:**	**s18**
Structure	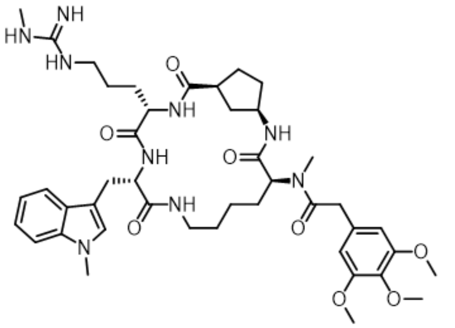
Synthesis:	Manual SPPS
Purification:	Flash chromatography Method A
HRMS (ESI-QToF) *m/z*:	[M+H]+ calc. 832.47, found 832.47

**Compound:**	**s19**
Structure	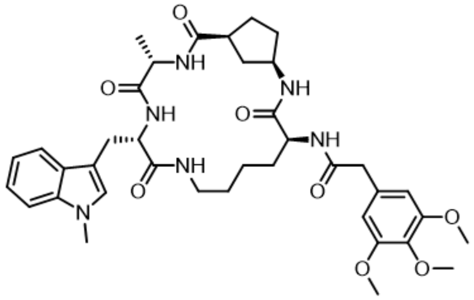
Synthesis:	Manual SPPS
Purification:	Flash chromatography Method A
HRMS (ESI-QToF) *m/z*:	[M+H]+ calc. 719.38, found 719.38

**Compound:**	**s20**
Structure	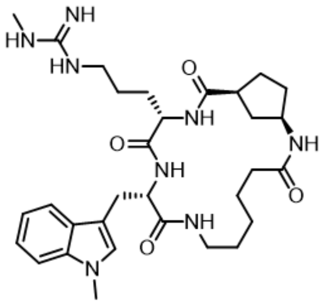
Synthesis:	Manual SPPS
Purification:	Flash chromatography Method A
HRMS (ESI-QToF) *m/z*:	[M+H]+ calc. 595.37, found 595.37

**Compound:**	**s21**
Structure	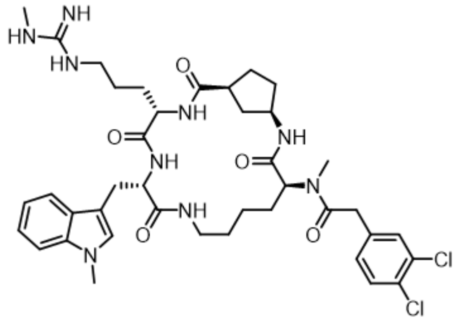
Synthesis:	Manual SPPS
Purification:	Flash chromatography Method A
HRMS (ESI-QToF) *m/z*:	$[M+H]^+$ calc. 810.36, found 810.36

**Compound:**	**s22**
Structure	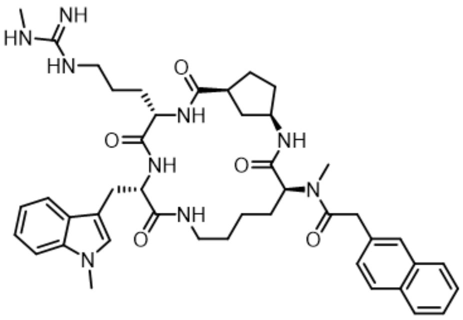
Synthesis:	Manual SPPS
Purification:	Flash chromatography Method A
HRMS (ESI-QToF) *m/z*:	$[M+H]^+$ calc. 792.46, found 792.45

**Compound:**	**s23**
Structure	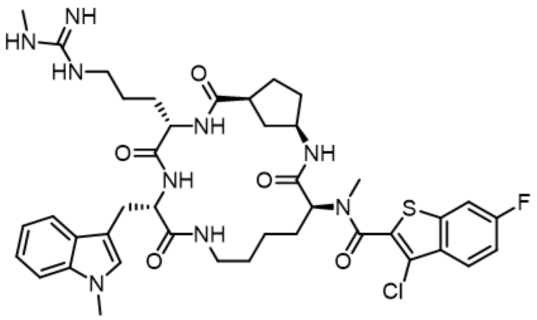
Synthesis:	Manual SPPS
Purification:	Flash chromatography Method A
HRMS (ESI-QToF) *m/z*:	$[M+H]^+$ calc. 836.35, found 836.35

10. LCMS data

Compound 1

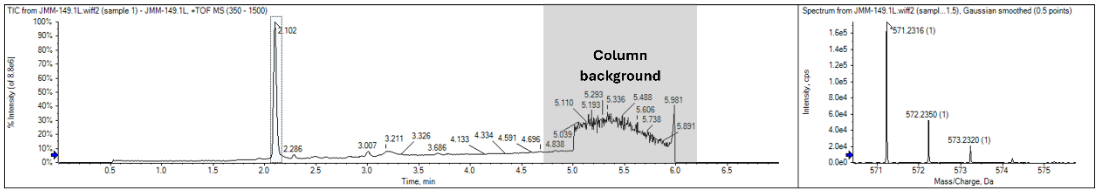

Compound 1c

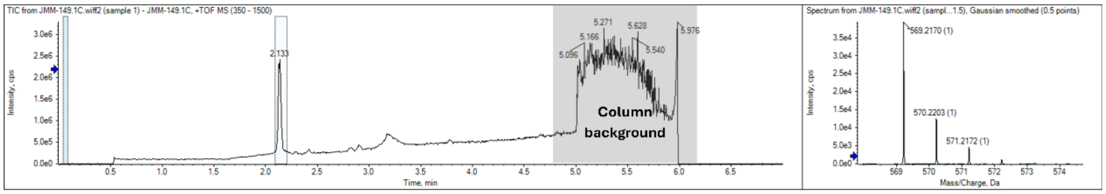

Compound 2

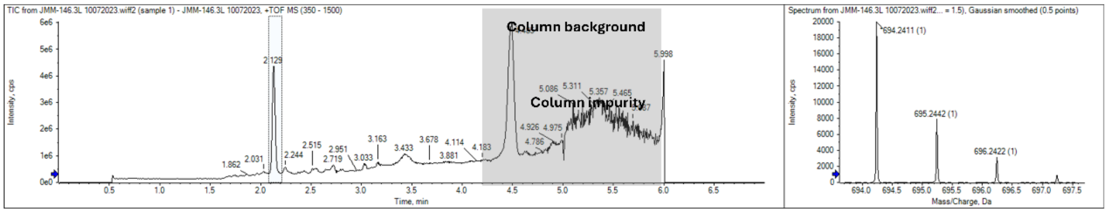

Compound 2c

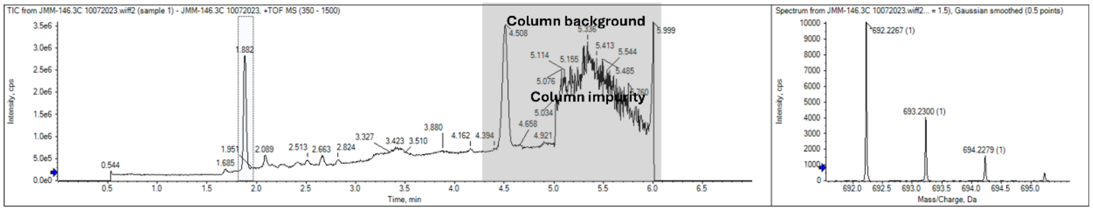

Compound 3

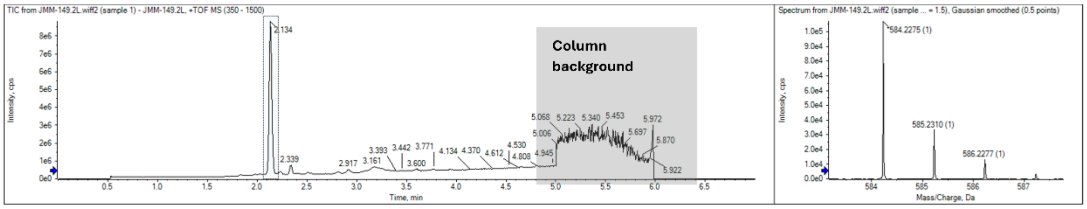

Compound 3c

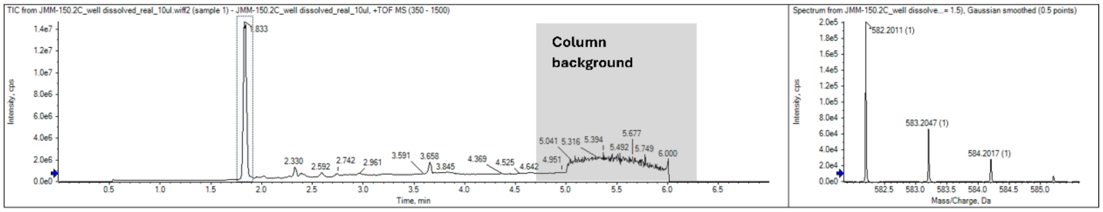

Compound 4

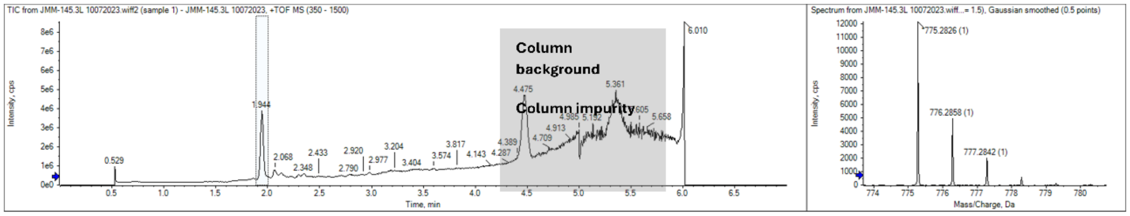

Compound 4c

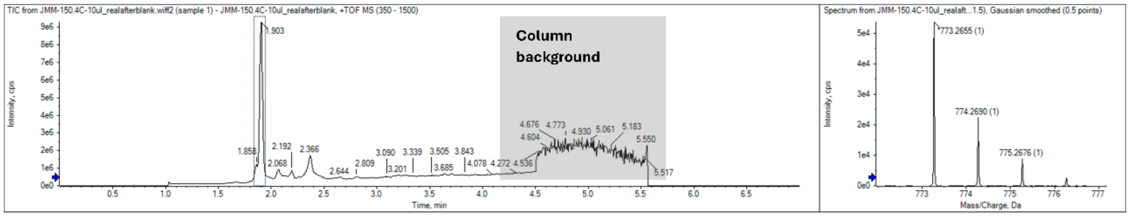

Compound 5

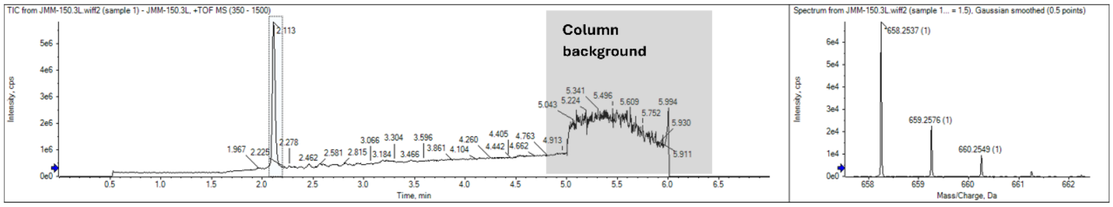

Compound 5c

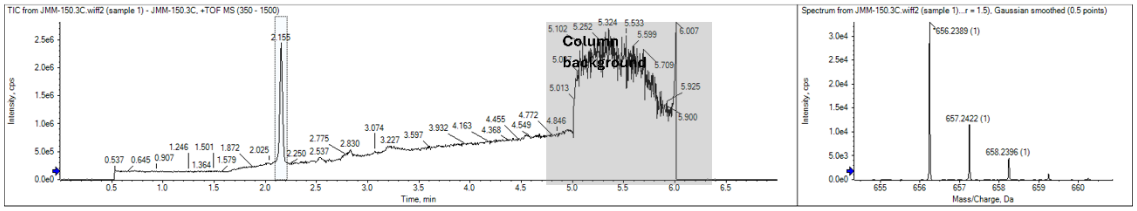

Compound 6

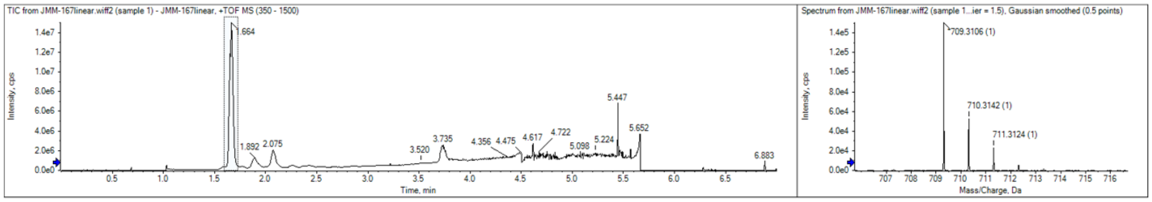

Compound 6c

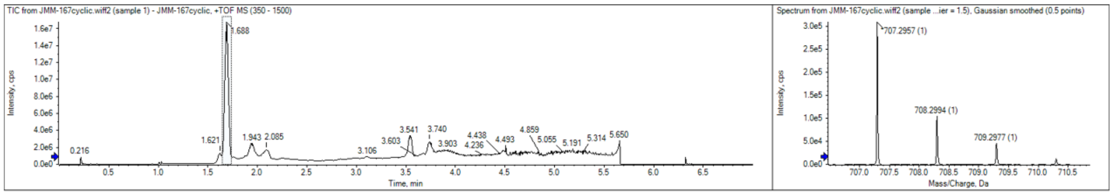

Compound 7

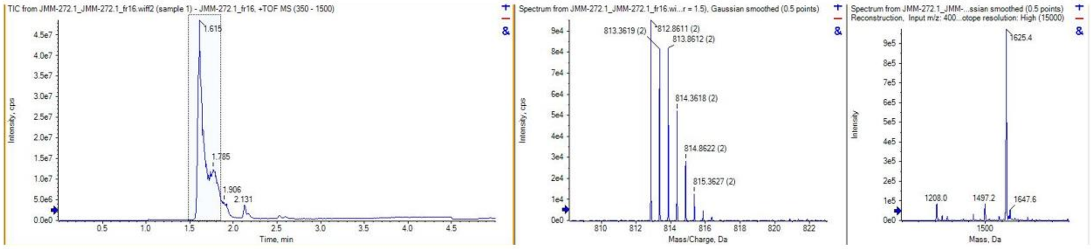

Compound 8

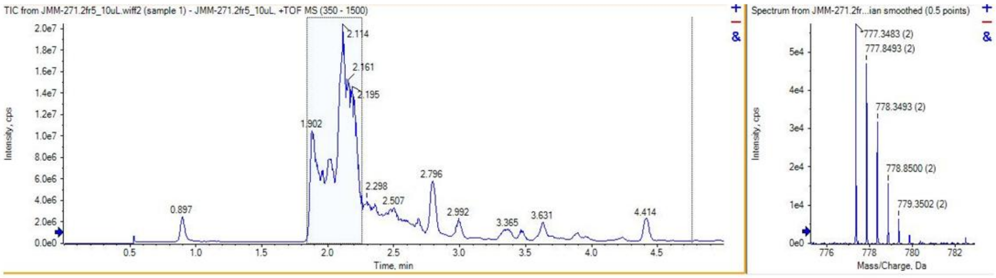

Compound 9

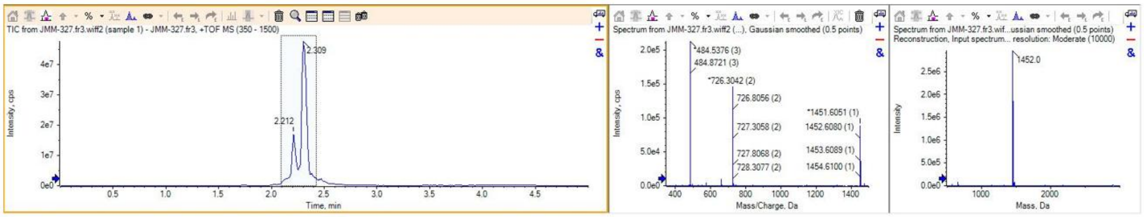

Compound 10

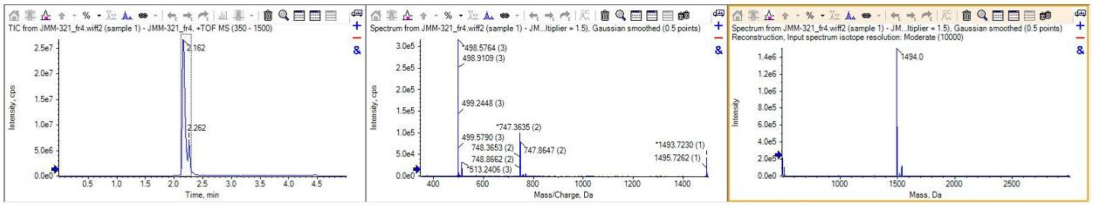

Compound 11

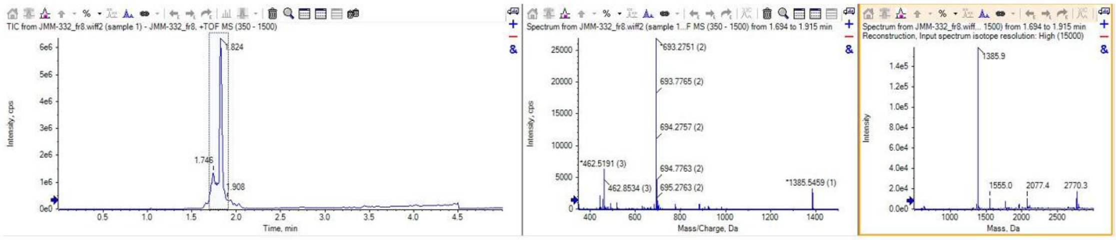

Compound 12

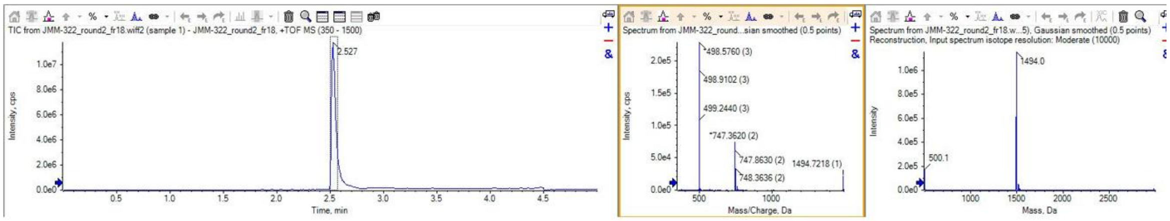

Compound 13

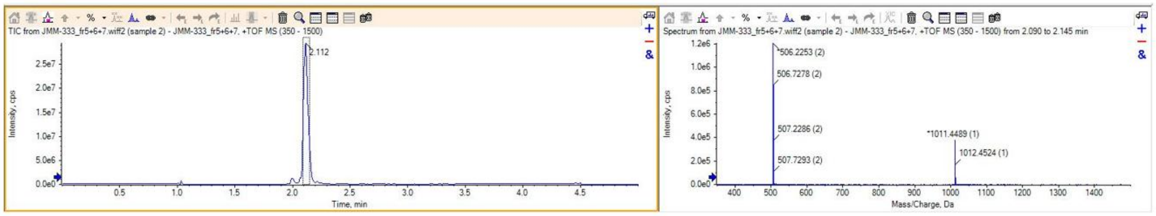

Compound 14

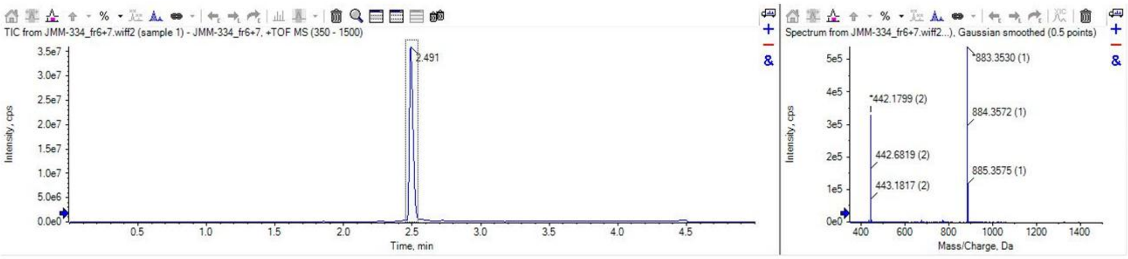

Compound 15

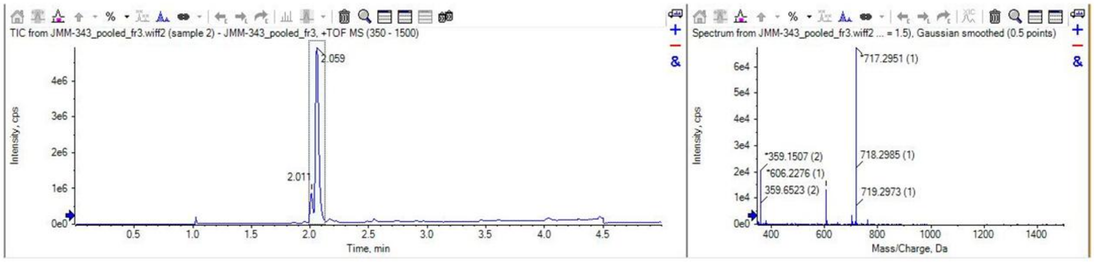

Compound 16

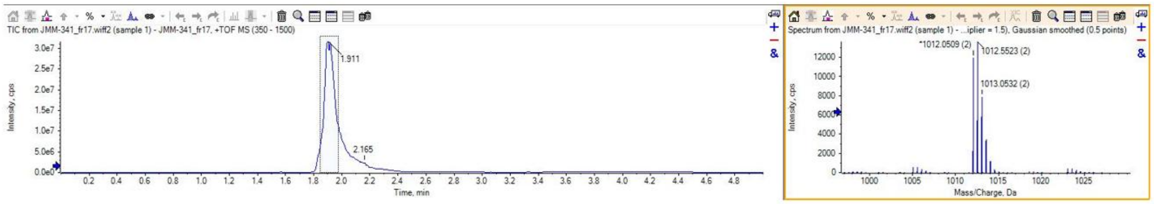

Compound 17

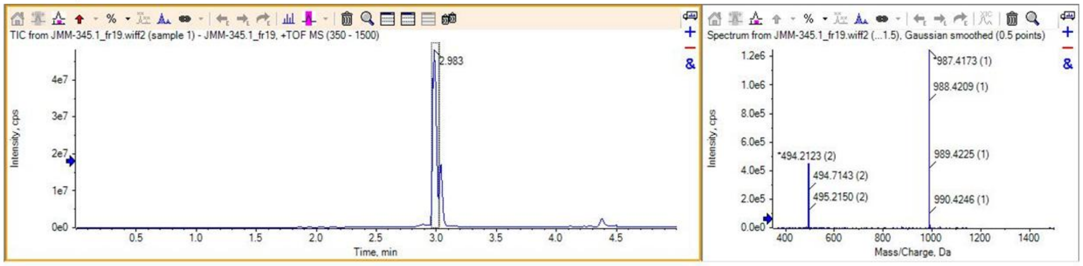

Compound 18

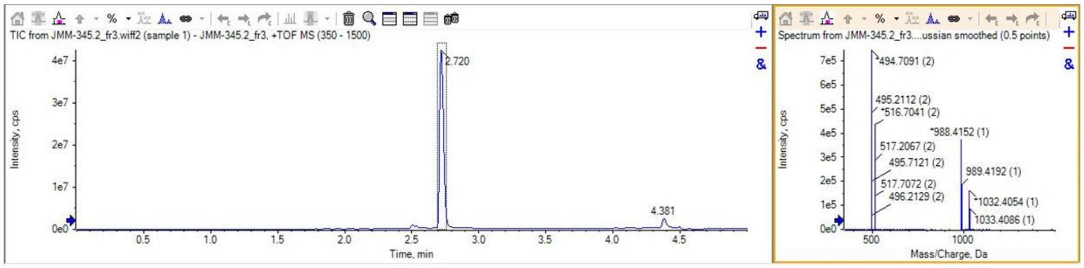

Compound 19

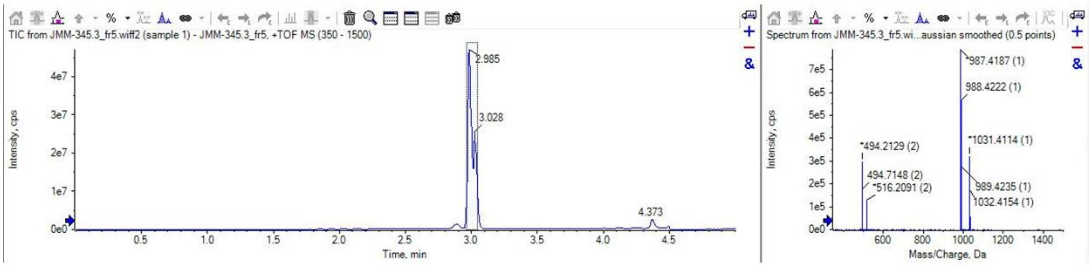

Compound 20

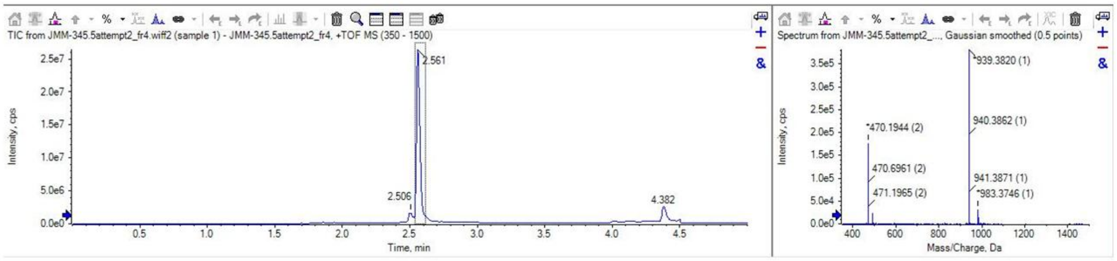

Compound 17Δ

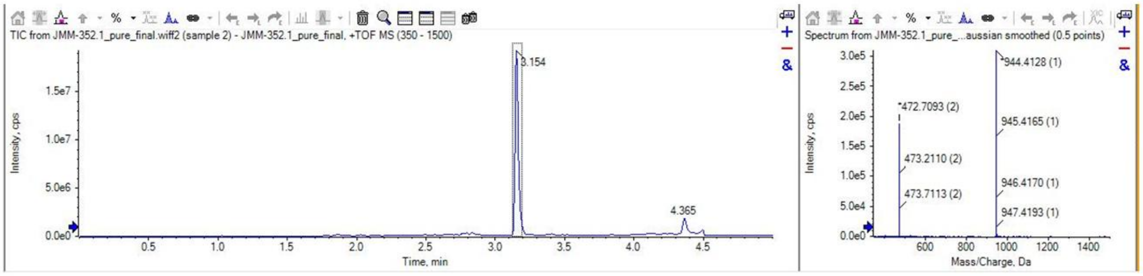

Compound 18Δ

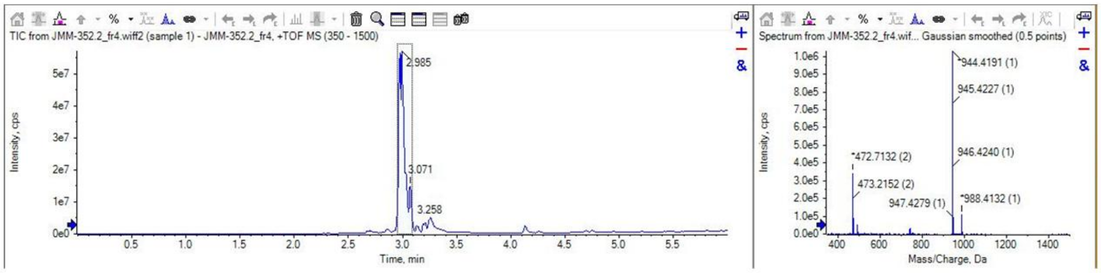

Compound 19Δ

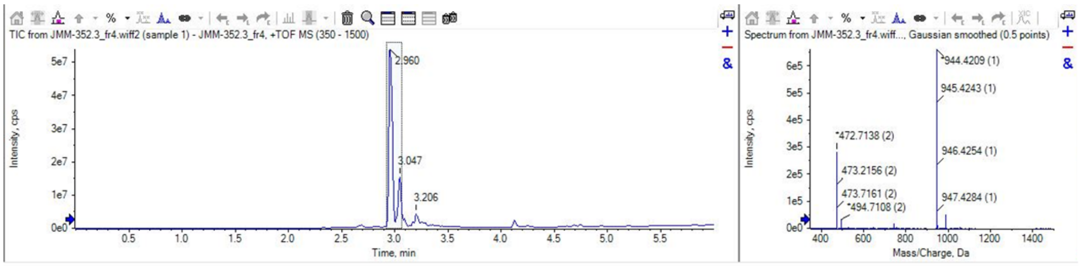

Compound 20Δ

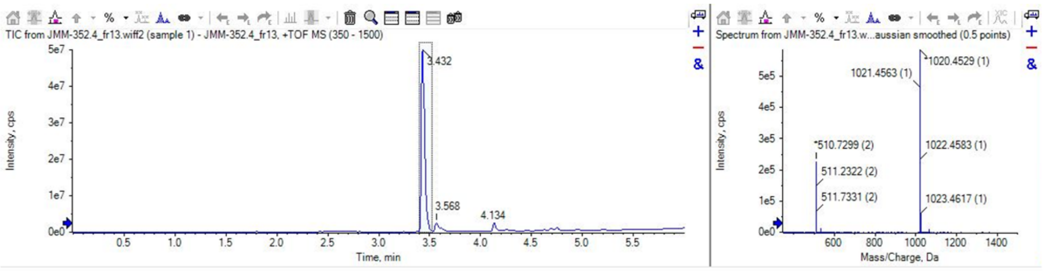

Compound 21

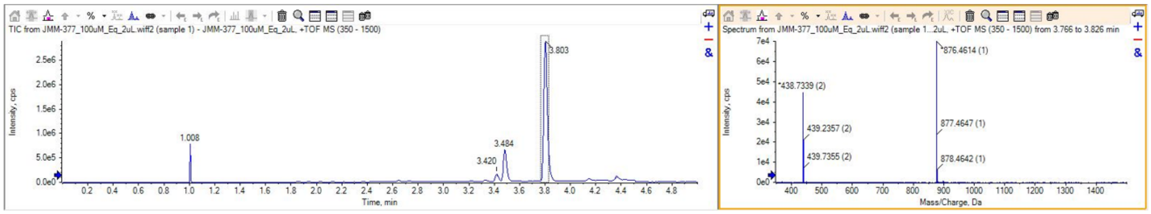

Compound 22

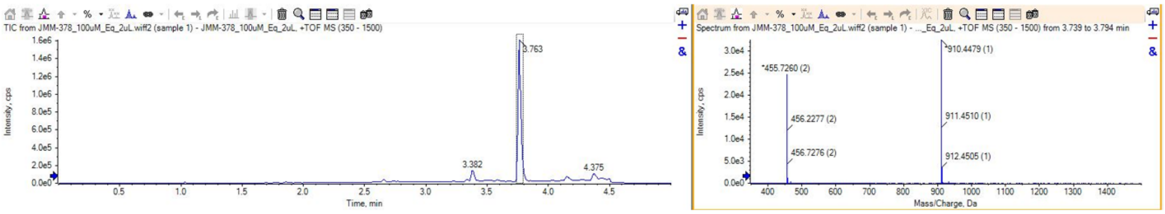

Compound 23

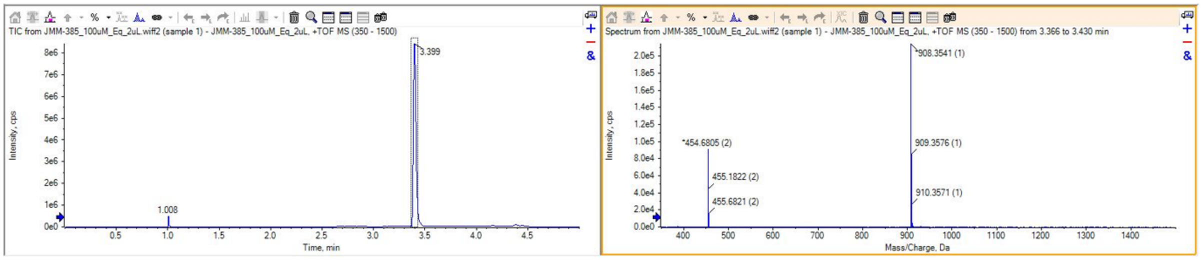

Compound 24

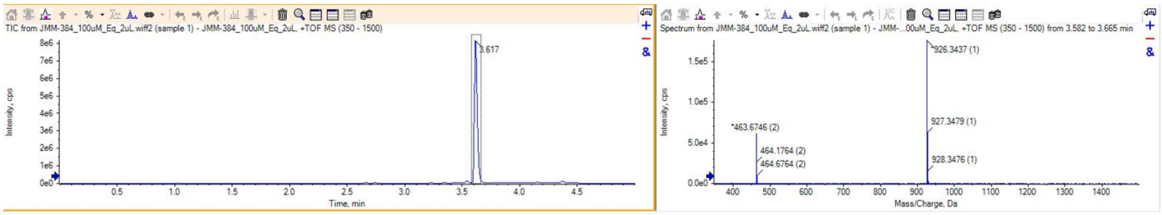

Compound 25

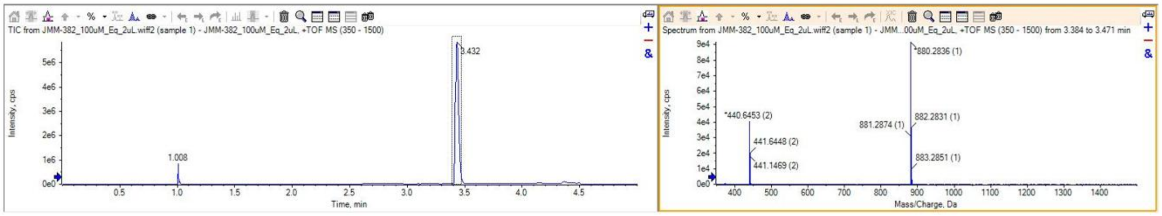

Compound 26

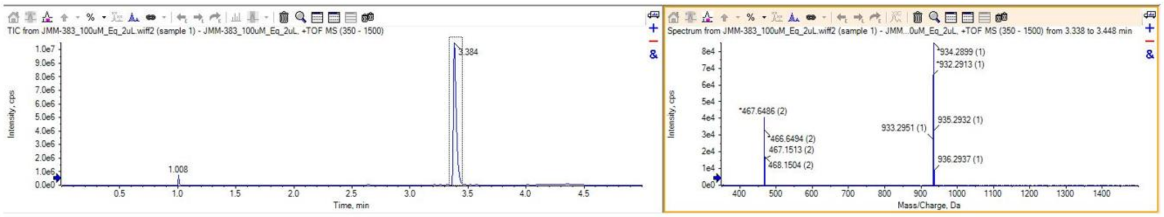

Compound 27

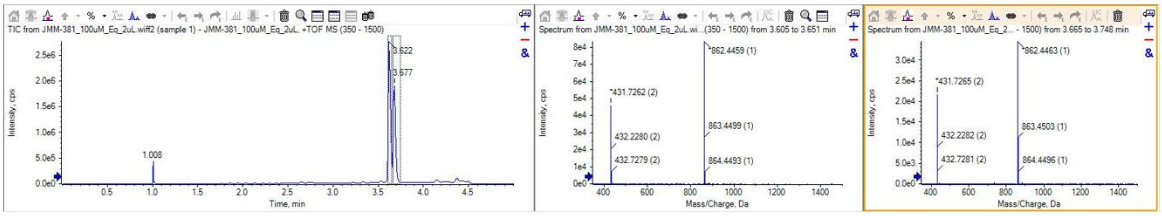

Compound 28

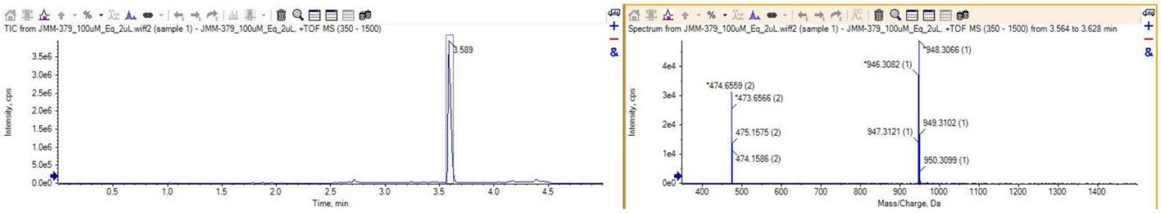

Compound 29

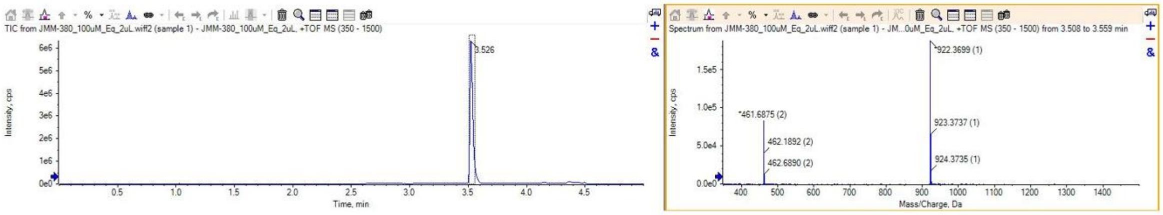

Compound 30

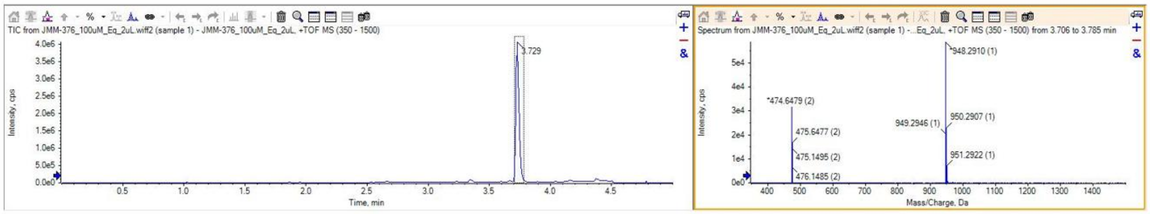

Compound s1

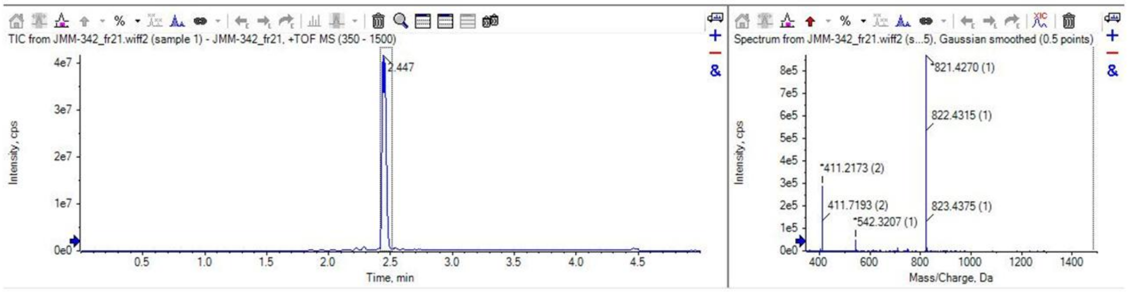

Compound s2

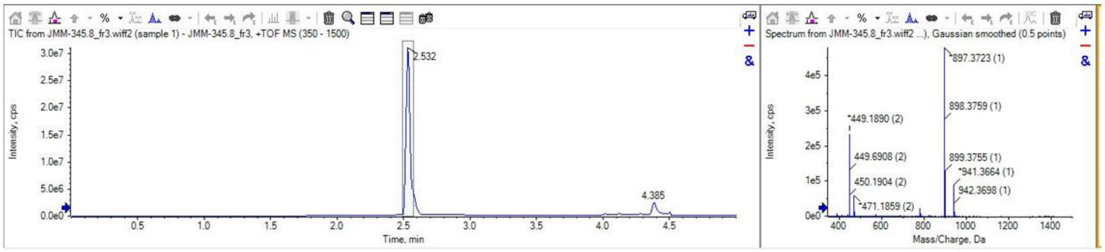

Compound s3

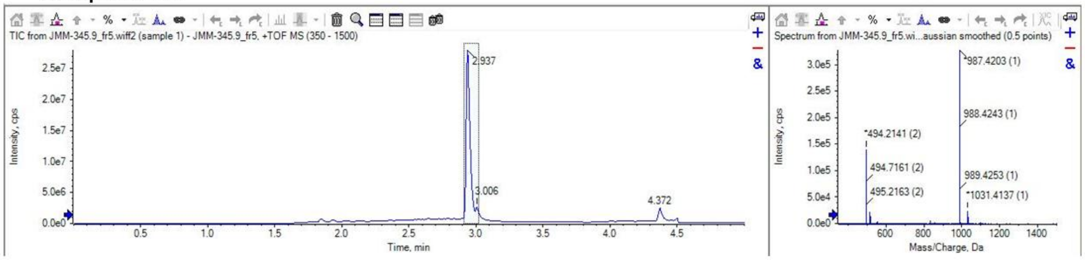

Compound s4

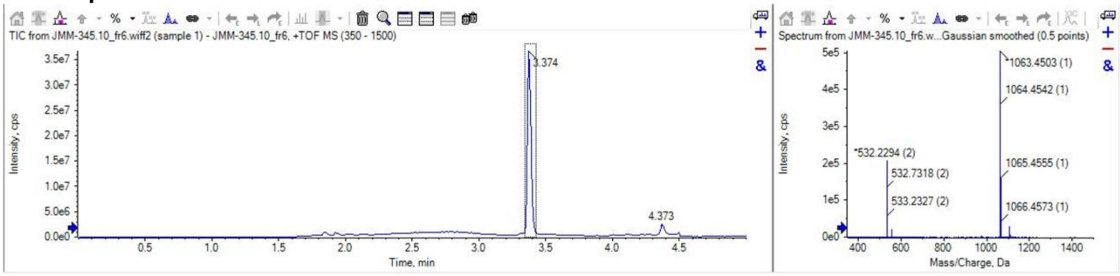

Compound s5

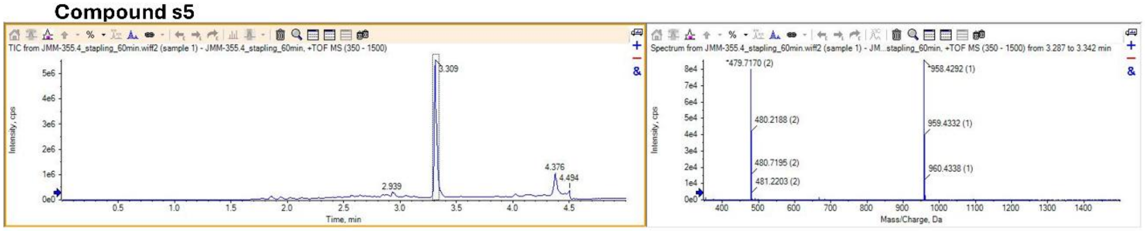

Compound s6

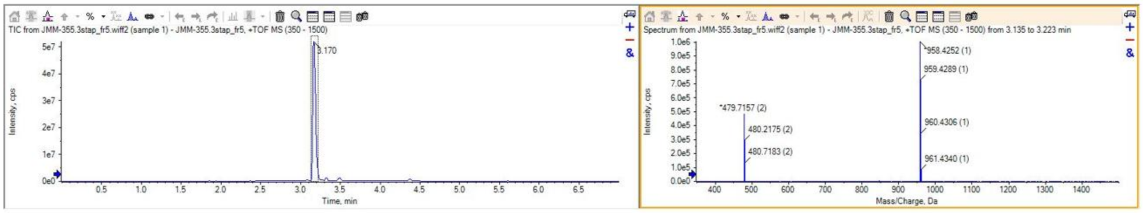

Compound s7

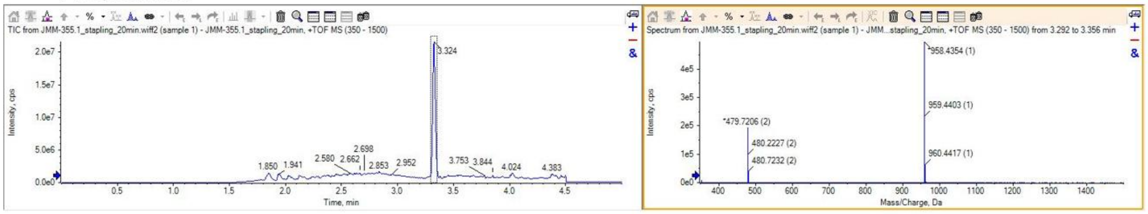

Compound s8

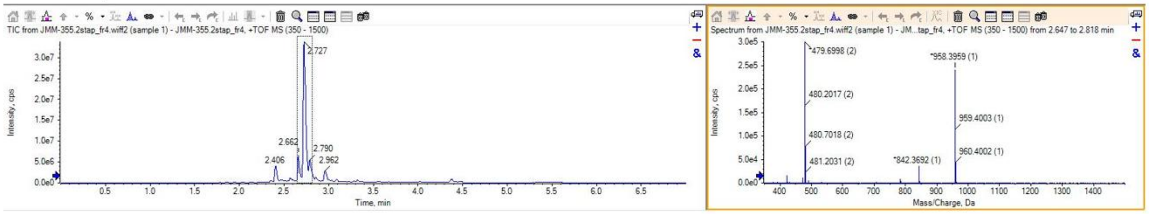

Compound s9

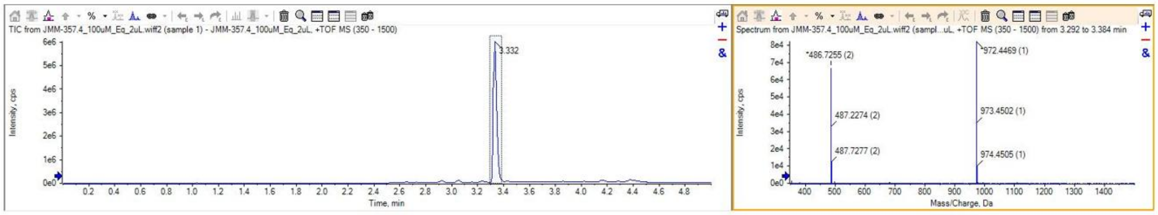

Compound s10

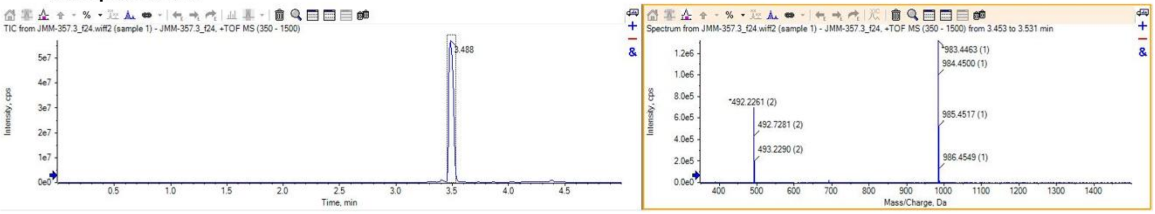

Compound s11

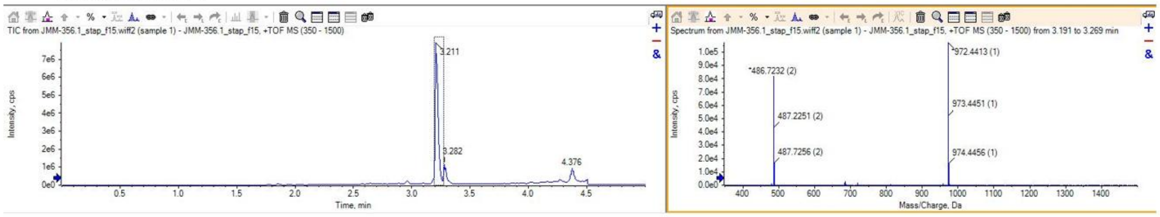

Compound s12

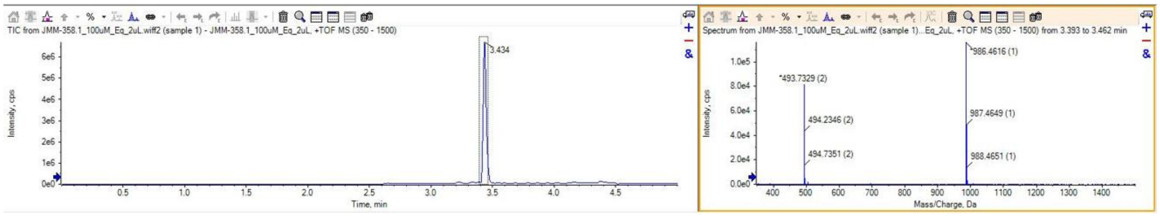

Compound s13

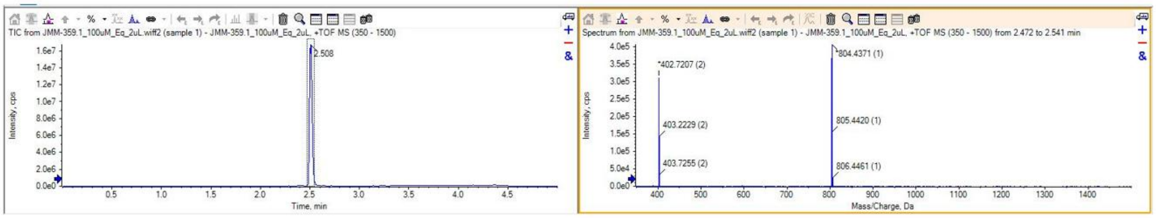

Compound s14

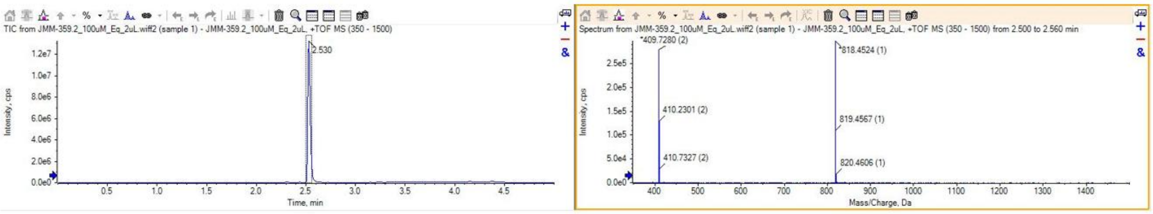

Compound s15

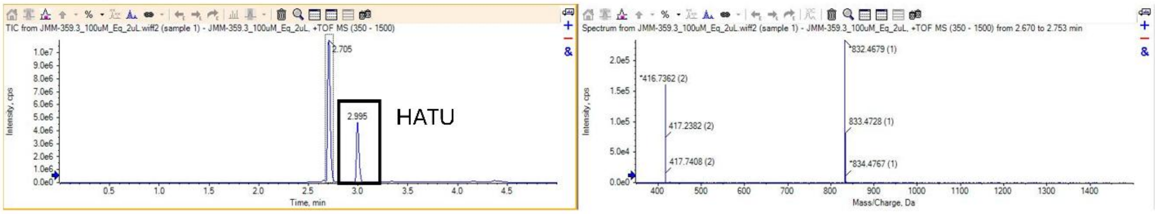

Compound s16

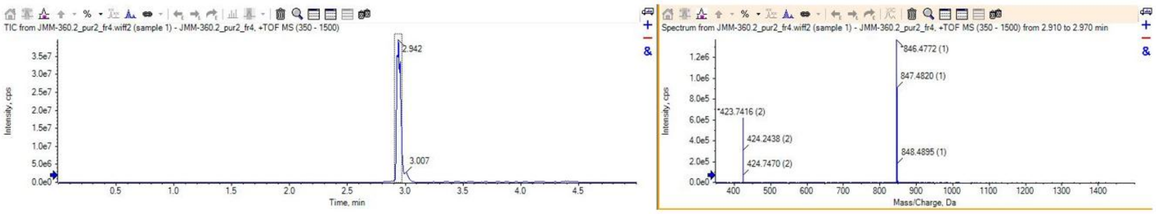

Compound s17

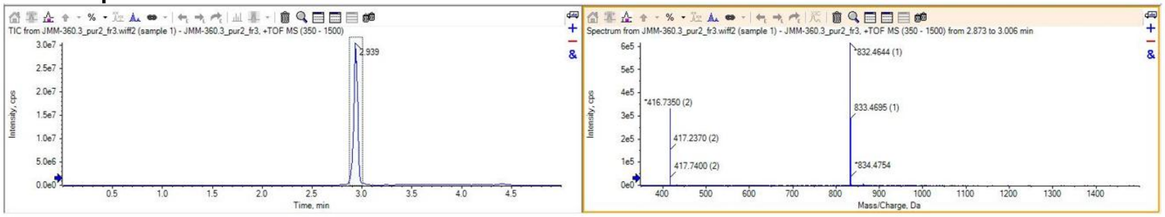

Compound s18

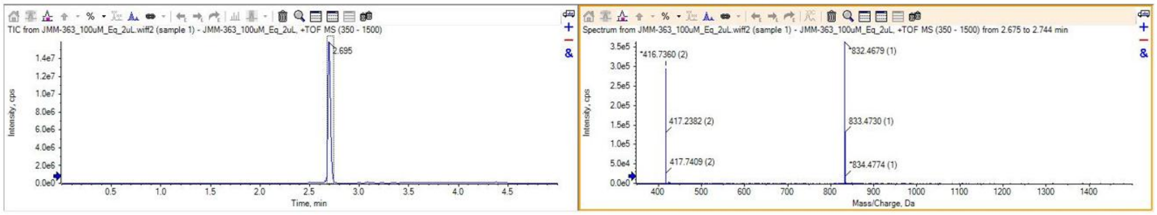

Compound s19

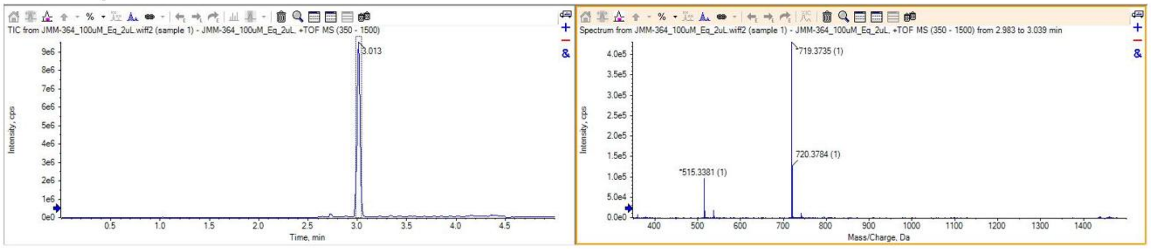

Compound s20

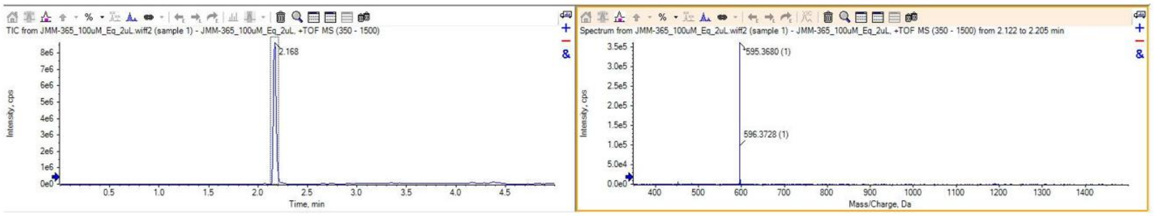

Compound s21

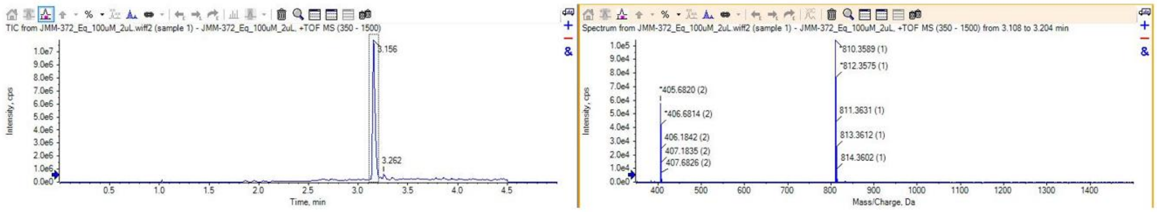

Compound s22

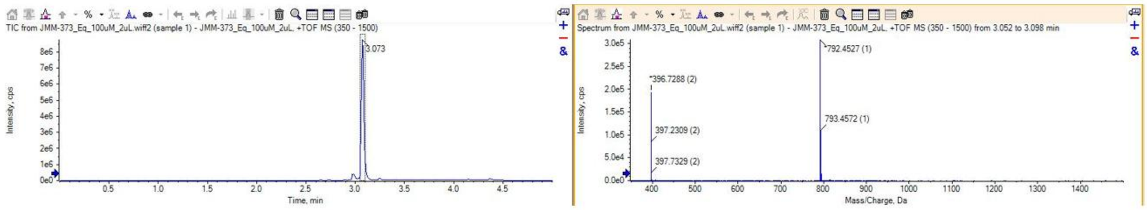

Compound s23

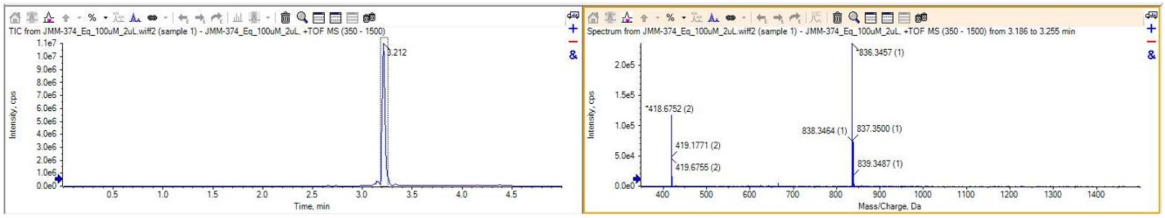

Compound s18

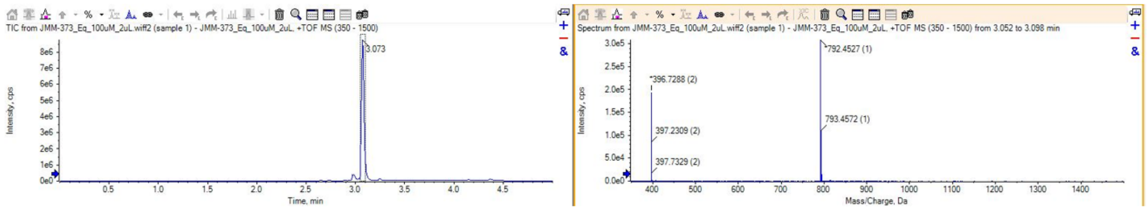

Compound s19

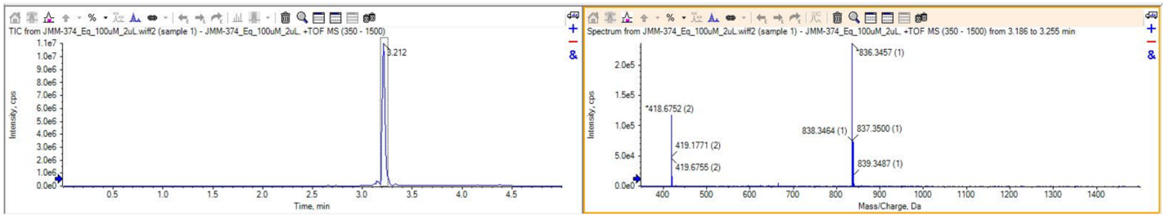

11. Supplementary References

(1) Helbik-Maciejewska, A.; Gitlin-Domagalska, A.; Glavaš, M.; Ptaszyńska, N.; Dębowski, D.; Łęgowska, A.; Rolka, K. Time-Reduced N-Methylation: Exploring a Faster Synthesis Technique. *J Org Chem* **2025**, *90* (22), 7182–7190. https://doi.org/10.1021/acs.joc.5c00083.

(2) Postma, T. M.; Albericio, F. N-Chlorosuccinimide, an Efficient Reagent for on-Resin Disulfide Formation in Solid-Phase Peptide Synthesis. *Org Lett* **2013**, *15* (3), 616–619. https://doi.org/10.1021/OL303428D/SUPPL_FILE/OL303428D_SI_001.PDF.

(3) Rappsilber, J.; Mann, M.; Ishihama, Y. Protocol for Micro-Purification, Enrichment, Pre-Fractionation and Storage of Peptides for Proteomics Using StageTips. *Nat Protoc* **2007**, *2* (8), 1896–1906. https://doi.org/10.1038/nprot.2007.261.

(4) Filius, M.; van Wee, R.; de Lannoy, C.; Westerlaken, I.; Li, Z.; Kim, S. H.; de Agrela Pinto, C.; Wu, Y.; Boons, G.-J.; Pabst, M.; de Ridder, D.; Joo, C. Full-Length Single-Molecule Protein Fingerprinting. *Nat Nanotechnol* **2024**, *19* (5), 652–659. https://doi.org/10.1038/s41565-023-01598-7.

(5) Filius, M.; Cui, T. J.; Ananth, A. N.; Docter, M. W.; Hegge, J. W.; van der Oost, J.; Joo, C. High-Speed Super-Resolution Imaging Using Protein-Assisted DNA-PAINT. *Nano Lett* **2020**, *20* (4), 2264–2270. https://doi.org/10.1021/acs.nanolett.9b04277.

(6) Chandradoss, S. D.; Haagsma, A. C.; Lee, Y. K.; Hwang, J.-H.; Nam, J.-M.; Joo, C. Surface Passivation for Single-Molecule Protein Studies. *Journal of Visualized Experiments* **2014**, No. 86. https://doi.org/10.3791/50549.

(7) Kim, S. H.; Kim, H.; Jeong, H.; Yoon, T.-Y. Encoding Multiple Virtual Signals in DNA Barcodes with Single-Molecule FRET. *Nano Lett* **2021**, *21* (4), 1694–1701. https://doi.org/10.1021/acs.nanolett.0c04502.

(8) Merz, M. L.; Habeshian, S.; Li, B.; David, J. A. G. L.; Nielsen, A. L.; Ji, X.; Il Khwildy, K.; Duany Benitez, M. M.; Phothirath, P.; Heinis, C. De Novo Development of Small Cyclic Peptides That Are Orally Bioavailable. *Nature Chemical Biology 2023 20:5* **2023**, *20* (5), 624–633. https://doi.org/10.1038/s41589-023-01496-y.

(9) Garcia Jimenez, D.; Vallaro, M.; Rossi Sebastiano, M.; Apprato, G.; D'Agostini, G.; Rossetti, P.; Ermondi, G.; Caron, G. Chamelogk: A Chromatographic Chameleonicity Quantifier to Design Orally Bioavailable Beyond-Rule-of-5 Drugs. *J Med Chem* **2023**, *66* (15), 10681–10693. https://doi.org/10.1021/acs.jmedchem.3c00823.

(10) Teuscher, K. B.; Meyers, K. M.; Wei, Q.; Mills, J. J.; Tian, J.; Alvarado, J.; Sai, J.; Van Meveren, M.; South, T. M.; Rietz, T. A.; Zhao, B.; Moore, W. J.; Stott, G. M.; Tansey, W. P.; Lee, T.; Fesik, S. W. Discovery of Potent Orally Bioavailable WD Repeat Domain 5 (WDR5) Inhibitors Using a Pharmacophore-Based Optimization. *J Med Chem* **2022**, *65* (8), 6287–6312. https://doi.org/10.1021/acs.jmedchem.2c00195.

(11) Sunseri, J.; Koes, D. R. Pharmit: Interactive Exploration of Chemical Space. *Nucleic Acids Res* **2016**, *44* (W1), W442–W448. https://doi.org/10.1093/nar/gkw287.

(12) Quartararo, A. J.; Gates, Z. P.; Somsen, B. A.; Hartrampf, N.; Ye, X.; Shimada, A.; Kajihara, Y.; Ottmann, C.; Pentelute, B. L. Ultra-Large Chemical Libraries for the Discovery of High-Affinity Peptide Binders. *Nat Commun* **2020**, *11* (1), 3183. https://doi.org/10.1038/s41467-020-16920-3.
