# Supplementary material for: Massive barcode-free chemical screenings enable the discovery of bioactive macrocycles with passive membrane permeability
Source: Nat Commun. 2026 Apr 10;17:5034. doi: 10.1038/s41467-026-71641-3 (PMC13243568; doi:10.1038/s41467-026-71641-3)
Supplement: Supplementary file 3 — Transparent Peer Review file [file 41467_2026_71641_MOESM3_ESM.pdf]

nature portfolio

# Peer Review File

# Massive barcode-free chemical screenings enable the discovery of bioactive macrocycles with passive membrane permeability

Corresponding Author: Dr Sebastian Pomplun

**This file contains all reviewer reports in order by version, followed by all author rebuttals in order by version.**

Version 0:

Reviewer comments:

Reviewer #1

(Remarks to the Author)
Pomplun and co workers report a barcode free chemical screening platform for the identification of putatively drug like target modulators. While the methodology is competently executed and supported by an elaborate workflow, the overall conceptual advance over existing chemical library–based screening and de novo sequencing approaches remains limited. The study is clearly positioned within an already well developed landscape that includes prior work by Heinis and co workers and by Gates and co workers, and the distinctions relative to these precedents are, in several instances, primarily technical rather than conceptual. In this context, the manuscript consistently employs over promotional language, particularly in the abstract, with claims that are insufficiently supported by the presented data and would require substantial moderation.
Despite the reported increase in library size, the broader generality and robustness of the platform remain questionable. This is apparent from the two proof of concept screens: the first performs well largely due to intentional pre biasing of the library, while the second yields very low hit counts, consistent with limitations commonly associated with narrow or low effective diversity libraries. The subsequent medicinal chemistry optimization following the second screen is undeniably extensive; however, this effort does not provide convincing validation of the intrinsic quality or added value of the screening platform itself, as comparable optimization trajectories have been demonstrated starting from weak micromolar hits across a wide range of unrelated screening formats. When considered in the context of related work in the field, the current study therefore appears mismatched with the scope and conceptual expectations of Nature Communications and may be more appropriately suited for a specialist or sister journal (e.g., Communications Biology or Communications Chemistry). While the platform reflects a high level of technical competence, substantial revision would be required to justify the strength of the claims and the journal placement.
Specific comments

Abstract: The statement “which typically exhibit poor drug like properties” is imprecise and misleading. Peptide macrocycles do not necessarily exhibit poor drug like properties per se; rather, they are often not inherently optimized for such properties. Given that unmodified peptides generally perform orders of magnitude worse, the current phrasing overstates the limitation and should be replaced with more accurate and nuanced language (e.g., “which typically are not optimized for drug like properties”) or explicitly clarify that the limitation refers to a lack of intrinsic optimization rather than absolute property deficits.
• Page 3, first paragraph: The abbreviation PPI is redundantly defined twice within the same paragraph, indicating insufficient editorial care.
• Page 3, third paragraph: The assertion that incorporation is limited to “a small set of unnatural residues” is outdated and factually inaccurate. Extensive genetic reprogramming enabling the incorporation of ~10 or more non canonical amino acids has already been demonstrated, particularly in mRNA display systems. If the authors intend to refer to chemical moieties beyond amino acids, this must be explicitly stated; otherwise, the text misrepresents the current state of the field.
• Page 4, first paragraph: The studies cited in References 33–36 are considerably more similar to the present work than acknowledged. While the authors attempt to distinguish their approach based on library size and implied drug likeness associated with macrocycle size, these distinctions are neither conceptually strong nor clearly articulated. As written, the contrast appears overstated and does not convincingly delineate a substantive advance.
• Page 5, Figure 1a: The data representation is inappropriate. Variable positions are discrete integers (e.g., 1, 2, 3) and should not be presented as a continuous variable implying fractional values. The current line plot is therefore misleading. Discrete data points, potentially connected by dashed lines if a visual guide is desired, would be more accurate.

• Page 5, Figure 1 caption: Several non standard abbreviations (e.g., BB for building block, HBD for hydrogen bond donor) are not defined. These should be explicitly written out at least in the figure caption, particularly given the generalist audience of Nature Communications.
• Page 5, Figure 1b: The rationale for crossing out the greyed out DNA in the "straightforward synthesis" panel is unclear and requires explicit explanation to avoid reader confusion.
• Page 5, Figure 1c: The figure omits the chemical synthesis of the library, a core component necessary for understanding the platform. Given that the authors explicitly contrast their method with biologically encoded approaches (mRNA/phage display), visualizing the chemical construction of the library is essential and its omission is a significant deficiency.
• Page 6, first paragraph: While disulfide chemistry is a logical starting point for linearization, the discussion is incomplete. Relevant recent advances in alternative linearization strategies (e.g., Gates et al.; Sando/Morimoto et al.) are not mentioned, despite being directly relevant and, in one case, already cited elsewhere in the manuscript.
• Page 7, top: As Nature Communications targets a broad readership, the PEAKS11 sequencing method must be briefly explained (at least one sentence) rather than assumed to be common knowledge.
• Page 8, Figure 2b: There is an apparent graphical error: the two cysteine residues are not connected in the middle top sequence example.
• Page 8, Figure 2d: Property calculations were performed while excluding lysine, a decision that is neither justified nor trivial. Inclusion of lysine would likely worsen key metrics (HBD count, molecular weight, logP), and given that lysine is present in the k_D measurements, its omission introduces bias. A corresponding analysis including lysine should be provided, at minimum in the Supporting Information.
• Page 9, bottom paragraph: The claim beginning with "Notably" overstates the uniqueness of the approach. Quality control of displayed peptides is also feasible in phage and mRNA display systems (e.g., single clone MALDI MS), and the claimed advantage must therefore be appropriately hedged.
• Page 10, top: The strong enrichment observed in the CAIX screen is largely a consequence of deliberate library pre biasing with sulfonamides. This highly expected outcome must be explicitly acknowledged rather than implicitly attributed to platform performance.
• Page 11, second paragraph: The comparison of k_D values to literature data is made without citation. At least one appropriate literature reference is required to support this comparison.
• Page 16: The described chemistry corresponds to what is widely known as stapling. The rationale for introducing the less common term "clipping" is unclear and should be justified or reconsidered to avoid unnecessary terminology inflation.
• Page 20, top: Claims regarding enhanced drug likeness are overstated. The drug like properties of the final lead compound arise predominantly from extensive medicinal chemistry optimization rather than from the screening platform itself. This distinction must be explicitly acknowledged or the claims substantially softened.
• Page 20, bottom: The avoidance of DNA handling is not unique to CycloSEL but applies to any purely synthetic, barcode free library. In its current form, many purported advantages are generic rather than platform specific, and it remains unclear what fundamentally distinguishes CycloSEL from other chemical libraries.
• Pages 21–22: The concluding statements are excessively promotional. The strategy is described as "straightforward," despite being technologically complex, and implied to have delivered a cell active lead, whereas this outcome resulted from extensive downstream medicinal chemistry. The discussion would be better served by emphasizing the genuine strengths—library size and access to bRo5 chemical space—rather than overstating impact.

Reviewer #2

(Remarks to the Author)
The manuscript of Pomplun and co-workers describes the construction of small, chemically diverse, non-encoded libraries of macrocyclic peptides by combinatorial split-and-pool chemistry for drug discovery using an affinity-selection MS-MS workflow. The authors produced small precursor cyclic SELs on solid support containing either 3 and 4 diversity elements flanked by cysteine residues and tested the recall rates before and after cyclisation (via cys-cys disulfides), as well as after re-linearization in reducing conditions. As these 64 and 256 member test libraries performed well with respect to recall rate after linearization, the author constructed a cyclic SEL of 16 Mio compounds, featuring 3 aminoacid diversity elements, an exocyclic N-terminal carboxylic acid disversity element, and a C-terminal lysine for conferring better ionization. The library was further subjected to affinity-based selections against a test target, CAIX, and a series hits containing the known CAIX binder phenylsulfonamide as extracyclic decorator was found to be strongly enriched. Furthermore, a "real" challenging target, WDR5 was investigated with the 16 mio CycloSEL and these selections gave rise two 2 hits, one of which could not be verified, while the other hit of ca. 100nM affinity was then investigated in detail. The resynthesized hit (without C-terminal lysine) was analyzed by BLI as 8 nM binder, while a scrambled cyclic peptide sequence did not show binding. The compound was further shown to efficiently disrupt the WDR-MLL interaction and various analogues (clipped as dithioethers rather than disulfides) produced (e.g., without C-terminal amide) and tested for membrane permeability (by PAMPA assay). The authors could show that the extracyclic decorator could be optimized for conferring permeability and an that an optimized variant (128 nM by BLI) could indeed inhibit AML cancer cell proliferation at low micromolar concentration and was stable in serum.
Taken together, the authors demonstrated the feasibility to synthesize a high quality, 16 Mio member, library of cyclic peptides and that this SEL could be successfully used in ASMS-MS for a control target and for WDR5. While only two hits were identified, notably, one of the WDR5 hits was nicely improved to obtain an active and cell-permeable inhibitor.
While the CycloSEL technology is naturally limited by sensitivity restraints, interrogating libraries of a few million members has shown to be feasible by the authors. The presented work is sound and well executed, holding strong promise on the CycloSEL technology. I can therefore recommend accepting the manuscript for publication in Nat Commun, after addressing the following points:

- I do not really like the expression "ultra-large library". What differentiates a large from an ultra-large library? Please be more specific.

- the authors have identified 2 hits for WDR, one of which was a false positive, such that hit selection for further optimization was easy. The authors should also discuss possibilities on the hit triage if there are too many hits identified by ASMS-MS.

- a C-terminal Lys was included in the large 16 Mio CycloSEL to "ensure consistent ionization and fragmentation". Will this always be necessary to include? Could the added Lys bias selection results?

- The authors should discuss about sensitivity and solubility limits.

- The author should discuss how many affinity-based selections could be performed with a synthesized CycloSEL

- In the selections, how many washing steps (only one?) were performed and how stringent was the washing. How impactful can this be on the selection outcome, i.e., is sensitivity given also for several washing steps?

- The authors describe the advantages of CycloSEL over DEL, yet they should also mention the disadvantage of non-amplifiability post-selection.

Reviewer #3

(Remarks to the Author)
This is an exciting piece of work by the Pumplun lab that really grabbed my attention when I read it. The technology presented for the synthesis and screening of cyclic peptides by affinity-selection mass-spectrometry has enormous potential because it allows large libraries of highly diverse cyclic peptides to be screened. The method enables using an unlimited number of unnatural building blocks. The authors show that this has allowed the development of membrane-permeable, target-specific peptides, which is a highly important but challenging goal in the pharmaceutical industry. Important binders identified were thoroughly analyzed with good controls (e.g. peptide with swapped key amino acids to show specificity of interaction).

I can recommend this work for publication. There are only a few aspects that I recommend to improve and that I describe below:

1. Identification of library members: the authors tested if peptides, after linearization, can be identified by MS/MS (Fig. 2c). The show that this works well. However, it was not so clear to me if the good coverage of identified peptides is because the initial libraries used were rather small (64 or 256 peptides only). Can the authors tell if they tried the same experiment with thousands or millions of peptides, and what the coverage was? Also, it is not stated in the main text what the concentrations/quantities of individual peptides in these samples was, and how these concentrations/quantities compare with those in the large libraries used later. I recommend adding this information to the main text and discuss these points.

2. Analysis of 2x 500 resin beads: When the authors released peptides from around 500 beads, they could identify an impressively high number of different peptides, suggesting a good quality of the library. I wondered what the estimated quantities of each different peptide in this experiment was and how this quantity compares to the quantity of each peptide used for the affinity selections (with the 16-mio library; e.g. analysis of input library). I recommend to add this important information to the main text.

3. Peptide identification procedure: The procedure is not fully clear for no-LC-MS experts. Do the authors first extract from the nLC-MS data all peptides in the library based on their intact mass, and compare their presence in the target sample and stre sample? Or do they analyze MS-MS data directly. I strongly recommend to walk the reader through this process by describing in a SI chapter each step with the help of an example peptides (or 2-3 examples including also a peptide that was not enriched).

4. Related to the point above is XIC peak analysis and elimination based on manual analysis (Figure 3a SI section 7.3): being not so familiar with these methods, I had difficulties to understand well the data and procedure. I recommend to discuss one spectra in more detail. E.g. what masses do we see? How can we identify the compounds based on the peaks seen? Something like this is shown in Figure 3b for peptide 7 and additional exampled in the SI. However, I could not follow the data completely. Was the MS data filtered for the expected mass of the peptide? The indicated “base peak query” of 508.688 seems too low to me for this molecule? I recommend to describe the “sequencing output” shown in Fig 3b in detail for readers who are less familiar with the specific MS or MS/MS methods.

5. BLI data in several figures: the authors have derived the KDs based on the kinetics (kon and koff). There is another way to analyze the data which is blotting the extent of binding (y-axis) relative to the applied concentrations, which is expected to give a sigmoidal curve if concentrations are shown as log values, with an inflection point at the KD. If I am doing so, much higher KD values would be obtained for most BLI data presented. I recommend to double-check the data. In particular, the curve of the higher concentrations should be close to the second highest one if the KD indicated is much lower than the highest conc. peptide injected. E.g. Fig 3b, the curve at 100 nM peptide 7 should not be higher than that at 50 nM if the Kd is 1 nM.

6. Membrane permeability: In figure 5i this is indicated as % passive membrane permeability. I recommend to determine/calculate the Papp in cm/sec, which is an absolute value and allows comparison with different molecules and across different studies.

Version 1:

Reviewer comments:

Reviewer #1

(Remarks to the Author)
The responses and revisions were made in satisfactory manner. No need for further revision.

Reviewer #2

(Remarks to the Author)
The authors have carefully and satisfyingly responded to my and as far as I could see also the other reviewers' comments and criticisms.
In my opinion, the manuscript is now acceptable for publication.

Additional comment:
I disagree with the removal of a part of the sentence on page 3 of the manuscript which was made due to the comment of reviewer 1, as it clearly showcases the potential of a purely chemical macrocyclic synthesis:
" However, these platforms are limited mostly to natural amino acids (or a small set of unnatural residues) and typically require large sequences of 8–14 residues to achieve sufficient chemical diversity"
I suggest to re-include or rephrase the sentence.

Reviewer #3

(Remarks to the Author)
The authors addressed all my questions and adjusted the manuscript accordingly. For me, the most important thing was to gain a better understanding of how the raw MS data led to the identification of the various hits. The authors have now described all steps in the SI, as well as providing examples that enable me, as an MS non-specialist, to follow their analysis and conclusions to a large extent. Other important changes are that the authors now provide detailed data on the number of molecules subjected to MS analysis in the various controls (e.g. test libraries or a sample of 500 beads), which is valuable for understanding the sensitivity and limits of the methods. They also addressed other key points, such as critically analysing the BLI data and providing membrane permeability data as absolute values.

As I wrote in my initial report, I am impressed by the strategy, which I believe has enormous potential for cyclic peptide drug development, and by the results, which culminate in well-characterised binders to two targets. I recommend publication of the work without further changes.

**Open Access** This Peer Review File is licensed under a Creative Commons Attribution 4.0 International License, which permits use, sharing, adaptation, distribution and reproduction in any medium or format, as long as you give appropriate credit to the original author(s) and the source, provide a link to the Creative Commons license, and indicate if changes were made.
In cases where reviewers are anonymous, credit should be given to 'Anonymous Referee' and the source.
The images or other third party material in this Peer Review File are included in the article's Creative Commons license, unless indicated otherwise in a credit line to the material. If material is not included in the article's Creative Commons license and your intended use is not permitted by statutory regulation or exceeds the permitted use, you will need to obtain permission directly from the copyright holder.
To view a copy of this license, visit https://creativecommons.org/licenses/by/4.0/

**Reviewer #1 (Remarks to the Author):**

Pomplun and co workers report a barcode free chemical screening platform for the identification of putatively drug like target modulators. While the methodology is competently executed and supported by an elaborate workflow, the overall conceptual advance over existing chemical library–based screening and de novo sequencing approaches remains limited. The study is clearly positioned within an already well developed landscape that includes prior work by Heinis and co workers and by Gates and co workers, and the distinctions relative to these precedents are, in several instances, primarily technical rather than conceptual. In this context, the manuscript consistently employs over promotional language, particularly in the abstract, with claims that are insufficiently supported by the presented data and would require substantial moderation. Despite the reported increase in library size, the broader generality and robustness of the platform remain questionable. This is apparent from the two proof of concept screens: the first performs well largely due to intentional pre biasing of the library, while the second yields very low hit counts, consistent with limitations commonly associated with narrow or low effective diversity libraries. The subsequent medicinal chemistry optimization following the second screen is undeniably extensive; however, this effort does not provide convincing validation of the intrinsic quality or added value of the screening platform itself, as comparable optimization trajectories have been demonstrated starting from weak micromolar hits across a wide range of unrelated screening formats. When considered in the context of related work in the field, the current study therefore appears mismatched with the scope and conceptual expectations of Nature Communications and may be more appropriately suited for a specialist or sister journal (e.g., Communications Biology or Communications Chemistry). While the platform reflects a high level of technical competence, substantial revision would be required to justify the strength of the claims and the journal placement.

We thank the reviewer for the careful reading of our manuscript and for the thoughtful comments regarding positioning, tone, and contextualization within the existing AS-MS/MS and peptide library screening landscape

While conceptual advance of this work may not lie in the individual technical components, which to a certain extend have precedents, the innovation comes from how they are integrated into a workflow that fundamentally repositions AS-MS/MS from a peptide discovery tool into a druglike macrocycle discovery engine operating in bRo5 space. Previous work has largely explored how peptide macrocycles can be decoded by MS/MS, in some cases just on a purely technical level, in other cases linked to affinity selections; here we demonstrate how MS/MS can be used to discover scaffolds that are intentionally pre-designed for downstream optimization into cell-active molecules.

We have adjusted the wording throughout manuscript to ensure that this contribution is communicated precisely and without overstating its implications.

Already in the abstract we rephrased to: …*Fully synthetic libraries offer greater flexibility in accessing broader chemical space. Leveraging recent advances in mass spectrometry based library techniques, here we report CycloSEL (Cyclic Self-Encoded Libraries), an end-to-end workflow, that screens synthetic macrocycle libraries enriched in drug-like 'beyond rule of five' features*…

We hope that in this way we better clarify the preexisiting context on which our workflow builds. In the following we have addressed all additional specific points raised by this reviewer:

Specific comments:

Abstract: The statement “which typically exhibit poor drug like properties” is imprecise and misleading. Peptide macrocycles do not necessarily exhibit poor drug like properties per se; rather, they are often not inherently optimized for such properties. Given that unmodified peptides generally perform orders of magnitude worse, the current phrasing overstates the limitation and should be replaced with more accurate and nuanced language (e.g., “which typically are not optimized for drug like properties”) or explicitly clarify that the limitation refers to a lack of intrinsic optimization rather than absolute property deficits.

We are happy to make the suggested correction in the abstract.

• Page 3, first paragraph: The abbreviation PPI is redundantly defined twice within the same paragraph, indicating insufficient editorial care.

We corrected.

• Page 3, third paragraph: The assertion that incorporation is limited to “a small set of unnatural residues” is outdated and factually inaccurate. Extensive genetic reprogramming enabling the incorporation of ~10 or more non canonical amino acids has already been demonstrated, particularly in mRNA display systems. If the authors intend to refer to chemical moieties beyond amino acids, this must be explicitly stated; otherwise, the text misrepresents the current state of the field.

We agree that recent advances in genetic code reprogramming, particularly in mRNA display systems, enable the incorporation of numerous non-canonical amino acids, and that our original wording did not adequately reflect this progress. We have therefore removed the statement from the manuscript.

The intention was not to deny the possibilities offered by display platforms, but a comparison to synthetic libraries enabling the straightforward incorporation of hundreds of different building blocks, without requiring flexizyme or enzyme reengineering. But we agree that without longer explanations it might not be justified.

• Page 4, first paragraph: The studies cited in References 33–36 are considerably more similar to the present work than acknowledged. While the authors attempt to distinguish their approach based on library size and implied drug likeness associated with macrocycle size, these distinctions are neither conceptually strong nor clearly articulated. As written, the contrast appears overstated and does not convincingly delineate a substantive advance.

We rephrased to:

*This workflow has previously been applied mainly for linear peptides, while interest in applying it to cyclic peptide libraries has been increasing with intriguing recent examples. [Refs, also the*

*reference to Morimoto/Sando was added here now] All of these studies focused on a typical peptide space, with no exploration of small macrocycles within the bRo5 space, aiming at delivering druglike compounds designed for further therapeutic developments.*

The core innovation of our work is then summarized in the following statement:

*With Cyclic Self-Encoded Libraries (CycloSEL) we show how AS-MS/MS platforms can discover small, drug-like macrocycles from fully synthetic, barcode-free, multimillion-member libraries, optimized to meet bRo5 criteria and address intracellular targets…*

Also, in our discussion we have a more detailed paragraph on previously described related methodologies to openly contextualize our workflow:

… *Because decoding relies on de novo peptide sequencing, clear fragmentation ladders are essential, which in turn requires linearization of cyclic compounds prior to MS/MS. Reported linearization methods include Edman degradation,[34,36] acidic imidazolidinone opening,[33] Pd-catalyzed ring opening,[36] thioether oxidation,[37] and disulfide reduction.[35] We chose disulfides for their robust and mild chemistry during both cyclization and linearization, and because they do not add bulk to our compact scaffolds. While convenient at the screening stage, we recognize the limitation that disulfides are prone to reductive cleavage inside cells, typically necessitating a linker replacement later…*

• Page 5, Figure 1a: The data representation is inappropriate. Variable positions are discrete integers (e.g., 1, 2, 3) and should not be presented as a continuous variable implying fractional values. The current line plot is therefore misleading. Discrete data points, potentially connected by dashed lines if a visual guide is desired, would be more accurate.

We corrected it.

• Page 5, Figure 1 caption: Several non standard abbreviations (e.g., BB for building block, HBD for hydrogen bond donor) are not defined. These should be explicitly written out at least in the figure caption, particularly given the generalist audience of Nature Communications.

We corrected it.

• Page 5, Figure 1b: The rationale for crossing out the greyed out DNA in the "straightforward synthesis" panel is unclear and requires explicit explanation to avoid reader confusion.

We added an explanation.

• Page 5, Figure 1c: The figure omits the chemical synthesis of the library, a core component necessary for understanding the platform. Given that the authors explicitly contrast their method with biologically encoded approaches (mRNA/phage display), visualizing the chemical construction of the library is essential and its omission is a significant deficiency.

We added a small schematic for the split and pool synthesis in Fig. 1c. The more detailed synthesis, including ring formation and linearization is shown in Fig. 2a.

• Page 6, first paragraph: While disulfide chemistry is a logical starting point for linearization, the discussion is incomplete. Relevant recent advances in alternative linearization strategies (e.g., Gates et al.; Sando/Morimoto et al.) are not mentioned, despite being directly relevant and, in one case, already cited elsewhere in the manuscript.

As mentioned above we describe the state of research for linearization strategies of macrocycles in our discussion. We also added the thioether oxidation with reference to Sando/Morimoto et al now:

*For large-scale screening and hit decoding, we used an affinity selection–tandem mass spectrometry (AS-MS/MS) workflow.[29] Because decoding relies on de novo peptide sequencing, clear fragmentation ladders are essential, which in turn requires linearization of cyclic compounds prior to MS/MS. Reported linearization methods include Edman degradation,[34,36] acidic imidazolidinone opening,[33] Pd-catalyzed ring opening,[36] thioether oxidation,[37] and disulfide reduction.[35] We chose disulfides for their robust and mild chemistry during both cyclization and linearization, and because they do not add bulk to our compact scaffolds. While convenient at the screening stage, we recognize the limitation that disulfides are prone to reductive cleavage inside cells, typically necessitating a linker replacement later. Depending on binding mode, this can be straightforward or highly challenging. In our WDR5 case, modeling indicated the disulfide was solvent-exposed, and indeed it tolerated several stable replacements. Ideally, future software and workflow advances should be developed to allow screening and decoding of inherently stable macrocycles without requiring any linearization elements.*

• Page 7, top: As Nature Communications targets a broad readership, the PEAKS11 sequencing method must be briefly explained (at least one sentence) rather than assumed to be common knowledge.

We added the following brief explanation: "*PEAKS11 performs de novo peptide sequencing by interpreting tandem MS/MS spectra to directly infer amino-acid sequences without relying on a database, using probabilistic scoring and fragment ion pattern recognition*."

In addition, as requested also by rev3 we added a more detailed explanation about the nanoLC-MS/MS and the decoding workflow to the supporting information Section 3.1.6.

• Page 8, Figure 2b: There is an apparent graphical error: the two cysteine residues are not connected in the middle top sequence example.

Corrected.

• Page 8, Figure 2d: Property calculations were performed while excluding lysine, a decision that is neither justified nor trivial. Inclusion of lysine would likely worsen key metrics (HBD count, molecular weight, logP), and given that lysine is present in the k_D measurements, its omission introduces bias. A corresponding analysis including lysine should be provided, at minimum in the Supporting Information.

We added the following statement to the text: *These calculations were performed excluding the C-terminal lysine, as it functions as an aiding tag for mass spectrometry and not as part of the*

*actual library design. In hits selected for further optimization, the activity of variants without lysine will need to be tested.*

Supporting this, we immediately tested the competition activity of the WDR5 hit with and without lysine, and confirmed it is not contributing to binding.

As any fixed appendix to a library, be it a big barcode or an ionization tag, the risk of it interfering positively or negatively with binding always exists and need to be assessed in the hit validation phase. We do not believe that adding property calculations including the lysine tag will be of added value to the manuscript.

• Page 9, bottom paragraph: The claim beginning with “Notably” overstates the uniqueness of the approach. Quality control of displayed peptides is also feasible in phage and mRNA display systems (e.g., single clone MALDI MS), and the claimed advantage must therefore be appropriately hedged.

In the same paragraph there is an extensive explanation on how the analysis of ~1000 beads enables a statistically representative quality control over the multimillion membered library. We respectfully disagree with this being comparable to single clone MALDI MS quality control, which is a quality control for the correct synthesis of a single peptide and not a statistically relevant library quality check.

• Page 10, top: The strong enrichment observed in the CAIX screen is largely a consequence of deliberate library pre biasing with sulfonamides. This highly expected outcome must be explicitly acknowledged rather than implicitly attributed to platform performance.

CAIX was explicitly chosen to demonstrate technical workflow validation in a predictable setting and not aiming at discovery of novel chemical matter. Our text explained that openly:

*… CAIX is particularly suitable for benchmarking library platforms due to its well-characterized preference for building blocks bearing aromatic sulfonamides, which facilitates the detection and quantification of true hits following selection…*

And*:*

*…our library contains an aryl sulfonamide directly coupled to the exocyclic macrocycle amine…*

More detailed expectations on the outcome of this affinity selection are also present in the surrounding text.

With these statements we believe that we clearly describe the fact that the CAIX selection aims at assessing whether the workflow technically works (e.g. pulling down ligands and being able to decode them, in the implemented concentrations etc.).

• Page 11, second paragraph: The comparison of k_D values to literature data is made without citation. At least one appropriate literature reference is required to support this comparison.

We added appropriate references.

Classified as Internal | Intern

• Page 16: The described chemistry corresponds to what is widely known as stapling. The rationale for introducing the less common term “clipping” is unclear and should be justified or reconsidered to avoid unnecessary terminology inflation.

The described chemistry actually has been first published and described by Peter Timmerman under the name CLIPS (Chemical Linkage of Peptides onto Scaffolds) – the corresponding paper is referenced in the section. Stapling is a term commonly more used when referring to stabilizing the alpha helical conformation of a peptide, therefore we would prefer not using it for these macrocycles to avoid any confusion.

• Page 20, top: Claims regarding enhanced drug likeness are overstated. The drug like properties of the final lead compound arise predominantly from extensive medicinal chemistry optimization rather than from the screening platform itself. This distinction must be explicitly acknowledged or the claims substantially softened.

We rephrased to: *In this study we described the technological development and benchmarking of the platform and then demonstrated how CycloSEL enables the rapid identification of potent binders for the AML driver WDR5 that via medicinal chemistry optimization could be transformed to macrocycles with passive membrane permeability and activity in leukemia cells.*

Also later in the discussion we pick up these points and explain:

*The initial hit from screening showed poor permeability (< 1% by PAMPA at 100 µM), but replacing the disulfide with a o-xylene clip and removing the C-terminal amide substantially improved this property, underscoring how starting from a bRo5 compliant library can facilitate macrocycle optimization.*

• Page 20, bottom: The avoidance of DNA handling is not unique to CycloSEL but applies to any purely synthetic, barcode free library. In its current form, many purported advantages are generic rather than platform specific, and it remains unclear what fundamentally distinguishes CycloSEL from other chemical libraries.

In the mentioned paragraph we start with mentioning and referencing other barcode free platforms. This discussion paragraph is not aimed at distinguishing cycloSEL for other barcode free platforms, but to contextualize barcode free platforms form genetically encoded platforms. To further clarify we rephrased to:

*In contrast, CycloSEL and synthetic libraries in general rely on straightforward solid-phase synthesis, which does not require DNA handling, allows organic solvents, and supports optimized reaction conditions.*

• Pages 21–22: The concluding statements are excessively promotional. The strategy is described as “straightforward,” despite being technologically complex, and implied to have delivered a cell active lead, whereas this outcome resulted from extensive downstream medicinal chemistry. The discussion would be better served by emphasizing the genuine strengths—library size and access

to bRo5 chemical space—rather than overstating impact.

We removed the statement about the platform being ‘straightforward’. We hope that the overall changes made throughout the manuscript do now better emphasize the core innovation of the work without overstating impact.

**Reviewer #2 (Remarks to the Author):**

The manuscript of Pomplun and co-workers describes the construction of small, chemically diverse, non-encoded libraries of macrocyclic peptides by combinatorial split-and-pool chemistry for drug discovery using an affinity-selection MS-MS workflow. The authors produced small precursor cyclic SELs on solid support containing either 3 and 4 diversity elements flanked by cysteine residues and tested the recall rates before and after cyclisation (via cys-cys disulfides), as well as after re-linearization in reducing conditions. As these 64 and 256 member test libraries performed well with respect to recall rate after linearization, the author constructed a cyclic SEL of 16 Mio compounds, featuring 3 aminoacid diversity elements, an exocyclic N-terminal carboxylic acid disversity element, and a C-terminal lysine for conferring better ionization. The library was further subjected to affinity-based selections against a test target, CAIX, and a series hits containing the known CAIX binder phenylsulfonamide as extracyclic decorator was found to be strongly enriched. Furthermore, a "real" challenging target, WDR5 was investigated with the 16 mio CycloSEL and these selections gave rise two 2 hits, one of which could not be verified, while the other hit of ca. 100nM affinity was then investigated in detail. The resynthesized hit (without C-terminal lysine) was analyzed by BLI as 8 nM binder, while a scrambled cyclic peptide sequence did not show binding. The compound was further shown to efficiently disrupt the WDR-MLL interaction and various analogues (clipped as dithioethers rather than disulfides) produced (e.g., without C-terminal amide) and tested for membrane permeability (by PAMPA assay). The authors could show that the extracyclic decorator could be optimized for conferring permeability and an that an optimized variant (128 nM by BLI) could indeed inhibit AML cancer cell proliferation at low micromolar concentration and was stable in serum.
Taken together, the authors demonstrated the feasibility to synthesize a high quality, 16 Mio member, library of cyclic peptides and that this SEL could be successfully used in ASMS-MS for a control target and for WDR5. While only two hits were identified, notably, one of the WDR5 hits was nicely improved to obtain an active and cell-permeable inhibitor.
While the CycloSEL technology is naturally limited by sensitivity restraints, interrogating libraries of a few million members has shown to be feasible by the authors. The presented work is sound and well executed, holding strong promise on the CycloSEL technology. I can therefore recommend accepting the manuscript for publication in Nat Commun, after addressing the following points:

We thank the reviewer for the careful reading of our manuscript and for the positive words regarding our work. We are happy to address all points raised:

- I do not really like the expression "ultra-large library". What differentiates a large from an ultra-large library? Please be more specific.

We removed the term ultra large and rephrased to the appropriate numbers.

- the authors have identified 2 hits for WDR, one of which was a false positive, such that hit selection for further optimization was easy. The authors should also discuss possibilities on the hit triage if there are too many hits identified by ASMS-MS.

The selection against CAIX exemplifies this potential problematic: a few hundred hit candidates were proposed by the sequencing outcome. While in this case looking at the sulfonamide presence could help indicating true hits from false positives, we describe the strict data analysis workflow that would be performed in absence of such knowledge. Before any resynthesis, each hit candidate spectrum is manually inspected, for peak shape, absence in the control group and quality of the fragmentation spectra. We show how In the CAIX set only true hits would have 'survived ' the data triage.

Here is the manuscript text:

*As resynthesis and testing of false positives represent a substantial challenge and waste of time and resources in any screening campaign, we assessed whether based on further data filtering we could narrow down the candidate list to true hits. We picked a number of candidates with and without CA74 and analyzed the extracted ion chromatogram in the raw data and compared it to the same extracted ion chromatogram in the control selection. Only candidates showing a reasonable peak shape and absence of the same peak in the control sample where further considered hits. This manual data inspection step indicated that all the candidates containing aryl sulfonamide CA74 had distinct peaks in the CAIX sample and were absent in the control, indicating specific target binding. The other candidate sequences, without CA74, did not pass the filtering criteria and could be discarded (**Supplementary Section 7.3**). This manual filtering step therefore can further reduce the candidate list to larger fractions of true positives.*

As seen in the case of the WDR5 hit that could not be validated as a true binder, there are instances in which a false positives still fulfills the criteria for a real hit. In comparison with any other discovery platform, individual or selection based, a true hit rate of 50% is still a very positive outcome.

- a C-terminal Lys was included in the large 16 Mio CycloSEL to "ensure consistent ionization and fragmentation". Will this always be necessary to include? Could the added Lys bias selection results?

We did perform the test library sequencing also without Lys and obtained a decreased sequence recall rate. The lysine both enhances ionization and leads to cleaner fragmentation ladders. While the lysine adds a constant modification to the library, which in specific cases might participate in binding (or hinder it), it appears as an acceptable compromise, given the advantage in detection sensitivity and sequencing quality. Compared to DNA, mRNA or entire phages linked to library members it is a very minor modification.

In our recent manuscript about barcode free small molecule libraries (https://www.nature.com/articles/s41467-025-65282-1) we worked without lysine tags. While the library scaffolds are not really comparable, in this case we had to use 10x higher per member concentrations for hit detection. Thus, in principle, the platform could potentially be optimized to

operate in absence of this tag, but it would require careful validation to weigh benefits vs limitations.

- The authors should discuss about sensitivity and solubility limits.

We expanded in the text to:

*Biotinylated CAIX was immobilized on magnetic streptavidin beads and incubated with the library (10 fmol/member, a quantity resulting in appropriate detection in the sensitivity range of the mass spectrometer). The library at this concentration (overall 160 µM) did not show signs of precipitation. For larger library sizes or different scaffolds, solubility limits, and with that maximal achievable library size would have to be individually reevaluated.*

- The author should discuss how many affinity-based selections could be performed with a synthesized CycloSEL

Typical scenario: CycloSEL-16M was synthesized on 2 g monosized Tentagel SPPS resin (loading 0.26mmol/g), yielding 326 mg of purified library (around 52% yield). The powder was converted in a 160 mM DMSO stock. For experiments at 10pM, 1 uL of this stick is used per selection well or 10uL for experiments at 100pM. Therefore, one library synthesis is capable to feed approximately from 170 to 1700 individual selection runs. Considering triplicates for selection and control it means that with our standard conditions used in this project a library can be used for ~250 controlled experiments.

We added the following text to the manuscript: *After this validation we cleaved the library, bulk purified it by reversed phase chromatography and obtained 326 mg (52% yield from a 500 µmol scale synthesis on 2 g of beads). Such quantity, using previously described screening concentrations, would enable approximately 1700 individual selection runs. Considering triplicates for target and control proteins that would enable over 250 experiments.*

- In the selections, how many washing steps (only one?) were performed and how stringent was the washing. How impactful can this be on the selection outcome, i.e., is sensitivity given also for several washing steps?

These details are given in the SI section describing the affinity selection. We use 5 washing steps (1 min. each). We did not systematically study the effect of more or less washings on the selection outcomes.

- The authors describe the advantages of CycloSEL over DEL, yet they should also mention the disadvantage of non-amplifiability post-selection.

Our discussion states: *Even with advanced mass spectrometry instrumentation, platforms like CycloSEL do not rival the sensitivity achievable through PCR and deep sequencing.*

**Reviewer #3 (Remarks to the Author):**

This is an exciting piece of work by the Pumplun lab that really grabbed my attention when I read it. The technology presented for the synthesis and screening of cyclic peptides by affinity-selection mass-spectrometry has enormous potential because it allows large libraries of highly diverse cyclic peptides to be screened. The method enables using an unlimited number of unnatural building blocks. The authors show that this has allowed the development of membrane-permeable, target-specific peptides, which is a highly important but challenging goal in the pharmaceutical industry. Important binders identified were thoroughly analyzed with good controls (e.g. peptide with swapped key amino acids to show specificity of interaction).

I can recommend this work for publication. There are only a few aspects that I recommend to improve and that I describe below:

We thank the reviewer for taking the time to read our manuscript and for the encouraging commentary on our work. We are happy to address all points:

1. Identification of library members: the authors tested if peptides, after linearization, can be identified by MS/MS (Fig. 2c). The show that this works well. However, it was not so clear to me if the good coverage of identified peptides is because the initial libraries used were rather small (64 or 256 peptides only). Can the authors tell if they tried the same experiment with thousands or millions of peptides, and what the coverage was? Also, it is not stated in the main text what the concentrations/quantities of individual peptides in these samples was, and how these concentrations/quantities compare with those in the large libraries used later. I recommend adding this information to the main text and discuss these points.

The test libraries mimic the complexity of a sample after affinity selection. While the selection starts with a very large library, only binders are retained. These samples rarely exceed a few hundred sequences. Previous reports show that high quality MS data enabling de novo sequencing of complex mixtures can be obtained for sample up to 1000-2000 sequences. Over that limit, coelution, ion suppression, instrument speed etc become limiting and the recall drops.

For the test libraries to study the sequencing behavior we inject 100 fmol per member, slightly more than used in the actual affinity selection (10 fmol/member).

2. Analysis of 2x 500 resin beads: When the authors released peptides from around 500 beads, they could identify an impressively high number of different peptides, suggesting a good quality of the library. I wondered what the estimated quantities of each different peptide in this experiment was and how this quantity compares to the quantity of each peptide used for the affinity selections (with the 16-mio library; e.g. analysis of input library). I recommend to add this important information to the main text.

According to our resin vendor Rapp Polymere, each monosized 30 uM bead can be estimated to have a capacity of $3.9x10^{-3}$ nmol. For the quality control we diluted the cleaved peptide mix to 100 fmol/member and for affinity selection to 10 fmol. We added the amounts used for QC to the main text.

3. Peptide identification procedure: The procedure is not fully clear for no-LC-MS experts. Do the authors first extract from the nLC-MS data all peptides in the library based on their intact mass,

and compare their presence in the target sample and strep sample? Or do they analyze MS-MS data directly. I strongly recommend to walk the reader through this process by describing in a SI chapter each step with the help of an example peptides (or 2-3 examples including also a peptide that was not enriched).

We added the following text to the SI Section 3.1.6:

**Summary on peptide analysis workflow**

*The analysis of the synthetic peptide library was performed using an automated workflow integrating Data-Dependent Acquisition (DDA) and the PEAKS computational suite. Upon injection into the nanoLC-MS/MS, peptides are first separated by hydrophobicity before entering the mass spectrometer. The instrument operates in DDA mode, which acts as an intelligent selector: it performs a high-resolution survey scan (MS1) to detect intact peptide masses, then immediately selects the most intense ions for fragmentation (MS/MS). To ensure high data quality and depth, the DDA settings utilize charge state screening to prioritize multi-charged peptides (*e.g., *z = +2 to +5) and intensity thresholds to filter out background noise. Furthermore, dynamic exclusion is applied to prevent the repetitive fragmentation of highly abundant peptides, allowing the instrument to "look deeper" into the library and capture lower-abundance sequences.*

*Once the raw data is acquired, PEAKS software initiates Feature Detection, a process that groups raw signals into distinct biological entities. Instead of analyzing isolated peaks, the software identifies isotopic clusters and aligns signals across their chromatographic elution time. It then performs deconvolution, mathematically collapsing multiple charge states into a single monoisotopic neutral mass. This ensures the de novo algorithm begins with a highly accurate precursor mass.*

*The core of the analysis is the de novo sequencing algorithm, which reconstructs the peptide sequence directly from the MS/MS fragment ions (primarily b and y series). By calculating the mass gaps between fragment peaks, PEAKS identifies the specific amino acids that bridge the sequence. Unlike database searching, this method does not require a reference proteome, making it ideal for verifying synthetic constructs. Each resulting sequence is assigned an Average Local Confidence (ALC) score, representing the mean confidence level across all amino acid residues in the chain. This ALC score serves as a rigorous statistical filter; an ALC of > 70 or 80% typically denotes a high-fidelity identification where the spectral evidence strongly supports the proposed primary structure.*

4. Related to the point above is XIC peak analysis and elimination based on manual analysis (Figure 3a SI section 7.3): being not so familiar with these methods, I had difficulties to understand well the data and procedure. I recommend to discuss one spectra in more detail. E.g. what masses do we see? How can we identify the compounds based on the peaks seen? Something like this is shown in Figure 3b for peptide 7 and additional exampled in the SI. However, I could not follow the data completely. Was the MS data filtered for the expected mass of the peptide? The indicated “base peak query” of 508.688 seems too low to me for this molecule? I recommend to describe the “sequencing output” shown in Fig 3b in detail for readers who are less familiar with the specific MS or MS/MS methods.

To better explain this point we added the following paragraph to the SI (Section 7.3):

After an affinity selection, a post-sequencing multi-stage manual triage was conducted to isolate true positive hits:

- **Sequence Filtering:** Proposed candidates were first filtered to ensure alignment with the library design scaffold (ZCXXXCK), where C represents fixed cysteines and K a fixed C-terminal lysine.
- **Chromatographic Validation:** For each candidate, an **Extracted Ion Chromatogram (XIC)** was generated from the raw data to confirm the presence of a well-defined peak with a characteristic chromatographic shape at the expected retention time.
- **Differential Specificity Analysis:** To eliminate non-specific binders (e.g., highly hydrophobic peptides), the exact mass of each candidate was searched in the control sample raw data. A candidate was only qualified as a "true hit" for re-synthesis if a distinct peak was detected in the target sample while being absent at the corresponding retention time in the control.

The example shown in Figure 3b is the charge state z = 2 of the hit. In the sample a clear peak is detected but not in the control. In this specific case, the exact molecular weight of the hit is 1,015.3604 Da so the charge state z = 2 => 508.688 Da.

5. BLI data in several figures: the authors have derived the KDs based on the kinetics (kon and koff). There is another way to analyze the data which is blotting the extent of binding (y-axis) relative to the applied concentrations, which is expected to give a sigmoidal curve if concentrations are shown as log values, with an inflection point at the KD. If I am doing so, much higher KD values would be obtained for most BLI data presented. I recommend to double-check the data. In particular, the curve of the higher concentrations should be close to the second highest one if the KD indicated is much lower than the highest conc. peptide injected. E.g. Fig 3b, the curve at 100 nM peptide 7 should not be higher than that at 50 nM if the Kd is 1 nM.

We agree that the BLI measurements for CAIX do not show ideal binding behavior since they do not reach saturation. BLI in some cases gives suboptimal results when lower molecular weight species are tested as opposed to protein protein intractionsWe added the following text:

*While the kinetic BLI traces did result in good fits (with $R^2$s between 0.97 and 0.99), the curves did not reach saturation at higher concentrations, indicating unideal binding behavior in these settings. However, as the experiment was meant as a confirmation of actual binding of predictable hits and not for exact $K_D$ determination of novel chemical matter, and as the $K_D$ closely fit the typical values found in literature for aryl sulfonamides binding to CAIX,* [21,44–46] *we did not further optimize the binding assay.*

The BLI binding curves for the WDR5 hit show very good binding behavior and reach saturation for the two highest concentrations. As for this hit we continued with further investigations we also confirmed those with two orthogonal assays (the BLI competition assay and the single molecule binding assay).

6. Membrane permeability: In figure 5i this is indicated as % passive membrane permeability. I

recommend to determine/calculate the Papp in cm/sec, which is an absolute value and allows comparison with different molecules and across different studies.

We have added that information to Fig. 5.
